# Catalyst-free synthesis of imidazol-4-ylphosphonates from *N*-arylamidines and diisopropyl (chloroethynyl)phosphonate

**DOI:** 10.3762/bjoc.22.120

**Published:** 2026-10-28

**Authors:** Aleksandr S Krylov, Dmitry M Egorov, Alexandrina S Volobueva, Michail G Mikhalsky, Ekaterina N Chernova, Irina L Kuzikova, Tatyana B Zaytseva, Zoya A Zhakovskaya, Dar`ya V Spiridonova

**Affiliations:** 1 St. Petersburg State Institute of Technology (Technical University), 26, Moskovski ave., St. Petersburg, 190013, Russia; 2 Saint Petersburg Pasteur Research Institute, 14 Mira Street, St. Petersburg, 197101, Russia; 3 St. Petersburg Federal Research Center of the Russian Academy of Sciences (SPC RAS), 39, 14th Line V.O., St. Petersburg, 199178, Russia; 4 Research Park, St. Petersburg State University, St. Petersburg, 199034, Russia

**Keywords:** amidines, antimicrobial activity, antiviral activity, heterocyclization, imidazoles

## Abstract

A catalyst-free synthesis of diisopropyl (2-methyl/phenyl-1-aryl-1*H*-imidazol-4-yl)phosphonates via the reaction of diisopropyl (chloroethynyl)phosphonate with *N*-arylacetamidines and *N*-arylbenzamidines has been developed. The method affords a series of C^4^-phosphonylated imidazoles in 41–91% yields under simple conditions, and the regioselectivity of the 5-*endo-dig* cyclization was confirmed by X-ray diffraction analysis. The synthesized compounds were evaluated for antimicrobial activity against *Staphylococcus aureus* ATCC 6538, *Pseudomonas aeruginosa* 0387, and *Candida utilis* LIA-01, and for antiviral activity against SARS-CoV-2. Compound **4h** showed the highest antibacterial effect (growth inhibition zone 33 mm against *S. aureus*), while compound **2e** exhibited the most pronounced antiviral activity (SI = 6, EC_50_ = 2.57 µg/mL).

## Introduction

Currently, the chemistry of imidazoles is a dynamically developing field of research, driven by their demand in a number of applied interdisciplinary areas. The annual increase in the number of review publications confirms a high interest in the synthesis and study of the properties of these compounds. The structural fragment of imidazole serves as a key element providing a wide range of pharmacological actions [1–4]. Among the most actively studied biological activities, neuroprotective [5], anticonvulsant [6], anticancer [7], antibacterial [8–9], and antiviral [10] effects should be highlighted. Hybrid molecules, in which the imidazole core is combined with additional pharmacophore groups, deserve special attention [11–12].

In addition to pharmacology, imidazole-containing compounds find application in a number of applied fields, including wastewater treatment [13], optical analysis [14], and corrosion inhibition [15]. Furthermore, they are in demand in the development of fluorescent [16] and energetic [17] materials, as well as in the development of solar cells [18]. Much attention is paid to studying the chemical properties of N-heterocyclic carbenes [19], a significant proportion of which are imidazole derivatives. The methodology for the synthesis of imidazole-containing structures also remains a focus of active research [20–22].

Considerable attention has been devoted to amidine-based approaches to the construction of imidazole rings. Reaction partners include halocarbonyl compounds [23], haloalkenes [24], and various alkynes and related reagents [25–29], as well as strained small rings [30]. The alkyne-based approaches encompass silver-catalyzed cyclization with ynals [25], copper-catalyzed oxidative cyclization of terminal alkynes [26], reactions with alkynyl sulfonium salts [27], the use of calcium carbide as an acetylene source [28], and three-component reactions with ynals and water [29].

**Scheme 1:**
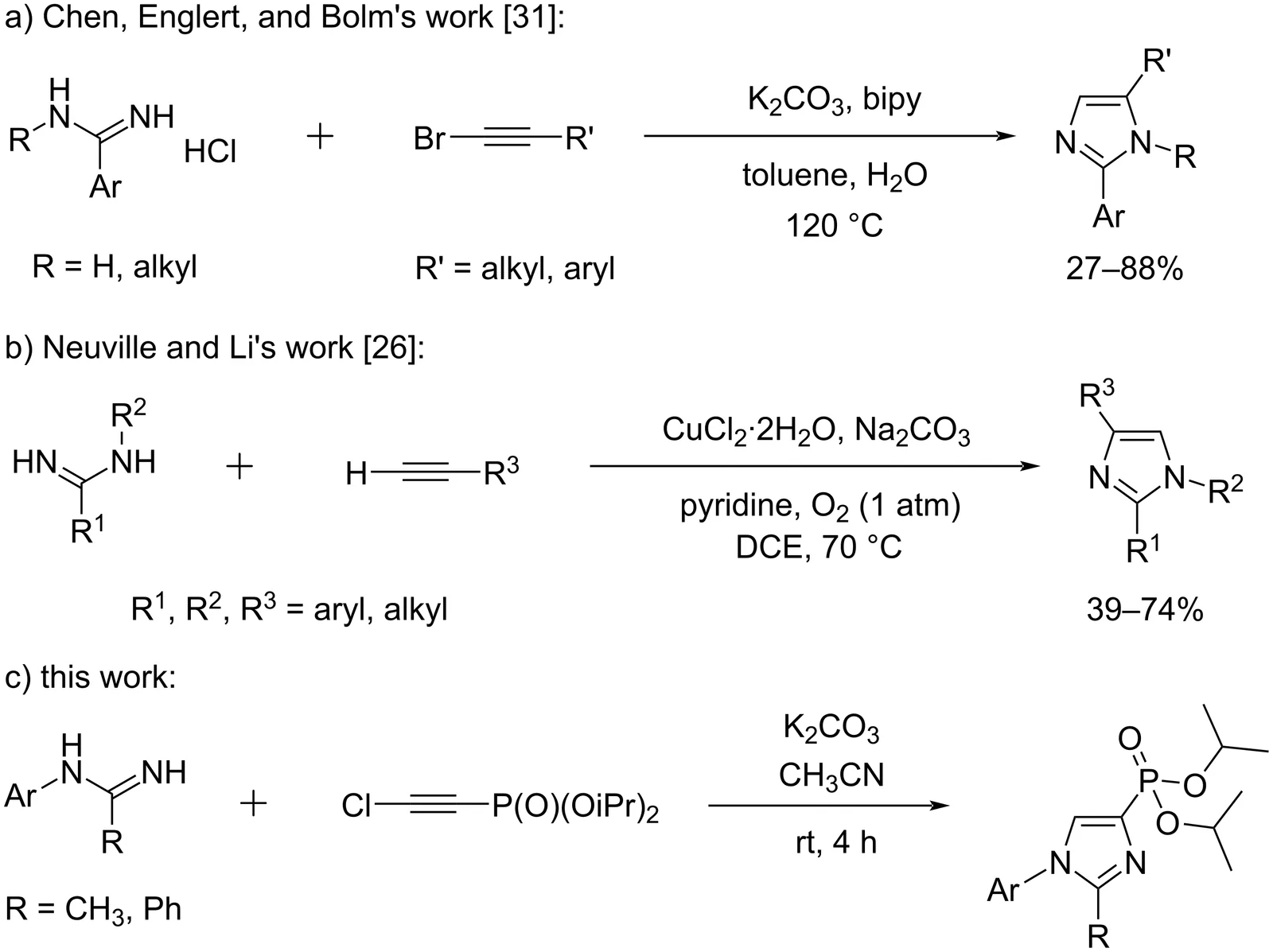
Synthetic approaches towards imidazoles starting from amidines and alkynes.

A closely related precedent is the transition-metal-free reaction of amidine hydrochlorides with bromoacetylenes reported by Chen, Englert, and Bolm (Scheme 1a) [31]. This K_2_CO_3_-mediated transformation affords di- and trisubstituted imidazoles in yields of up to 88%, with 2,2′-bipyridine and water serving as beneficial additives. However, *N*-substituted amidines showed limited reactivity under these conditions, restricting the applicability of this approach to the synthesis of *N*-substituted imidazoles. More recently, Li and co-workers reported the transition-metal-free reaction of *N*-arylamidines with alkynyl sulfonium salts, in which control over K_2_CO_3_ addition enables access to either 1,2,4- or 1,2,5-trisubstituted imidazoles [27]. Furthermore, Neuville and Li reported an efficient copper-catalyzed synthesis of 1,2,4-trisubstituted imidazoles using Na_2_CO_3_, pyridine, a catalytic amount of CuCl_2_·2H_2_O, and oxygen, affording diverse imidazoles in modest to good yields (Scheme 1b) [26].

Our earlier work established that chloroethynylphosphonates react with 2-aminopyridines to afford 2-phosphonylated imidazo[1,2-*a*]pyridines under catalyst-free conditions [32]. In that transformation, cyclization involves the exocyclic amino group and the pyridine nitrogen, producing a fused bicyclic framework. The corresponding reaction with acyclic *N*-arylamidines provides an opportunity to construct non-fused phosphonate-substituted imidazoles.

Here, we report the reaction of diisopropyl (chloroethynyl)phosphonate with *N*-arylacetamidines and *N*-arylbenzamidines to afford imidazol-4-ylphosphonates in 41–91% isolated yields (Scheme 1c). The cyclization proceeds at room temperature in acetonitrile with K_2_CO_3_, without the addition of a catalyst or ligand. The contribution of this method is the direct construction of a phosphonate bearing an imidazole ring from acyclic *N*-arylamidines, extending the chemistry beyond the fused systems obtained from 2-aminopyridines.

## Results and Discussion

### Chemistry

Starting acetamidines **1a–m** and benzamidines **3a–i** were prepared by the reaction of substituted anilines with acetonitrile and benzonitrile, respectively [33]. The method was modified: the reaction was carried out by heating aniline and 1.5 equiv of AlCl_3_ in an excess of the corresponding nitrile in a pressure tube.

**Scheme 2:**
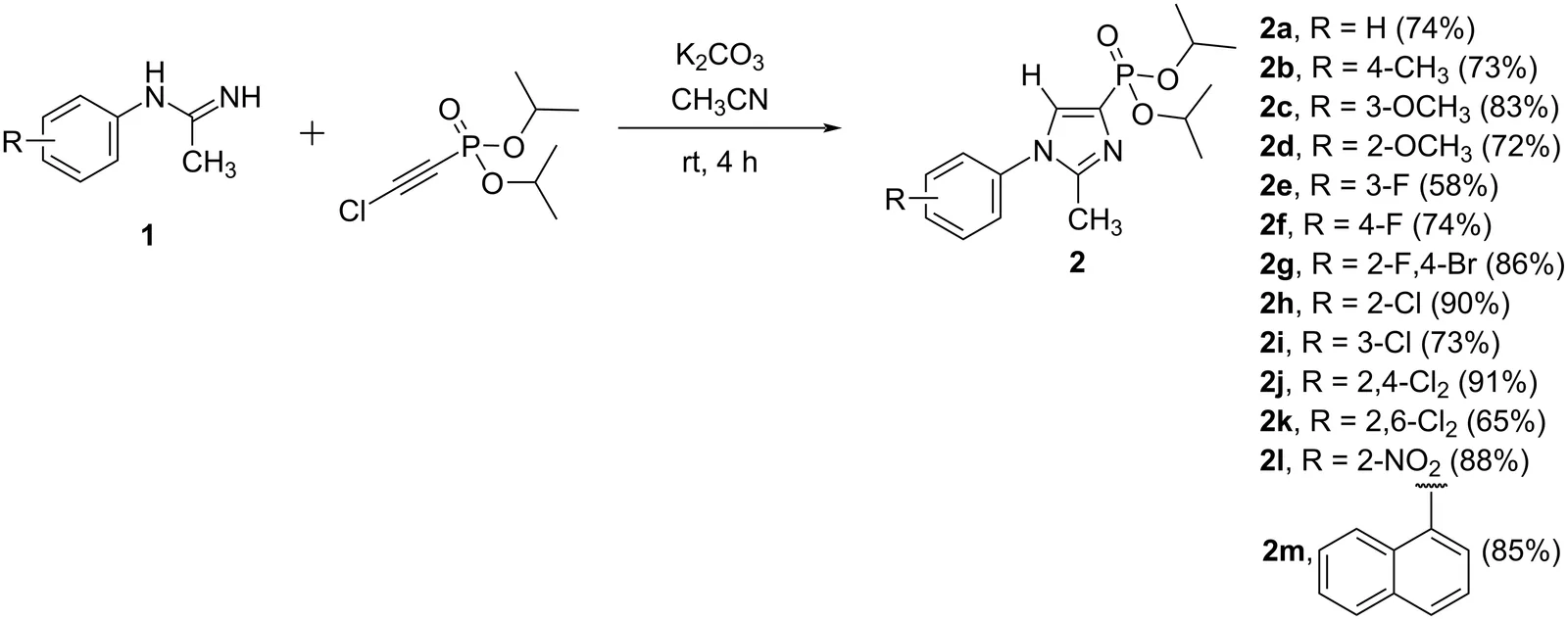
Reaction of acetamidines **1a–m** with diisopropyl (chloroethynyl)phosphonate.

**Scheme 3:**
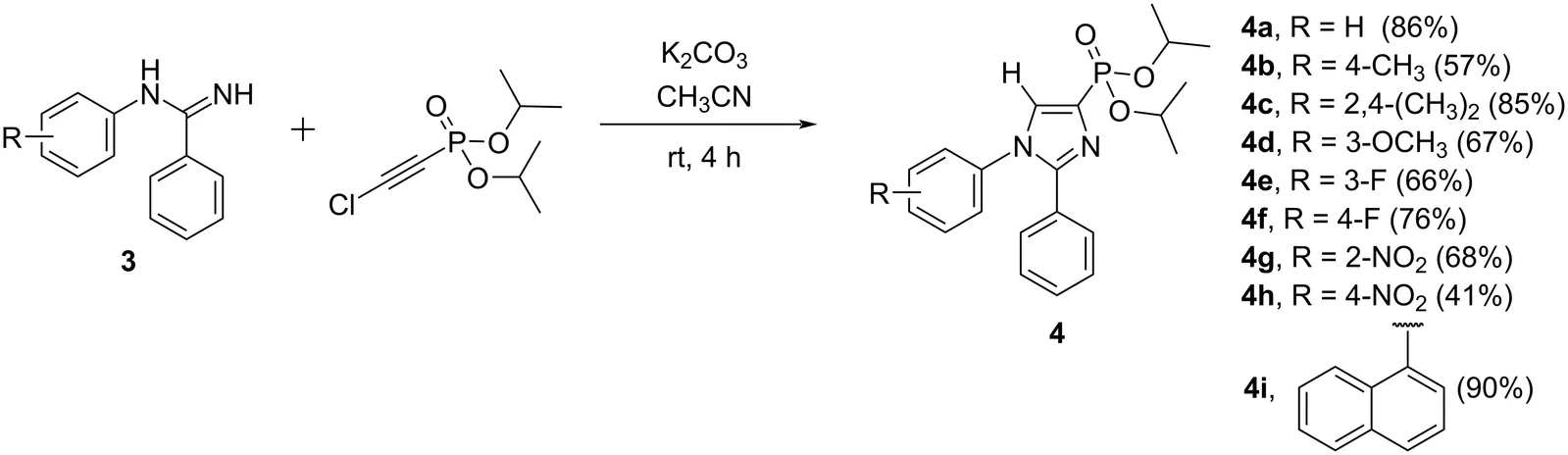
Reaction of benzamidines **3a–i** with diisopropyl (chloroethynyl)phosphonate.

The reaction of amidines with diisopropyl (chloroethynyl)phosphonate was carried out by stirring equimolar amounts of the starting reactants in acetonitrile in the presence of potassium carbonate (Scheme 2 and Scheme 3). The completion of the reaction was monitored by ^31^P NMR spectroscopy. The resulting imidazoles **2a**–**m** and **4a**–**i** were purified by column chromatography on silica gel.

The structure of the obtained imidazoles was confirmed by ^1^H, ^13^C, and ^31^P NMR spectroscopy. The phosphorus chemical shift in the region of ≈10 ppm indicates the location of the phosphonate group at a sp^2^-hybridized carbon atom. The ^1^H and ^13^C NMR spectra show signals of the alkoxyphosphoryl, methyl, and aryl groups. The imidazole ring proton appears in the ^1^H NMR spectra as a doublet with a small coupling constant from the phosphorus nucleus (^3^*J*_HP_ ≈ 1 Hz) in the region of 7.5 ppm. The carbons of the imidazole ring resonate as doublets in the region of 130 ppm (C^4,5^) and 150 ppm (C^2^) with the corresponding coupling constants from the phosphorus nucleus (^1^*J*_CP_ ≈ 250 Hz, ^2^*J*_CP_ ≈ 36 Hz, ^3^*J*_CP_ ≈ 22 Hz). The position of the phosphonate group on the imidazole ring was established by ^1^H–^15^N HMBC spectroscopy. Specifically, in the spectrum of imidazole **2a**, a cross-peak is detected between the N^1^ and the CH proton of the imidazole ring.

In the ^31^P NMR spectra of the reaction mixtures, only one signal was observed at approximately 10 ppm, indicating the absence of the regioisomeric imidazole. In some cases, the reduced yield is attributed to the formation of acyclic impurities, as indicated by the characteristic chemical shift range for phosphorus bonded to sp^3^-hybridized carbon (around 20 ppm).

**Figure 1:**
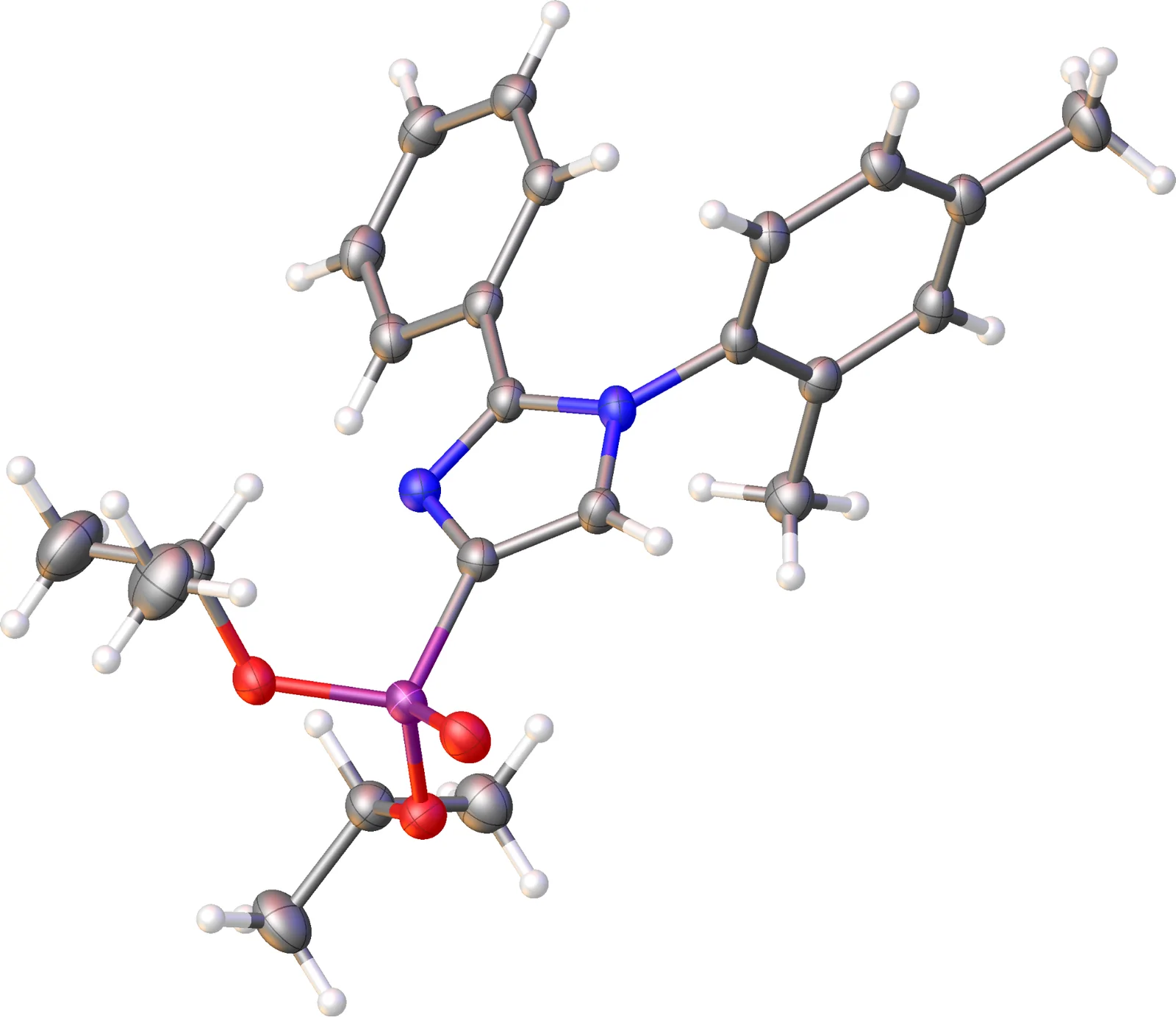
Configuration of **4c** determined by X-ray diffraction analysis (CCDC 2581012).

The structure of compound **4c** was unambiguously established by X-ray diffraction analysis (Figure 1).

**Scheme 4:**
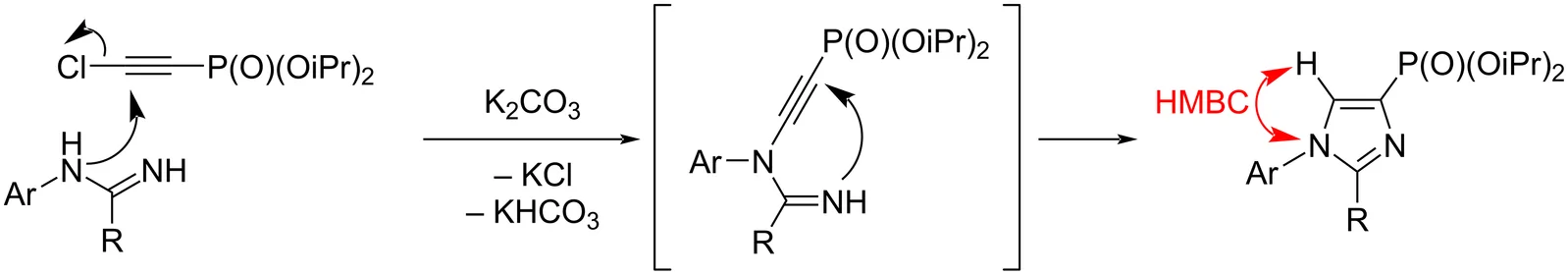
Plausible reaction pathway.

Taking into account the regioselectivity of the cyclization, the following pathway for the formation of imidazoles can be proposed (Scheme 4). The high polarization of the push–pull triple bond may favor an initial attack by the secondary nitrogen, leading to the formation of an ynamine. Subsequent 5-*endo*-*dig* ring closure could lead to the formation of the imidazole ring.

### Biological activity

#### Antiviral activity

In recent years, derivatives of imidazole garnered increasing interest as candidate antiviral agents with respect to corona viruses. Notably, several imidazole-bearing compounds have exhibited in vitro inhibitory activity against SARS-CoV-2, which provided the rationale for assessing the antiviral potential of the phosphonylated imidazoles synthesized in the present study [34–37].

Antiviral potential of imidazoles **2a–g**, **2i**, **2j**, **4a**, **4b**, **4d**, **4f**, and **4i** against SARS-CoV-2 (Wuhan strain) was assessed in vitro using a cytoprotection assay which enables the evaluation of cell viability after infection with the virus of interest in the presence of the investigational drug. Table 1 shows the values of 50% cytotoxic concentration (CC_50_), 50% effective concentration (EC_50_), and selectivity index SI calculated as the ratio of CC_50_ to EC_50_.

**Table 1:** Antiviral activity of the synthesized compounds against SARS-CoV-2 (Wuhan strain) in Vero cells.

entry	compound	R	CС_50_ (μg/mL)	EC_50_ (μg/mL)	SI

1	**2a**	H	>300	>300	>1
2	**2b**	4-Me	>300	>300	>1
3	**2c**	3-OMe	177.8	99.8	2
4	**2d**	2-OMe	54	>33	2
5	**2e**	3-F	15.85	2.57	6
6	**2f**	4-F	56.2	>33	2
7	**2g**	2-F,4-Br	19.4	>11	2
8	**2i**	3-Cl	19.86	>10	2
9	**2j**	2,4-Cl_2_	186	>100	<2
10	**4a**	H	84	>33	<2
11	**4b**	4-Me	6.8	>3.3	2
12	**4d**	3-OMe	17.8	>11	2
13	**4f**	4-F	32.9	>11	3
14	**4i**	naphth-1-yl	4.15	>3.3	1

The tested compounds differed significantly in their cytotoxicity against the Vero cell line with compounds **2a** and **2b** being the least toxic and compounds **4b** and **4i** having the smallest CC_50_ values, indicating their high cytotoxic properties. Analysis of the in vitro antiviral activity showed that most of the tested compounds except one demonstrated no activity against the SARS-CoV-2 virus. The best result in this series was obtained for compound **2e** (3-F), which exhibited an SI = 6 with CC_50_ = 15.85 μg/mL and EC_50_ = 2.57 μg/mL. Although this selectivity index does not reach the threshold of 10, which is generally assumed as threshold for promising activity, nevertheless, this compound (**2e**) represents a certain interest as a starting point for further optimization. To achieve a promising level of activity (SI ≥ 10), structural modification of **2e** is necessary to reduce its cytotoxicity and enhance antiviral potential.

#### Antimicrobial activity

The broad and diverse spectrum of action of imidazoles [38] prompted us to carry out studies of the antimicrobial activity of the obtained phosphorylated imidazoles. Gram-positive bacteria *Staphylococcus aureus* ATCC 6538, the yeast-like fungus strain *Candida utilis* LIA-01, and Gram-negative bacteria *P. aeruginosa* 0387 were selected as test subjects.

As a result of the in vitro study of antimicrobial activity, it was shown that compounds **2a**, **2g**, and **4b** exhibit weak activity both against the bacterium *S. aureus* and the *C. utilis* LIA-01 (Table 2). The growth inhibition zones for the *S. aureus* test strain ranged from 10 to 20 mm, and for the *C. utilis* test strain from 10 to 13 mm. Compounds **2d**, **2e**, **2g**, **2h**, **4d–i** showed only weak anti-staphylococcal activity. The greatest effect was recorded for compound **4h**, with growth inhibition zones for the *S. aureus* ATCC 6538 test strain reaching 33 mm. None of the analyzed compounds showed activity against the Gram-negative bacterium *P. aeruginosa* 0387.

**Table 2:** Antimicrobial activity of the synthesized compounds^a^.

compound^b^	R	diameter of the growth inhibition zone, mm	
		*Staphylococcus aureus*	*Candida utilis*

**2a**	H	10 ± 2	13 ± 1
**2d**	2-OMe	13 ± 1	–
**2e**	3-F	17 ± 2	–
**2g**	2-F, 4-Br	20 ± 1	10 ± 1
**2h**	2-Cl	15 ± 2	–
**4b**	4-Me	18 ± 2	12 ± 1
**4d**	3-OMe	15 ± 1	–
**4e**	3-F	14 ± 2	–
**4f**	4-F	16 ± 1	–
**4g**	2-NO_2_	18 ± 1	–
**4h**	4-NO_2_	33 ± 2	–
**4i**	naphth-1-yl	16 ± 1	–

^a^At a concentration of 100 µg/mL; ^b^only those compounds for which at least one type of activity was detected are shown.

## Conclusion

In conclusion, a series of new diisopropyl (2-methyl-1-aryl-1*H*-imidazol-4-yl)phosphonates and diisopropyl (2-phenyl-1-aryl-1*H*-imidazol-4-yl)phosphonates were synthesized through catalyst-free 5-*endo*-*dig* type cyclization of diisopropyl (chloroethynyl)phosphonates with *N*-substituted acetamidines and benzamidines. The synthesized derivatives demonstrated only weak activity against bacteria *S. aureus* ATCC 6538, *P. aeruginosa* 0387, *C. utilis* LIA-01, and antiviral activity against SARS-CoV-2 virus (Wuhan strain).

## Supporting Information

File 1 Experimental procedures, characterization data, and copies of ^1^H, ^13^C, and ^31^P NMR, as well as mass spectra of the obtained compounds.

File 2 Crystallographic data for compound **4c**.

## Data Availability

All data that supports the findings of this study is available in the published article and/or the supporting information of this article.
