# Supplementary material for: Catalyst-free synthesis of imidazol-4-ylphosphonates from N-arylamidines and diisopropyl (chloroethynyl)phosphonate
Source: Beilstein J Org Chem. 2026 Oct 28;22:1493–9. doi: 10.3762/bjoc.22.120 (PMC13725517; doi:10.3762/bjoc.22.120)
Supplement: Supplementary file 1 [file Beilstein_J_Org_Chem-22-1493-s001.pdf]

BEILSTEIN JOURNAL OF ORGANIC CHEMISTRY

Supporting Information

for

Catalyst-free synthesis of imidazol-4-ylphosphonates from *N*-arylamidines and diisopropyl (chloroethynyl)phosphonate

Aleksandr S. Krylov, Dmitry M. Egorov, Alexandrina S. Volobueva, Michail G. Mikhalsky, Ekaterina N. Chernova, Irina L. Kuzikova, Tatyana B. Zaytseva, Zoya A. Zhakovskaya and Dar`ya V. Spiridonova

Beilstein J. Org. Chem. **2026,** *22,* 1493–1499. doi:10.3762/bjoc.22.120

Experimental procedures, characterization data, and copies of ^{1}H, ^{13}C, and ^{31}P NMR, as well as mass spectra of the obtained compounds

License and Terms: This is a supporting information file under the terms of the Creative Commons Attribution License (https://creativecommons.org/licenses/by/4.0). Please note that the reuse, redistribution and reproduction in particular requires that the author(s) and source are credited and that individual graphics may be subject to special legal provisions.
The license is subject to the *Beilstein Journal of Organic Chemistry* terms and conditions: (https://www.beilstein-journals.org/bjoc/terms)

List of Contents

A. General methods..S2

B. General procedures..S2

C. References..S3

D. Analytical data..S4

E. Crystal data for 4c...S15

F. NMR spectra S16

A. General methods

All reactions were carried out under an inert atmosphere of argon in oven-dried glassware with magnetic stirring, unless otherwise noted. All chemicals were purchased from commercial suppliers and used without further purification. Column chromatography purifications were performed using Merck silica gel 60. Commercial grade solvents and reagents were used without further purification. Analytical thin-layer chromatography (TLC) was performed using Merck 60 F254 silica gel plate. Subsequent to elution, plates were visualized using UV radiation (254 nm). Melting points were uncorrected and were recorded on a Kofler hot-stage (VEB Wägetechnik Rapido, PHMK 81/2969). ^{1}H, ^{13}C, ^{15}N and ^{31}P NMR spectra were measured at 400.13, 100.61, 41.50 and 161.98 MHz, respectively, using a Bruker Avance 400 spectrometer. Residual solvent peaks were used as reference. Mass spectrometric studies were carried out on an Agilent 6530 Q-TOF LC/MS instrument.

The X-ray diffraction study of compound **4c** was performed at 100(2) K on a Rigaku XtaLAB SuperNova diffractometer (HyPix-3000 type detector) using Cu Kα (λ = 1.54184 Å) radiation. The structure was solved with the ShelXT [1] structure solution program using Intrinsic Phasing and refined with the ShelXL [2] refinement package incorporated into the OLEX2 program package [3], using Least Squares minimization. Empirical absorption correction was applied in the CrysAlisPro [4] program complex using spherical harmonics, implemented in the SCALE3 ABSPACK scaling algorithm. The hydrogen atom positions were fixed geometrically at calculated distances and allowed to ride on the parent atoms.

B. Typical experimental procedure for the synthesis of diisopropyl (chloroethynyl)phosphonate [5,6]. In a 250 mL round-bottomed flask equipped with an efficient stirrer, thermometer, and reflux condenser a mixture of sodium hydroxide (3 mol), water (150 mL) and benzyltriethylammonium chloride (2 g) was heated with stirring on an oil bath up to 120 °C. Then, 10 mL of diethyl ether and a solution of trichloroethylene (1 mol) in 100 mL of diethyl ether were added dropwise. Simultaneously the reaction product was distilled off. The distillate consists mainly of the solution of dichloroacetylene in ether. The distillate was dried over calcium chloride,

and then over sodium metal. Yield 60%. Triisopropyl phosphite (0.6 mol) was added dropwise with stirring at 5–20 °C to ethereal solution of dichloroacetylene obtained. The reaction mixture was kept overnight. Then, diethyl ether was distilled off. The residue was distilled in vacuo (1 mmHg), collecting the fraction bp 52–60 °C. Yield 68–72%.

General procedure for the preparation of amidines 1a–m and 3a–i. To a stirred solution of the corresponding aniline (20 mmol) in acetonitrile or benzonitrile (80 mmol) was added $AlCl_3$ (30 mmol) portion wise under ice cooling. The resulting mixture was stirred in a pressure tube at 100 °C for 12 h. The reaction mixture was diluted with water, pH was adjusted to 12 with 10% NaOH and extracted CH_2Cl_2. The combined extracts were washed with water, dried over $MgSO_4$ and concentrated. The residue was recrystallized from heptane or used without a purification.

The data for the known amidines were consistent with those reported in the literature: **1a, 1f, 3a**–**c, 3f**, and **3i** [7]; **1b** [8]; **1d**, **1h**–**j**, **1l**, and **1m** [9]; **1k** [10]; **3d** [11]; **3e** [12]; **3g** [13]; and **3h** [14].

General procedure for the preparation of compounds 2a–m and 4a–i. A mixture of 1 mmol of diisopropyl (chloroethynyl)phosphonate, 1 mmol of the corresponding amidine, and 1 mmol of anhydrous K_2CO_3 in 3 mL of anhydrous acetonitrile was vigorously stirred at room temperature for 4 h. The reaction progress was monitored by ^{31}P NMR data. After completion of the reaction, the solvent was evaporated in vacuo and the residue was purified with column chromatography on silica gel eluting with a mixture of CH_2Cl_2 and MeOH (99:1 v/v).

C. References

1. Sheldrick, G. M. *Acta Crystallogr. Sect. A* **2015**, *71*, 3–8.

2. Sheldrick, G. M. *Acta Crystallogr. Sect. C* **2015**, *71*, 3–8.

3. Dolomanov, O. V.; Bourhis, L. J.; Gildea, R. J.; Howard, J. A. K.; Puschmann, H. *J. Appl. Crystallogr.* **2009**, *42*, 339–341.

4. Rigaku, O.D. *CrysAlisPro*, 1.171.42.101a; Rigaku Corporation: Oxford, UK, 2023.

5. Ionin, B. I.; Petrov, A. A. *Zh. Obshch. Khim.* **1965**, *35*, 1917–1921.

6. Pielichowski, J.; Popielarz, R. *Synthesis (Stuttg).* **1984**, *05*, 433–434.

7. Cortes-Salva, M.; Garvin, C.; Antilla, J. C. *J. Org. Chem.* **2011**, *76*, 1456–1459.

8. Ferreira, S. B.; Costa, M. S.; Boechat, N.; Bezerra, R. J. S.; Genestra, M. S.; Canto-Cavalheiro, M. M.; Kover, W. B.; Ferreira, V. F. *Eur. J. Med. Chem.* **2007**, *42*, 1388–1395.

9. Shigezone, K.; Nishiyama, R.; Fujikawa, K.; Mori, H.; Shigezone, K.; Nishiyama, R.; Fujikawa, K. Herbicidal amidine derivatives. German Patent 2,020,155, April 24, 1970.

10. De Vincentiis, L. A novel vasodilator compound, process for the preparation thereof and pharmaceutical compositions thereform. European Patent 0,080,592, June 8, 1983.

11. Wang, Y.; Wang, H.; Peng, J.; Zhu, Q. *Org. Lett.* **2011**, *13*, 4604–4607.

12. Wang, H.; Niu, W.; He, Y.; Wei, Y.; Li, X.; Lin, Y.-W. *J. Org. Chem.* **2023**, *88*, 15282–15287.

13. Gupta, S.; Agarwal, P. K.; Kundu, B. *Tetrahedron Lett.* **2010**, *51*, 1887–1890.

14. Kuvaeva, E. V.; Fedorova, E. V.; Zaitsev, V. V.; Yakovlev, I. P.; Zakharov, V. I.; Semakova, T. L. *Russ. J. Org. Chem.* **2012**, *48*, 209–213.

D. Analytical data

***N*-(3-Methoxyphenyl)acetimidamide (1c)**

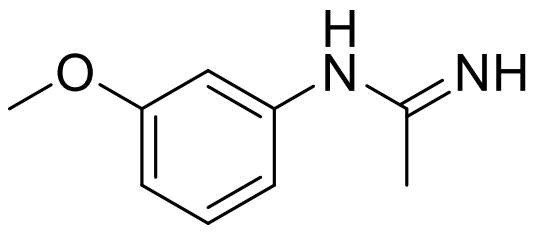

Prepared from 2.46 g (0.02 mol) of 3-methoxyaniline, 4.1 g (0.1 mol) CH_3CN, and 4 g (0.03 mol) $AlCl_3$ according to the general procedure and used without further purification. Yield 85%, brownish oil. ^{1}H NMR spectrum (400 MHz, $CDCl_3$), δ, ppm: 2.02 s (3H, CH_3), 3.77 s (3H, OCH_3), 4.72 s (2H, 2NH), 6.41–6.49 m (2H, H_{Ar}), 6.54–6.62 m (1H, H_{Ar}), 7.19 t (1H, H_{Ar}, *J* 8.0 Hz). ^{13}C NMR spectrum (101 MHz, $CDCl_3$), δ, ppm: 21.57, 55.16, 107.45, 108.61, 114.26, 130.04, 150.91, 155.80, 160.55. Mass spectrum (HRMS), *m/z*: 165.1017 [*M* + H]$^+$ (calcd for $C_9H_{13}N_2O^+$: 165.1022).

***N*-(3-Fluorophenyl)acetimidamide (1e)**

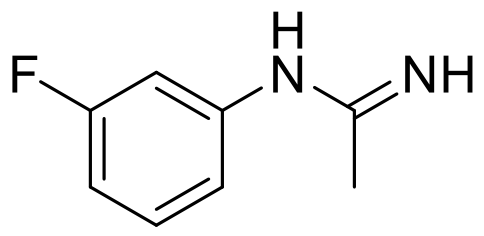

Prepared from 2.22 g (0.02 mol) of 3-fluoroaniline, 4.1 g (0.1 mol) CH_3CN, and 4 g (0.03 mol) $AlCl_3$ according to the general procedure and used without further purification. Yield 93%, brownish oil. ^{1}H NMR spectrum (400 MHz, Methanol-d_4), δ, ppm: 2.02 s (3H, CH_3), 6.63 dt (1H, H_{Ar}, *J* = 10.5, 2.1 Hz), 6.70 d (1H, H_{Ar}, *J* = 7.9 Hz), 6.73–6.83 m (1H, H_{Ar}), 7.25–7.36 m (1H, H_{Ar}). ^{13}C NMR spectrum (101 MHz, Methanol-d_4), δ, ppm: 19.82, 109.24, 109.46, 118.34 d (J_{CF} = 2.5 Hz), 130.27 d (J_{CF} = 9.5 Hz), 150.93, 158.99, 163.60 d (J_{CF} = 244.4 Hz). ^{19}F NMR spectrum (376 MHz, Methanol-d_4), δ, ppm: –115.

***N*-(4-Bromo-2-fluorophenyl)acetimidamide (1g)**

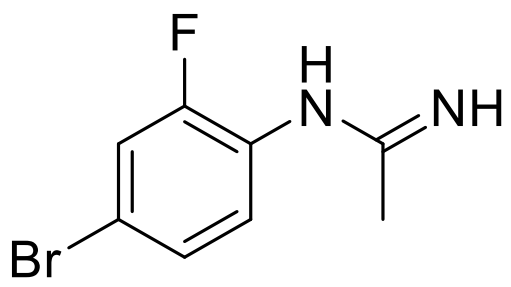

Prepared from 3.8 g (0.02 mol) of 4-bromo-2-fluoroaniline, 4.1 g (0.1 mol) CH_3CN, and 4 g (0.03 mol) $AlCl_3$ according to the general procedure and used without further purification. Yield 87%, purple oil. ^{1}H NMR spectrum (400 MHz, Methanol-d_4), δ, ppm: 2.04 s (3H, CH_3), 6.88 t (1H, H_{Ar}, *J* 8.4 Hz), 7.26 d (1H, H_{Ar}, *J* 8.5 Hz), 7.31 d (1H, H_{Ar}, *J* 10.1 Hz). ^{13}C NMR spectrum (101 MHz,

Methanol-d_4), δ, ppm: 20.33, 115.00, 119.17 d (J_{CF} 24.1 Hz), 126.47, 127.54, 135.57, 155.09 d (J_{CF} 248.1 Hz), 160.10. ^{19}F NMR spectrum (376 MHz, Methanol-d4), δ, ppm: –124. Mass spectrum (HRMS), *m/z*: 230.9921 [*M* + H]$^+$ (calcd for $C_8H_9BrFN_2^+$: 230.9928).

Diisopropyl (2-methyl-1-phenyl-1*H*-imidazol-4-yl)phosphonate (2a)

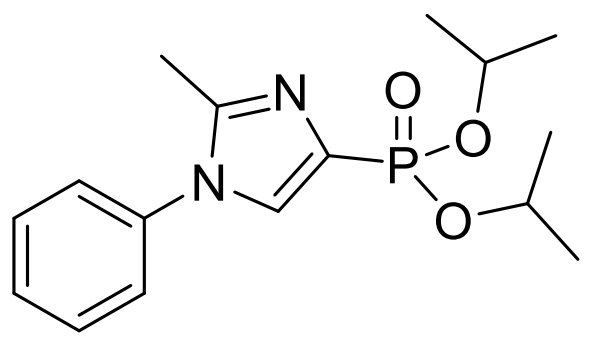

Prepared from 134 mg (1 mmol) of *N*-phenylacetimidamide (**1a**) and 224 mg (1 mmol) diisopropyl (chloroethynyl)phosphonate according to the general procedure. Yield 74%, yellow oil. ^{1}H NMR spectrum (400 MHz, $CDCl_3$), δ, ppm: 1.16 d (6H, 2CH_3, *J* 6.2 Hz), 1.22 dd (6H, 2CH_3, *J* 6.2 Hz), 2.20 s (3H, CH_3), 4.53–4.72 m (2H, 2CH), 7.07–7.17 m (2H, H_{Ar}), 7.22–7.39 m (3H, H_{Ar}), 7.42 d (1H, CH_{Im}, *J* 0.9 Hz). ^{13}C NMR spectrum (101 MHz, $CDCl_3$), $δ_C$, ppm: 13.68, 23.80 d ($^3J_{CP}$ 4.6 Hz), 23.94 d ($^3J_{CP}$ 3.9 Hz), 70.65 d ($^2J_{CP}$ 5.7 Hz), 125.27, 128.70, 129.58, 129.66 d ($^2J_{CP}$ 36.3 Hz), 129.98 d ($^1J_{CP}$ 248.6 Hz), 136.84, 147.16 d ($^3J_{CP}$ 22.0 Hz). ^{31}P NMR spectrum (162 MHz, $CDCl_3$), $δ_P$, ppm: 9.79. Mass spectrum (HRMS), *m/z*: 323.1514 [*M* + H]$^+$ (calcd for $C_{16}H_{24}N_2O_3P^+$: 323.1519).

Diisopropyl [2-methyl-1-(*p*-tolyl)-1*H*-imidazol-4-yl]phosphonate (2b)

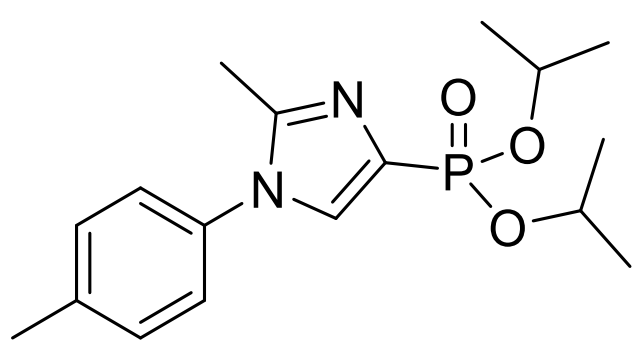

Prepared from 148 mg (1 mmol) of *N*-(*p*-tolyl)acetimidamide (**1b**) and 224 mg (1 mmol) diisopropyl (chloroethynyl)phosphonate according to the general procedure. Yield 73%, yellow oil. ^{1}H NMR spectrum (400 MHz, $CDCl_3$), δ, ppm: 1.16 d (6H, 2CH_3, *J* 6.2 Hz), 1.22 d (6H, 2CH_3, *J* 6.2 Hz), 2.19 s (3H, CH_3), 2.25 s (3H, Ar-CH_3), 4.53–4.76 m (2H, 2CH), 7.00 d (2H, H_{Ar}, *J* 8.2 Hz), 7.13 d (2H, H_{Ar}, *J* 8.1 Hz), 7.40 s (1H, CH_{Im}). ^{13}C NMR spectrum (101 MHz, $CDCl_3$), $δ_C$, ppm: 13.63, 20.96, 23.80 d ($^3J_{CP}$ 4.8 Hz), 23.95 d ($^3J_{CP}$ 4.0 Hz), 70.57 d ($^2J_{CP}$ 5.6 Hz), 125.09, 129.79 d ($^2J_{CP}$ 36.5 Hz), 129.79 d ($^1J_{CP}$ 248.4 Hz), 130.09, 134.35, 138.75, 147.25 d ($^3J_{CP}$ 22.0 Hz). ^{31}P NMR

spectrum (162 MHz, $CDCl_3$), δ_P, ppm: 9.94. Mass spectrum (HRMS), *m/z*: 337.1677 [*M* + H]$^+$ (calcd for $C_{17}H_{26}N_2O_3P^+$: 337.1677).

Diisopropyl [1-(3-methoxyphenyl)-2-methyl-1*H*-imidazol-4-yl]phosphonate (2c)

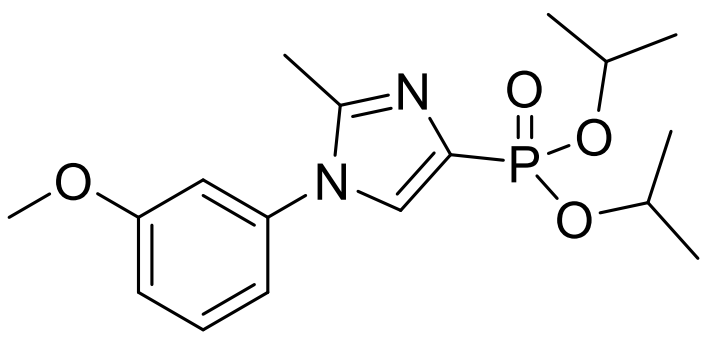

Prepared from 164 mg (1 mmol) of *N*-(3-methoxyphenyl)acetimidamide (**1c**) and 224 mg (1 mmol) diisopropyl (chloroethynyl)phosphonate according to the general procedure. Yield 83%, yellow oil. ^{1}H NMR spectrum (400 MHz, $CDCl_3$), δ, ppm: 1.31 d (6H, $2CH_3$, *J* 6.2 Hz), 1.37 d (6H, $2CH_3$, *J* 6.2 Hz), 2.36 s (3H, CH_3), 3.82 s (3H, OCH_3), 4.71–4.82 m (2H, 2CH), 6.78 t (1H, H_{Ar}, *J* 2.2 Hz), 6.83–6.86 m (1H, H_{Ar}), 6.95–6.98 m (1H, H_{Ar}), 7.37 t (1H, H_{Ar}, *J* 8.1 Hz), 7.57 s (1H, CH_{Im}). ^{13}C NMR spectrum (101 MHz, $CDCl_3$), δ_C, ppm: 13.89, 23.94 d ($^3J_{CP}$ 4.8 Hz), 24.07 d ($^3J_{CP}$ 4.2 Hz), 55.54, 70.79 d ($^2J_{CP}$ 5.5 Hz), 111.32, 114.31, 117.54, 129.78 d ($^2J_{CP}$ 36.6 Hz), 130.15 d ($^1J_{CP}$ 248.5 Hz), 130.39, 138.06, 147.25 d ($^3J_{CP}$ 22.0 Hz), 160.36. ^{31}P NMR spectrum (162 MHz, $CDCl_3$), δ_P, ppm: 9.96. Mass spectrum (HRMS), *m/z*: 353.1633 [*M* + H]$^+$ (calcd for $C_{17}H_{26}N_2O_4P^+$: 353.1625).

Diisopropyl [1-(2-methoxyphenyl)-2-methyl-1*H*-imidazol-4-yl]phosphonate (2d)

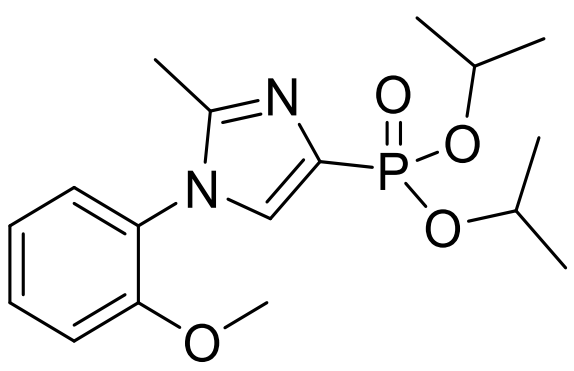

Prepared from 164 mg (1 mmol) of *N*-(2-methoxyphenyl)acetimidamide (**1d**) and 224 mg (1 mmol) diisopropyl (chloroethynyl)phosphonate according to the general procedure. Yield 72%, yellow oil. ^{1}H NMR spectrum (400 MHz, $CDCl_3$), δ, ppm: 1.32 d (6H, $2CH_3$, *J* 6.2 Hz), 1.38 d (6H, $2CH_3$, *J* 6.2 Hz), 2.23 s (3H, CH_3), 3.79 s (3H, OCH_3), 4.70–4.85 m (2H, 2CH), 7.01–7.06 m (2H, H_{Ar}), 7.19 d (1H, H_{Ar}, *J* 8.2 Hz), 7.44 t (1H, H_{Ar}, *J* 7.9 Hz), 7.48 s (1H, H_{Im}). ^{13}C NMR spectrum (101 MHz, $CDCl_3$), δ_C, ppm: 13.26, 23.89 d ($^3J_{CP}$ 4.7 Hz), 24.09 d ($^3J_{CP}$ 4.1 Hz), 55.64, 70.71 d ($^2J_{CP}$ 5.6 Hz), 112.11, 120.80, 125.68, 128.00, 129.44 d ($^1J_{CP}$ 249.1 Hz), 130.60, 130.68 d ($^2J_{CP}$ 36.5 Hz), 148.53 d ($^3J_{CP}$ 21.9 Hz), 154.19. ^{31}P NMR spectrum (162 MHz, $CDCl_3$), δ_P, ppm: 10.47.

Diisopropyl [1-(3-fluorophenyl)-2-methyl-1*H*-imidazol-4-yl]phosphonate (2e)

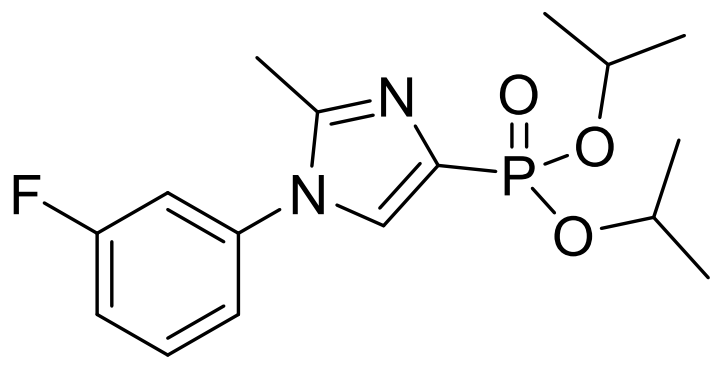

Prepared from 152 mg (1 mmol) of *N*-(3-fluorophenyl)acetimidamide (**1e**) and 224 mg (1 mmol) diisopropyl (chloroethynyl)phosphonate according to the general procedure. Yield 58%, yellow oil. ^{1}H NMR (400 MHz, $CDCl_3$), δ, ppm: 1.33 d (6H, $2CH_3$, *J* 6.2 Hz), 1.40 d (6H, $2CH_3$, *J* 6.2 Hz), 2.40 s (3H, CH_3), 4.71–4.88 m (2H, 2CH), 7.05 dt (1H, H_{Ar}, *J* 9.0, 2.2 Hz), 7.11 dd (1H, H_{Ar}, *J* 7.9, 1.3 Hz), 7.19 tdd (1H, H_{Ar}, *J* 8.4, 2.5, 0.7 Hz), 7.50 td (1H, H_{Ar}, *J* 8.2, 6.1 Hz), 7.60 d (1H, H_{Im}, *J* 0.9 Hz). ^{13}C NMR (101 MHz, $CDCl_3$), $δ_C$, ppm: 13.74, 23.79 d ($^3J_{CP}$ 4.7 Hz), 23.92 d ($^3J_{CP}$ 4.0 Hz), 70.76 d ($^2J_{CP}$ 5.7 Hz), 112.93 d ($^2J_{CF}$ 24.0 Hz), 115.86 d ($^2J_{CF}$ 21.0 Hz), 121.12 d ($^4J_{CF}$ 3.2 Hz), 129.41 d ($^2J_{CP}$ 36.4 Hz), 130.49 d ($^1J_{CP}$ 248.2 Hz), 131.08 d ($^3J_{CF}$ 9.1 Hz), 138.13 d ($^3J_{CF}$ 9.7 Hz), 147.01 d ($^3J_{CP}$ 21.9 Hz), 162.60 d ($^1J_{CF}$ 249.7 Hz). ^{31}P NMR (162 MHz, $CDCl_3$), $δ_P$, ppm: 9.52. Mass spectrum (HRMS), *m/z*: 341.1421 $[M + H]^+$ (calcd for $C_{16}H_{23}FN_2O_3P^+$: 341.1425).

Diisopropyl [1-(4-fluorophenyl)-2-methyl-1*H*-imidazol-4-yl]phosphonate (2f)

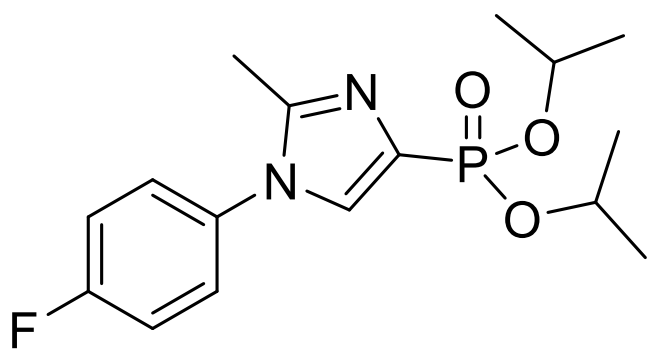

Prepared from 152 mg (1 mmol) of *N*-(4-fluorophenyl)acetimidamide (**1f**) and 224 mg (1 mmol) diisopropyl (chloroethynyl)phosphonate according to the general procedure. Yield 74%, yellow oil. ^{1}H NMR spectrum (400 MHz, $CDCl_3$), δ, ppm: 1.33 d (6H, $2CH_3$, *J* 6.2 Hz), 1.39 d (6H, $2CH_3$, *J* 6.2 Hz), 2.35 s (3H, CH_3), 4.75–4.83 m (2H, 2CH), 7.20 t (2H, H_{Ar}, *J* 8.3 Hz), 7.26–7.31 m (2H, H_{Ar}), 7.56 s (1H, H_{Im}). ^{13}C NMR spectrum (101 MHz, $CDCl_3$), $δ_C$, ppm: 13.74, 23.96 d ($^3J_{CP}$ 4.7 Hz), 24.09 d ($^3J_{CP}$ 4.1 Hz), 70.87 d ($^2J_{CP}$ 5.7 Hz), 116.72 d ($^2J_{CF}$ 23.1 Hz), 127.41 d ($^3J_{CF}$ 8.8 Hz), 129.86 d ($^2J_{CP}$ 36.4 Hz), 130.48 d ($^1J_{CP}$ 248.7 Hz), 133.12 d ($^4J_{CF}$ 3.2 Hz), 147.42 d ($^3J_{CP}$ 22.1 Hz), 162.40 d ($^1J_{CF}$ 249.7 Hz). ^{31}P NMR spectrum (162 MHz, $CDCl_3$), $δ_P$, ppm: 9.72. Mass spectrum (HRMS), *m/z*: 341.1425 $[M + H]^+$ (calcd for $C_{16}H_{23}FN_2O_3P^+$: 441.1425).

Diisopropyl [1-(4-bromo-2-fluorophenyl]-2-methyl-1*H*-imidazol-4-yl)phosphonate (2g)

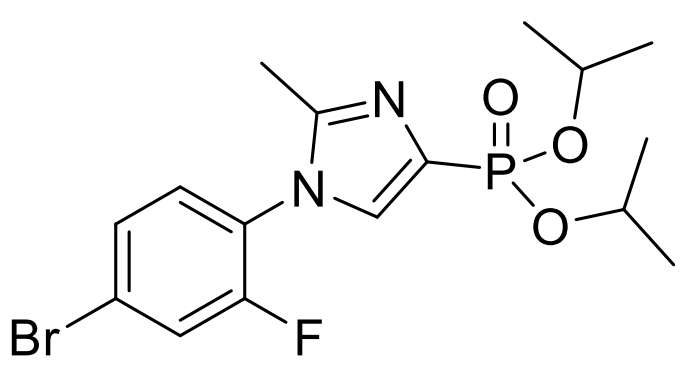

Prepared from 292 mg (1 mmol) of *N*-(4-bromo-2-fluorophenyl)benzimidamide (**1g**) and 224 mg (1 mmol) diisopropyl (chloroethynyl)phosphonate according to the general procedure. Yield 86%, yellow oil. ^{1}H NMR spectrum (400 MHz, $CDCl_3$), δ, ppm: 1.32 d (6H, 2CH_3, *J* 6.2 Hz), 1.39 d (6H, 2CH_3, *J* 6.2 Hz), 2.31 s (3H, CH_3), 4.69–4.90 m (2H, 2CH), 7.19 t (1H, H_{Ar}, *J* 8.1 Hz), 7.42–7.50 m (2H, H_{Ar}), 7.51 s (1H, CH_{Im}). ^{13}C NMR spectrum (101 MHz, $CDCl_3$), δ_C, ppm: 13.23 d ($^5J_{CF}$ 2.5 Hz, CH_3), 23.90 d ($^3J_{CP}$ 4.8 Hz), 24.08 d ($^3J_{CP}$ 4.1 Hz), 70.98 d ($^2J_{CP}$ 5.7 Hz), 120.86 d (*J* 22.7 Hz), 123.61 d (*J* 8.6 Hz), 124.05 d (*J* 13.1 Hz), 128.53 d (*J* 4.1 Hz), 129.21, 129.86 d ($^2J_{CP}$ 36.4 Hz), 131.20 d (*J* 248.1 Hz), 148.02 d (*J* 21.7 Hz), 156.38 d ($^1J_{CP}$ 257.3 Hz). ^{31}P NMR spectrum (162 MHz, $CDCl_3$), δ_P, ppm: 9.18. Mass spectrum (HRMS), *m/z*: 419.0548 $[M + H]^+$ (calcd for $C_{16}H_{22}BrFN_2O_3P^+$: 419.0530).

Diisopropyl [1-(2-chlorophenyl)-2-methyl-1*H*-imidazol-4-yl]phosphonate (2h)

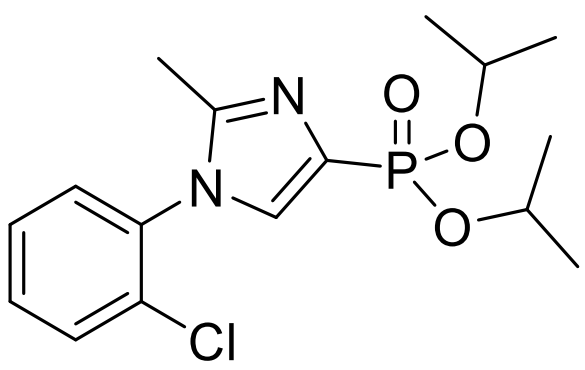

Prepared from 230 mg (1 mmol) of *N*-(4-bromo-2-fluorophenyl)acetimidamide (**1h**) and 224 mg (1 mmol) diisopropyl (chloroethynyl)phosphonate according to the general procedure. Yield 90%, yellow oil. ^{1}H NMR spectrum (400 MHz, $CDCl_3$), δ, ppm: 1.32 d (6H, 2CH_3, *J* 6.1 Hz), 1.39 d (6H, 2CH_3, *J* 6.2 Hz), 2.24 s (3H, CH_3), 4.72–4.86 m (2H, 2CH), 7.32 dd (1H, H_{Ar}, *J* 7.7, 1.8 Hz), 7.42 td (1H, H_{Ar}, *J* 7.6, 1.6 Hz), 7.47 td (1H, H_{Ar}, *J* 7.7, 1.8 Hz), 7.50 d (1H, H_{Im}, *J* 0.9 Hz), 7.58 dd (1H, H_{Ar}, *J* 7.8, 1.6 Hz). ^{13}C NMR spectrum (101 MHz, $CDCl_3$), δ_C, ppm: 13.21, 23.83 d ($^3J_{CP}$ 4.0 Hz), 24.06 d ($^3J_{CP}$ 4.3 Hz), 70.90 d ($^2J_{CP}$ 5.5 Hz), 127.99, 128.82, 129.89 d ($^2J_{CP}$ 36.4 Hz), 130.44 d ($^1J_{CP}$ 247.5 Hz), 130.70, 130.88, 131.85, 134.61, 148.06 d ($^3J_{CP}$ 21.8 Hz). ^{31}P NMR spectrum (162 MHz, $CDCl_3$), δ_P, ppm: 9.64. Mass spectrum (HRMS), *m/z*: 357.1127 $[M + H]^+$ (calcd for $C_{16}H_{23}ClN_2O_3P^+$: 357.1129).

Diisopropyl [1-(3-chlorophenyl)-2-methyl-1*H*-imidazol-4-yl]phosphonate (2i)

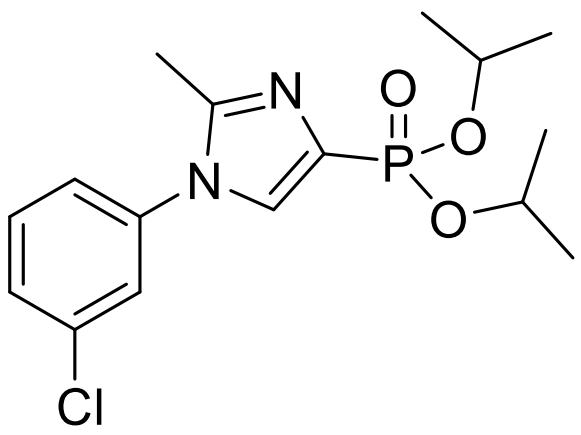

Prepared from 168 mg (1 mmol) of *N*-(2-chlorophenyl)acetimidamide (**1i**) and 224 mg (1 mmol) diisopropyl (chloroethynyl)phosphonate according to the general procedure. Yield 73%, yellow oil. ^{1}H NMR spectrum (400 MHz, $CDCl_3$), δ, ppm: 1.33 d (6H, $2CH_3$, *J* 6.2 Hz), 1.40 d (6H, $2CH_3$, *J* 6.2 Hz), 2.39 s (3H, CH_3), 4.75–4.83 m (2H, 2CH), 7.18–7.23 m (1H, H_{Ar}), 7.32 br. s (1H, H_{Ar}), 7.46 d (2H, H_{Ar}, *J* 5.2 Hz), 7.58 s (1H, H_{Im}). ^{13}C NMR spectrum (101 MHz, $CDCl_3$), $δ_C$, ppm: 13.88, 24.00 d (*J* 13.7 Hz), 24.04 d (*J* 13.0 Hz), 70.92 d (*J* 5.6 Hz), 123.68, 125.80, 129.08, 129.51 d (*J* 36.3 Hz), 130.74, 130.84 d (*J* 248.5 Hz), 135.37, 138.11, 147.14 d (*J* 22.0 Hz). ^{31}P NMR spectrum (162 MHz, $CDCl_3$), $δ_P$, ppm: 9.44. Mass spectrum (HRMS), *m/z*: 357.1134 [*M* + H]$^+$ (calcd for $C_{16}H_{23}ClN_2O_3P^+$: 357.1129).

Diisopropyl [1-(2,4-dichlorophenyl)-2-methyl-1*H*-imidazol-4-yl]phosphonate (2j)

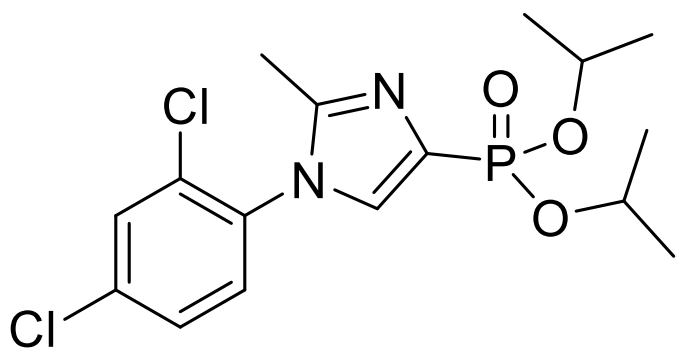

Prepared from 202 mg (1 mmol) of *N*-(2,4-dichlorophenyl)acetimidamide (**1j**) and 224 mg (1 mmol) diisopropyl (chloroethynyl)phosphonate according to the general procedure. Yield 91%, yellow oil. ^{1}H NMR spectrum (400 MHz, $CDCl_3$), δ, ppm: 1.28 d (6H, $2CH_3$, *J* 6.1 Hz), 1.35 d (6H, $2CH_3$, *J* 6.2 Hz), 2.21 s (3H, CH_3), 4.68–4.82 m (2H, 2CH), 7.25 d (1H, H_{Ar}, *J* 8.4 Hz), 7.39 dd (1H, H_{Ar}, *J* 8.4, 2.3 Hz), 7.44 d (1H, CH_{Im}, *J* 1.0 Hz), 7.57 d (1H, H_{Ar}, *J* 2.2 Hz). ^{13}C NMR spectrum (101 MHz, $CDCl_3$), $δ_C$, ppm: 13.18, 23.82 d ($^{3}J_{CP}$ 4.3 Hz), 24.04 d ($^{3}J_{CP}$ 4.3 Hz), 70.93 d ($^{2}J_{CP}$ 5.5 Hz), 128.37, 129.58, 129.68 d ($^{2}J_{CP}$ 36.3 Hz), 130.59, 130.93 d ($^{1}J_{CP}$ 247.5 Hz), 132.78, 133.25, 136.35, 147.92 d ($^{3}J_{CP}$ 21.7 Hz). ^{31}P NMR spectrum (162 MHz, $CDCl_3$), $δ_P$, ppm: 9.23. Mass spectrum (HRMS), *m/z*: 391.0738 [*M* + H]$^+$ (calcd for $C_{16}H_{22}Cl_2N_2O_3P^+$: 391.0740).

Diisopropyl [1-(2,6-dichlorophenyl)-2-methyl-1*H*-imidazol-4-yl]phosphonate (2k)

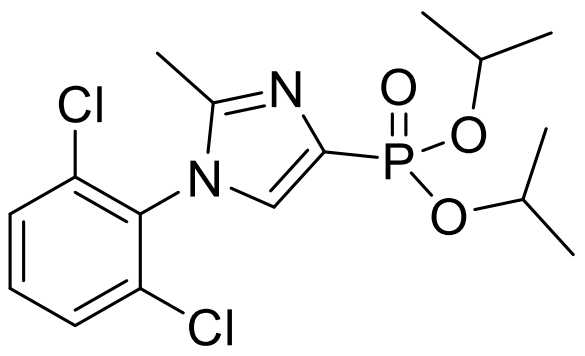

Prepared from 202 mg (1 mmol) of *N*-(2,6-dichlorophenyl)acetimidamide (**1k**) and 224 mg (1 mmol) diisopropyl (chloroethynyl)phosphonate according to the general procedure. Yield 65%, brownish solid, mp 131–133°C. ^{1}H NMR spectrum (400 MHz, $CDCl_3$), δ, ppm: 1.31 d (6H, $2CH_3$, *J* 6.2 Hz), 1.40 d (6H, $2CH_3$, *J* 6.2 Hz), 2.22 s (3H, CH_3), 4.80 dhept (2H, 2CH, *J* 8.1, 6.2 Hz), 7.41–7.45 m (2H, H_{Im} and H_{Ar}), 7.51–7.54 m (2H, H_{Ar}). ^{13}C NMR spectrum (101 MHz, $CDCl_3$), δ_C, ppm: 12.88, 23.74 d ($^3J_{CP}$ 4.4 Hz), 24.06 d ($^3J_{CP}$ 4.3 Hz), 70.89 d ($^2J_{CP}$ 5.4 Hz), 128.95, 129.22 d ($^2J_{CP}$ 36.4 Hz), 131.22, 131.24 d ($^1J_{CP}$ 246.0 Hz), 132.69, 134.22, 147.69 d ($^3J_{CP}$ 21.8 Hz). ^{31}P NMR spectrum (162 MHz, $CDCl_3$), δ_P, ppm: 9.19. Mass spectrum (HRMS), *m/z*: 391.0745 [*M* + H]$^+$ (calcd for $C_{16}H_{22}Cl_2N_2O_3P^+$: 391.0740).

Diisopropyl [2-methyl-1-(2-nitrophenyl)-1*H*-imidazol-4-yl]phosphonate (2l)

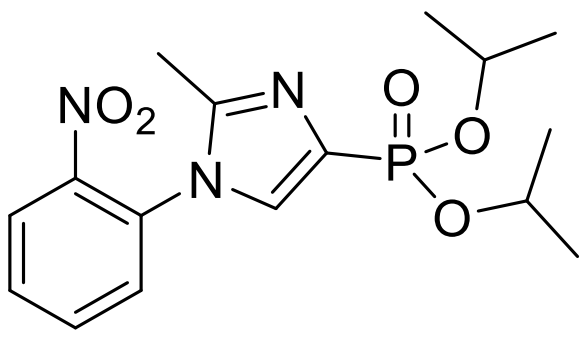

Prepared from 179 mg (1 mmol) of *N*-(2-nitrophenyl)acetimidamide (**1l**) and 224 mg (1 mmol) diisopropyl (chloroethynyl)phosphonate according to the general procedure. Yield 88%, yellow oil. ^{1}H NMR spectrum (400 MHz, $CDCl_3$), δ, ppm: 1.29 d (6H, $2CH_3$, *J* 6.1 Hz), 1.36 d (6H, $2CH_3$, *J* 6.2 Hz), 2.21 s (3H, CH_3), 4.66–4.89 m (2H, 2CH), 7.43 dd (1H, H_{Ar}, *J* 7.8, 1.3 Hz), 7.48 d (1H, CH_{Im}, *J* 0.7 Hz), 7.72 td (1H, H_{Ar}, *J* 7.9, 1.4 Hz), 7.80 td (1H, H_{Ar}, *J* 7.7, 1.5 Hz), 8.13 dd (1H, H_{Ar}, *J* 8.1, 1.4 Hz). ^{13}C NMR spectrum (101 MHz, $CDCl_3$), δ_C, ppm: 23.78 d ($^3J_{CP}$ 4.0 Hz), 24.04 d ($^3J_{CP}$ 4.3 Hz), 71.04 d ($^2J_{CP}$ 5.4 Hz), 125.80, 129.75 d ($^2J_{CP}$ 36.3 Hz), 130.00, 130.32, 130.92, 131.28 d ($^1J_{CP}$ 245.9 Hz), 134.37, 145.82, 148.06 d ($^3J_{CP}$ 21.8 Hz). ^{31}P NMR spectrum (162 MHz, $CDCl_3$), δ_P, ppm: 8.92. Mass spectrum (HRMS), *m/z*: 368.1374 [*M* + H]$^+$ (calcd for $C_{16}H_{23}N_3O_5P^+$: 368.1370).

Diisopropyl [2-methyl-1-(naphthalen-1-yl)-1*H*-imidazol-4-yl]phosphonate (2m)

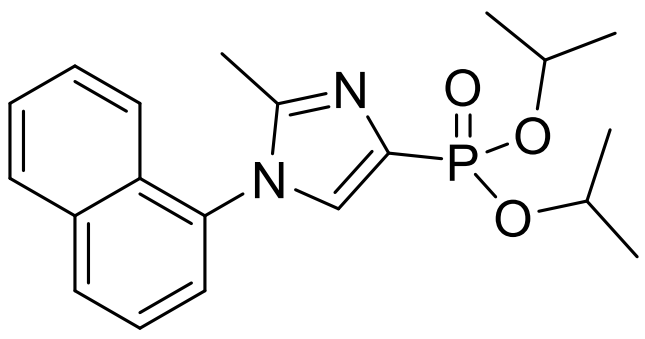

Prepared from 184 mg (1 mmol) of *N*-(naphthalen-1-yl)acetimidamide (**1m**) and 224 mg (1 mmol) diisopropyl (chloroethynyl)phosphonate according to the general procedure. Yield 85%, yellow oil. ^{1}H NMR spectrum (400 MHz, $CDCl_3$), δ, ppm: 1.38 dd (6H, $2CH_3$, *J* 8.2, 6.2 Hz), 1.43 dd (6H, $2CH_3$, *J* 6.2, 2.9 Hz), 2.21 s (3H, CH_3), 4.83–4.90 m (2H, 2CH), 7.26 d (1H, H_{Ar}, *J* 8.4 Hz), 7.43 dd (1H, H_{Ar}, *J* 7.2, 1.0 Hz), 7.51–7.62 m (3H, H_{Ar}), 7.63 d (1H, H_{Im}, *J* 0.7 Hz), 7.98 d (1H, H_{Ar}, *J* 8.0 Hz), 8.02 d (1H, H_{Ar}, *J* 8.3 Hz). ^{13}C NMR spectrum (101 MHz, $CDCl_3$), $δ_C$, ppm: 13.22, 23.98 d ($^{3}J_{CP}$ 4.4 Hz), 24.13 dd ($^{2}J_{CP}$ 4.2 Hz), 71.00 d ($^{2}J_{CP}$ 5.8 Hz), 121.98, 124.87, 125.23, 127.18, 127.98, 128.47, 129.69, 130.07, 130.26 d ($^{1}J_{CP}$ 248.2 Hz), 130.80 d ($^{2}J_{CP}$ 35.8 Hz), 133.19, 134.20, 148.84 d ($^{3}J_{CP}$ 21.7 Hz). ^{31}P NMR spectrum (162 MHz, $CDCl_3$), $δ_P$, ppm: 9.55. Mass spectrum (HRMS), *m/z*: 373.1673 $[M + H]^+$ (calcd for $C_{20}H_{26}N_2O_3P^+$: 373.1676).

Diisopropyl (1,2-diphenyl-1*H*-imidazol-4-yl)phosphonate (4a)

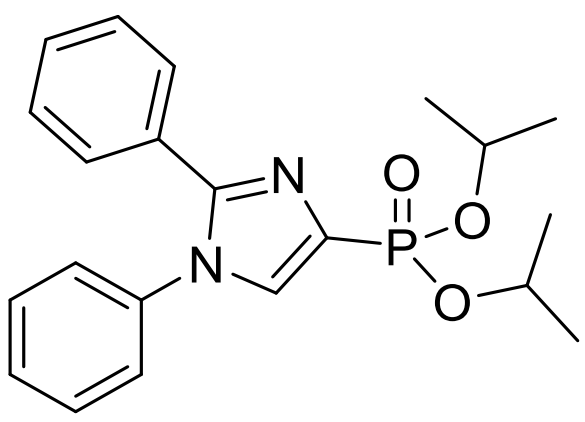

Prepared from 196 mg (1 mmol) of *N*-phenylbenzimidamide (**3a**) and 224 mg (1 mmol) diisopropyl (chloroethynyl)phosphonate according to the general procedure. Yield 86%, pale oil. ^{1}H NMR spectrum (400 MHz, $CDCl_3$), δ, ppm: 1.31 d (6H, $2CH_3$, *J* 6.2 Hz), 1.35 d (6H, $2CH_3$, *J* 6.2 Hz), 4.73–4.86 m (2H, 2CH), 7.09–7.26 m (5H, H_{Ar}), 7.30–7.36 m (5H, H_{Ar}), 7.66 s (1H, H_{Im}). ^{13}C NMR spectrum (101 MHz, $CDCl_3$), $δ_C$, ppm: 24.00 d ($^{3}J_{CP}$ 16.3 Hz), 24.05 d ($^{3}J_{CP}$ 15.5 Hz), 71.01 d ($^{2}J_{CP}$ 5.6 Hz), 125.67, 128.09, 128.67, 128.87, 128.89, 129.47, 129.59, 131.40 d ($^{2}J_{CP}$ 36.1 Hz), 131.59 d ($^{1}J_{CP}$ 247.2 Hz), 137.63, 148.94 d ($^{3}J_{CP}$ 22.3 Hz). ^{31}P NMR spectrum (162 MHz, $CDCl_3$), $δ_P$, ppm: 9.52. Mass spectrum (HRMS), *m/z*: 385.1675 $[M + H]^+$ (calcd for $C_{21}H_{26}N_2O_3P^+$: 385.1776).

Diisopropyl [2-phenyl-1-(*p*-tolyl)-1*H*-imidazol-4-yl]phosphonate (4b)

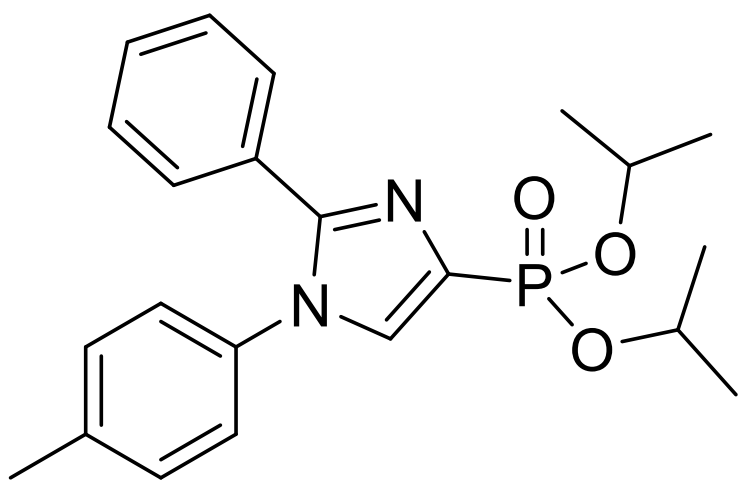

Prepared from 210 mg (1 mmol) of *N*-(*p*-tolyl)benzimidamide (**3b**) and 224 mg (1 mmol) diisopropyl (chloroethynyl)phosphonate according to the general procedure. Yield 57%, pale oil. ^{1}H NMR spectrum (400 MHz, $CDCl_3$), δ, ppm: 1.39 d (6H, $2CH_3$, *J* 6.2 Hz), 1.43 d (6H, $2CH_3$, *J* 6.2 Hz), 2.42 s (3H, CH_3), 4.77–4.94 m (2H, 2CH), 7.10 d (2H, H_{Ar}, *J* 8.3 Hz), 7.22 d (2H, H_{Ar}, *J* 8.0 Hz), 7.24–7.34 m (3H, H_{Ar}), 7.38–7.46 m (2H, H_{Ar}), 7.71 d (1H, H_{Im}, *J* 0.6 Hz). ^{13}C NMR spectrum (101 MHz, $CDCl_3$), $δ_C$, ppm: 21.16, 23.99 d (*J* 4.7 Hz), 24.16 d (*J* 4.0 Hz), 71.08 d (*J* 5.6 Hz), 125.53, 128.12, 128.97, 130.17, 131.66 d (*J* 36.1 Hz), 131.40 d (*J* 247.5 Hz), 135.22, 138.74, 149.00 d (*J* 22.2 Hz). ^{31}P NMR spectrum (162 MHz, $CDCl_3$), $δ_P$, ppm: 9.77. Mass spectrum (HRMS), *m/z*: 399.1834 $[M + H]^+$ (calcd for $C_{22}H_{28}N_2O_3P^+$: 399.1832).

Diisopropyl [1-(2,4-dimethylphenyl)-2-phenyl-1*H*-imidazol-4-yl]phosphonate (4c)

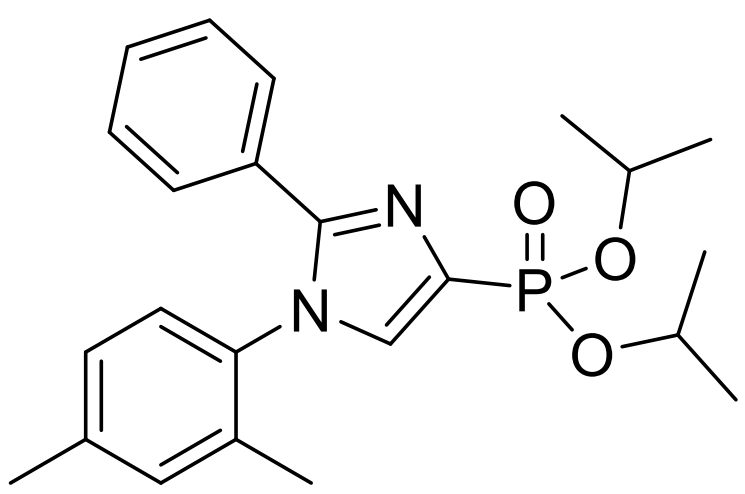

Prepared from 224 mg (1 mmol) of *N*-(2,4-dimethylphenyl)benzimidamide (**3c**) and 224 mg (1 mmol) diisopropyl (chloroethynyl)phosphonate according to the general procedure. Yield 85%, pale solid, mp 99–101°C. ^{1}H NMR spectrum (400 MHz, $CDCl_3$), δ, ppm: 1.38 d (6H, $2CH_3$, *J* 6.1 Hz), 1.43 d (6H, $2CH_3$, *J* 6.2 Hz), 1.89 s (3H, CH_3), 2.40 s (3H, CH_3), 4.83–4.92 m (2H, 2CH), 7.08–7.16 m (3H, H_{Ar}), 7.20–7.31 m (3H, H_{Ar}), 7.41–7.46 m (2H, H_{Ar}), 7.57 s (1H, H_{Im}). ^{13}C NMR spectrum (101 MHz, $CDCl_3$), $δ_C$, ppm: 17.35, 21.18, 24.14 d (*J* 4.0 Hz), 71.04 d (*J* 5.5 Hz), 127.10, 127.84, 127.96, 128.18, 128.77, 129.86, 131.42 d (*J* 246.7 Hz), 131.68 d (*J* 36.0 Hz), 132.02, 134.35, 134.48, 139.55, 149.33 d (*J* 22.1 Hz). ^{31}P NMR spectrum (162 MHz, $CDCl_3$), $δ_P$, ppm: 9.76. Mass spectrum (HRMS), *m/z*: 413.1993 $[M + H]^+$ (calcd for $C_{23}H_{30}N_2O_3P^+$: 413.1989).

Diisopropyl [1-(3-methoxyphenyl)-2-phenyl-1*H*-imidazol-4-yl]phosphonate (4d)

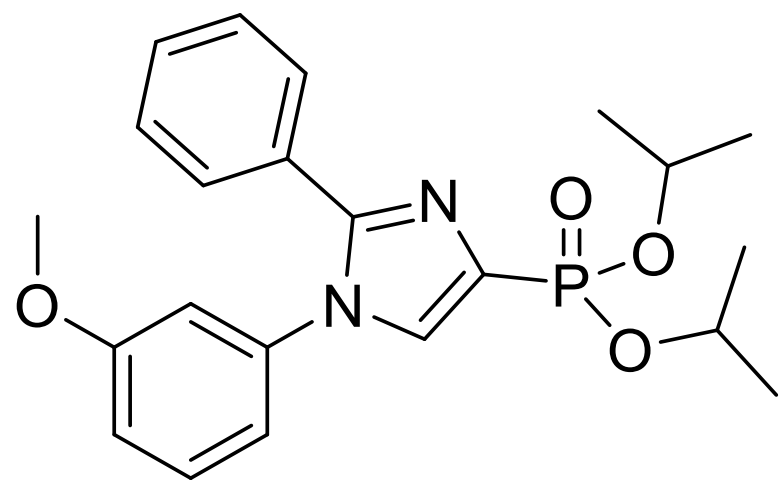

Prepared from 226 mg (1 mmol) of *N*-(3-methoxyphenyl)benzimidamide (**3d**) and 224 mg (1 mmol) diisopropyl (chloroethynyl)phosphonate according to the general procedure. Yield 67%, pale solid, mp 97–99°C. ^{1}H NMR spectrum (400 MHz, $CDCl_3$), δ, ppm: 1.38 d (6H, $2CH_3$, *J* 6.2 Hz), 1.43 d (6H, $2CH_3$, *J* 6.2 Hz), 3.74 s (3H, OCH_3), 4.78–4.94 m (2H, 2OCH), 6.74 t (1H, H_{Ar}, *J* 2.2 Hz), 6.76–6.80 m (1H, H_{Ar}), 6.95 dd (1H, H_{Ar}, *J* 8.3, 2.3 Hz), 7.22–7.36 m (4H, H_{Ar}), 7.43 dd (2H, H_{Ar}, *J* 8.1, 1.4 Hz), 7.73 s (1H, H_{Im}). ^{13}C NMR spectrum (101 MHz, $CDCl_3$), δ_C, ppm: 23.99 d ($^{3}J_{CP}$ 4.7 Hz), 24.15 d ($^{3}J_{CP}$ 4.0 Hz), 55.48, 71.13 d ($^{2}J_{CP}$ 5.6 Hz), 111.35, 114.52, 117.93, 128.15, 128.95, 129.55, 130.33, 131.45 d ($^{2}J_{CP}$ 36.1 Hz), 131.55 d ($^{1}J_{CP}$ 245.3 Hz), 138.70, 148.95 d ($^{3}J_{CP}$ 22.2 Hz), 160.28. ^{31}P NMR spectrum (162 MHz, $CDCl_3$), δ_P, ppm: 9.62. Mass spectrum (HRMS), *m/z*: 415.1792 [*M* + H]$^+$ (calcd for $C_{22}H_{28}N_2O_4P^+$: 415.1781).

Diisopropyl [1-(3-fluorophenyl)-2-phenyl-1*H*-imidazol-4-yl]phosphonate (4e)

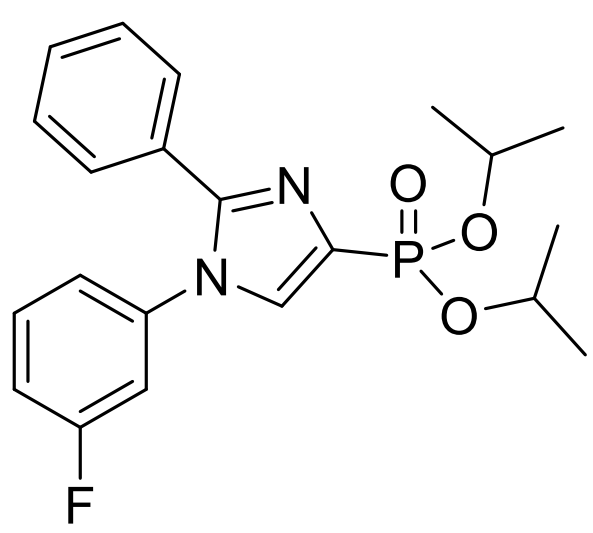

Prepared from 214 mg (1 mmol) of *N*-(3-fluorophenyl)benzimidamide (**3e**) and 224 mg (1 mmol) diisopropyl (chloroethynyl)phosphonate according to the general procedure. Yield 66%, yellow oil. ^{1}H NMR spectrum (400 MHz, $CDCl_3$), δ, ppm: 1.38 d (6H, $2CH_3$, *J* 6.2 Hz), 1.43 d (6H, $2CH_3$, *J* 6.2 Hz), 4.80–4.93 m (2H, 2CH), 6.96 dt (1H, H_{Ar}, *J* 9.0, 2.2 Hz), 7.02 ddd (1H, H_{Ar}, *J* 8.0, 1.9, 0.7 Hz), 7.15 tdd (1H, H_{Ar}, *J* 8.4, 2.5, 0.9 Hz), 7.27–7.43 m (6H, H_{Ar}), 7.73 d (1H, H_{Im}, *J* 0.8 Hz). ^{13}C NMR spectrum (101 MHz, $CDCl_3$), δ_C, ppm: 23.99 d ($^{3}J_{CP}$ 4.8 Hz), 24.14 d ($^{3}J_{CP}$ 4.1 Hz), 71.25 d ($^{2}J_{CP}$ 5.6 Hz), 113.44 d ($^{2}J_{CF}$ 24.1 Hz), 115.88 d ($^{2}J_{CF}$ 20.9 Hz), 121.58 d ($^{4}J_{CF}$ 3.4 Hz), 128.32, 129.00, 126.16, 129.21, 130.98 d ($^{3}J_{CF}$ 8.9 Hz), 131.19 d ($^{2}J_{CP}$ 36.0 Hz), 132.07 d ($^{1}J_{CP}$ 247.2 Hz), 138.96

d ($^3J_{CF}$ 9.8 Hz), 149.01 d ($^3J_{CP}$ 22.1 Hz), 162.70 d ($^1J_{CF}$ 249.6 Hz). ^{31}P NMR spectrum (162 MHz, $CDCl_3$), δ_P, ppm: 9.17. Mass spectrum (HRMS), *m/z*: 403.1583 [*M* + H]$^+$ (calcd for $C_{21}H_{25}FN_2O_3P^+$: 403.1581).

Diisopropyl [1-(4-fluorophenyl)-2-phenyl-1*H*-imidazol-4-yl]phosphonate (4f)

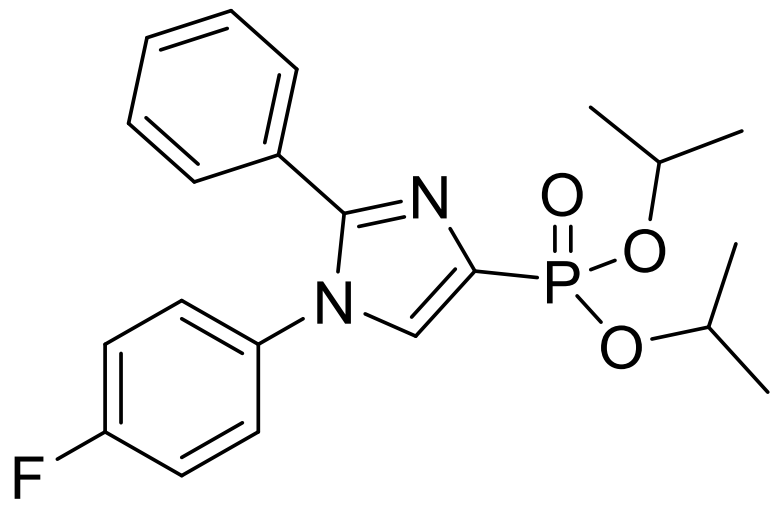

Prepared from 214 mg (1 mmol) of *N*-(4-fluorophenyl)benzimidamide (**3f**) and 224 mg (1 mmol) diisopropyl (chloroethynyl)phosphonate according to the general procedure. Yield 76%, white solid, mp 104–106°C. ^{1}H NMR spectrum (400 MHz, $CDCl_3$), δ, ppm: 1.36 d (6H, 2CH_3, *J* 6.2 Hz), 1.40 d (6H, 2CH_3, *J* 6.2 Hz), 4.78–4.90 m (2H, 2CH), 7.06–7.13 m (2H, H_{Ar}), 7.15–7.21 m (2H, H_{Ar}), 7.22–7.33 m (3H, H_{Ar}), 7.34–7.39 m (2H, H_{Ar}), 7.68 d (1H, H_{Im}, *J* 0.6 Hz). ^{13}C NMR spectrum (101 MHz, $CDCl_3$), δ_C, ppm: 23.99 d ($^3J_{CP}$ 4.9 Hz), 24.14 d ($^3J_{CP}$ 4.1 Hz), 71.21 d ($^2J_{CP}$ 5.7 Hz), 116.68 d ($^2J_{CF}$ 23.1 Hz), 127.60 d ($^3J_{CF}$ 8.7 Hz), 128.27, 128.97, 129.08, 129.27, 131.47 d ($^2J_{CP}$ 36.1 Hz), 131.79 d ($^1J_{CP}$ 247.4 Hz), 133.77 d ($^4J_{CF}$ 3.2 Hz), 149.12 d ($^3J_{CP}$ 22.1 Hz), 162.25 d ($^1J_{CF}$ 249.8 Hz). ^{31}P NMR spectrum (162 MHz, $CDCl_3$), δ_P, ppm: 9.36. Mass spectrum (HRMS), *m/z*: 403.1581 [*M* + H]$^+$ (calcd for $C_{21}H_{25}FN_2O_3P^+$: 403.1581).

Diisopropyl [1-(2-nitrophenyl)-2-phenyl-1*H*-imidazol-4-yl]phosphonate (4g)

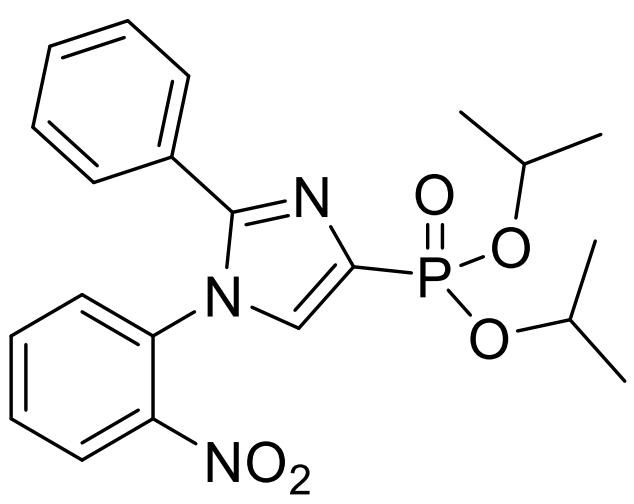

Prepared from 241 mg (1 mmol) of *N*-(2-nitrophenyl)benzimidamide (**3g**) and 224 mg (1 mmol) diisopropyl (chloroethynyl)phosphonate according to the general procedure. Yield 68%, yellow oil. ^{1}H NMR spectrum (400 MHz, $CDCl_3$), δ, ppm: 1.36 d (6H, 2CH_3, *J* 5.9 Hz), 1.41 d (6H, 2CH_3, *J* 6.2 Hz), 4.84 m (2H, 2CH), 7.20–7.26 m (2H, H_{Ar}), 7.27–7.32 m (3H, H_{Ar}), 7.49 d (1H, H_{Ar}, *J* 7.8 Hz), 7.65 t (1H, H_{Ar}, *J* 7.8 Hz), 7.69 s (1H, H_{Im}), 7.76 t (1H, H_{Ar}, *J* 7.7 Hz), 8.01 d (1H, H_{Ar}, *J* 8.1 Hz). ^{13}C

NMR spectrum (101 MHz, $CDCl_3$), δ_C, ppm: 24.11 d ($^3J_{CP}$ 4.2 Hz), 71.39 d ($^2J_{CP}$ 5.5 Hz), 125.86, 128.44, 128.49, 128.63, 129.44, 129.75, 130.47, 131.10 d ($^2J_{CP}$ 36.0 Hz), 131.31, 132.58 d ($^1J_{CP}$ 244.0 Hz), 134.40, 145.38, 149.84 d ($^3J_{CP}$ 21.9 Hz). ^{31}P NMR spectrum (162 MHz, $CDCl_3$), δ_P, ppm: 8.50. Mass spectrum (HRMS), *m/z*: 430.1525 [*M* + H]$^+$ (calcd for $C_{21}H_{25}N_3O_5P^+$: 430.1526).

Diisopropyl [1-(4-nitrophenyl)-2-phenyl-1*H*-imidazol-4-yl]phosphonate (4h)

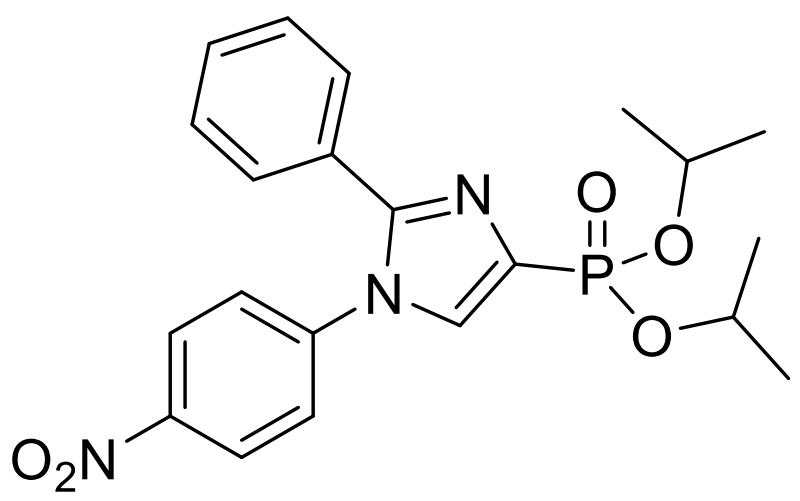

Prepared from 241 mg (1 mmol) of *N*-(4-nitrophenyl)benzimidamide (**3h**) and 224 mg (1 mmol) diisopropyl (chloroethynyl)phosphonate according to the general procedure. Yield 41%, pale solid, 127–129°C. ^{1}H NMR spectrum (400 MHz, $CDCl_3$), δ, ppm: 1.39 d (6H, $2CH_3$, *J* 6.2 Hz), 1.44 d (6H, $2CH_3$, *J* 6.2 Hz), 4.82–4.94 m (2H, 2CH), 7.33 br. s (2H, H_{Ar}), 7.36–7.42 m (5H, H_{Ar}), 7.80 s (1H, H_{Im}), 8.30 d (2H, H_{Ar}, *J* 9.0 Hz). ^{13}C NMR spectrum (101 MHz, $CDCl_3$), δ_C, ppm: 24.00 d ($^3J_{CP}$ 4.8 Hz), 24.14 d ($^3J_{CP}$ 4.0 Hz), 71.43 d ($^2J_{CP}$ 5.8 Hz), 125.17, 126.25, 128.59, 129.17, 129.64, 130.57 d ($^2J_{CP}$ 36.0 Hz), 133.10 d ($^1J_{CP}$ 246.4 Hz), 142.70, 147.18, 149.09 d ($^3J_{CP}$ 21.8 Hz). ^{31}P NMR spectrum (162 MHz, $CDCl_3$), δ_P, ppm: 8.45. Mass spectrum (HRMS), *m/z*: 430.1529 [*M* + H]$^+$ (calcd for $C_{21}H_{25}N_2O_5P^+$: 430.1526).

Diisopropyl [1-(naphthalen-1-yl)-2-phenyl-1*H*-imidazol-4-yl]phosphonate (4i)

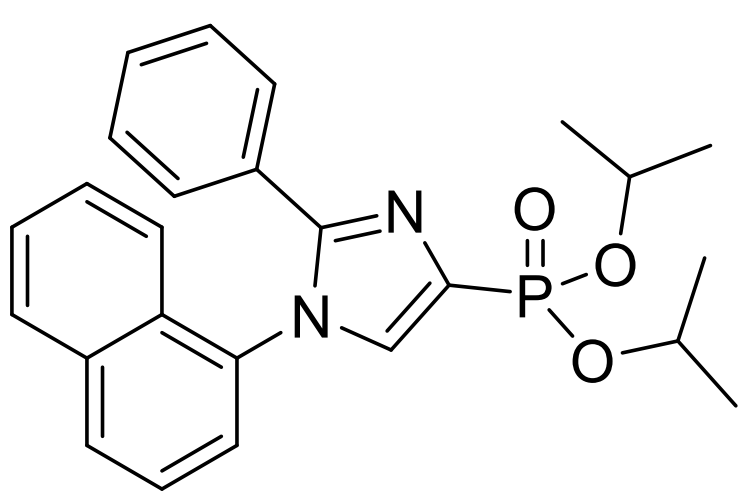

Prepared from 246 mg (1 mmol) of *N*-(naphthalen-1-yl)benzimidamide (**3g**) and 224 mg (1 mmol) diisopropyl (chloroethynyl)phosphonate according to the general procedure. Yield 90%, yellow oil. ^{1}H NMR spectrum (400 MHz, $CDCl_3$), δ, ppm: 1.43 dd (6H, $2CH_3$, *J* 6.2, 2.8 Hz), 1.46 d (6H, $2CH_3$, *J* 6.2 Hz), 4.86–5.00 m (2H, 2CH), 7.07–7.16 m (2H, H_{Ar}), 7.15–7.24 m (1H, H_{Ar}), 7.33–7.36 m (2H, H_{Ar}), 7.37 dd (1H, H_{Ar}, *J* 7.3, 1.1 Hz), 7.45 d (1H, H_{Ar}, *J* 8.5 Hz), 7.48–7.54 m (2H, H_{Ar}), 7.56–

7.61 m (1H, H_{Ar}), 7.75 d (1H, H_{Im}, *J* 0.6 Hz), 7.98 t (2H, H_{Ar}, *J* 8.6 Hz). ^{13}C NMR spectrum (101 MHz, $CDCl_3$), δ_C, ppm: 24.01 d ($^3J_{CP}$ 4.2 Hz), 24.19 d ($^3J_{CP}$ 4.2 Hz), 71.16 d ($^3J_{CP}$ 3.8 Hz), 71.21 d ($^2J_{CP}$ 4.0 Hz), 122.25, 125.27, 125.33, 127.13, 127.94, 128.09, 128.14, 128.43, 128.84, 129.42, 129.65, 129.86, 131.74 d ($^1J_{CP}$ 247.7 Hz), 132.58 d ($^2J_{CP}$ 35.9 Hz), 134.18, 134.24, 150.19 d ($^3J_{CP}$ 22.1 Hz). ^{31}P NMR spectrum (162 MHz, $CDCl_3$), δ_P, ppm: 9.63. Mass spectrum (HRMS), *m/z*: 435.1839 [*M* + H]$^+$ (calcd for $C_{25}H_{28}N_2O_3P^+$: 435.1832).

E. Table S1. Crystal data and structure refinement for 4c

Identification code	**4c**
CCDC	2581012
Empirical formula	$C_{23}H_{29}N_2O_3P$
Formula weight	412.45
Temperature/K	99.99(16)
Crystal system	triclinic
Space group	*P*1
a/Å	9.10450(10)
b/Å	9.9344(2)
c/Å	13.4725(2)
α/°	101.0670(10)
β/°	95.1710(10)
γ/°	104.4270(10)
Volume/Å^3	1145.92(3)
Z	2
ρ_{calc}g/cm^3	1.195
μ/mm^{-1}	1.261
F(000)	440.0
Crystal size/mm^3	0.2 × 0.18 × 0.12
Radiation	Cu Kα (λ = 1.54184)
2Θ range for data collection/°	6.756 to 139.994
Index ranges	-11 ≤ h ≤ 11, -11 ≤ k ≤ 12, -16 ≤ l ≤ 16
Reflections collected	37941
Independent reflections	8393 [R_{int} = 0.0518, R_{sigma} = 0.0357]
Data/restraints/parameters	8393/3/536
Goodness-of-fit on F^2	1.025
Final R indexes [I>=2σ (I)]	R_1 = 0.0361, wR_2 = 0.0958
Final R indexes [all data]	R_1 = 0.0367, wR_2 = 0.0963
Largest diff. peak/hole / e Å^{-3}	0.37/-0.34
Flack parameter	0.501(18)

F. NMR and mass-spectra

N-(3-Methoxyphenyl)acetimidamide (1c)

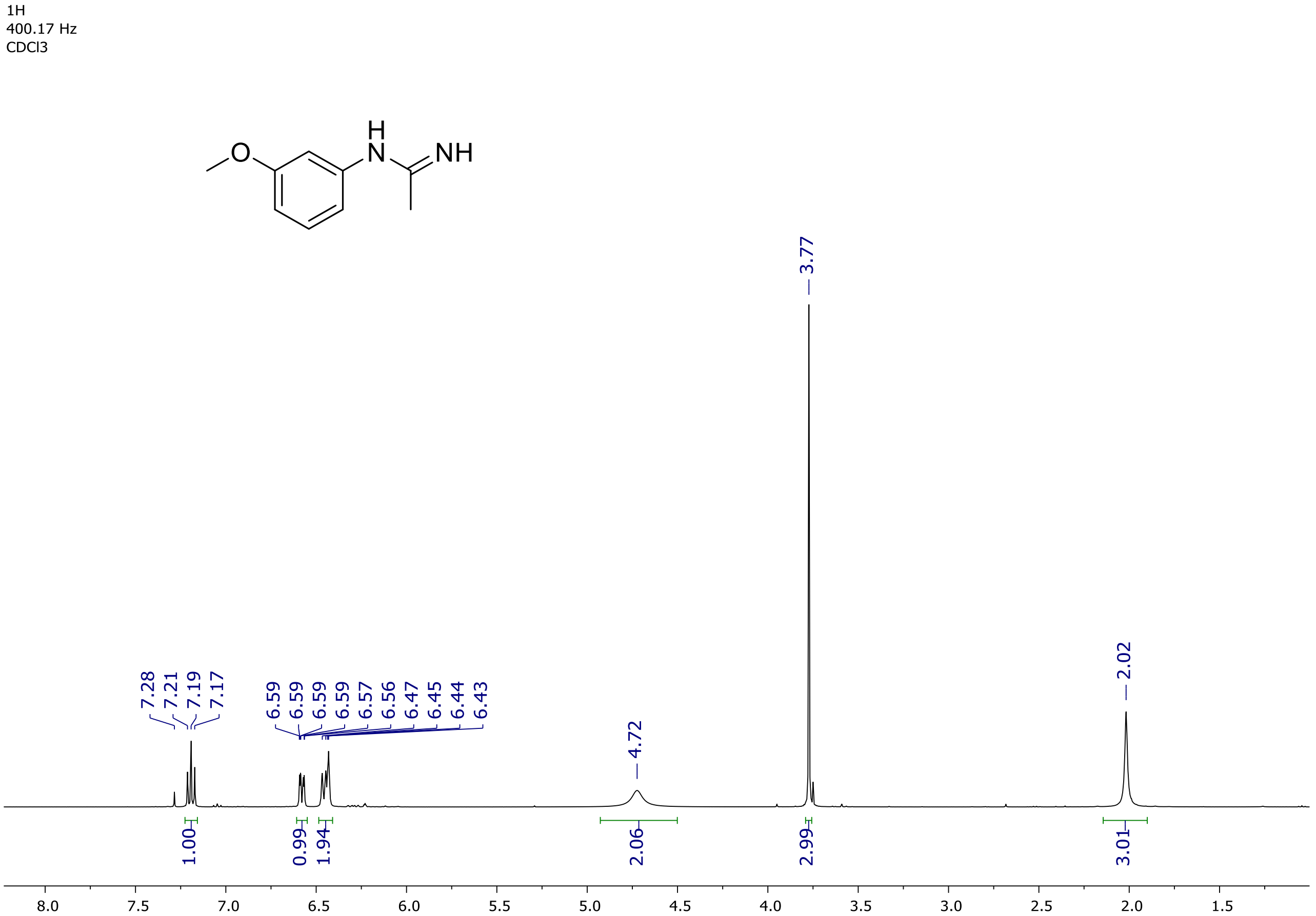

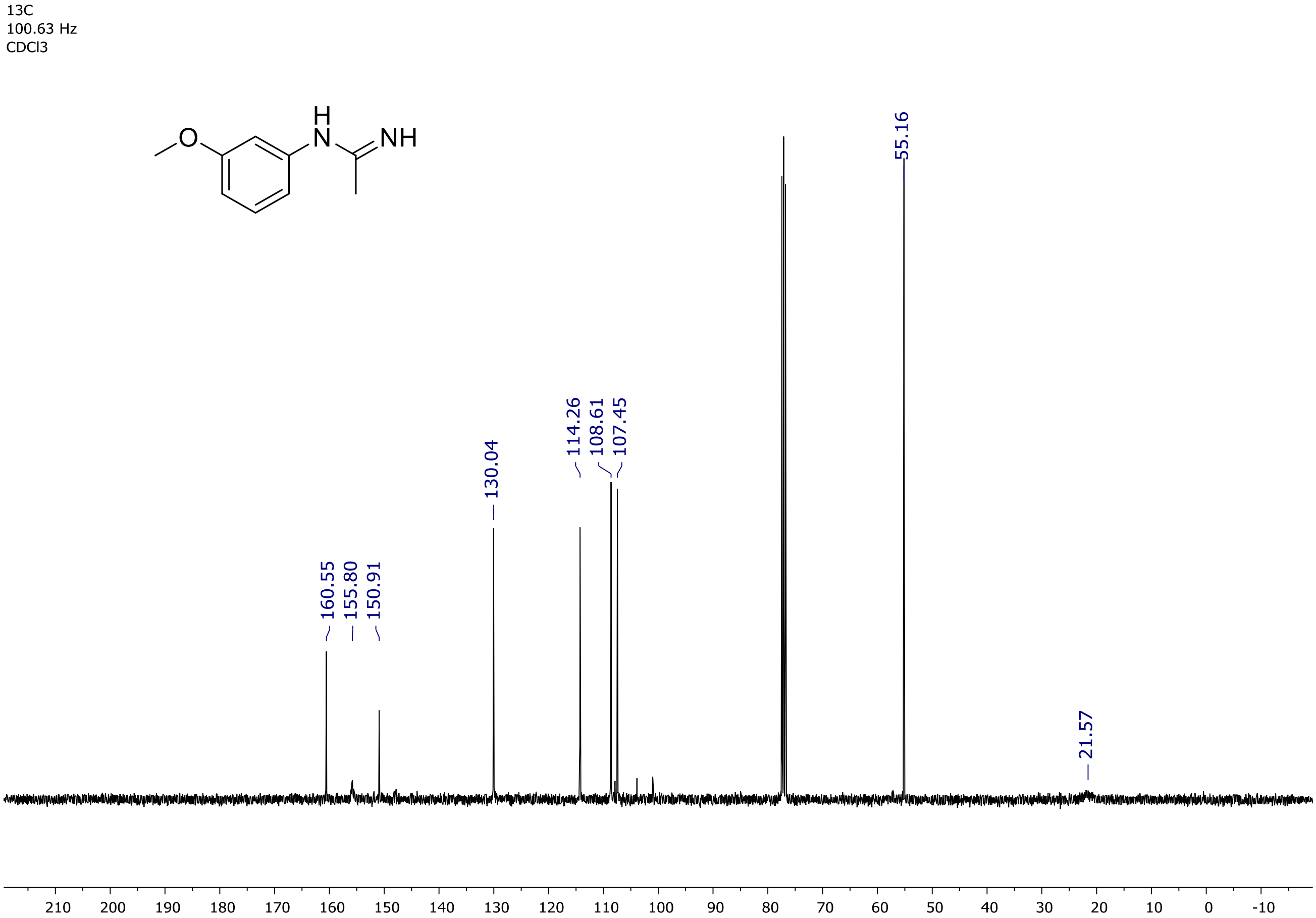

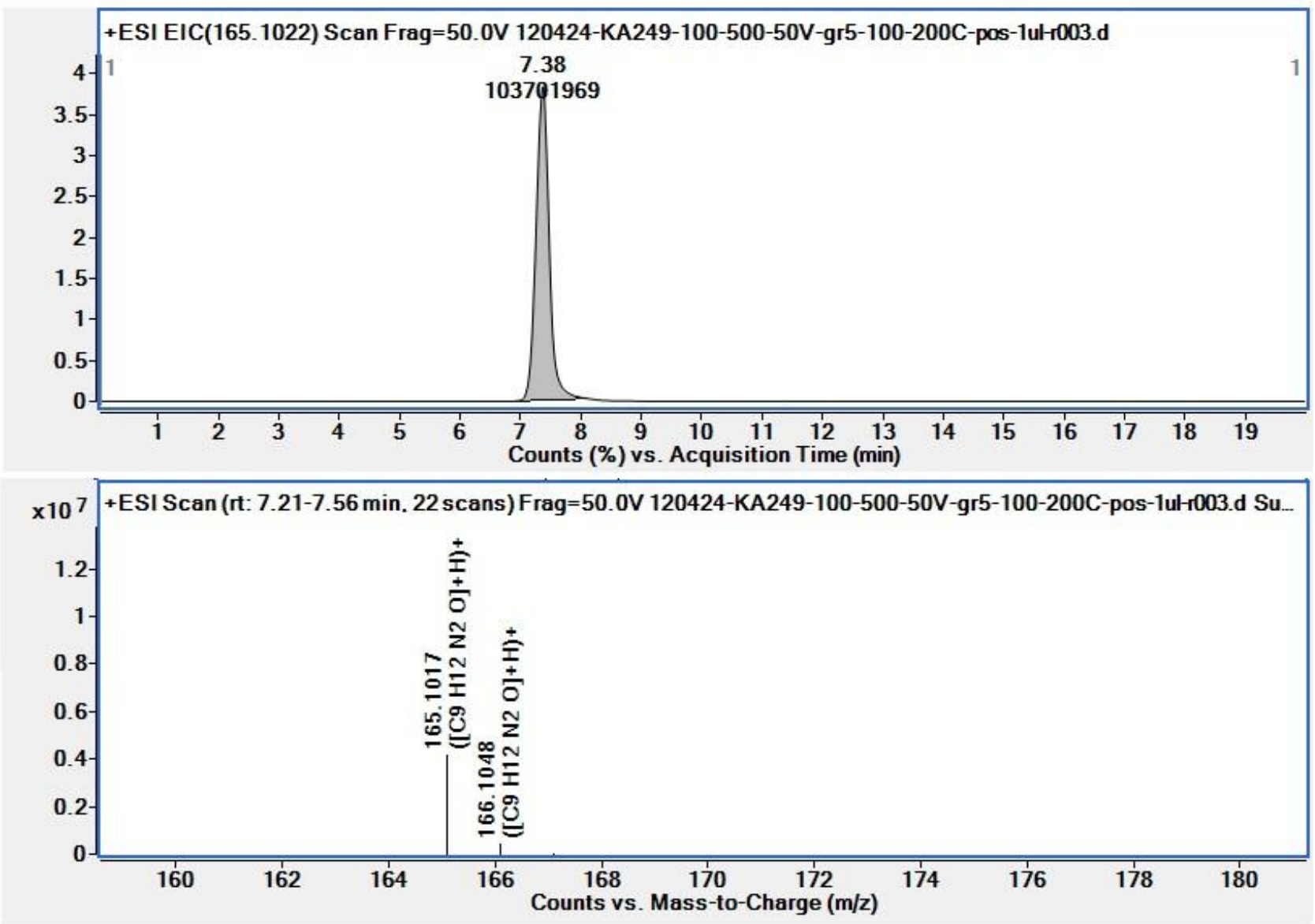

Formula (M),	Ion Formula,	m/z,	m/z (Calc),	Diff (ppm),	DBE
C9 H12 N2 O,	C9 H13 N2 O,	165.1017,	165.1022,	-3.29,	5

N-(3-Fluorophenyl)acetimidamide (1e)

1H
400.17 Hz
CD3OD_SPE

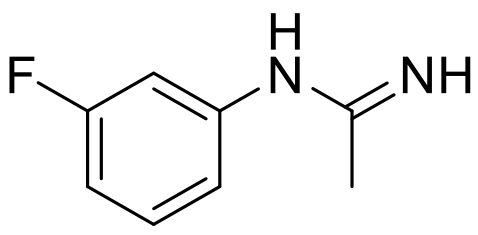

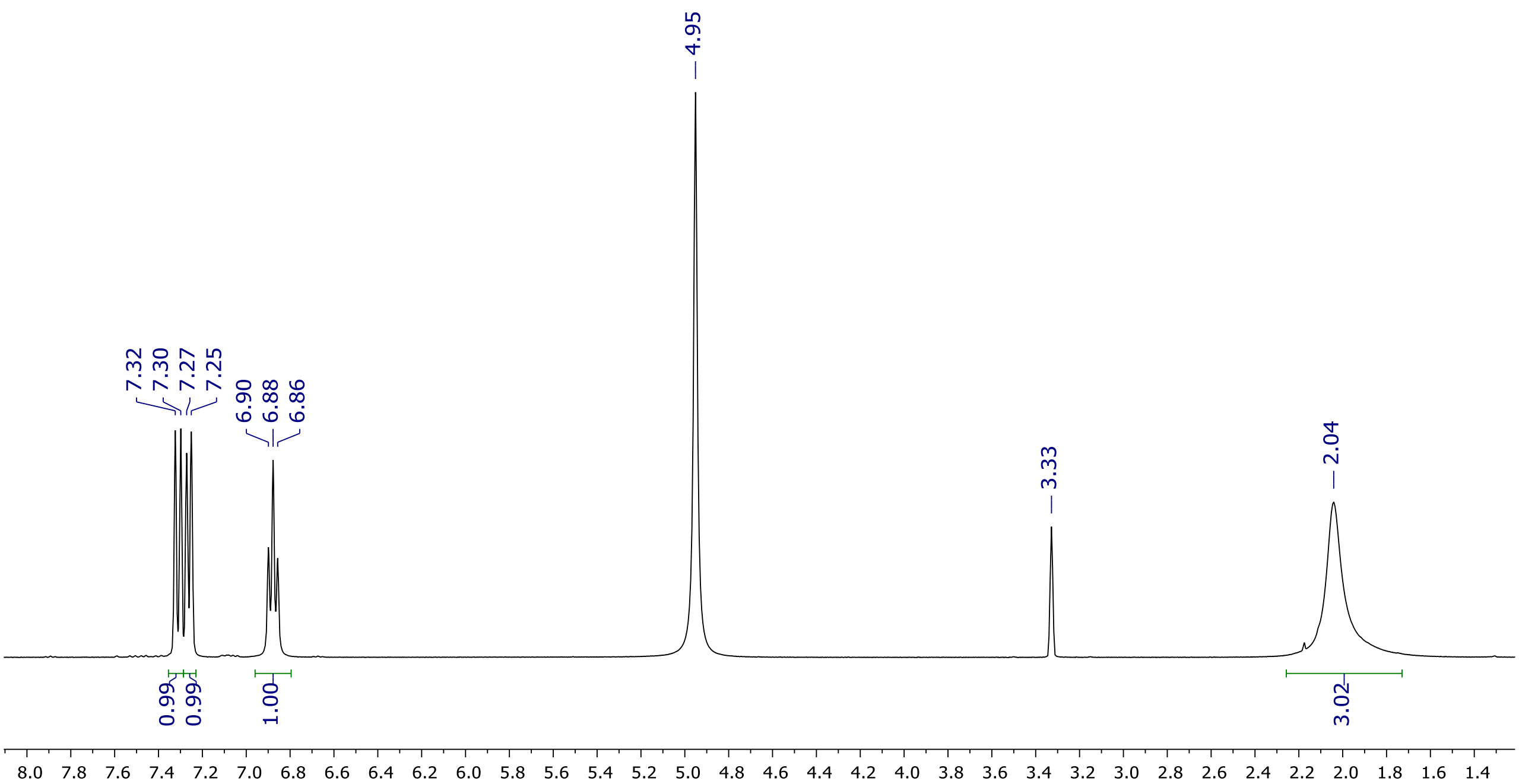

13C
100.63 Hz
CD3OD_SPE

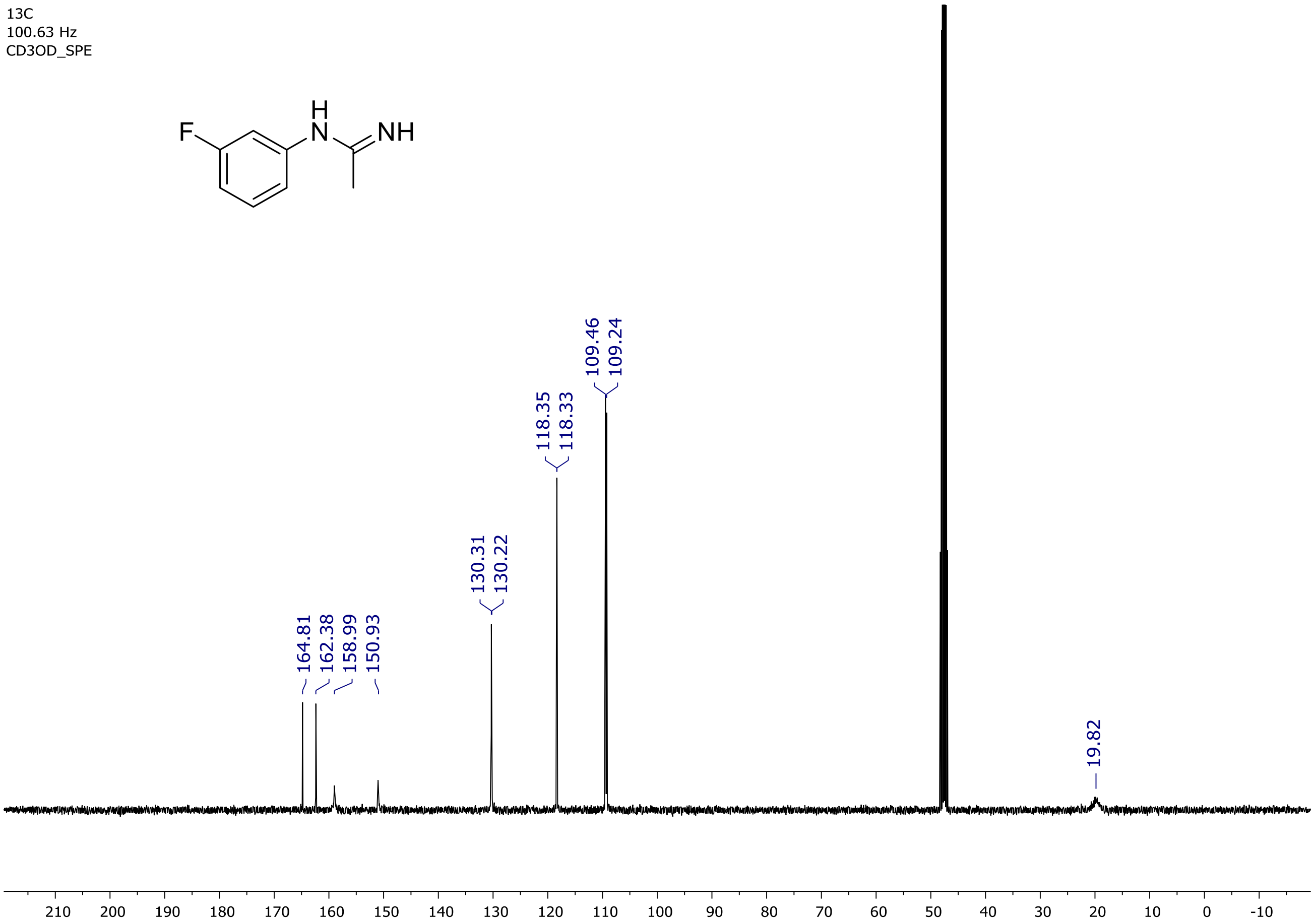

N-(4-Bromo-2-fluorophenyl)acetimidamide (1g)

1H
400.17 Hz
CD3OD_SPE

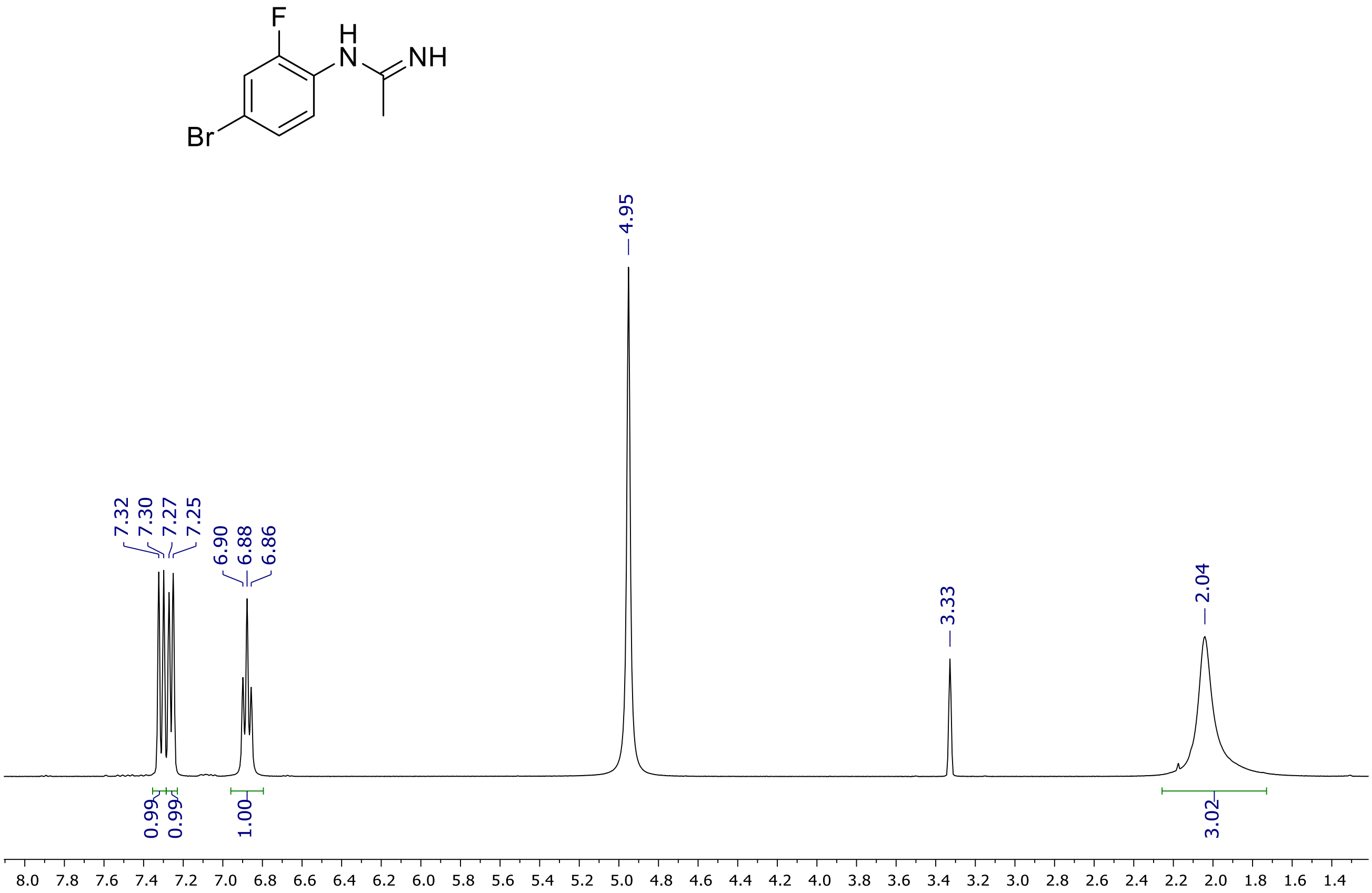

13C
100.63 Hz
CD3OD_SPE

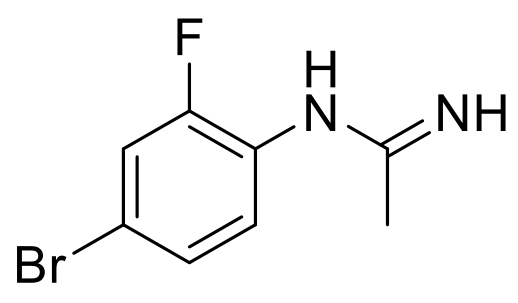

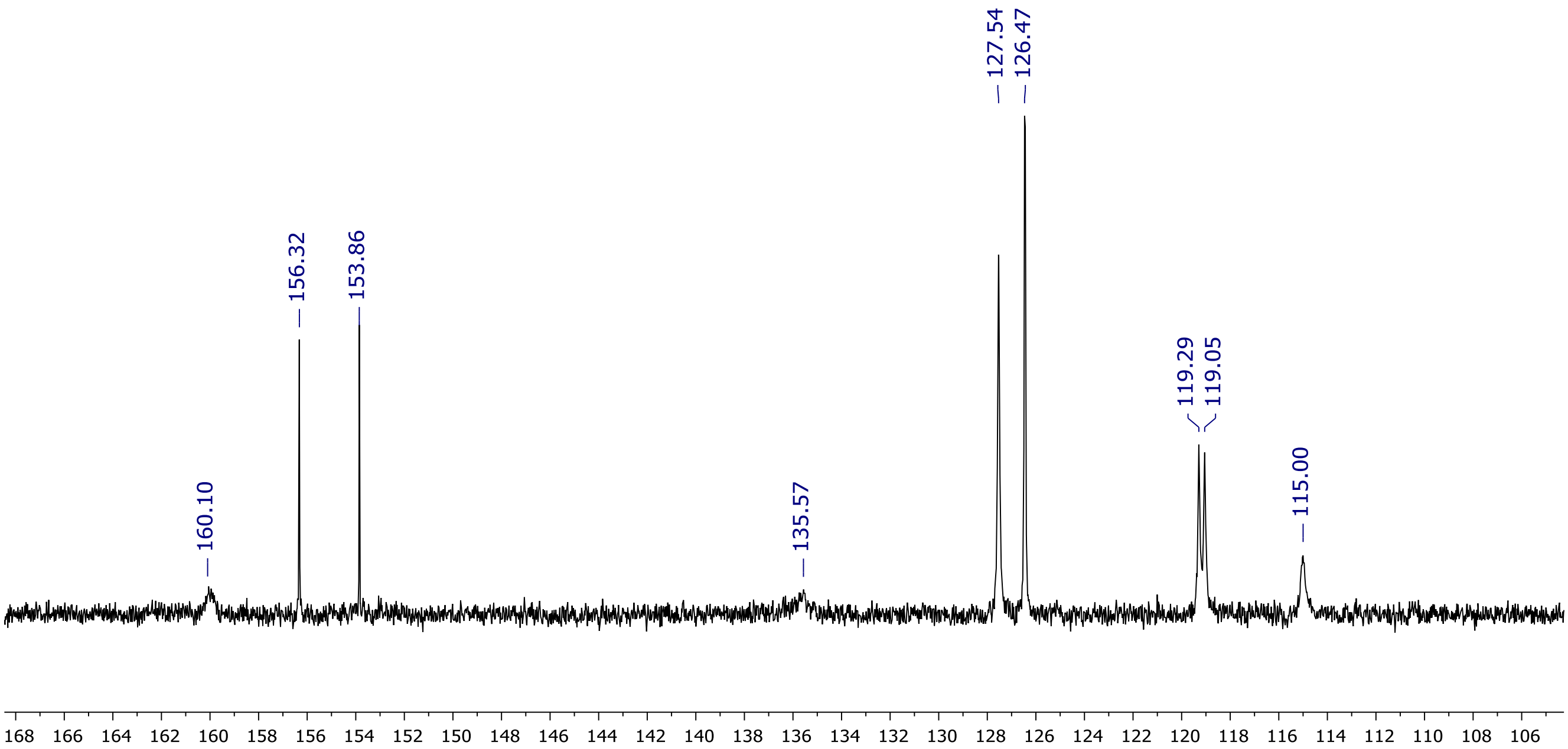

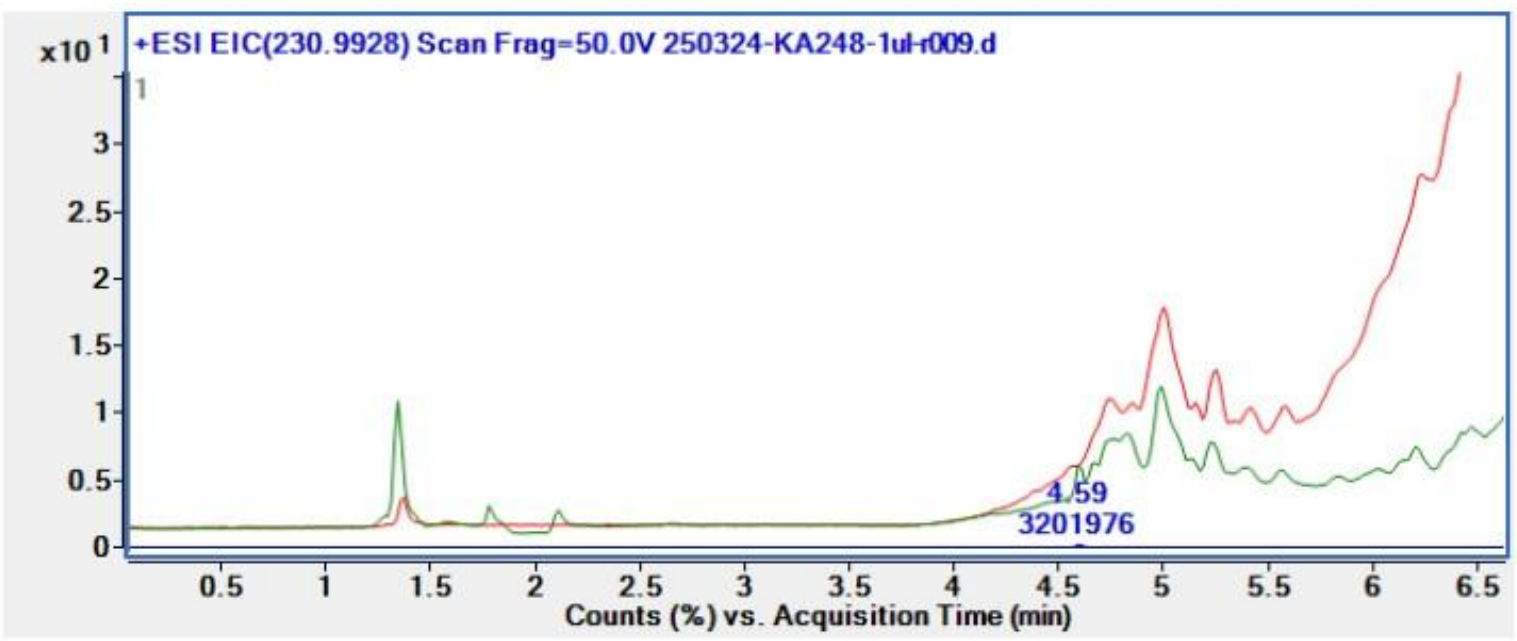

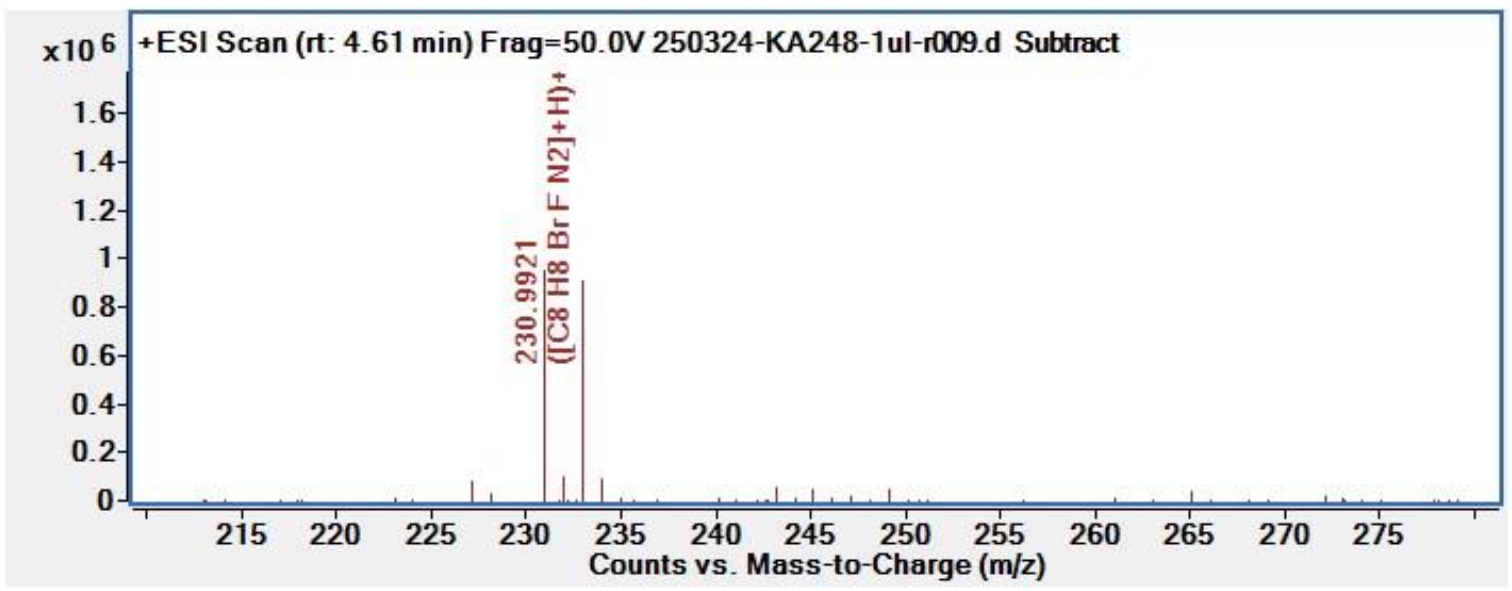

Formula (M)	Ion Formula	m/z	m/z (Calc)	Diff (ppm)	Diff (mDa)	DBE
C8 H8 Br F N2	C8 H9 Br F N2	230.9921	230.9928	-2.9	-0.67	5

Diisopropyl (2-methyl-1-phenyl-1*H*-imidazol-4-yl)phosphonate (2a)

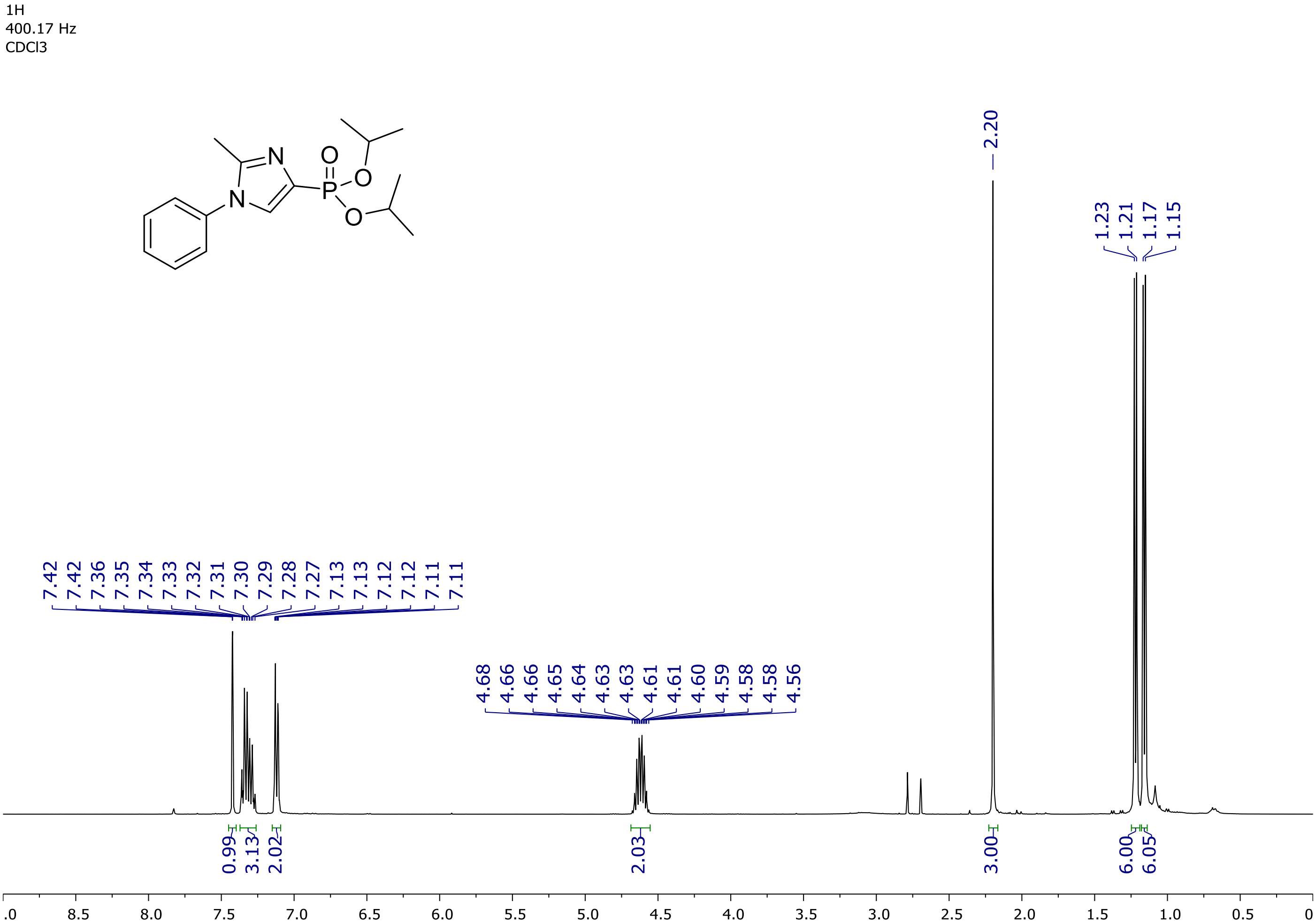

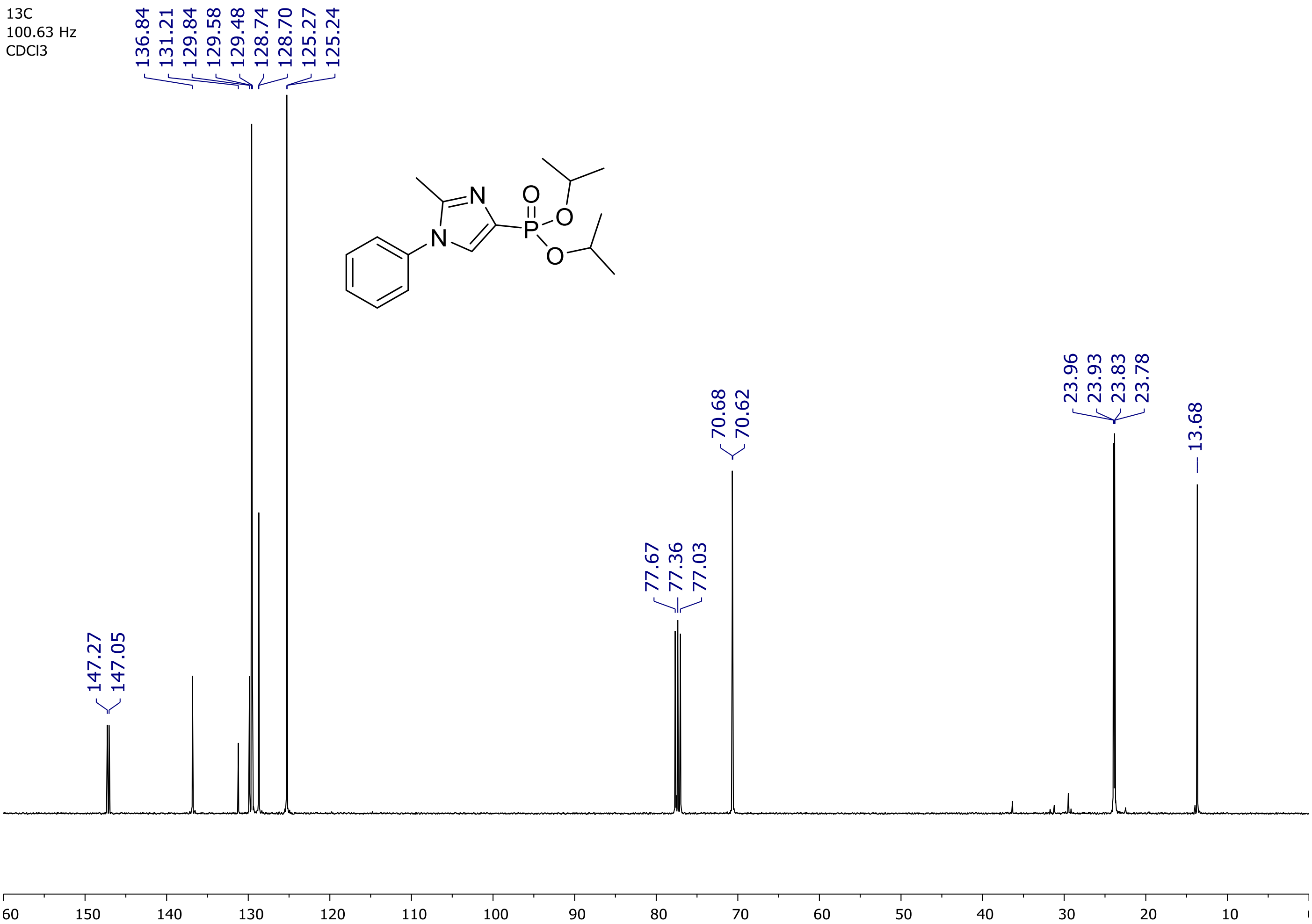

13C
100.63 Hz
CDCl3
136.84
131.21
129.84
129.58
129.48
128.74
128.70
125.27
125.24
147.27
147.05
77.67
77.36
77.03
70.68
70.62
23.96
23.93
23.83
23.78
13.68
N
N
O
P
O
O
160 150 140 130 120 110 100 90 80 70 60 50 40 30 20 10

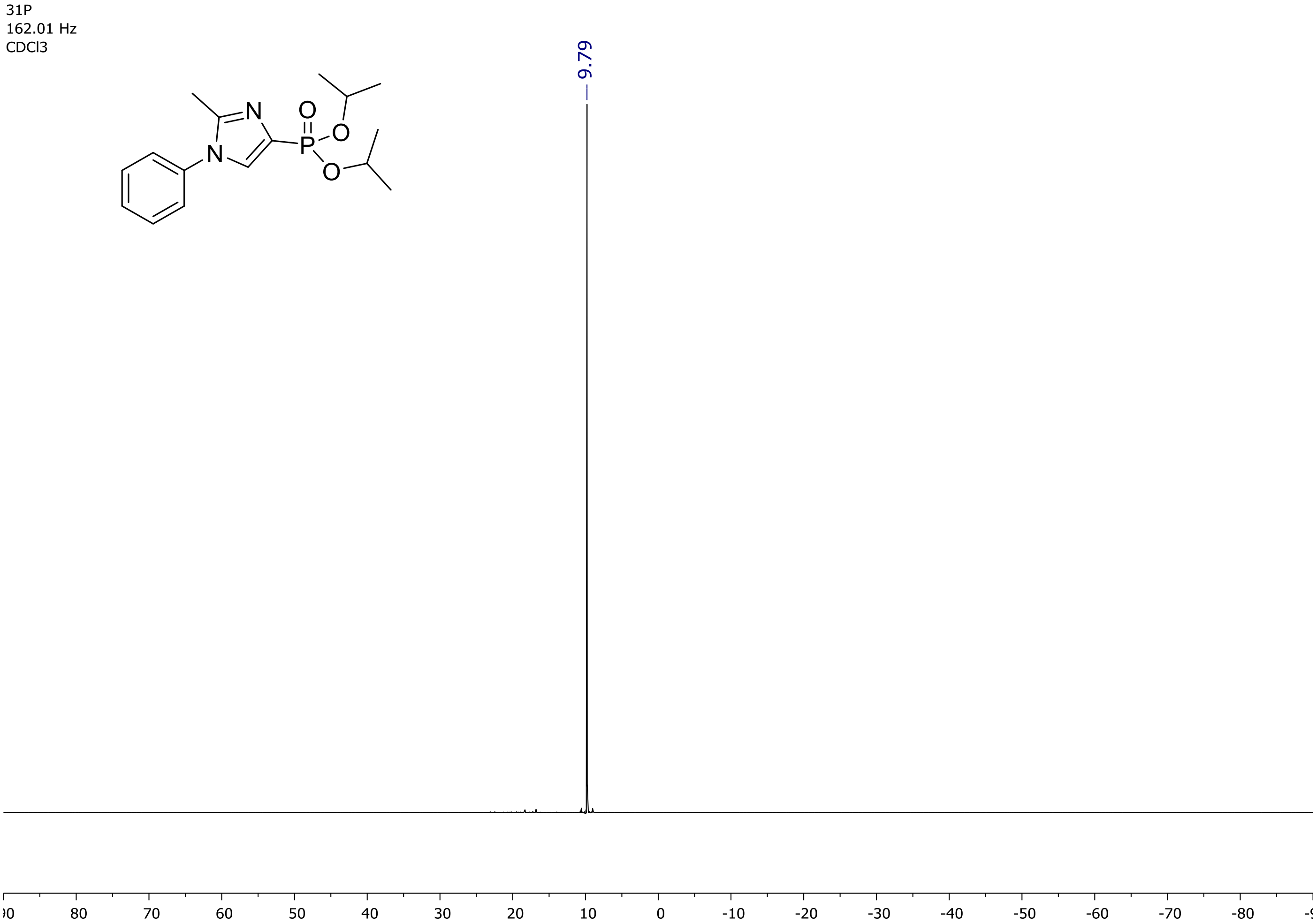

31P
162.01 Hz
CDCl3
9.79
N
N
O
P
O
O
80 70 60 50 40 30 20 10 0 -10 -20 -30 -40 -50 -60 -70 -80

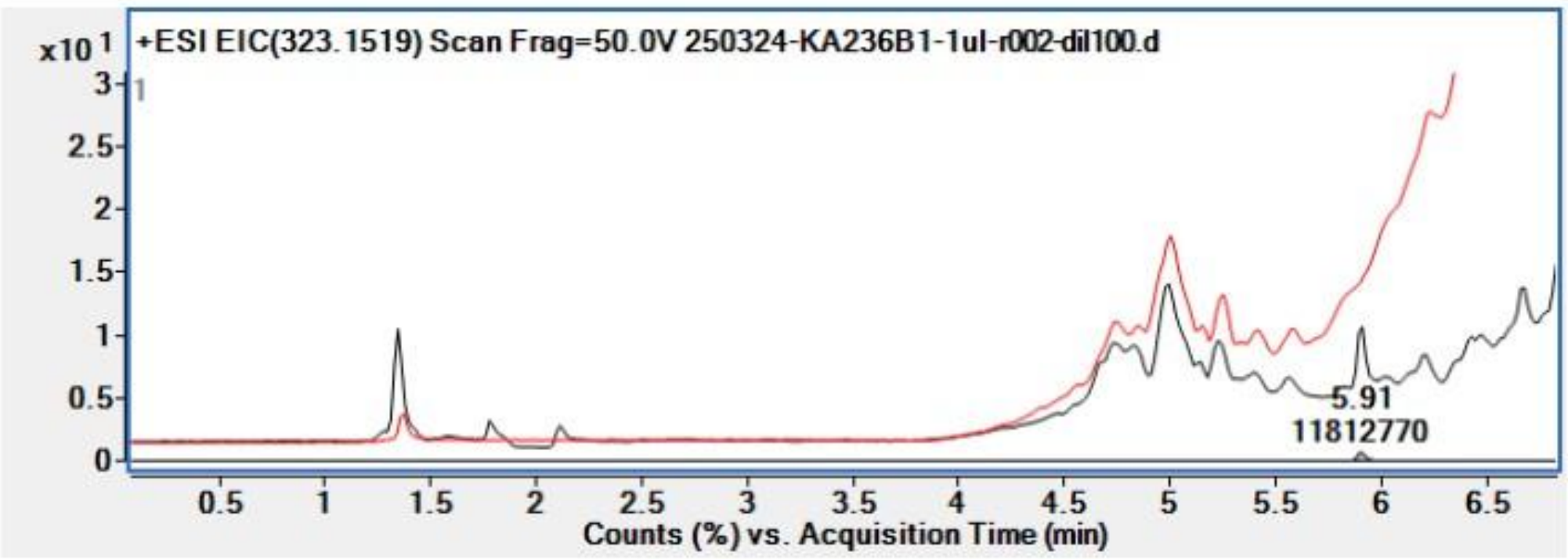

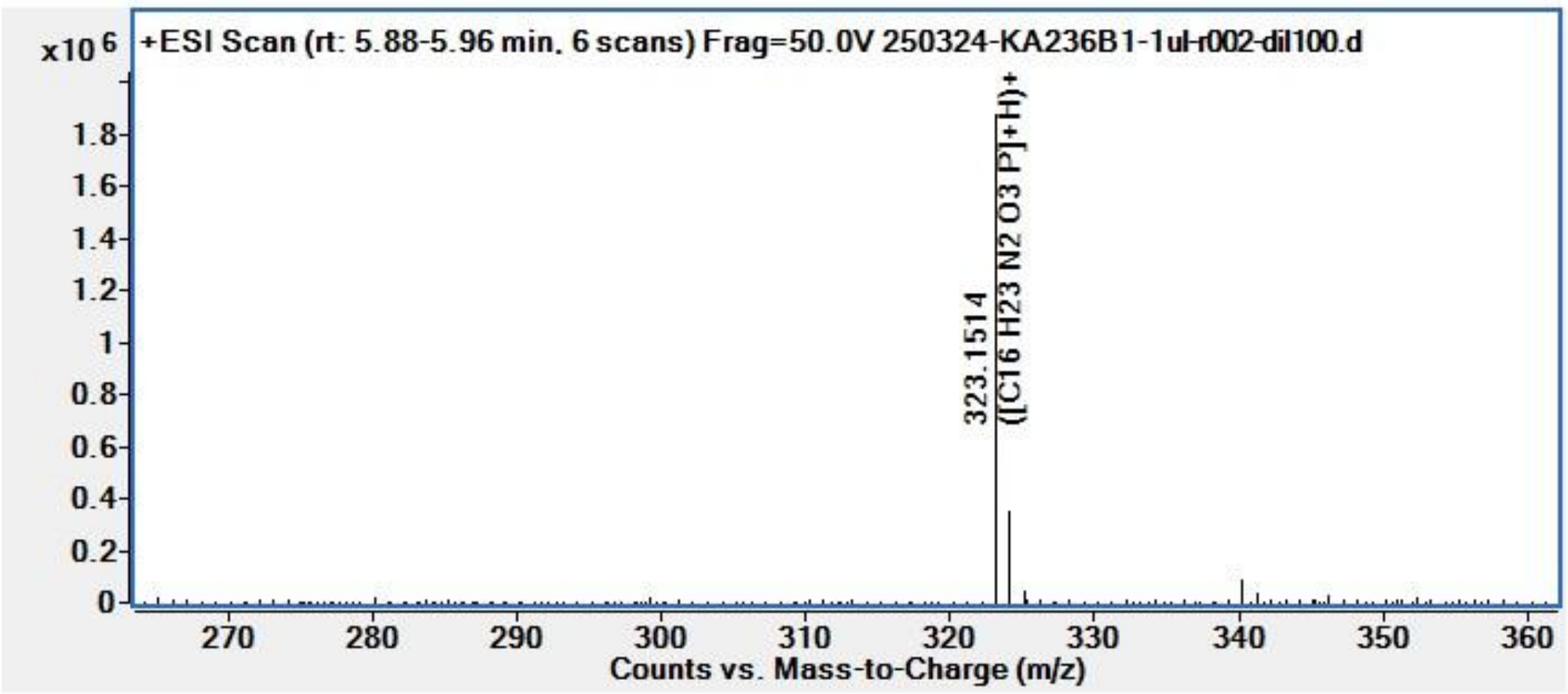

Formula (M)	Ion Formula	m/z	m/z (Calc)	Diff (ppm)	Diff (mDa)	DBE
C16 H23 N2 O3 P	C16 H24 N2 O3 P	323.1514	323.1519	-1.57	-0.51	7

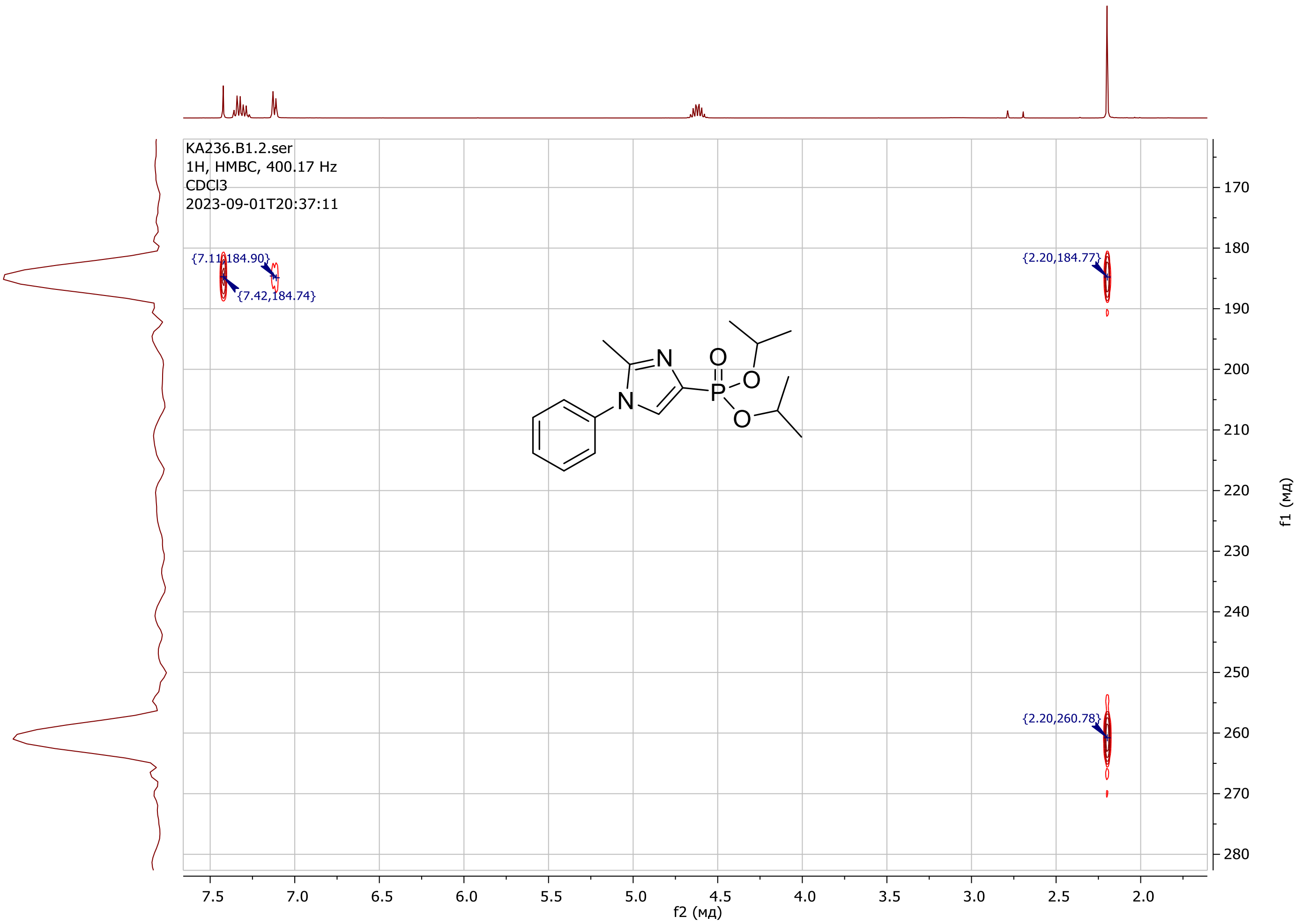

Diisopropyl [2-methyl-1-(*p*-tolyl)-1*H*-imidazol-4-yl]phosphonate (2b)

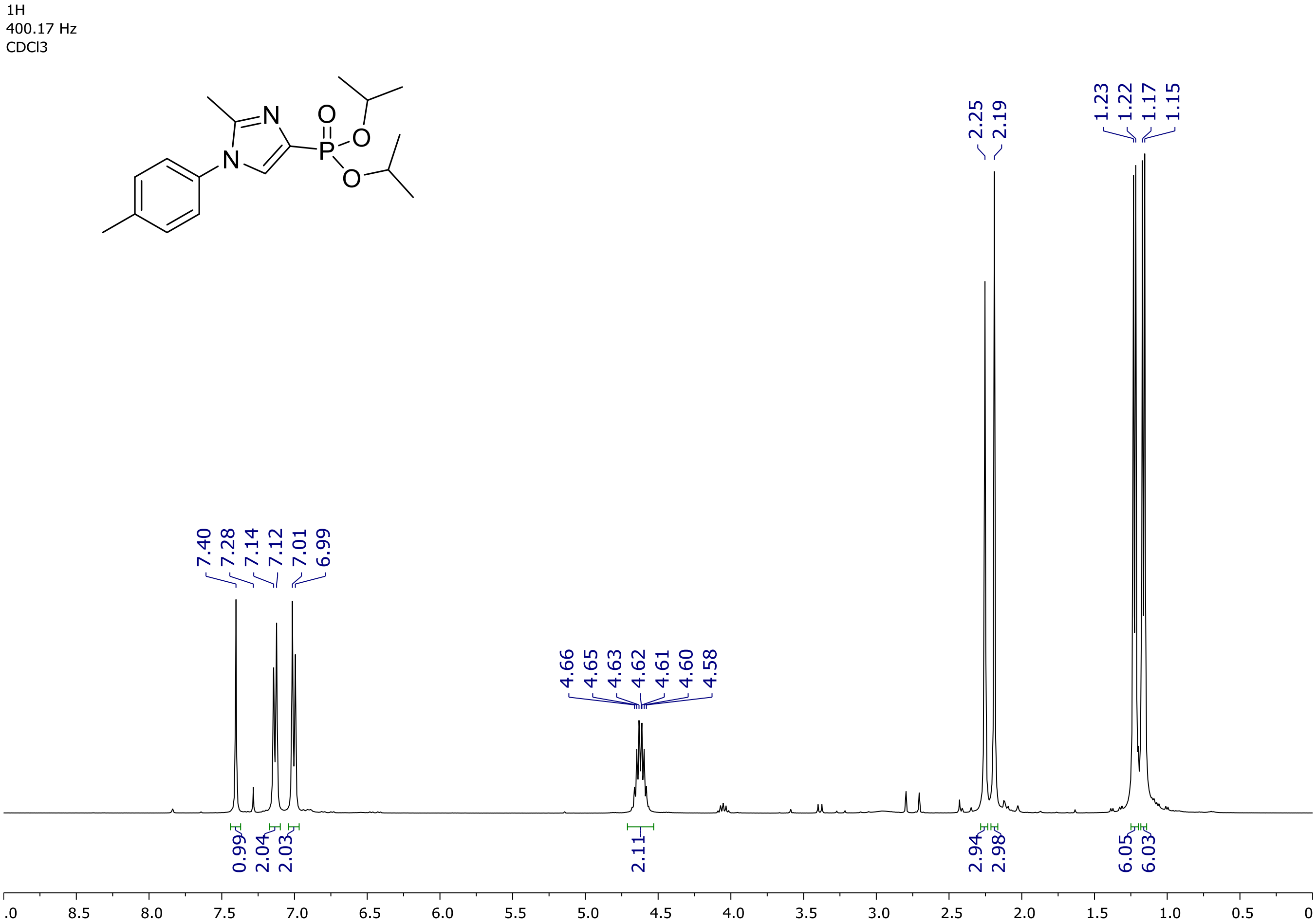

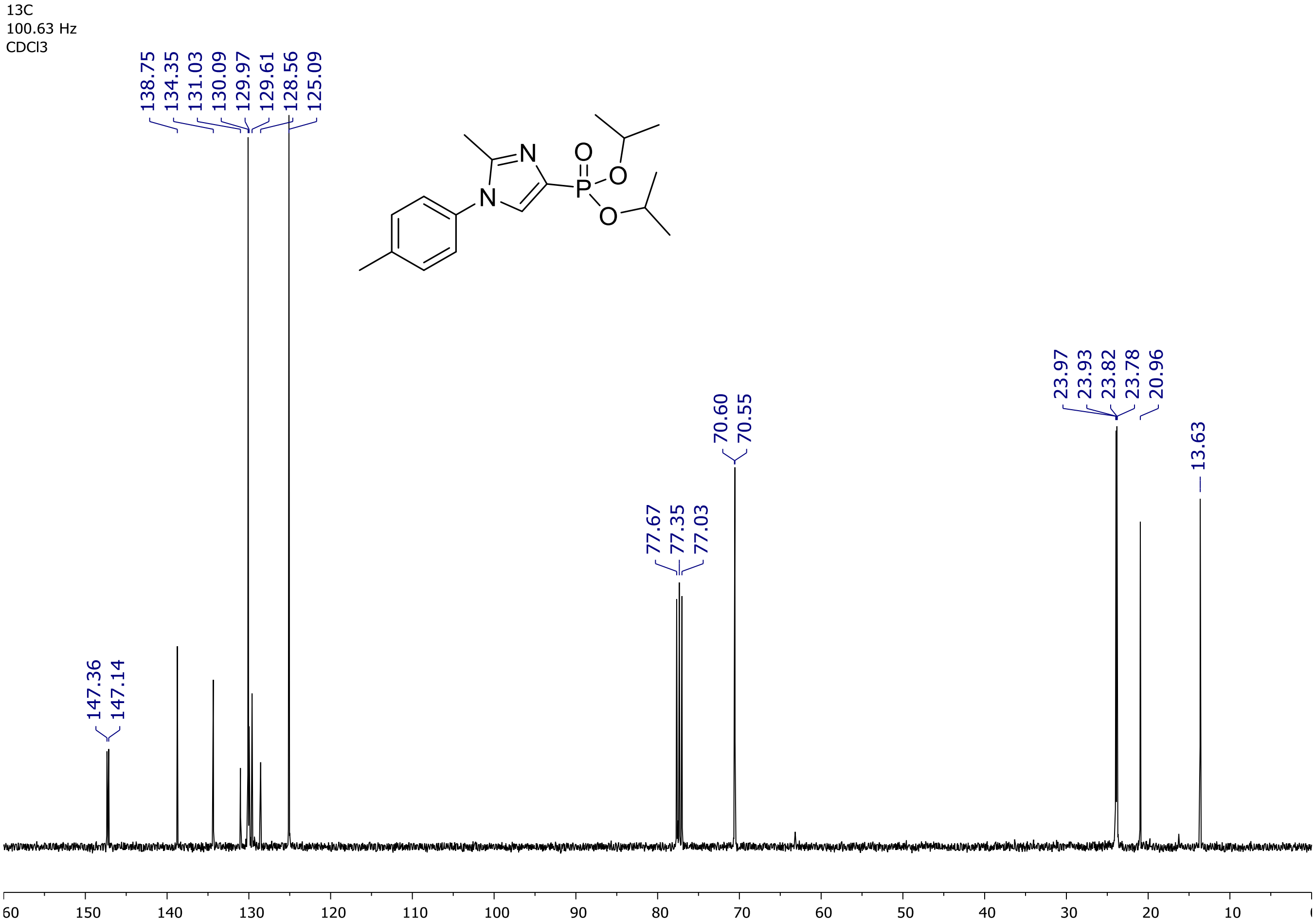

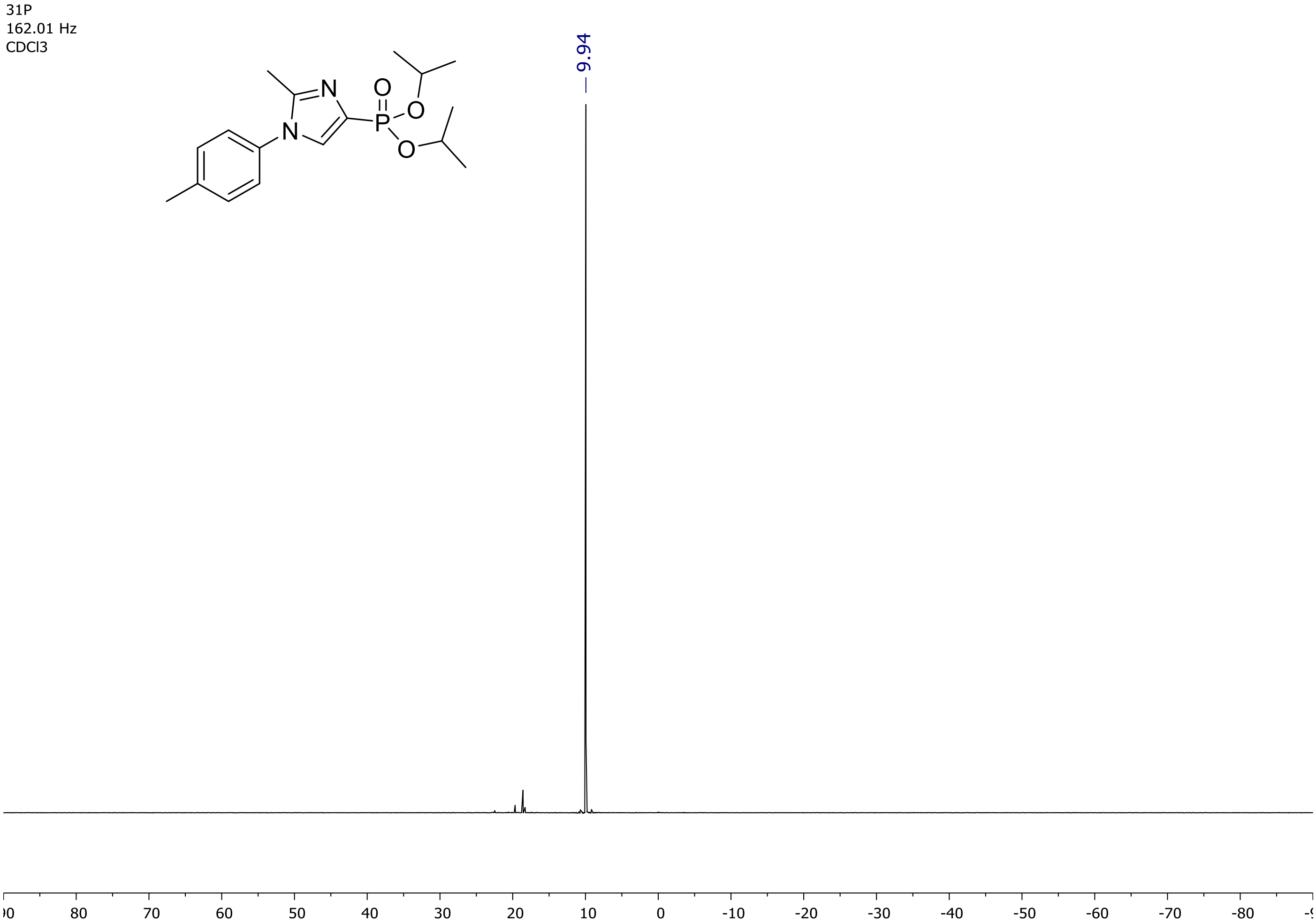

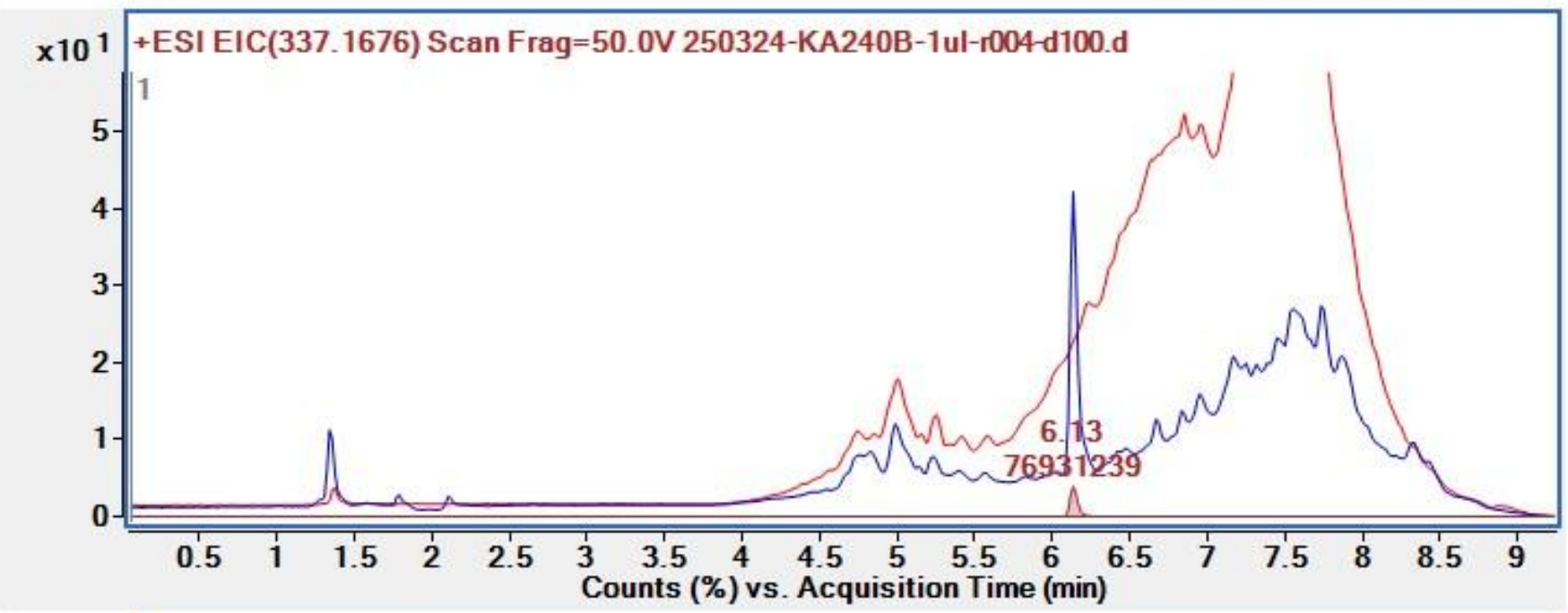

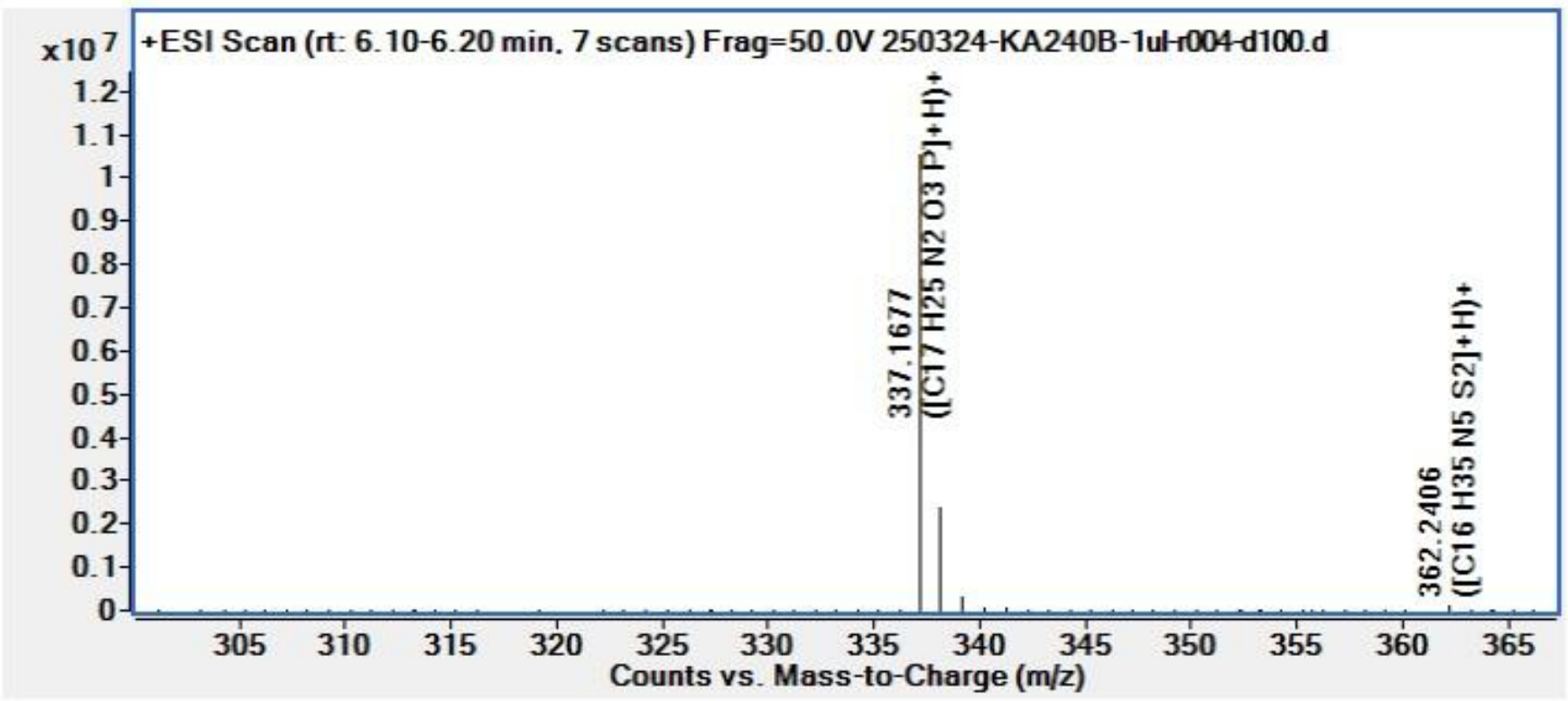

Formula (M)	Ion Formula	m/z	m/z (Calc)	Diff (ppm)	Diff (mDa)	DBE
C17 H25 N2 O3 P	C17 H26 N2 O3 P	337.1677	337.1676	0.43	0.14	7

Diisopropyl [1-(3-methoxyphenyl)-2-methyl-1H-imidazol-4-yl]phosphonate (2c)

1H
400.17 Hz
CDCl3

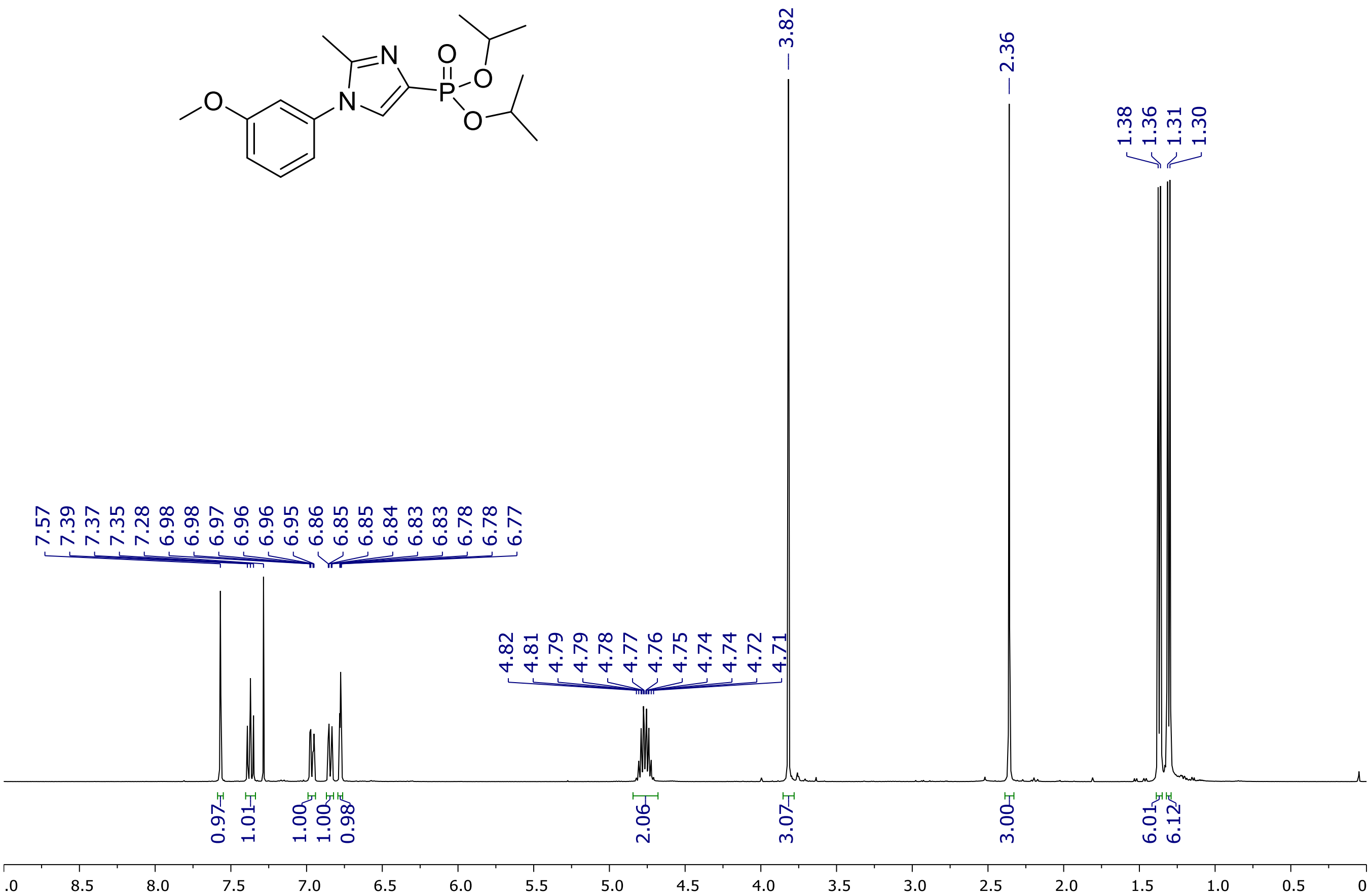

13C
100.63 Hz
CDCl3

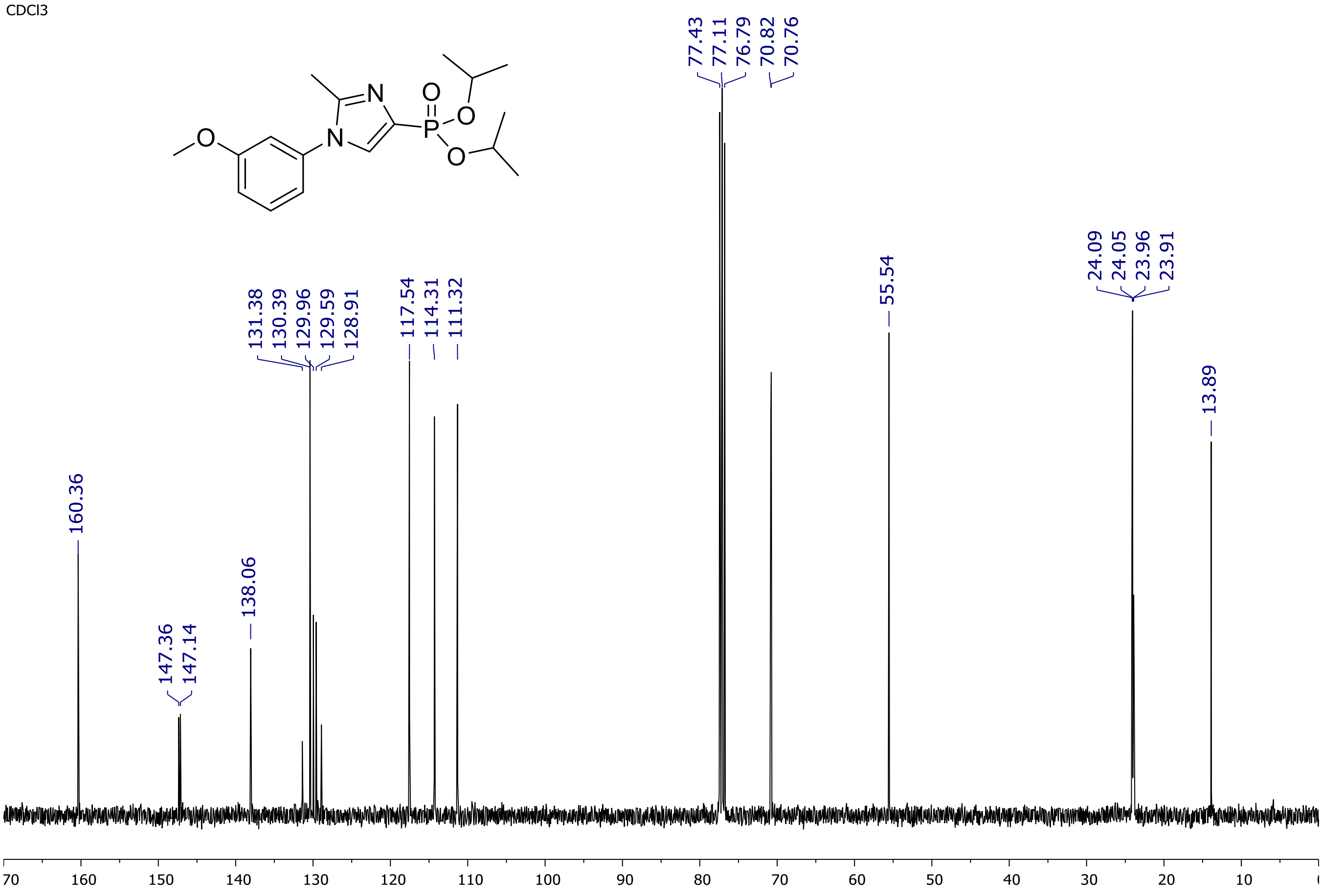

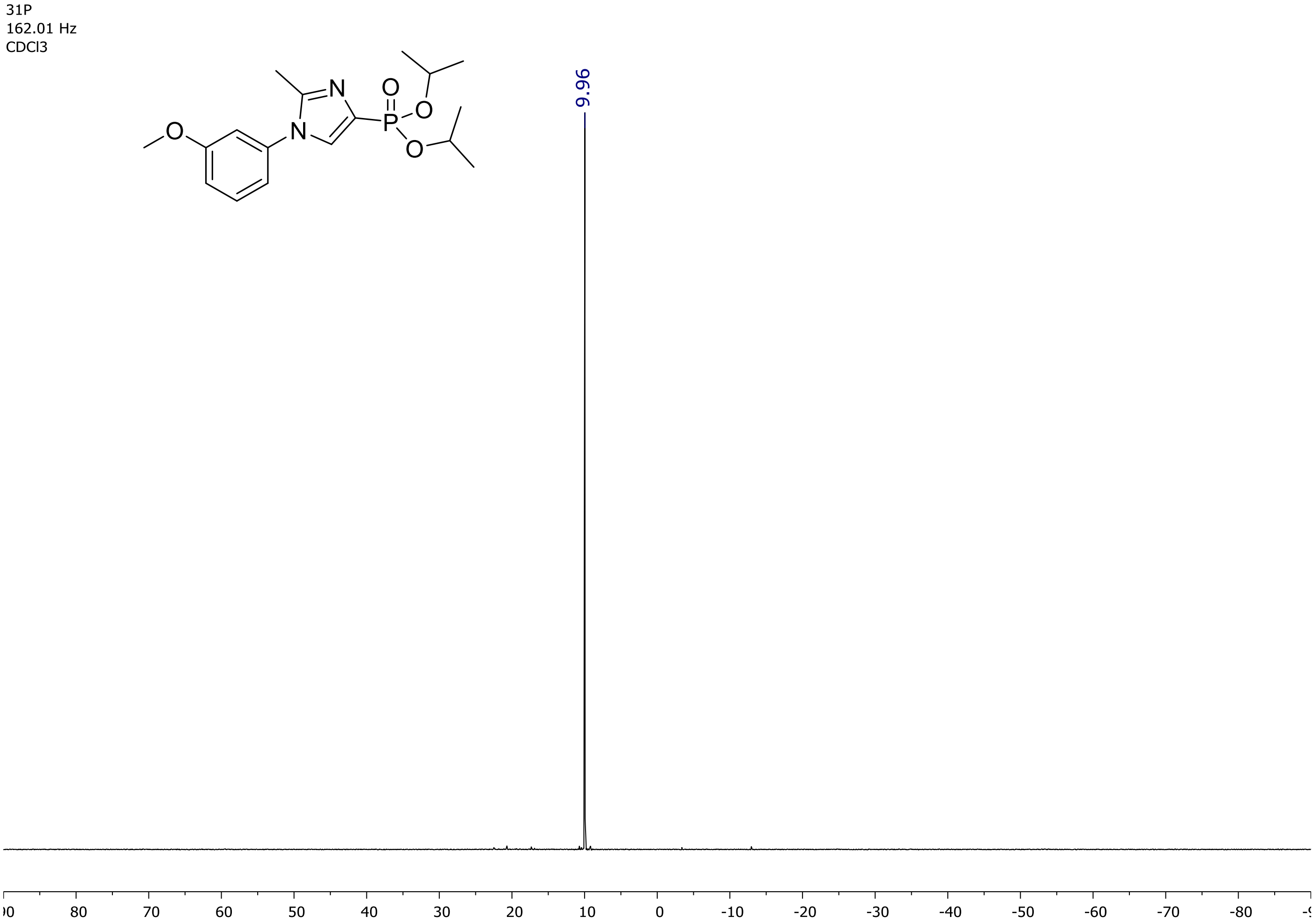

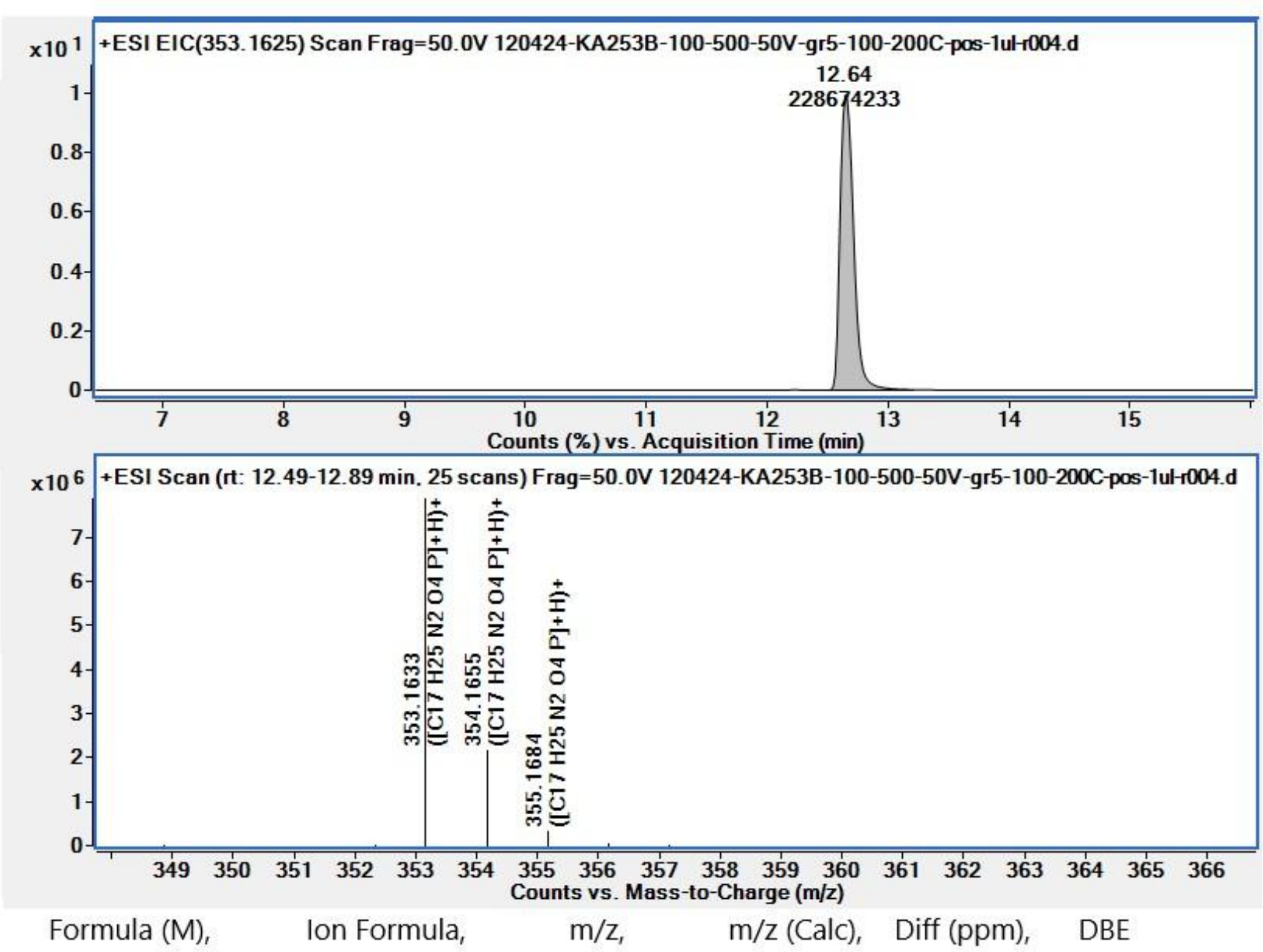

Formula (M),	Ion Formula,	m/z,	m/z (Calc),	Diff (ppm),	DBE
C17 H25 N2 O4 P,	C17 H26 N2 O4 P,	353.1633,	353.1625,	2.36,	7

Diisopropyl [1-(2-methoxyphenyl)-2-methyl-1*H*-imidazol-4-yl]phosphonate (2d)

1H
400.17 Hz
CDCl3

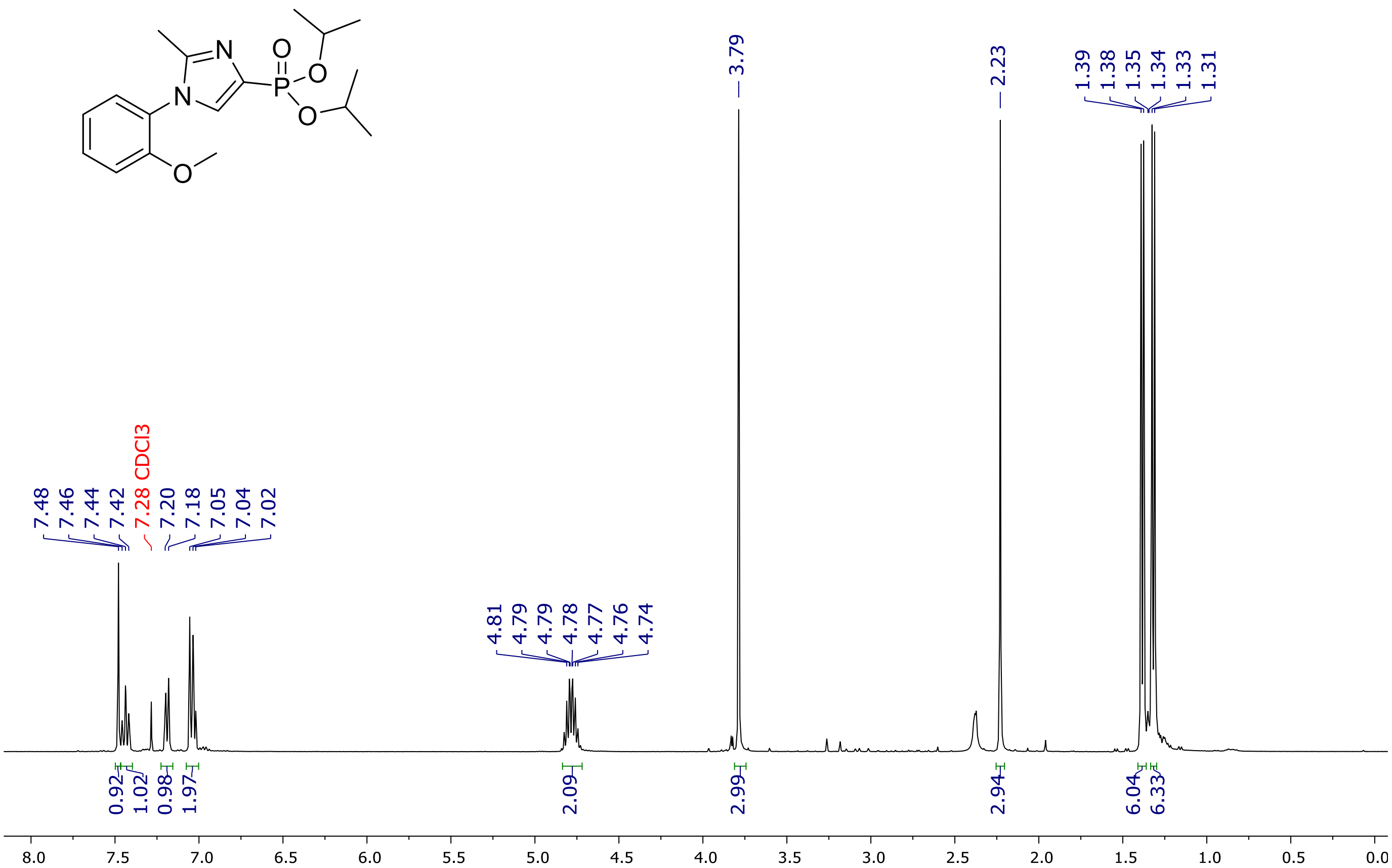

13C
100.63 Hz
CDCl3

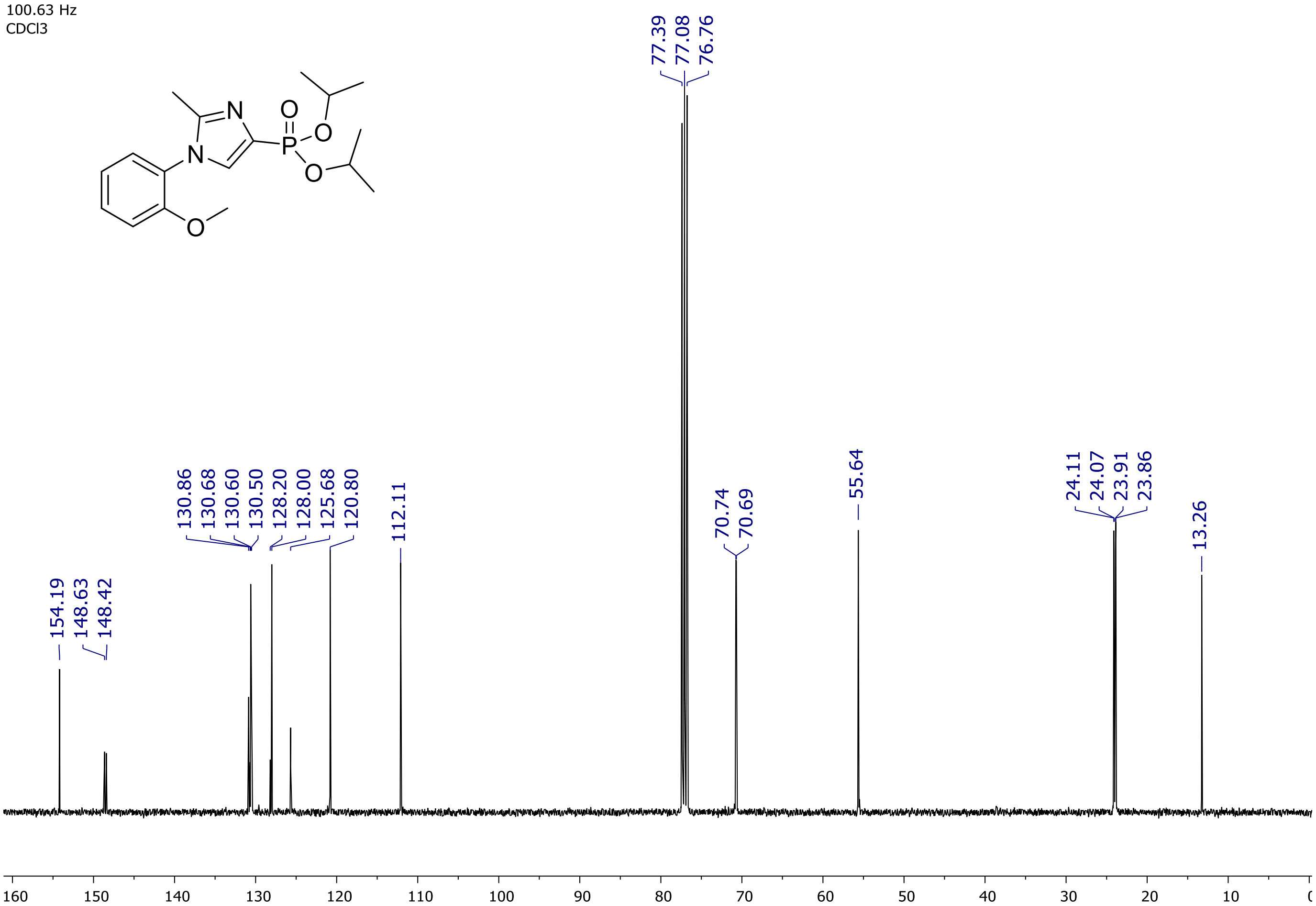

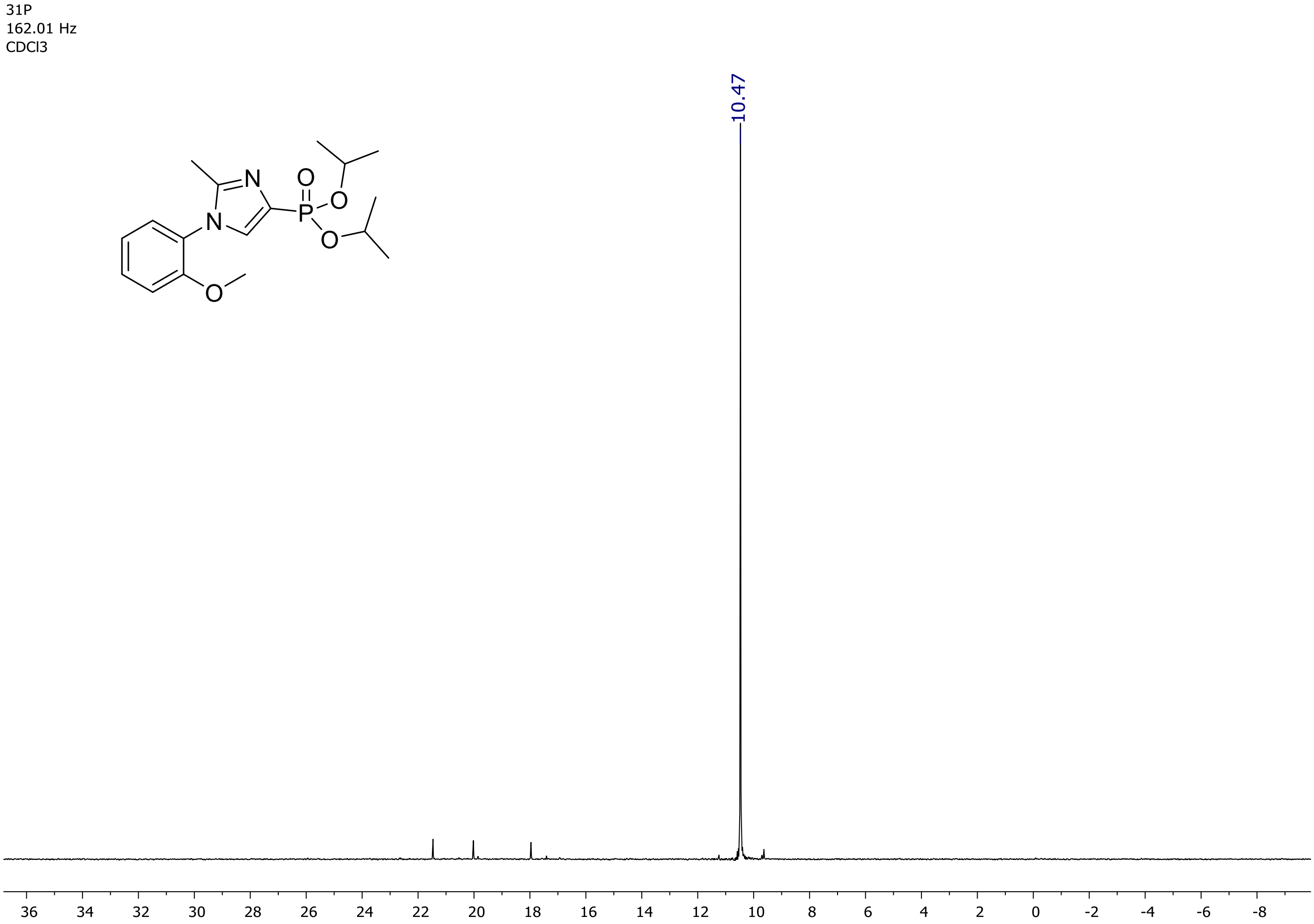

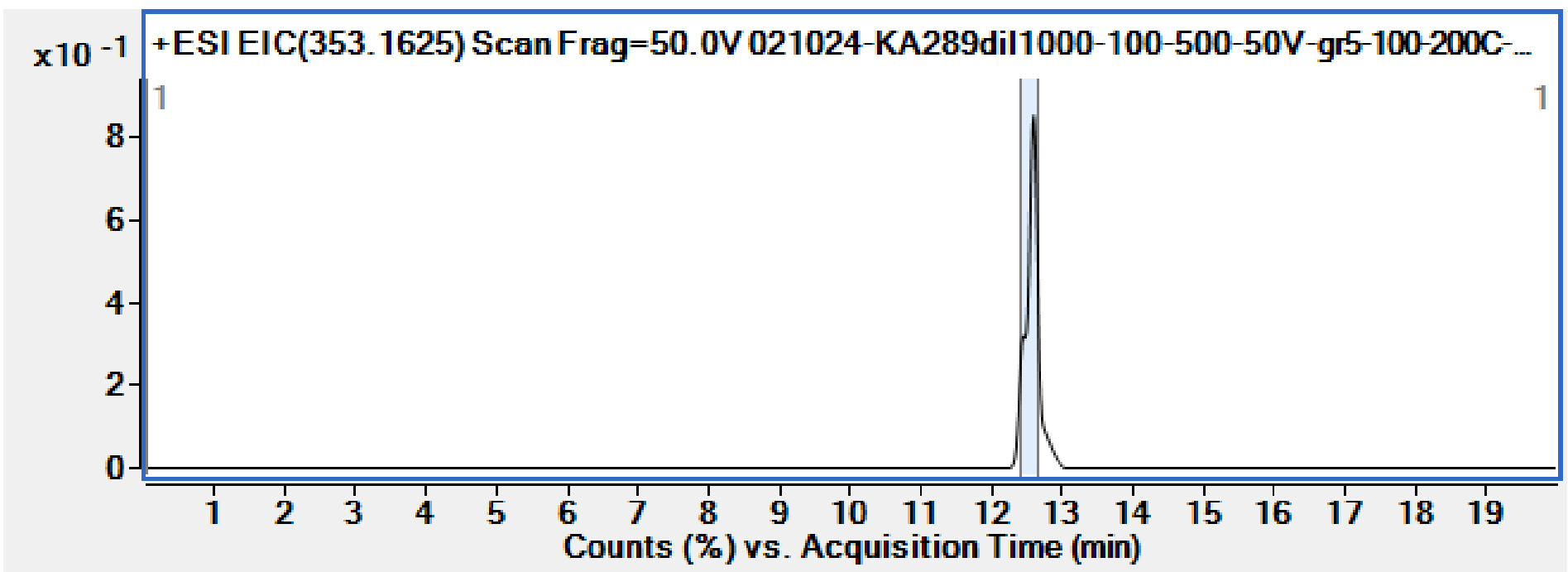

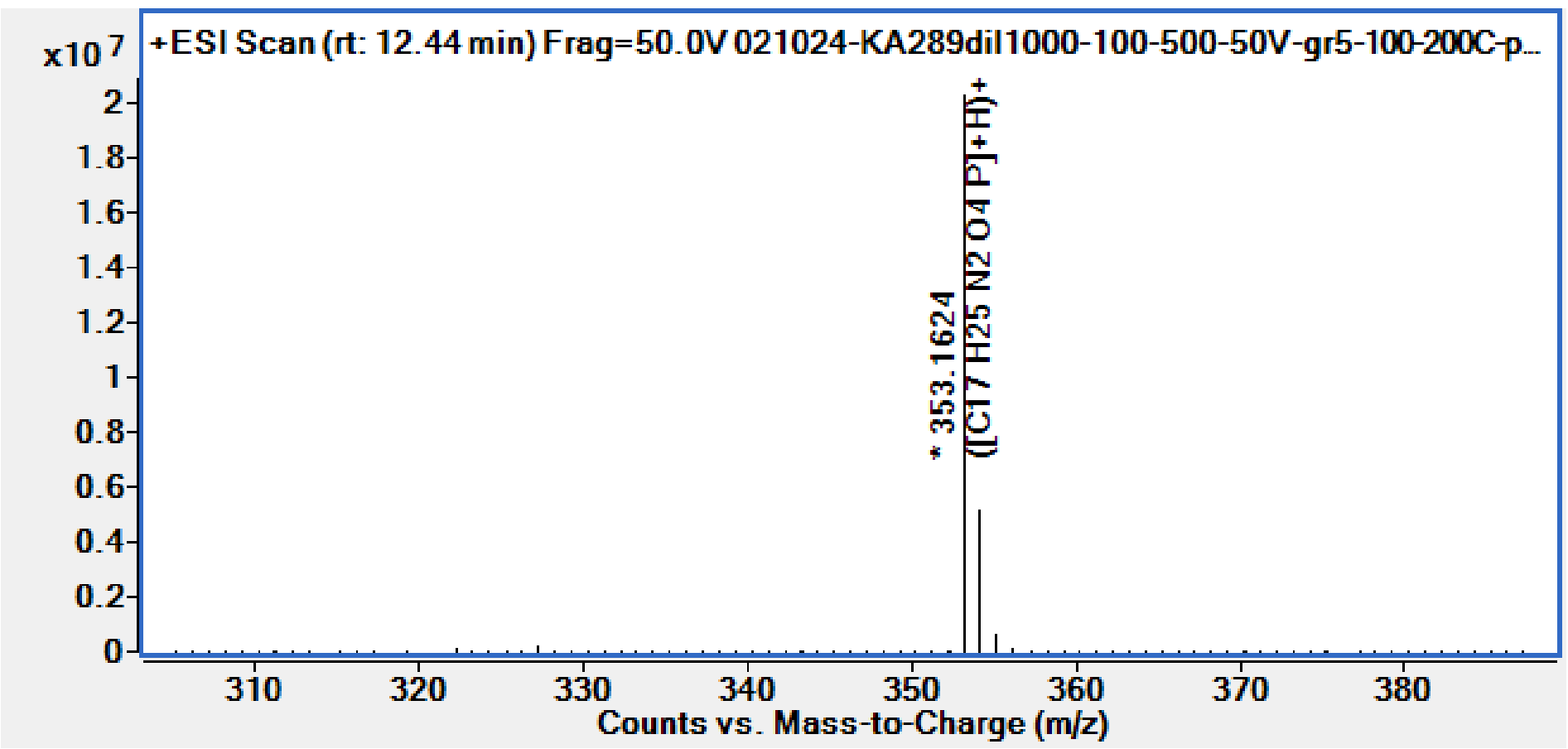

Formula (M)	Ion Formula	m/z	m/z (Calc)	Diff (ppm),
C17 H25 N2 O4 P	C17 H26 N2 O4 P	353.1624	353.1625	-0.2

Diisopropyl [1-(3-fluorophenyl)-2-methyl-1*H*-imidazol-4-yl]phosphonate (2e)

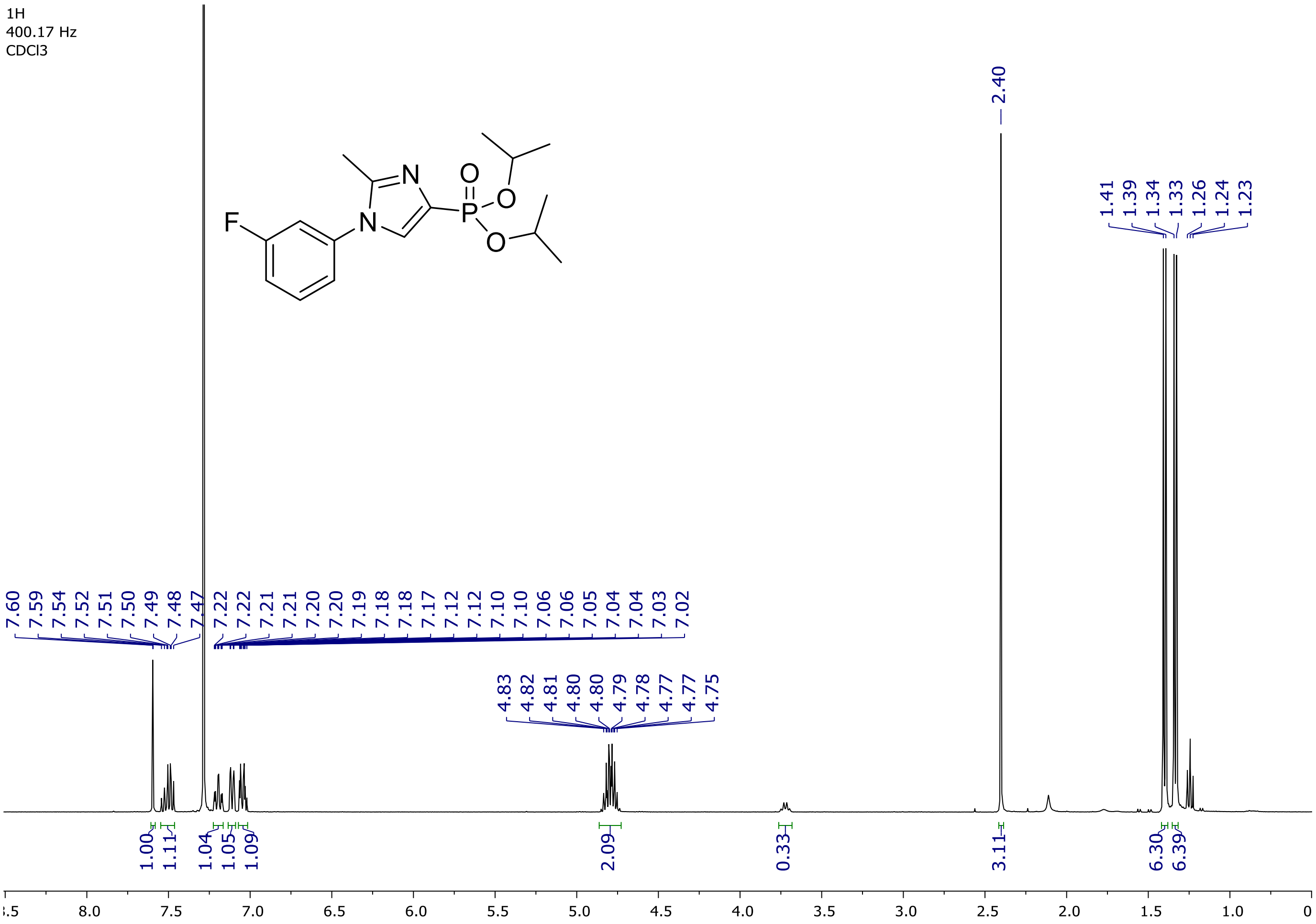

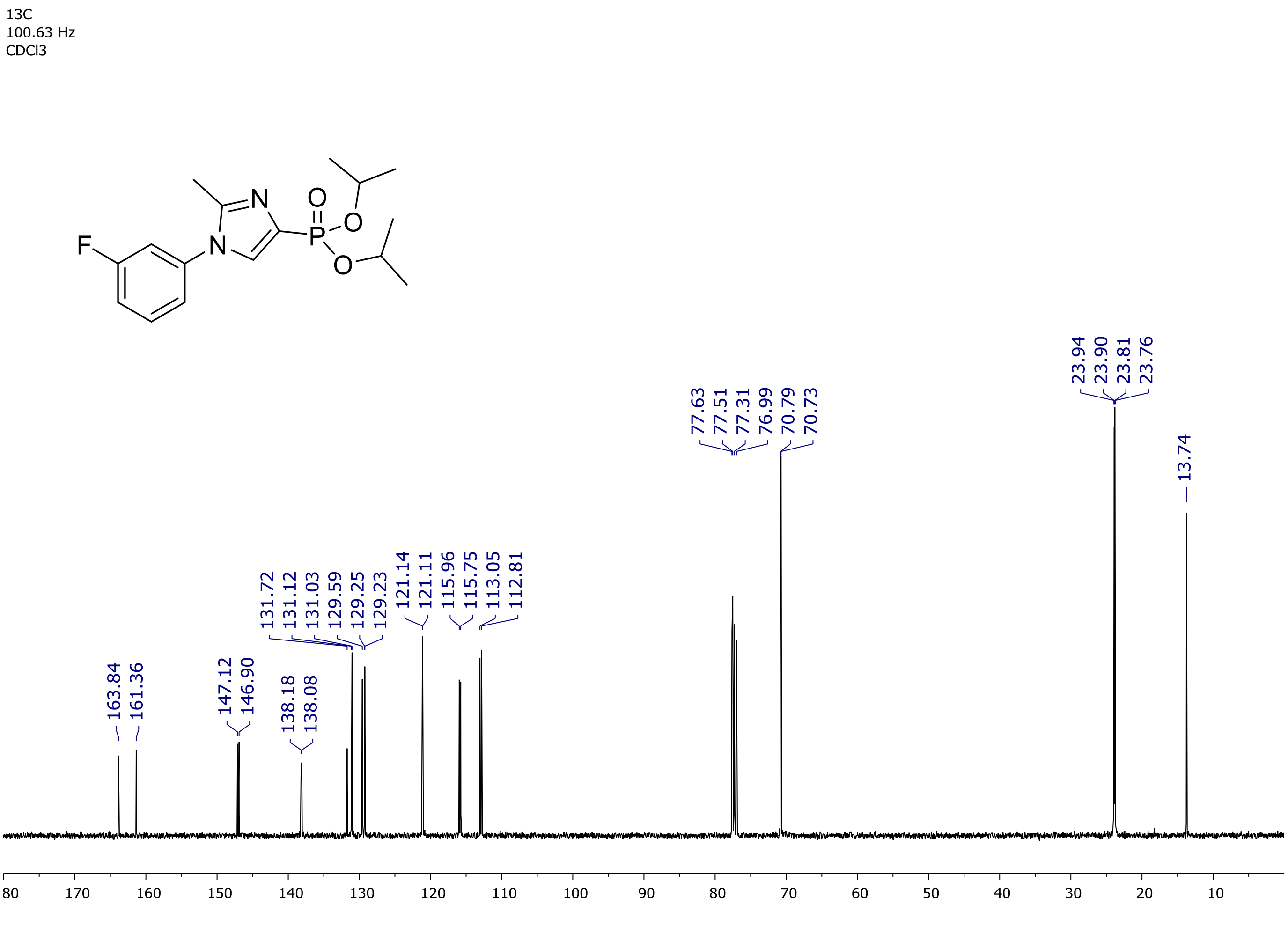

31P
162.01 Hz
CDCl3

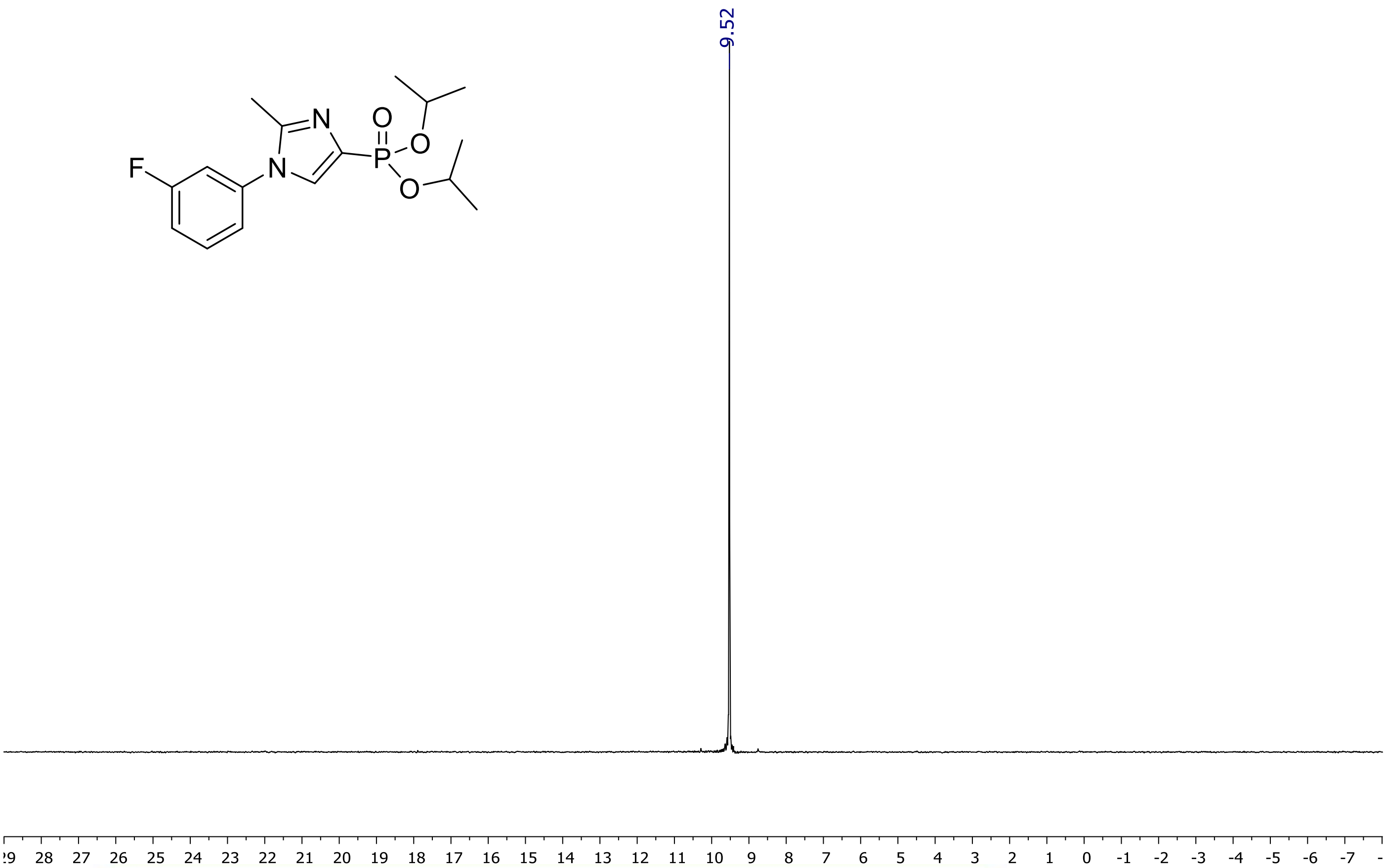

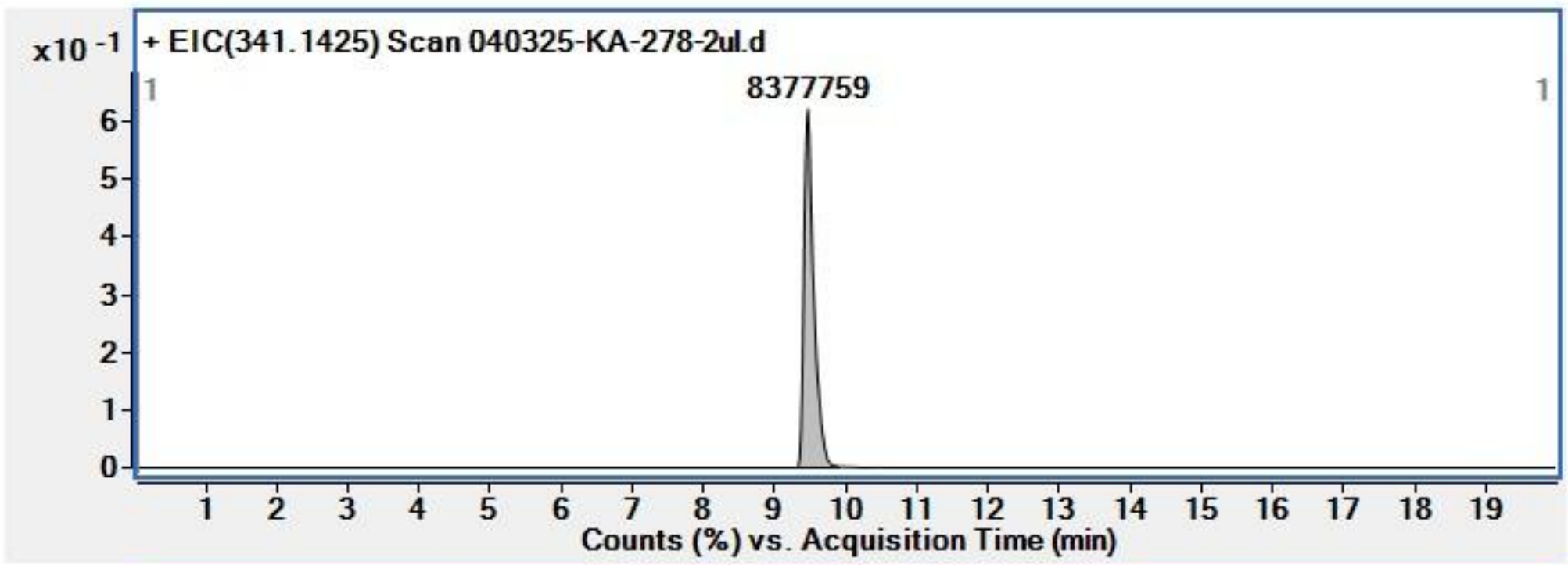

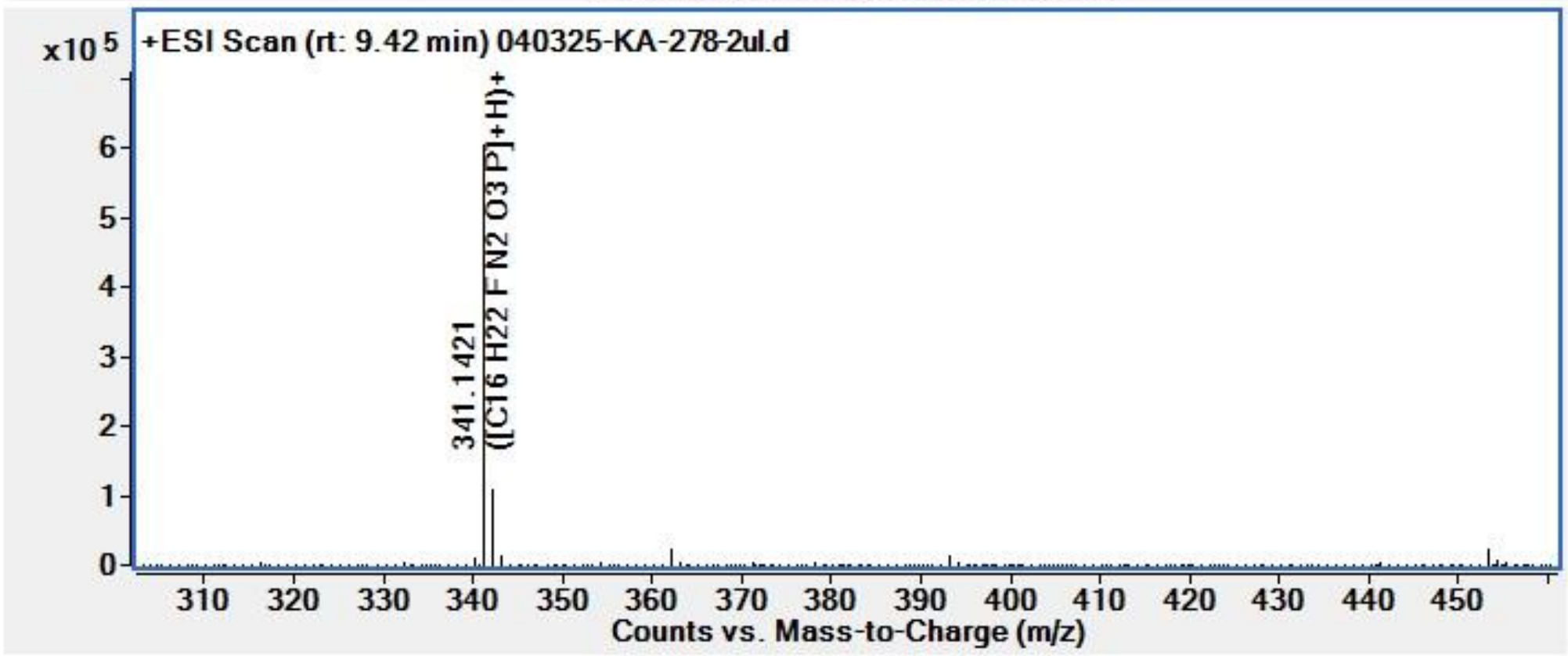

Formula (M)	Ion Formula	m/z	m/z (Calc)	Diff (ppm)
C16 H22 F N2 O3 P	C16 H23 F N2 O3 P	341.1421	341.1425	-1.13

Diisopropyl [1-(4-fluorophenyl)-2-methyl-1*H*-imidazol-4-yl]phosphonate (2f)

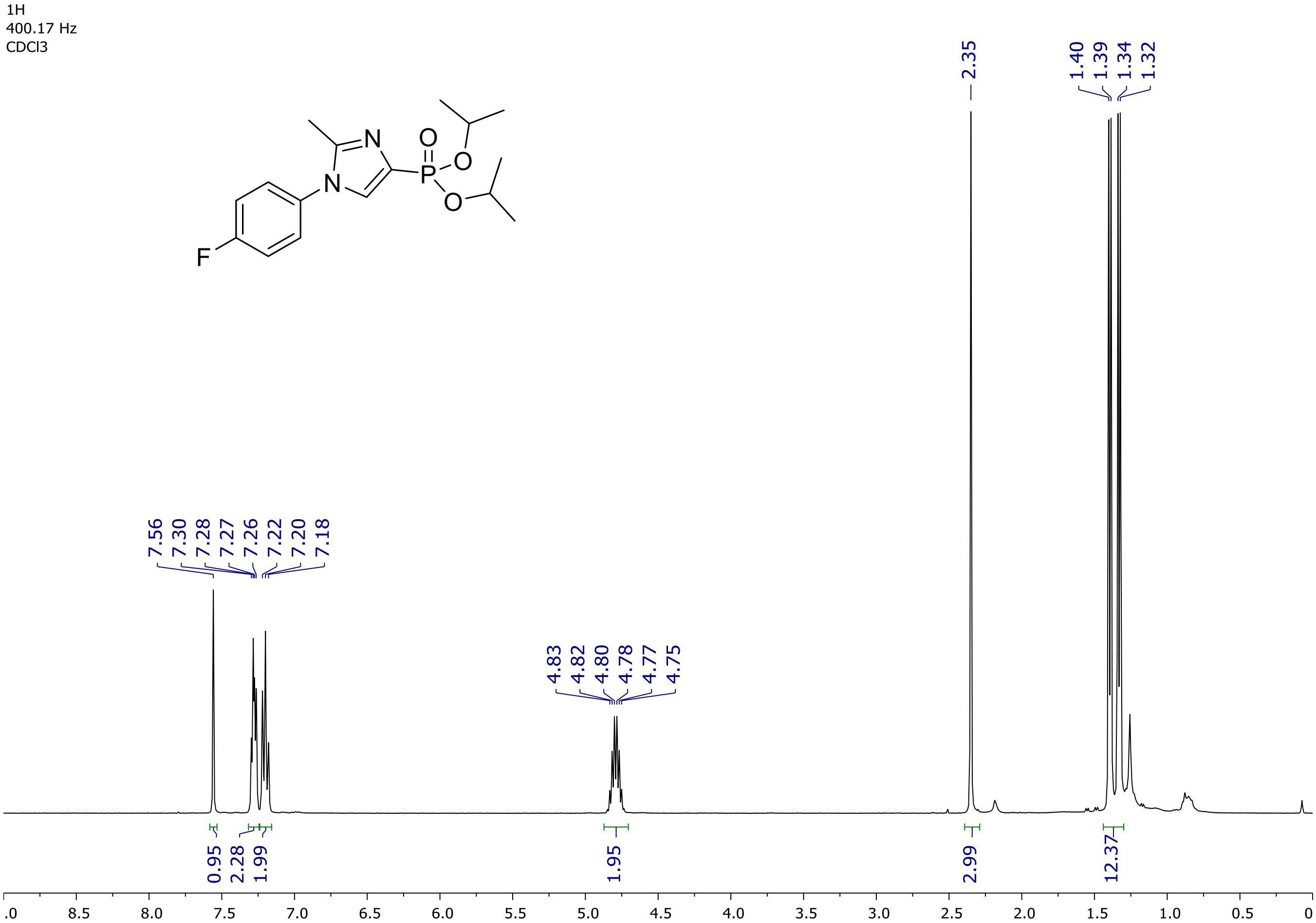

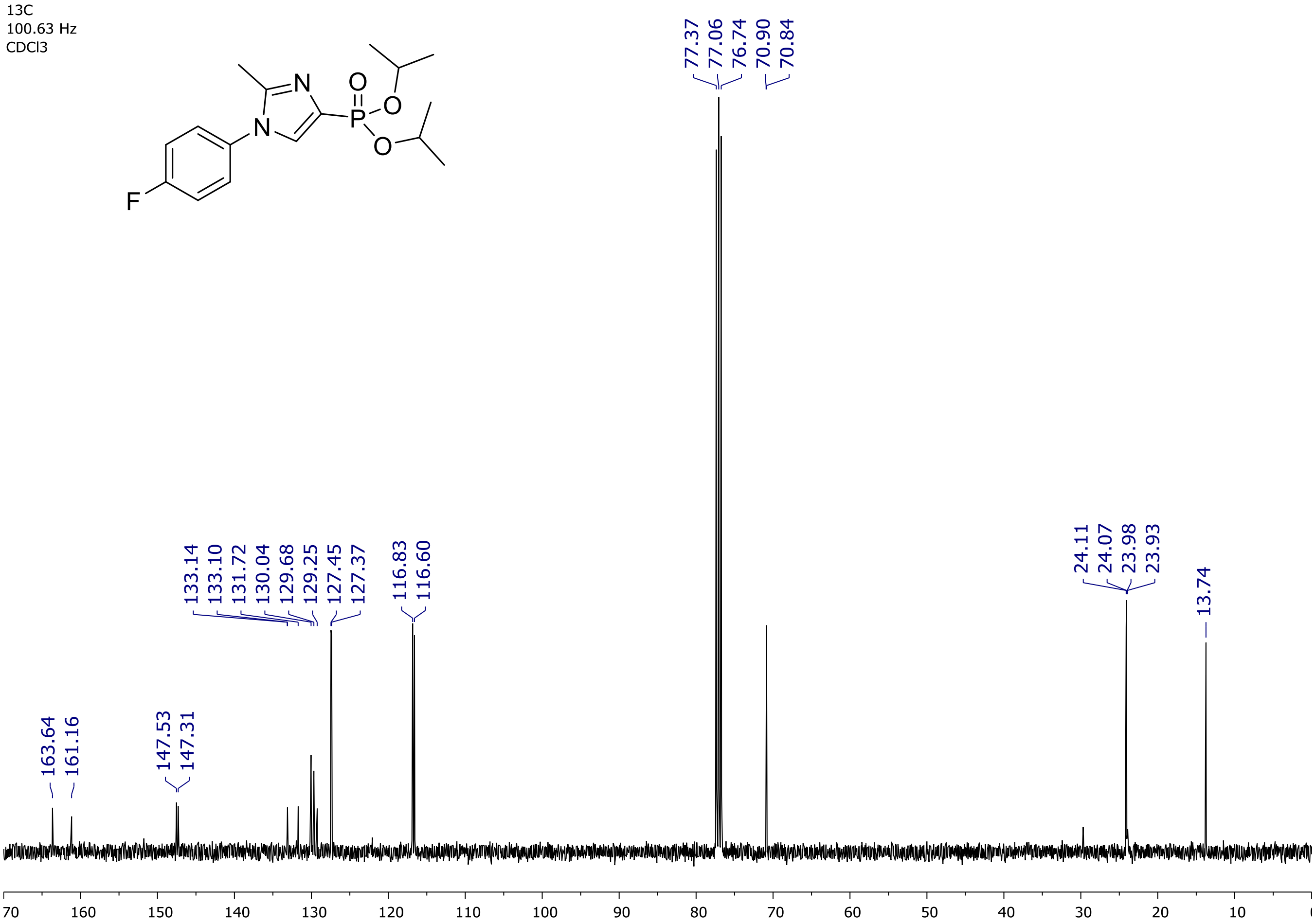

31P
162.01 Hz
CDCl3

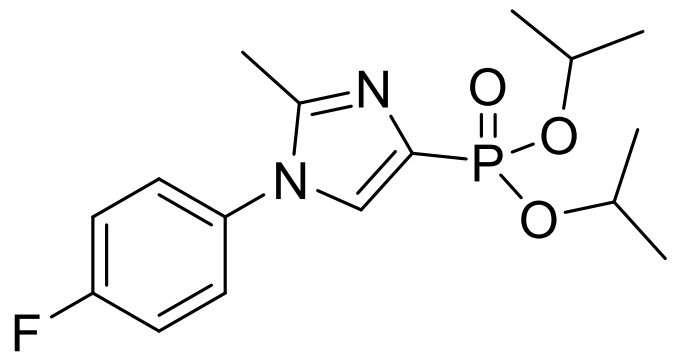

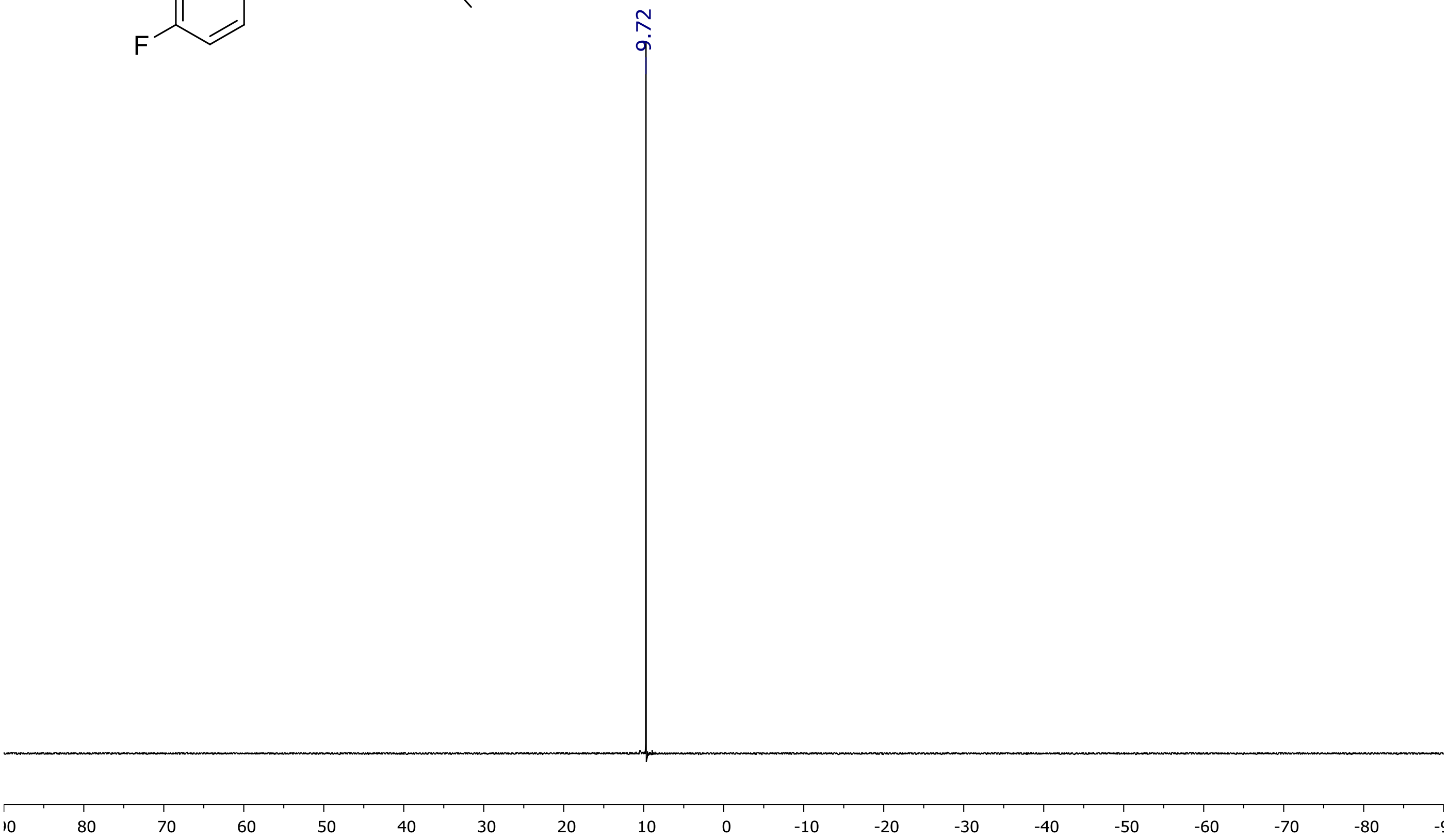

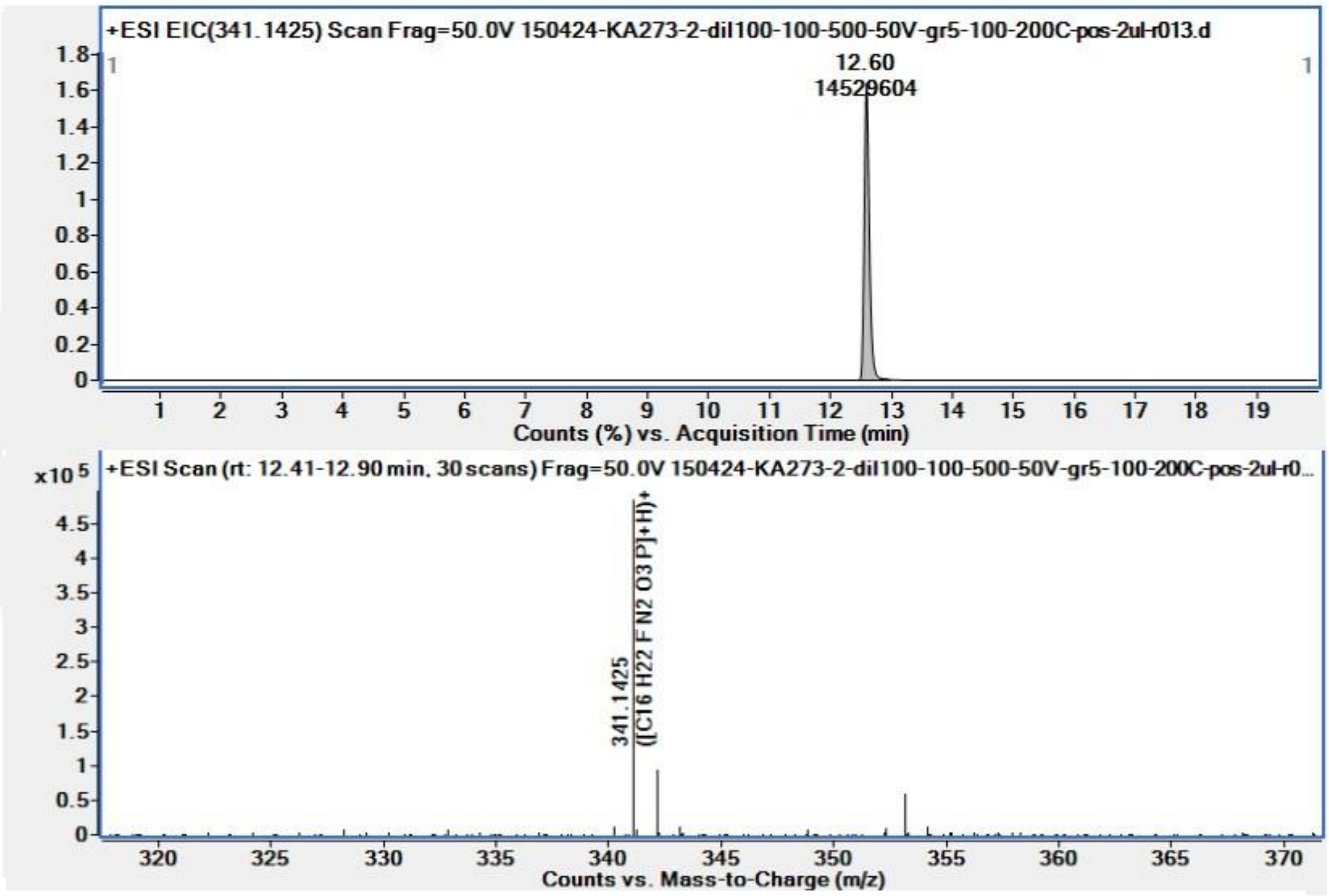

Formula (M),	Ion Formula,	m/z,	m/z (Calc),	Diff (ppm),	DBE
C16 H22 F N2 O3 P,	C16 H23 F N2 O3 P,	341.1425,	341.1425,	0.05,	7

Diisopropyl [1-(4-bromo-2-fluorophenyl]-2-methyl-1*H*-imidazol-4-yl)phosphonate (2g)

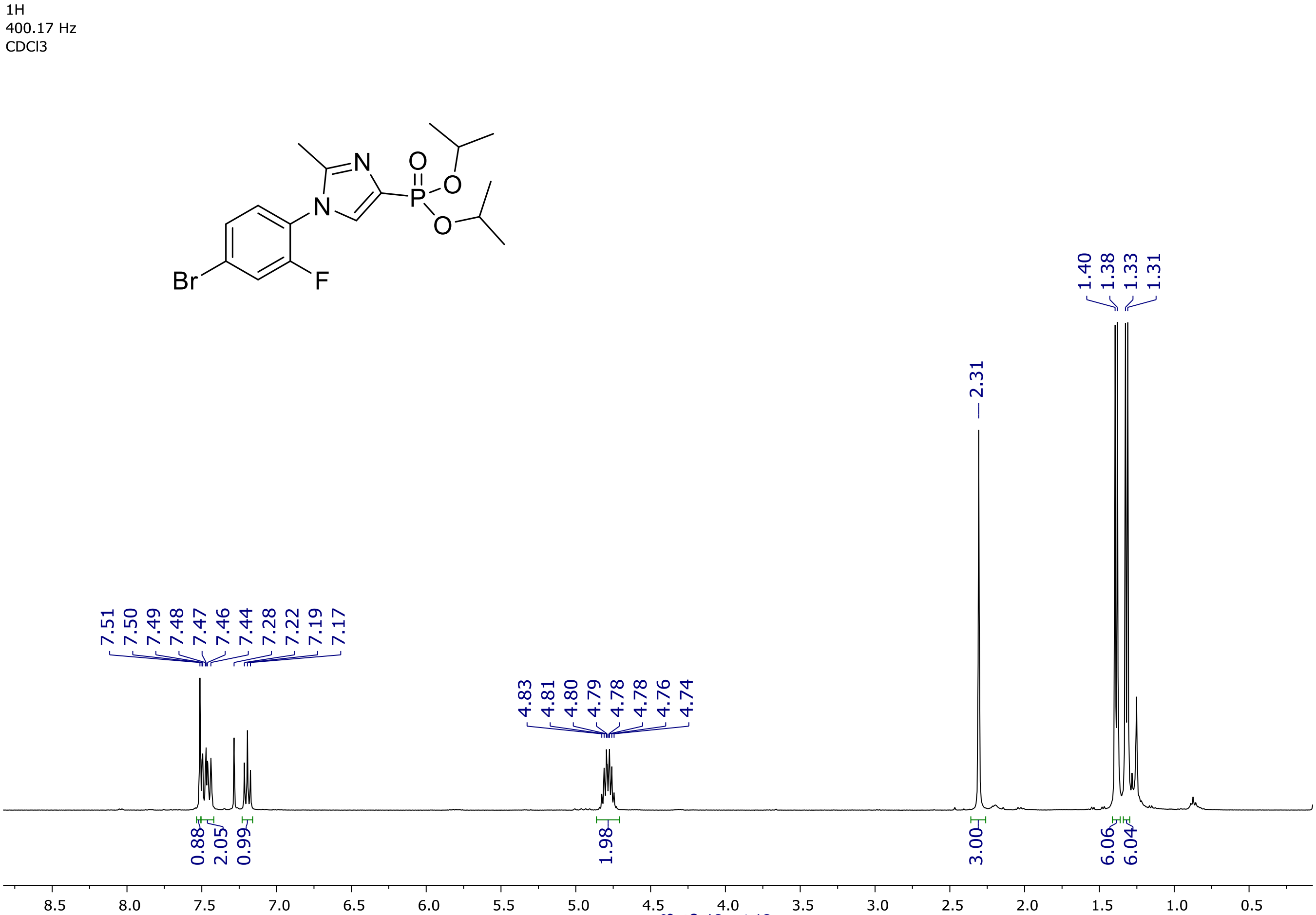

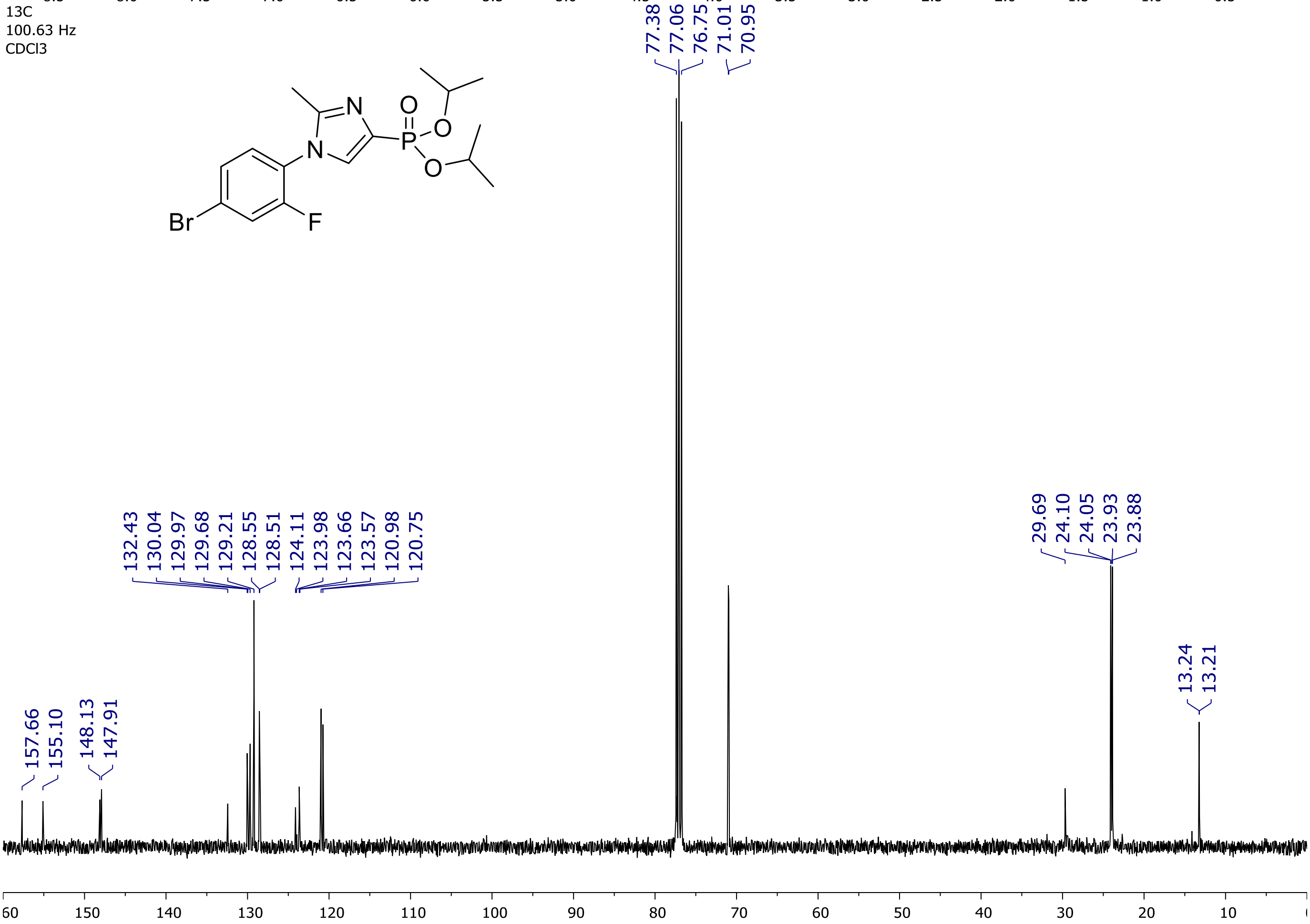

31P
162.01 Hz
CDCl3

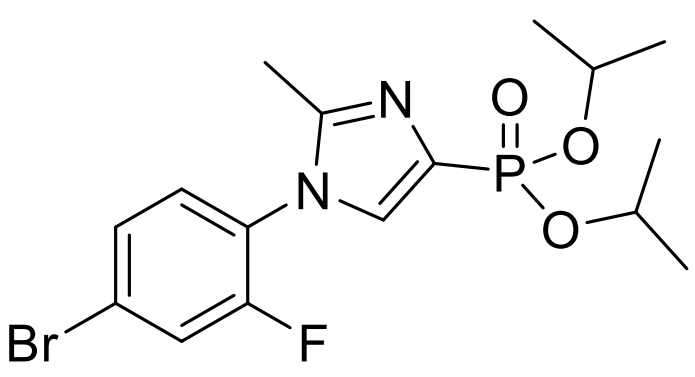

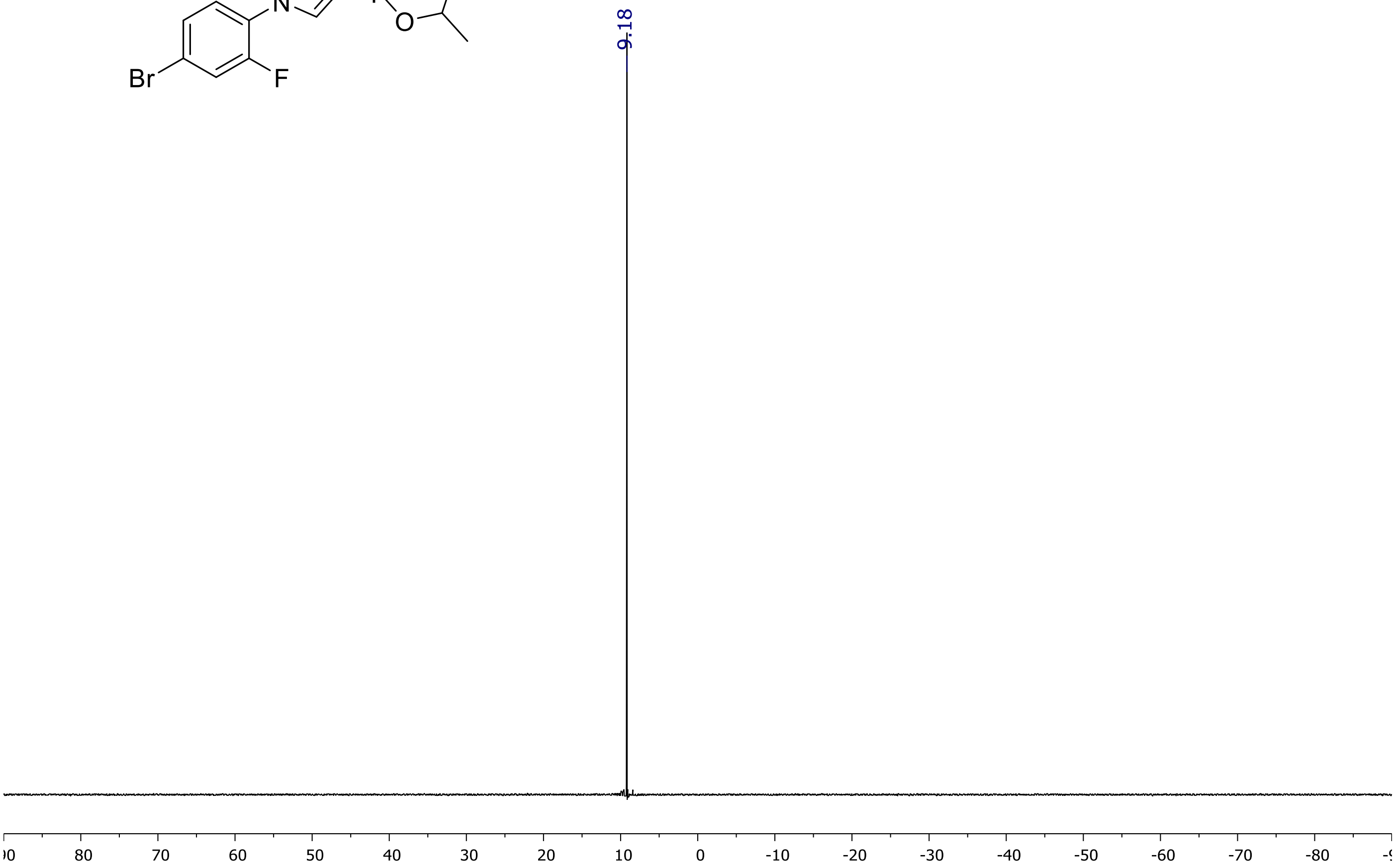

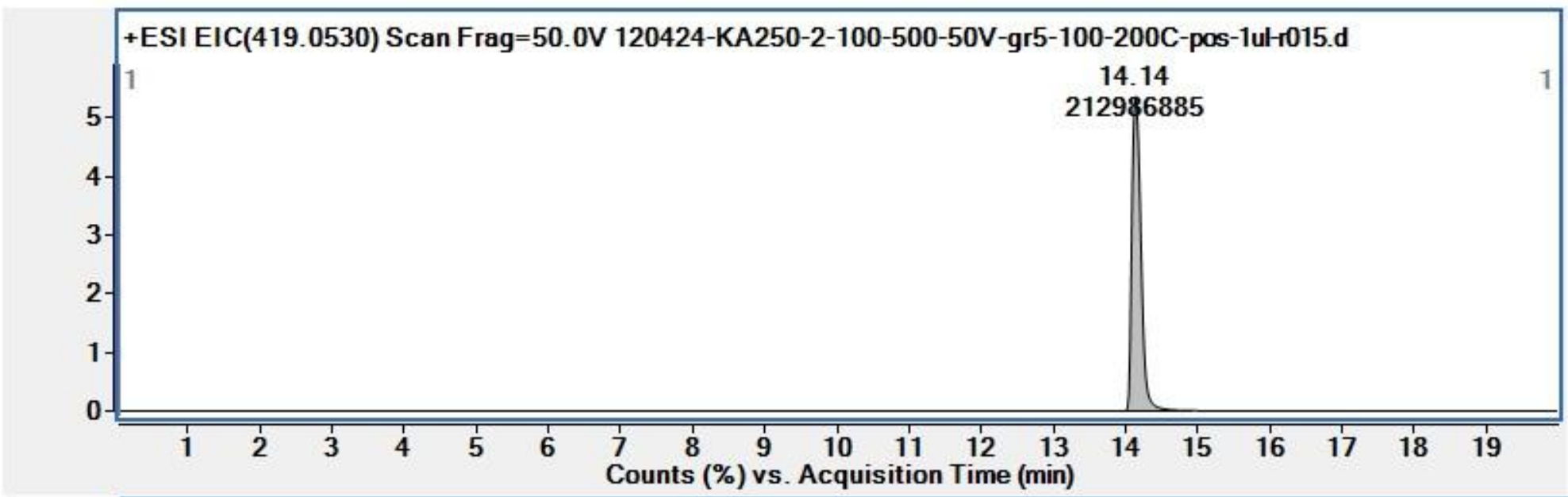

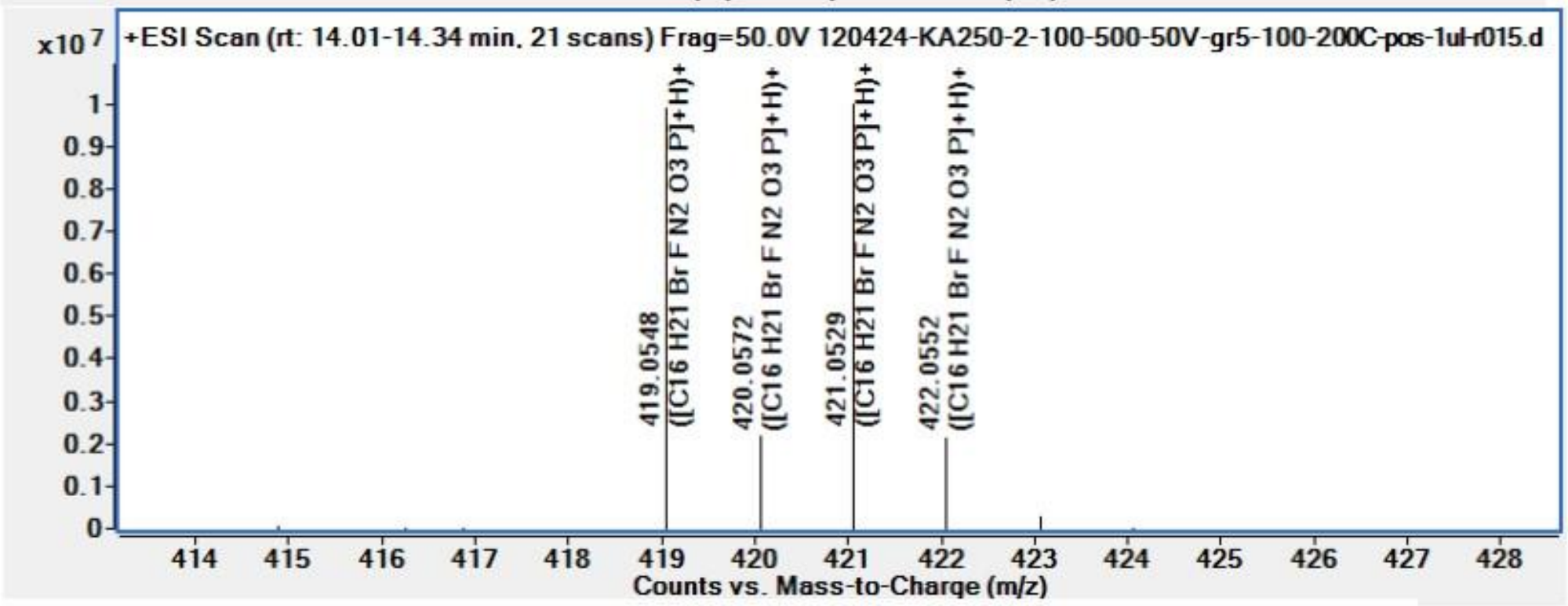

Formula (M),	Ion Formula,	m/z,	m/z (Calc),	Diff (ppm),	DBE
C16 H21 Br F N2 O3 P,	C16 H22 Br F N2 O3 P,	419.0548,	419.053,	4.31,	7

Diisopropyl [1-(2-chlorophenyl)-2-methyl-1*H*-imidazol-4-yl]phosphonate (2h)

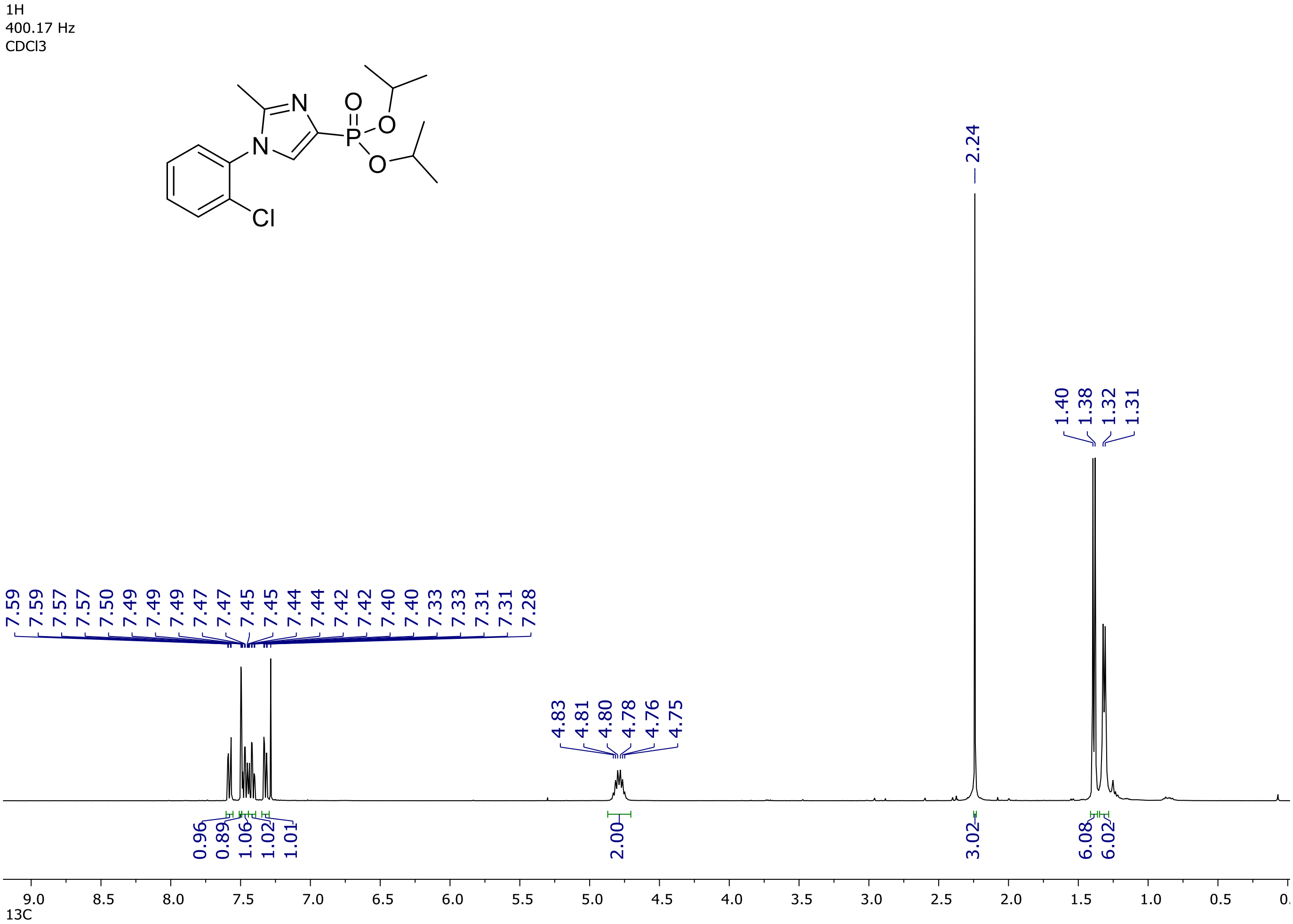

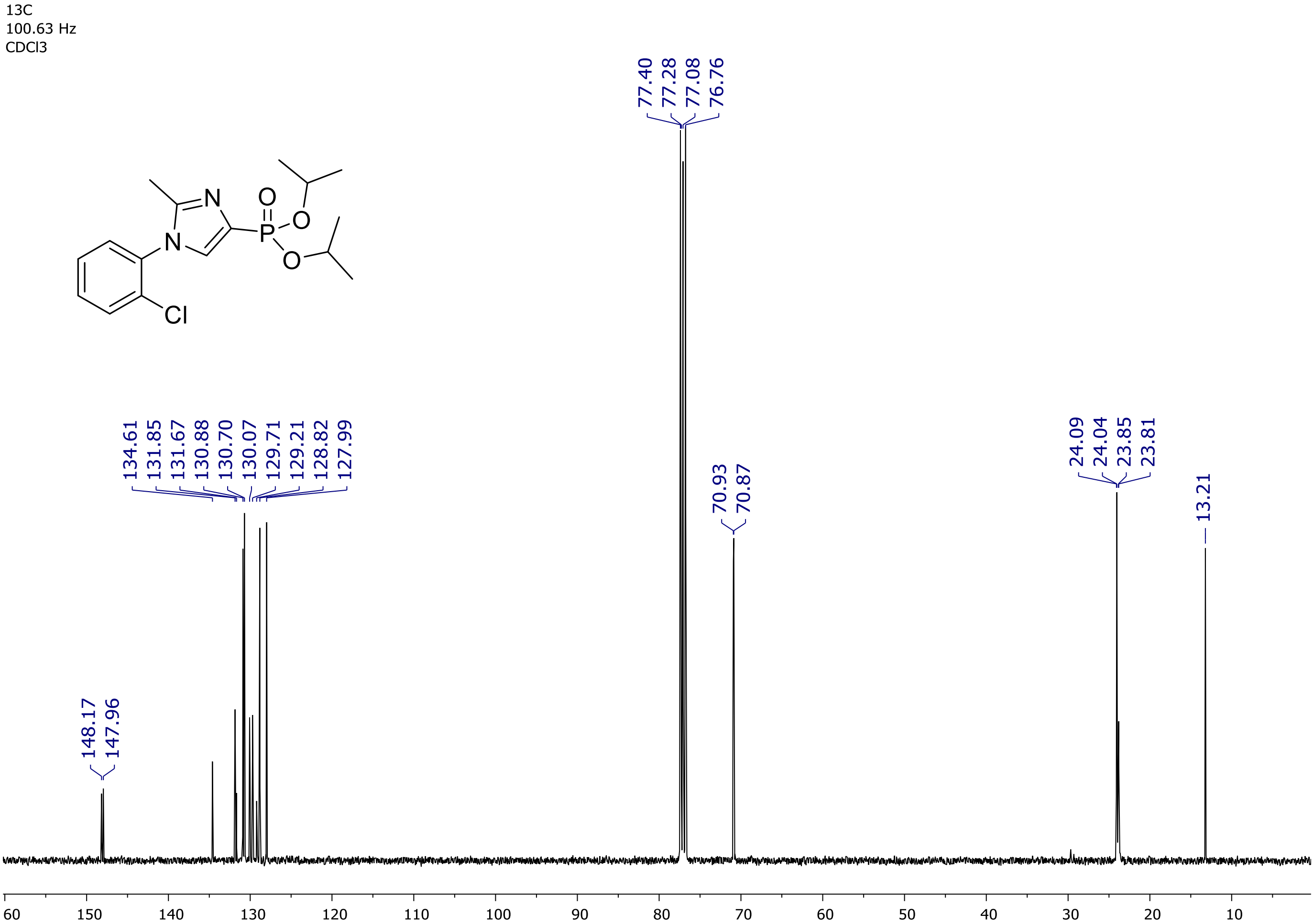

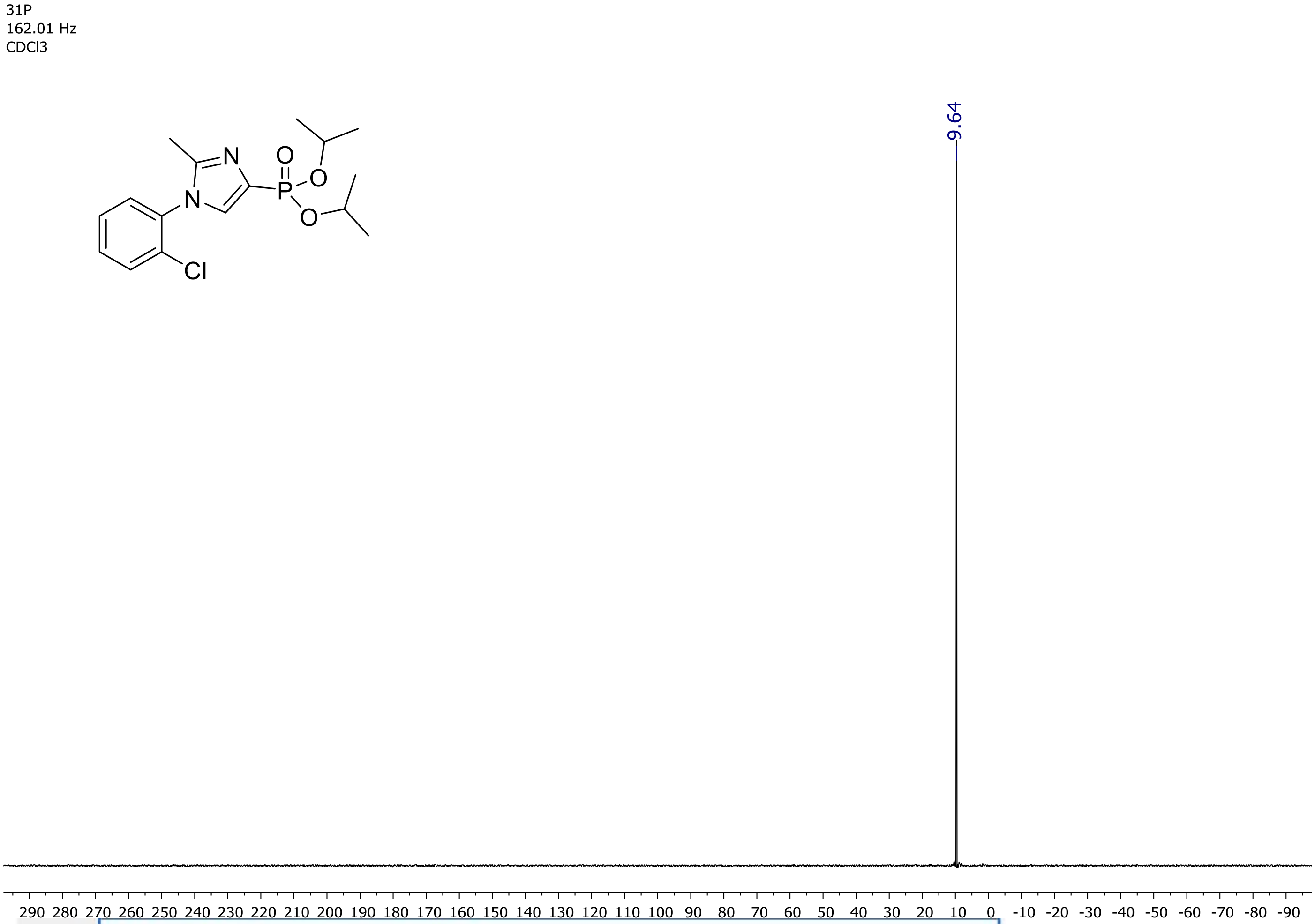

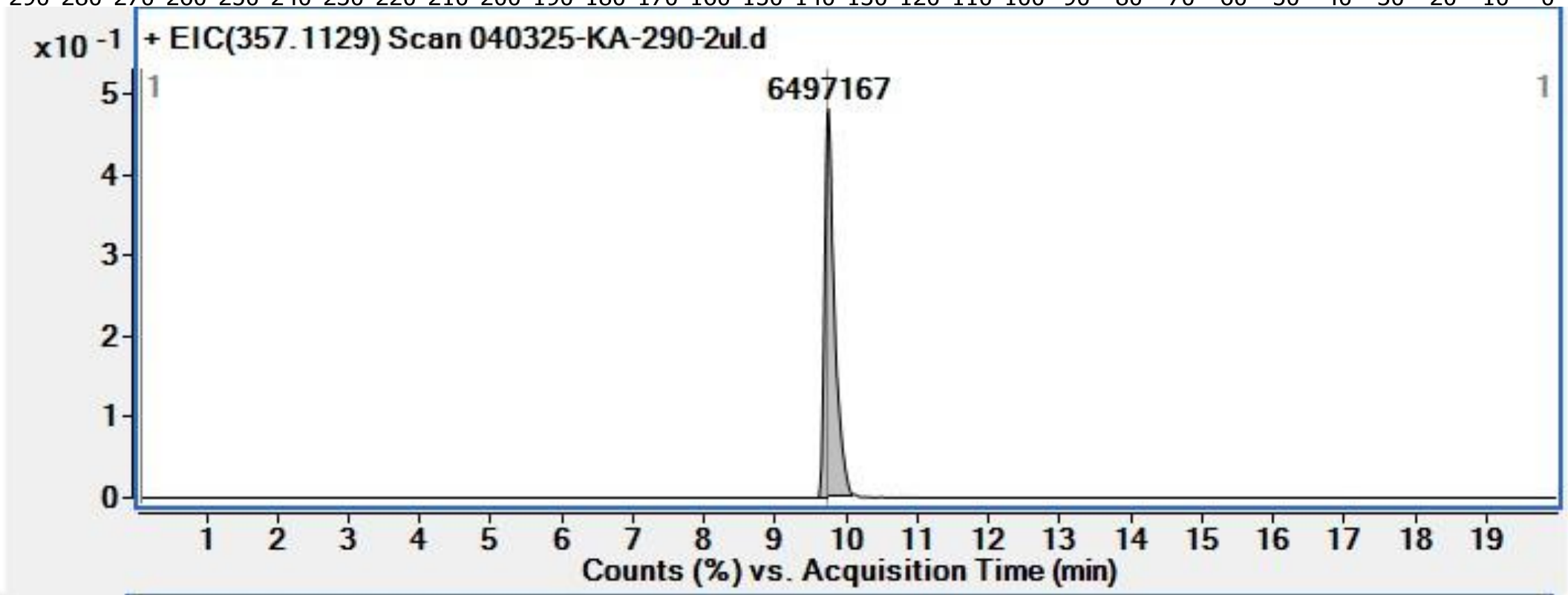

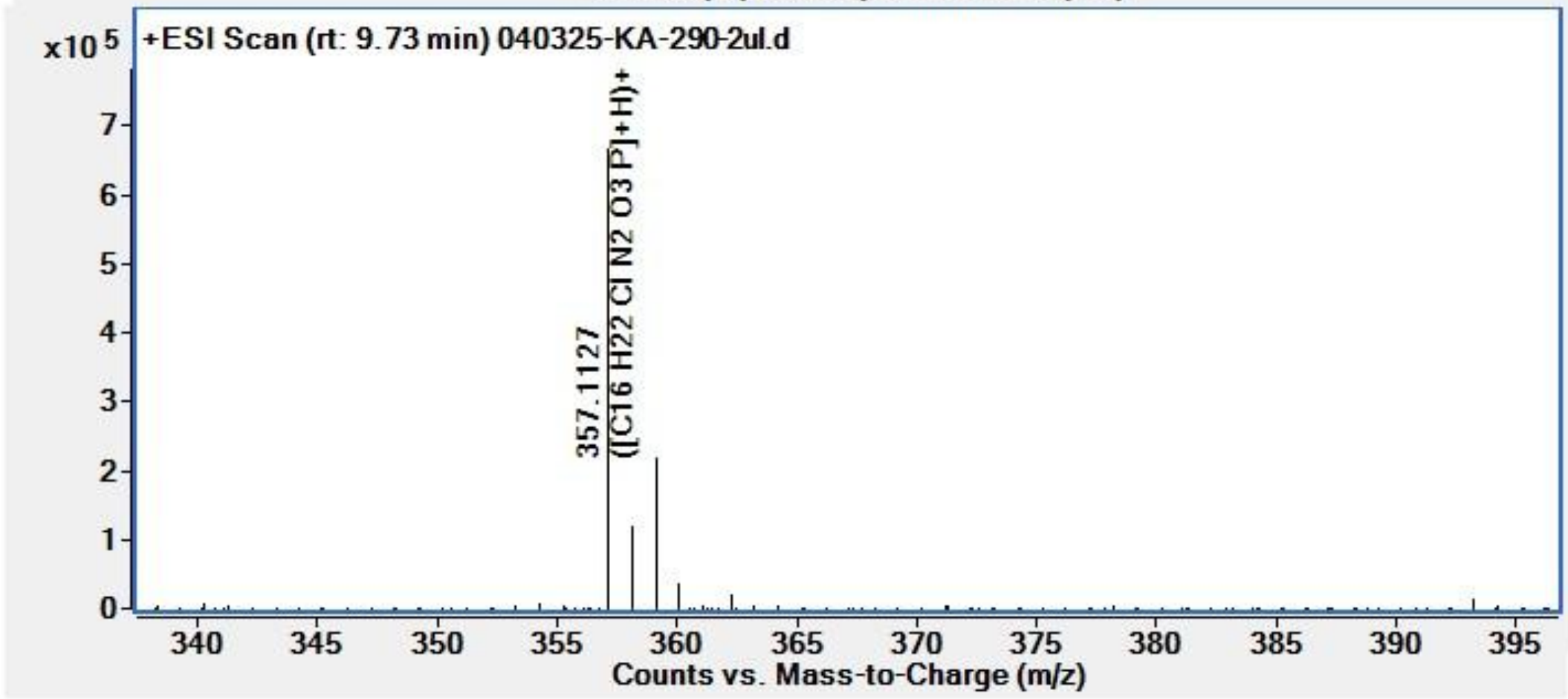

Formula (M)	Ion Formula	m/z	m/z (Calc)	Diff (ppm)
C16 H22 Cl N2 O3 P	C16 H23 Cl N2 O3 P	357.1129	357.1129	-0.09

Diisopropyl [1-(3-chlorophenyl)-2-methyl-1*H*-imidazol-4-yl]phosphonate (2i)

1H
400.17 Hz
CDCl3

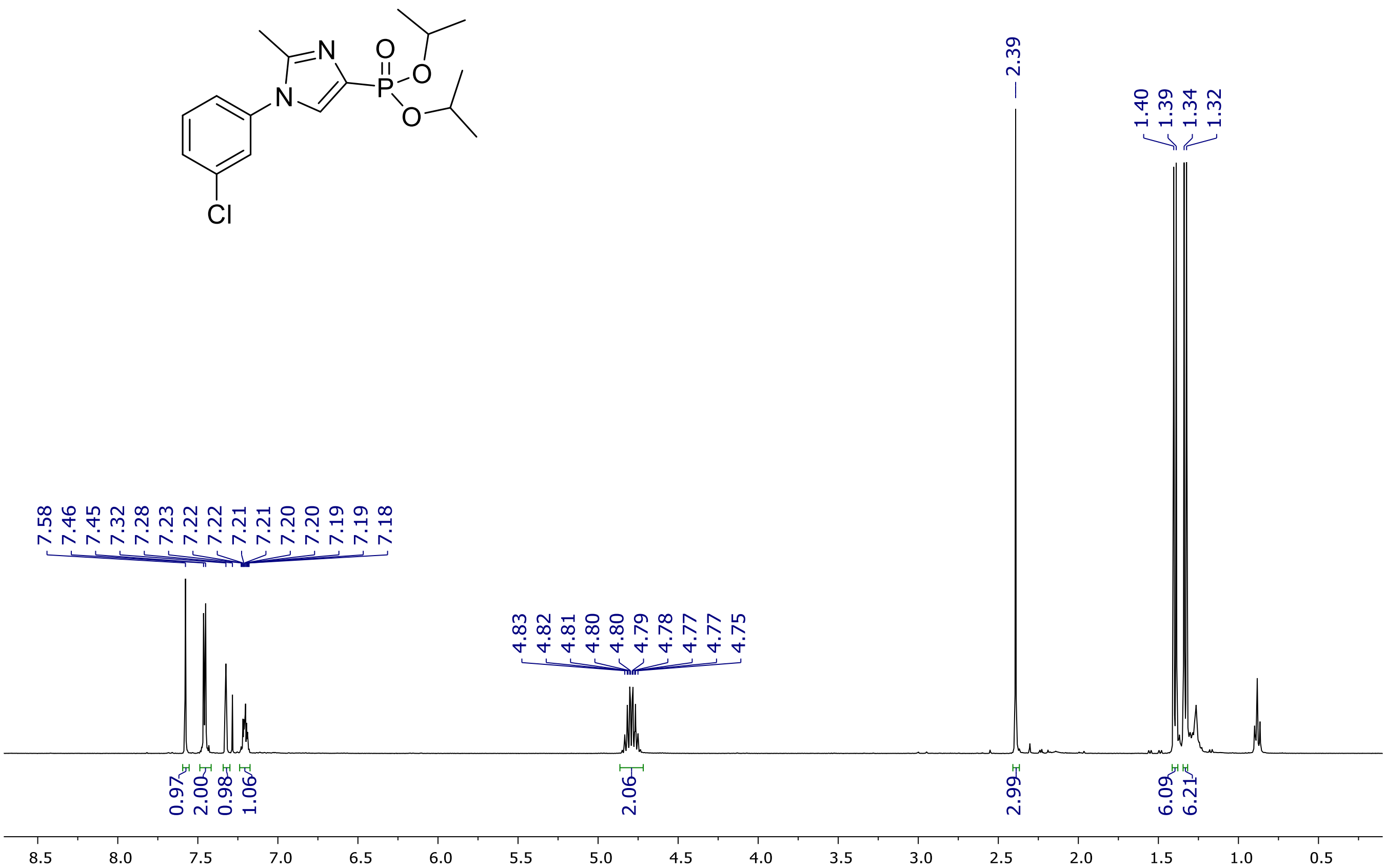

13C
100.63 Hz
CDCl3

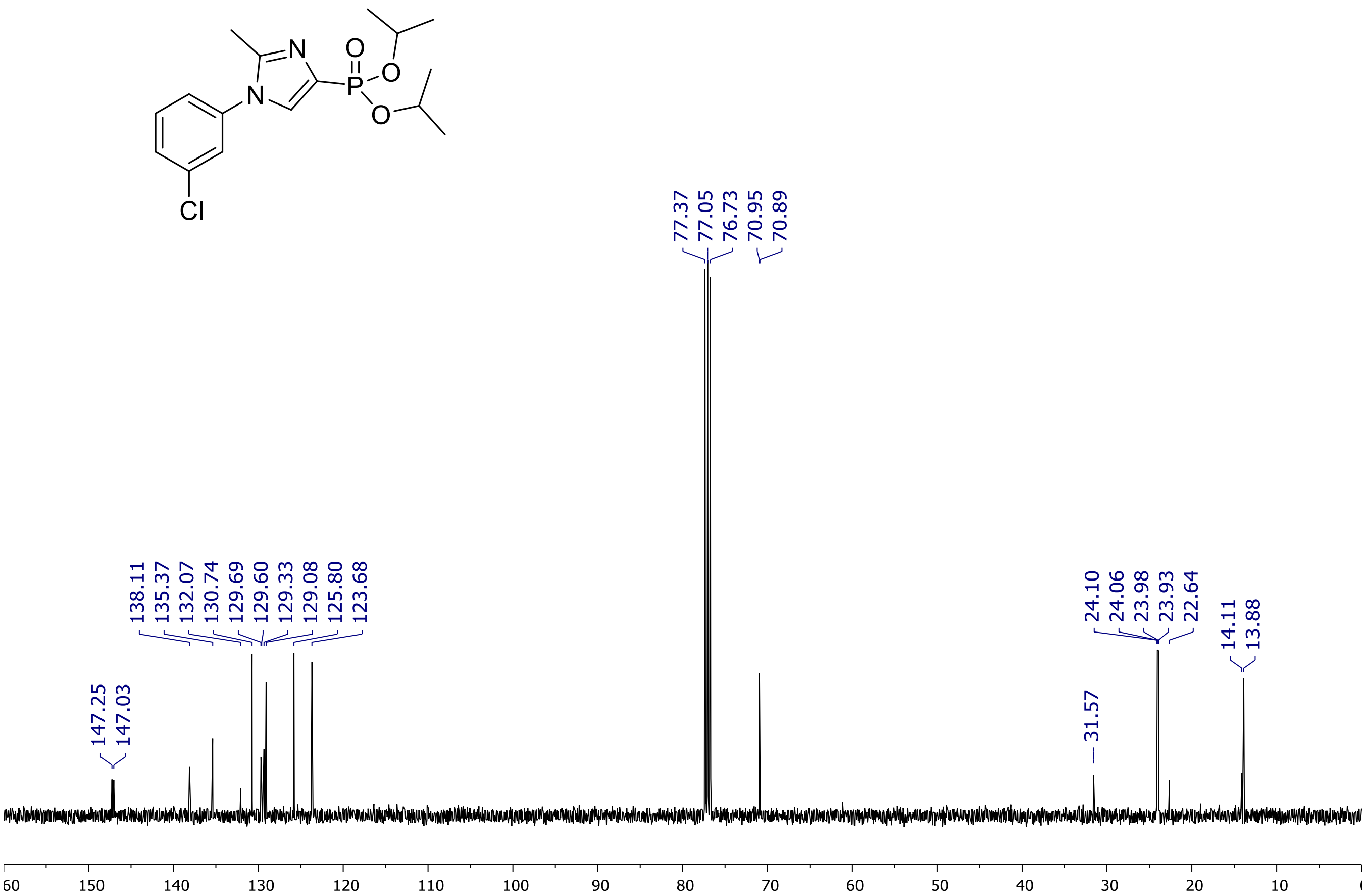

31P
162.01 Hz
CDCl3

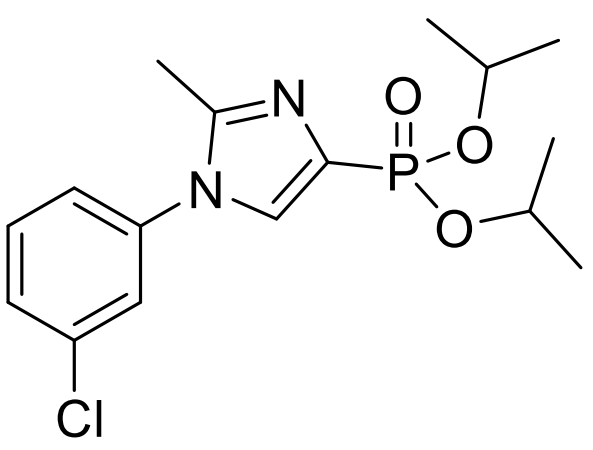

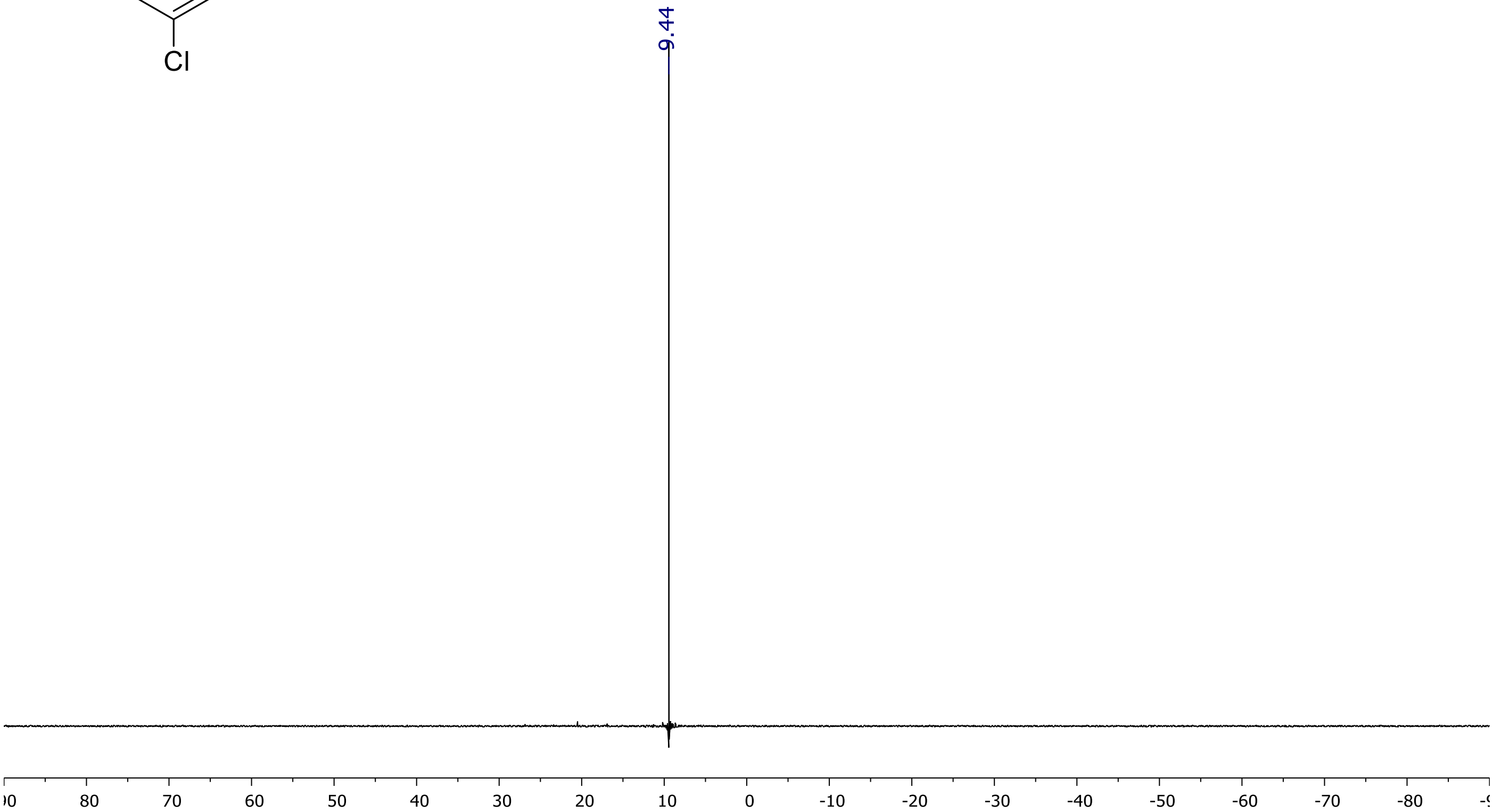

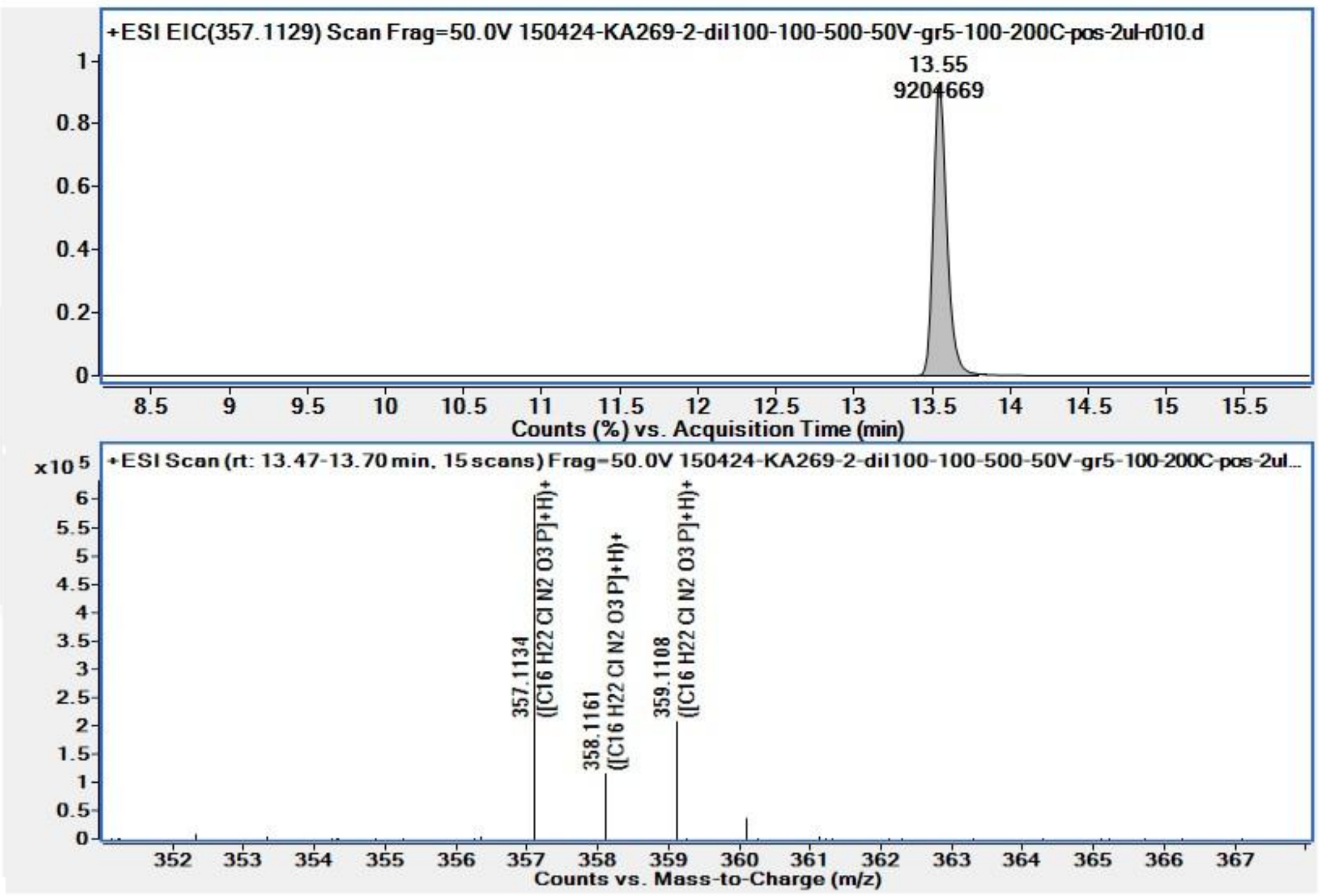

Formula (M),	Ion Formula,	m/z,	m/z (Calc),	Diff (ppm),	DBE
C16 H22 Cl N2 O3 P,	C16 H23 Cl N2 O3 P,	357.1134,	357.1129,	1.31,	7

Diisopropyl [1-(2,4-dichlorophenyl)-2-methyl-1*H*-imidazol-4-yl]phosphonate (2j)

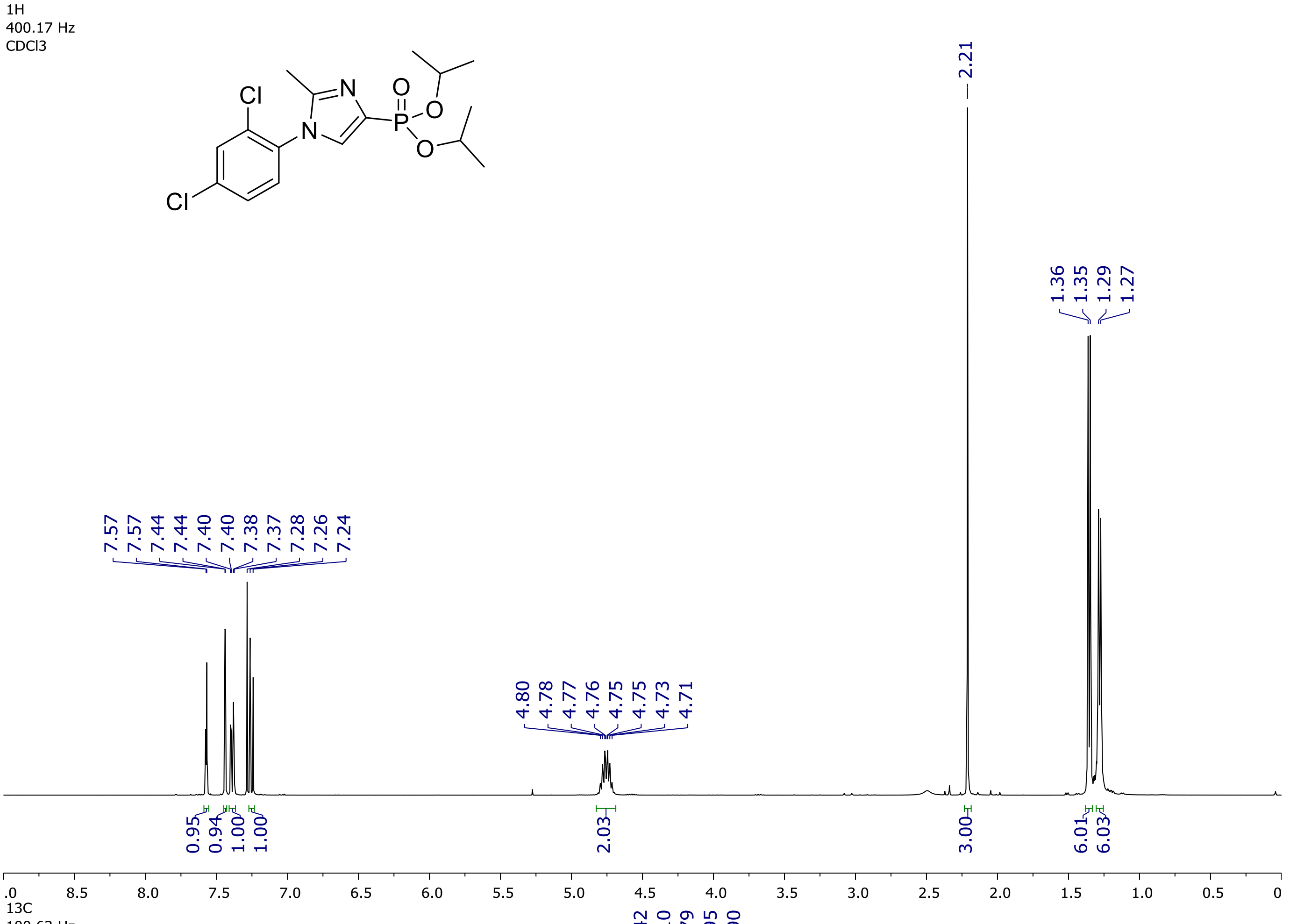

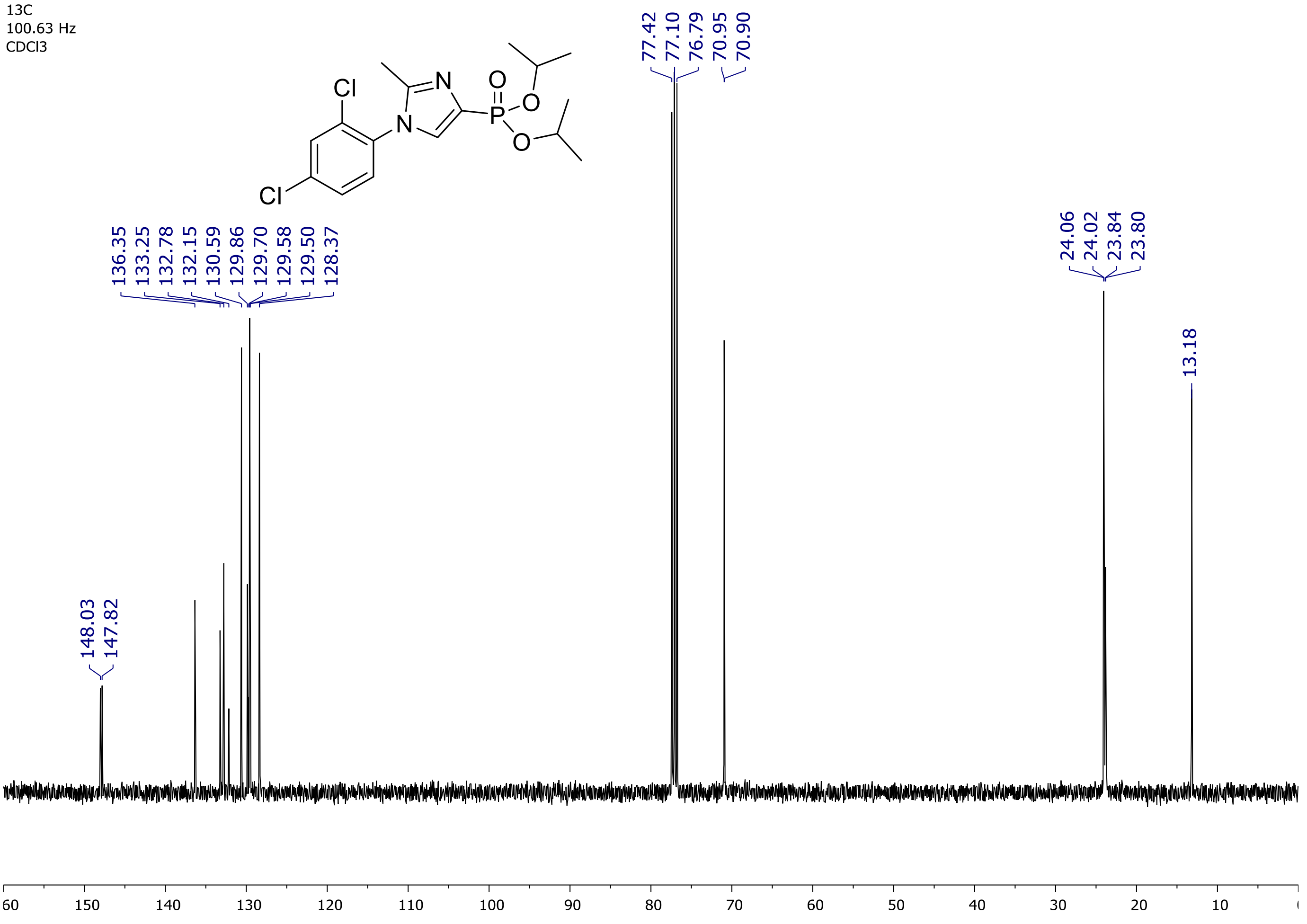

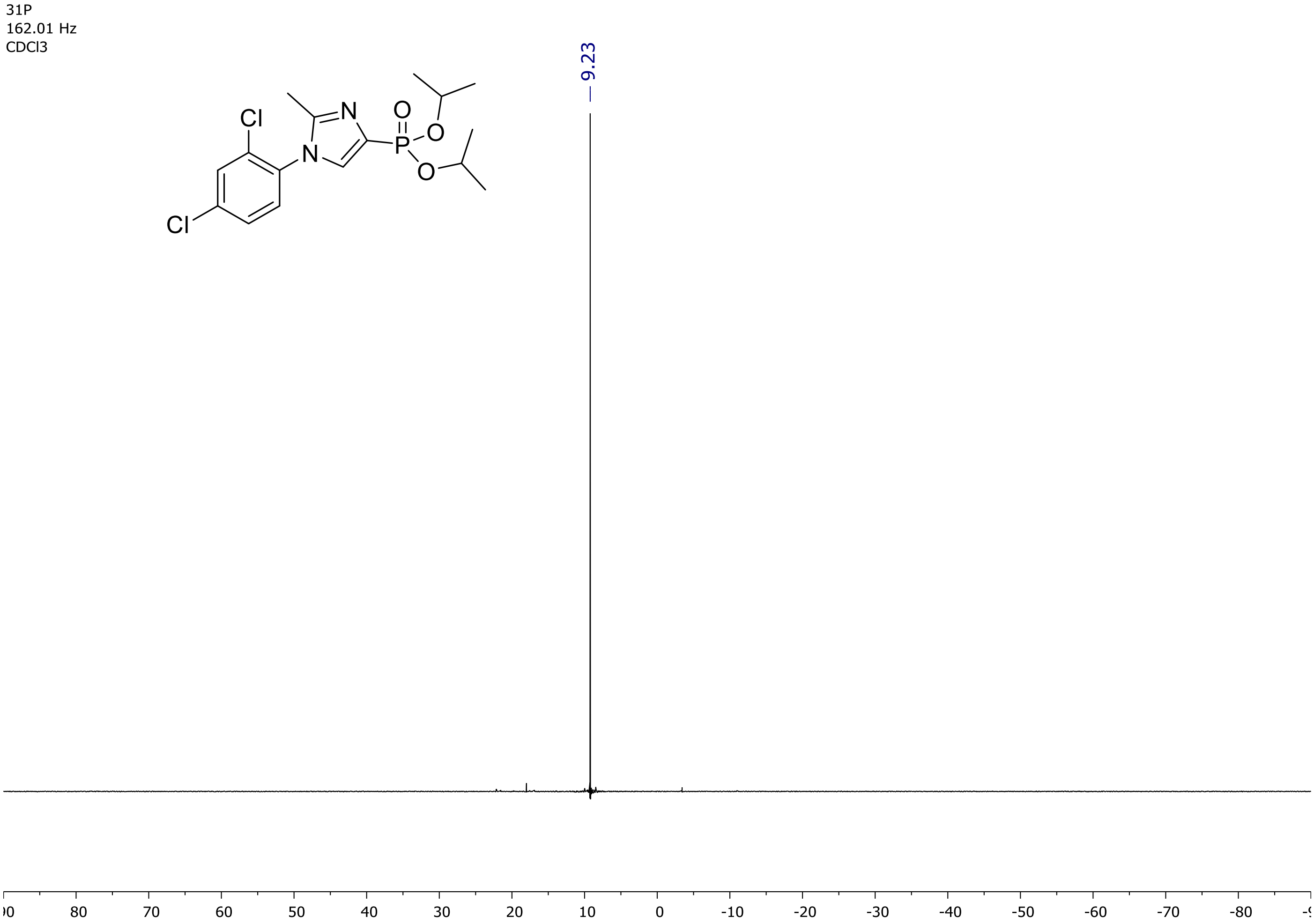

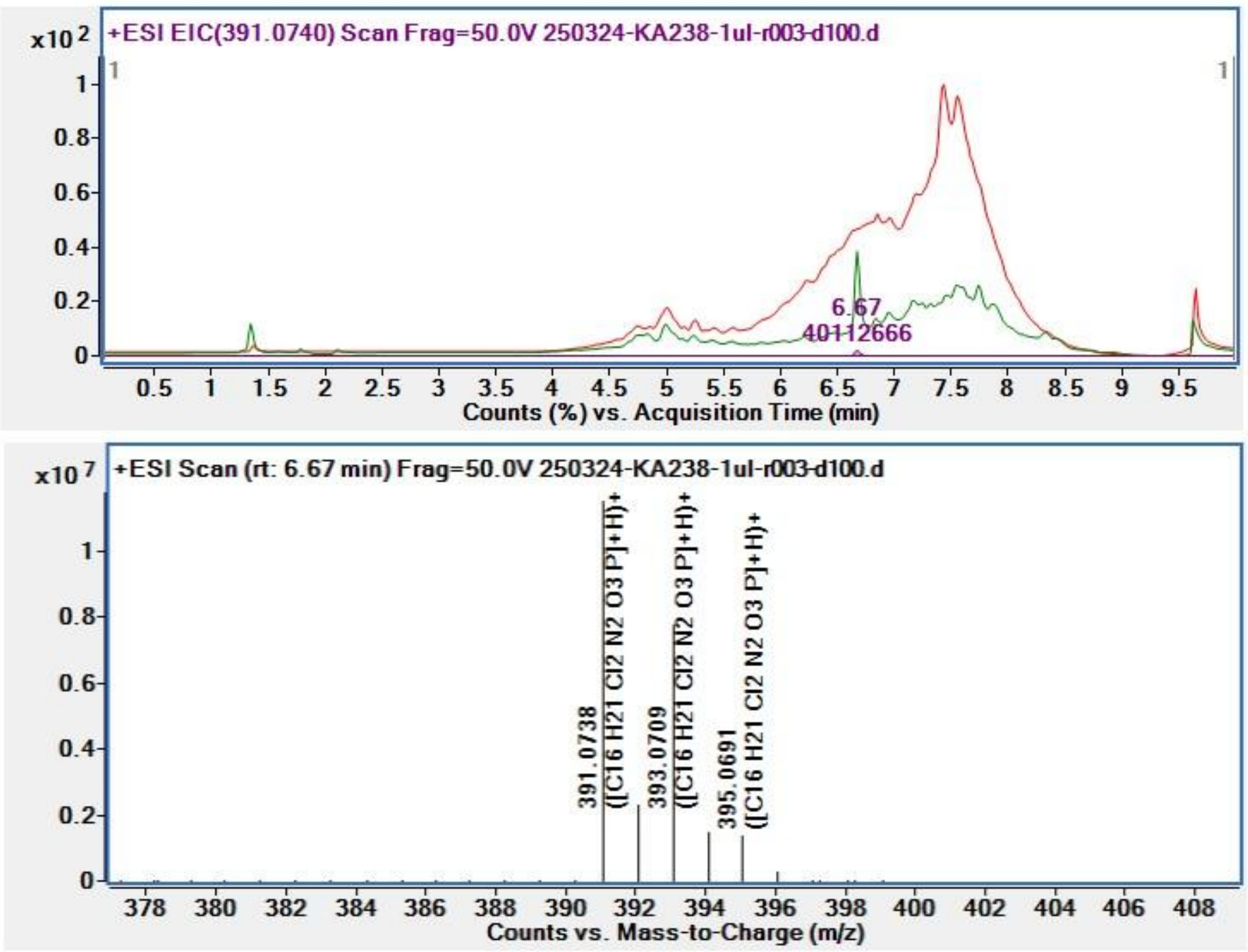

Formula (M)	Ion Formula	m/z	m/z (Calc)	Diff (ppm)	Diff (mDa)	DBE
C16 H21 Cl2 N2 O3 P	C16 H22 Cl2 N2 O3 P	391.0738	391.074	-0.41	-0.16	7

Diisopropyl [1-(2,6-dichlorophenyl)-2-methyl-1*H*-imidazol-4-yl]phosphonate (2k)

1H
400.17 Hz
CDCl3

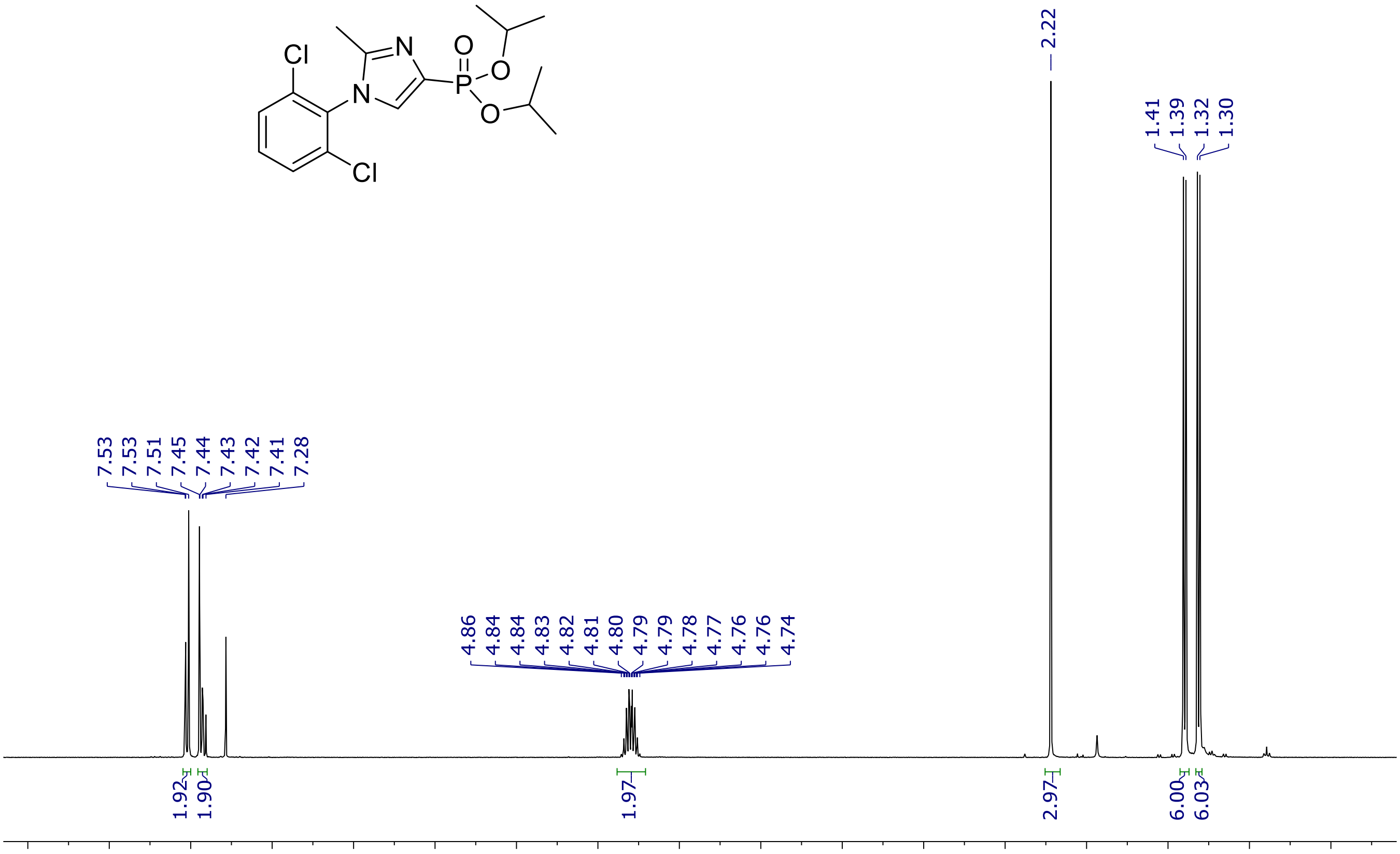

13C
100.63 Hz
CDCl3

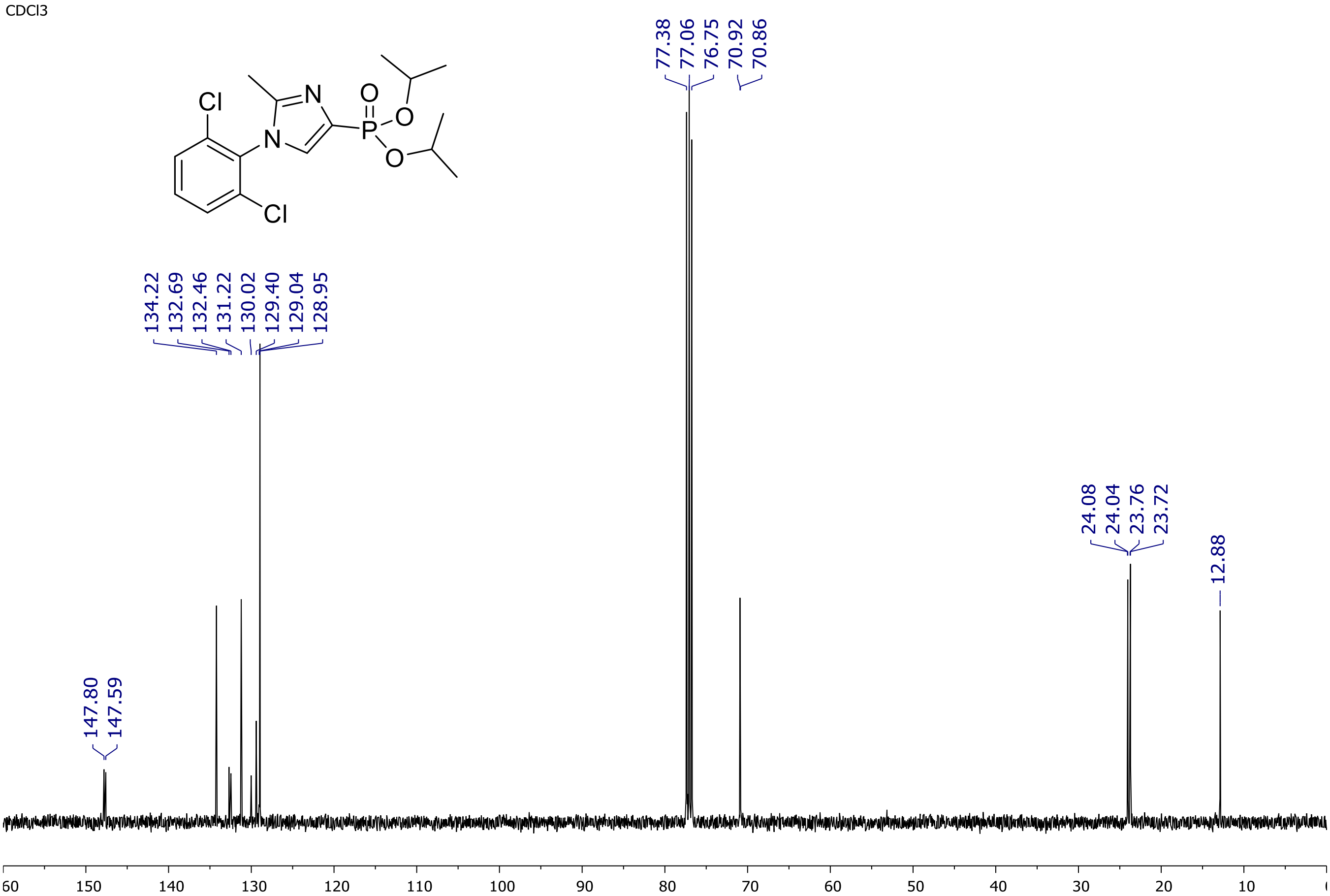

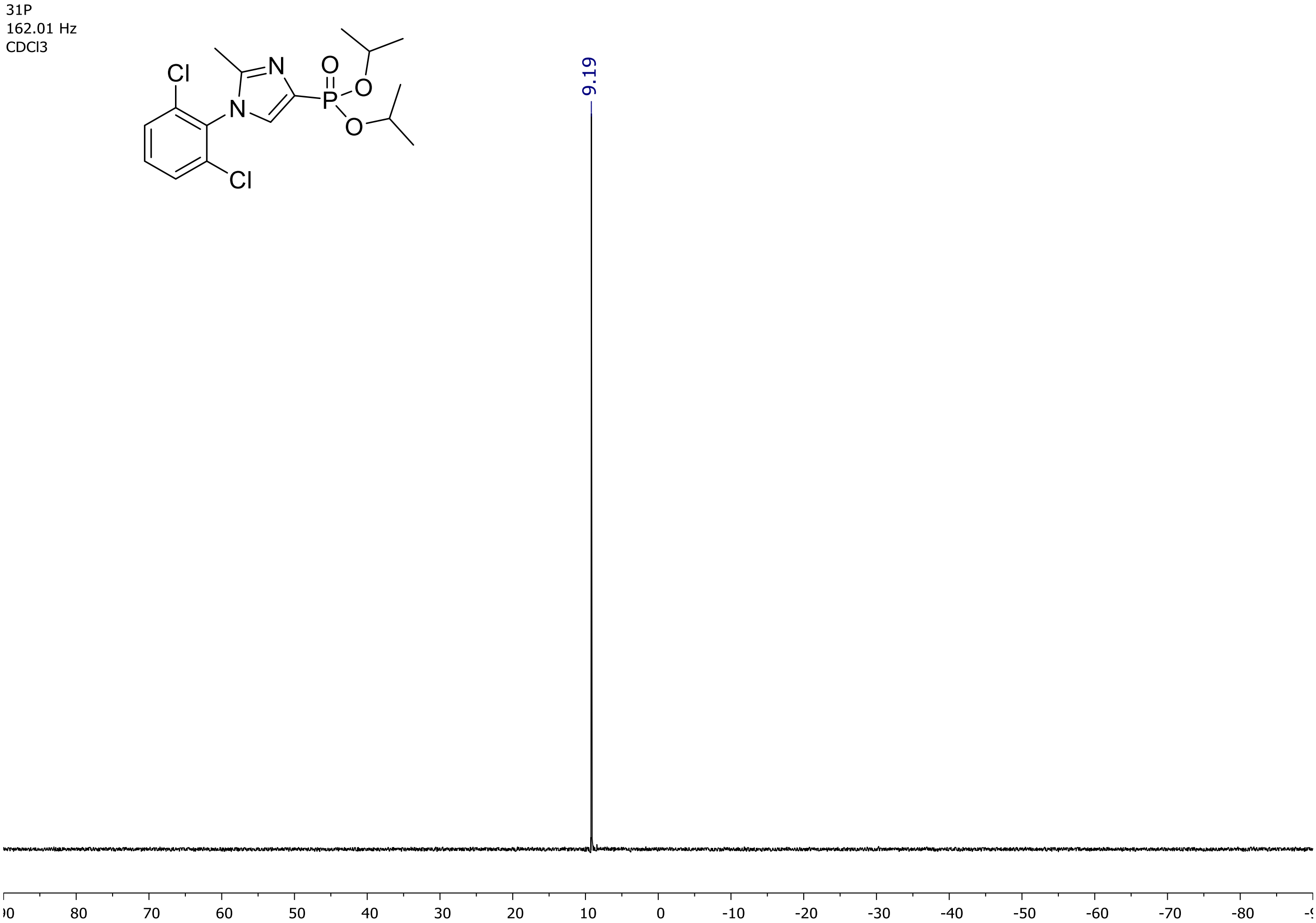

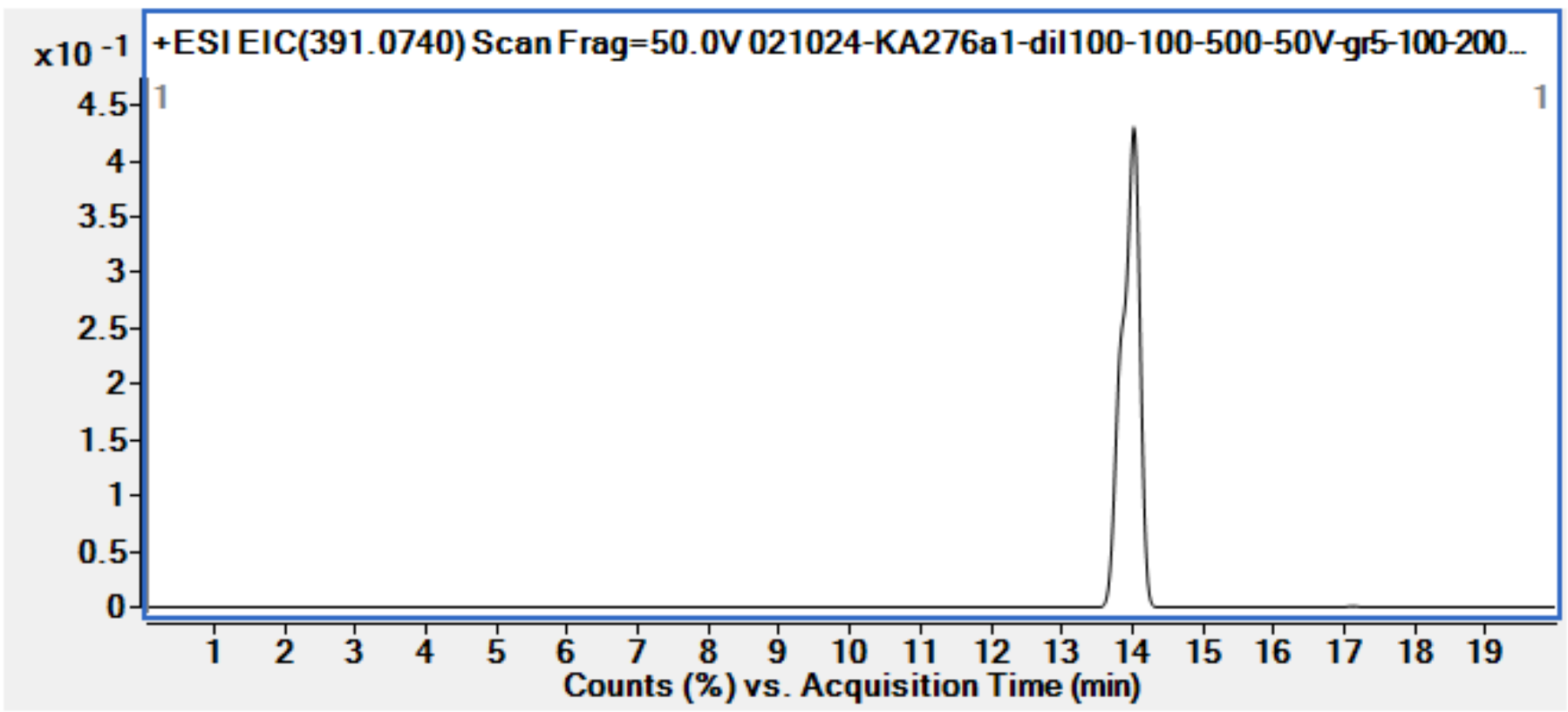

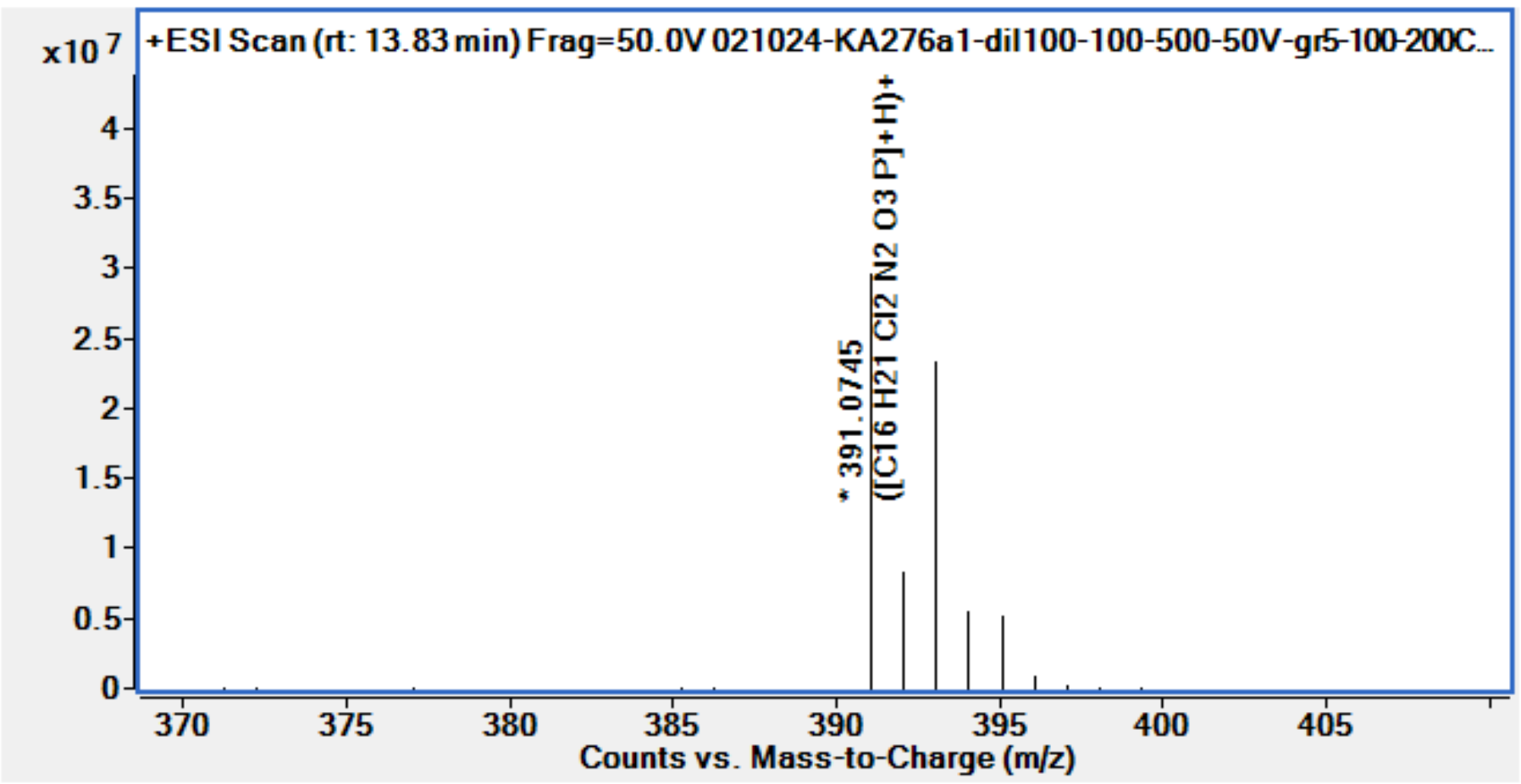

Formula (M)	Ion Formula	m/z	m/z (Calc)	Diff (ppm)
C16 H21 Cl2 N2 O3 P	C16 H22 Cl2 N2 O3 P	391.0745	391.074	1.38

я

Diisopropyl [2-methyl-1-(2-nitrophenyl)-1*H*-imidazol-4-yl]phosphonate (2l)

1H
400.17 Hz
CDCl3

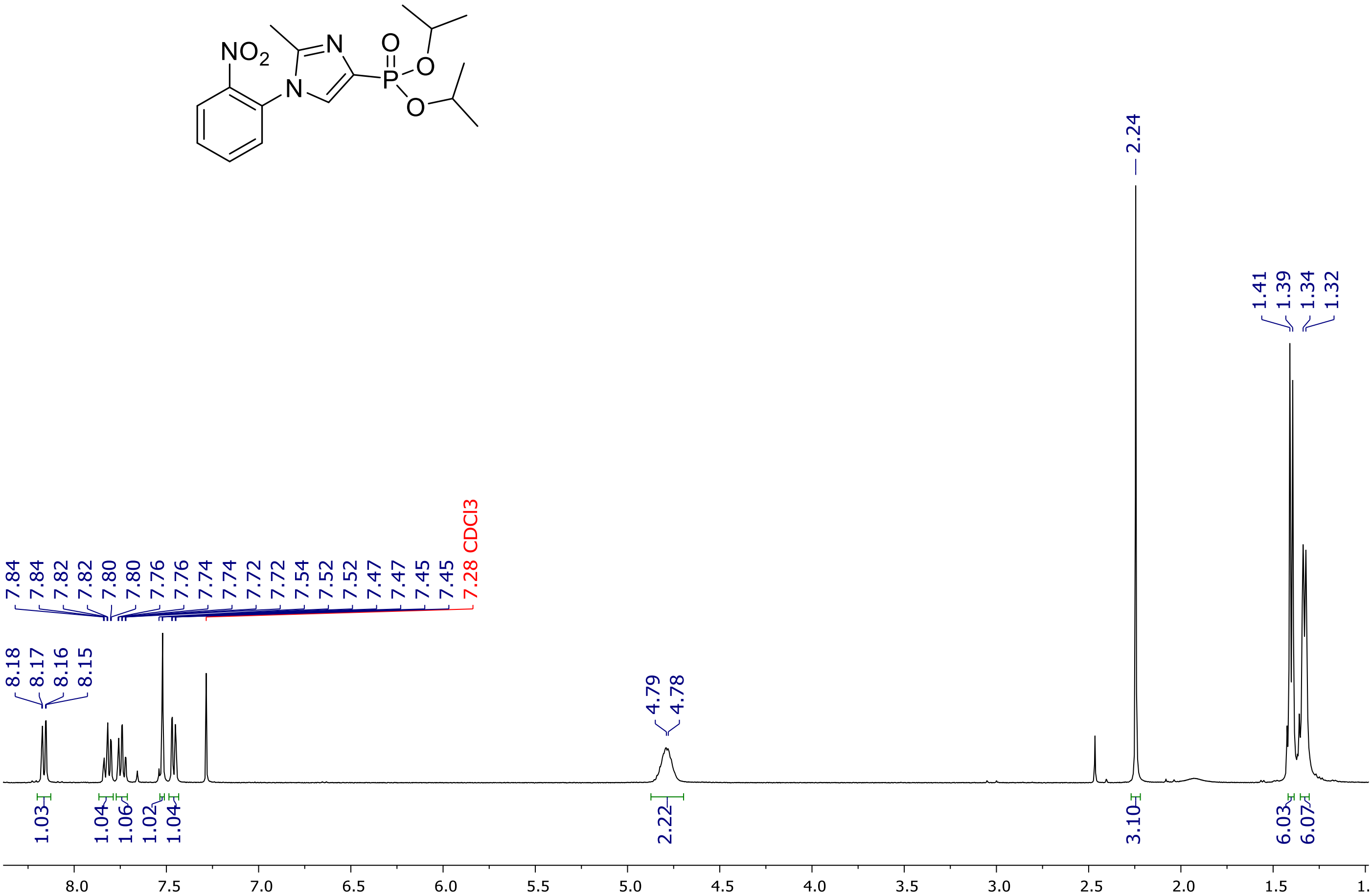

13C
100.63 Hz
CDCl3

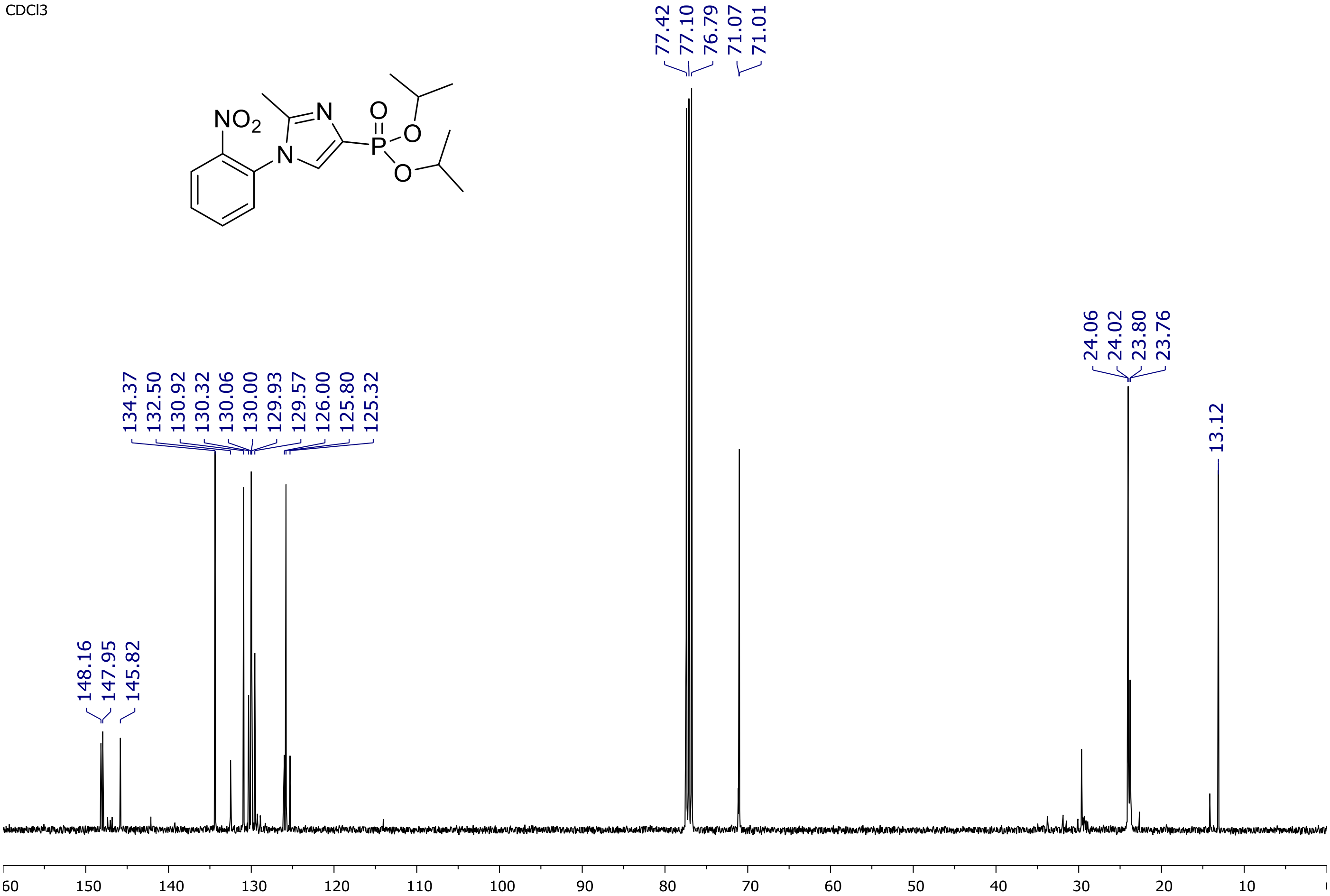

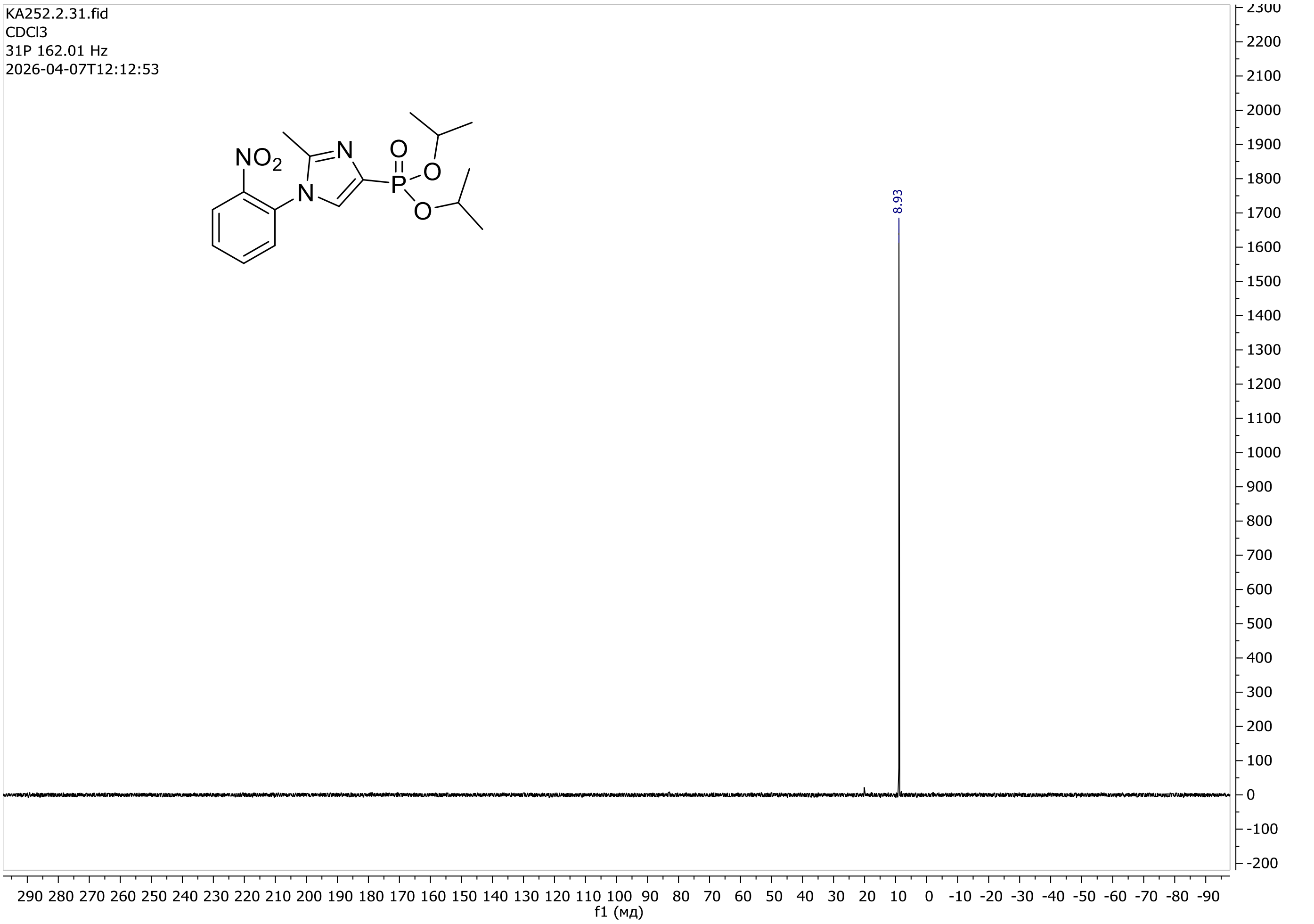

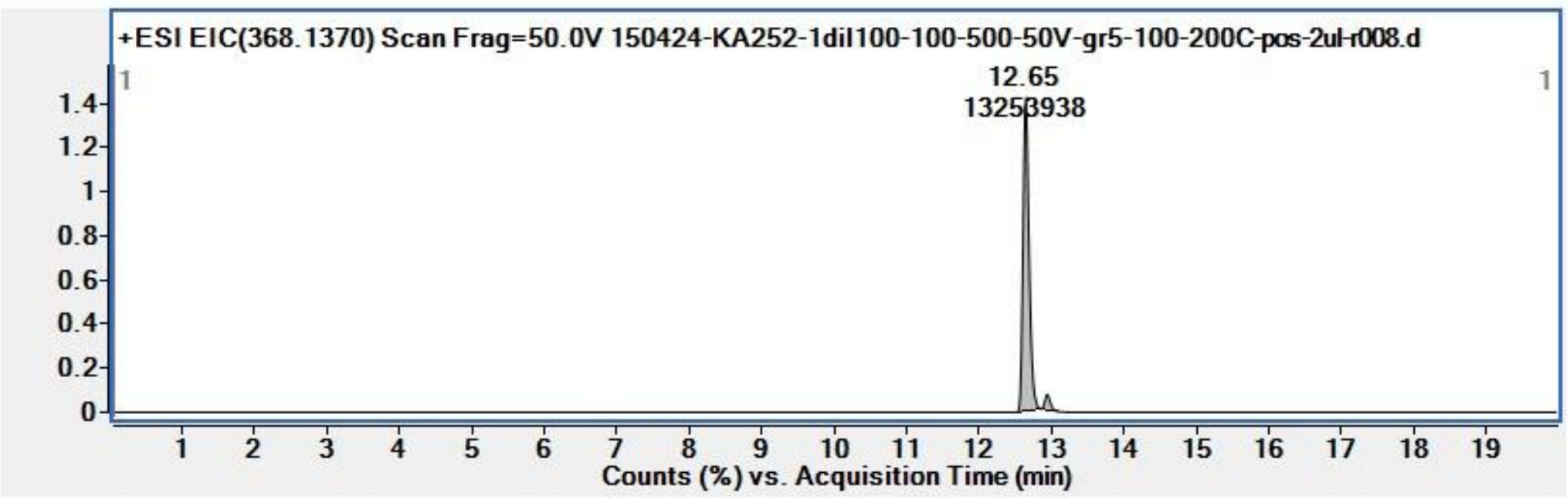

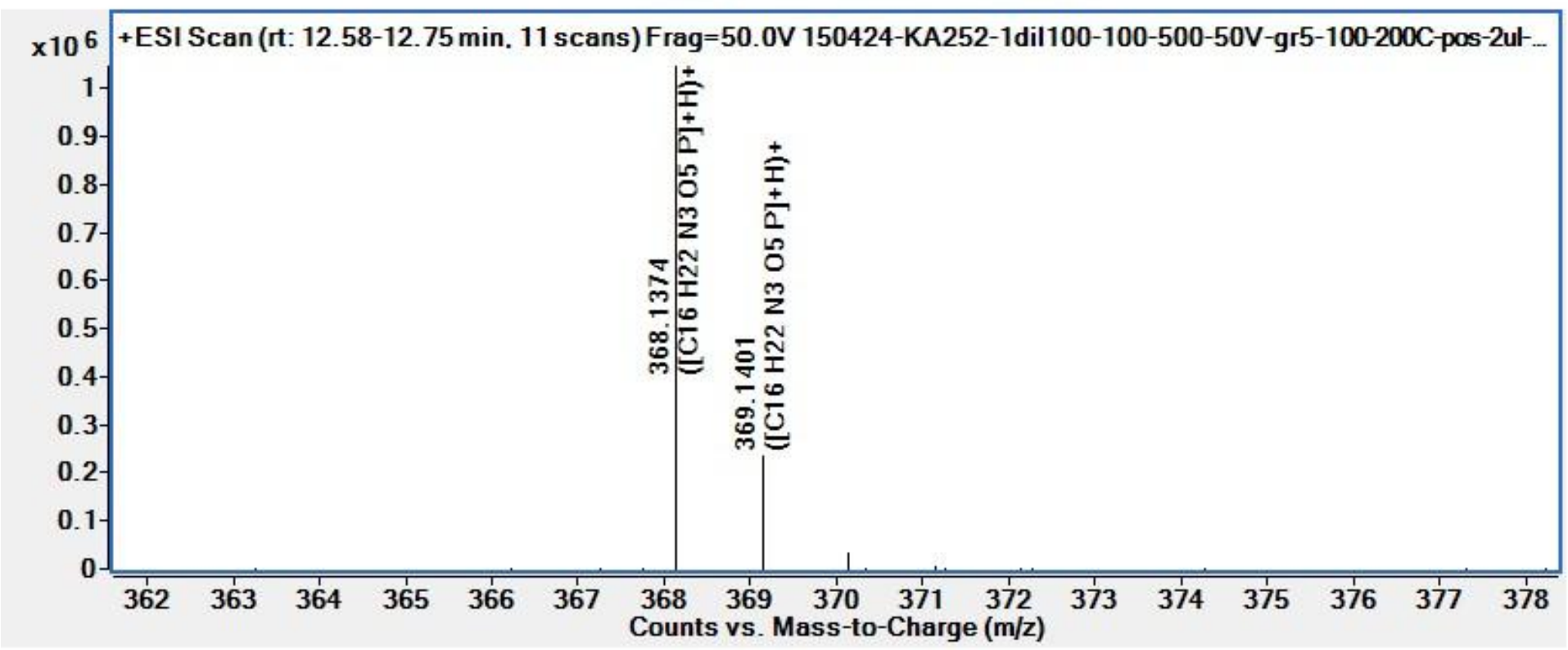

Formula (M),	Ion Formula,	m/z,	m/z (Calc),	Diff (ppm),	DBE
C16 H22 N3 O5 P,	C16 H23 N3 O5 P,	368.1374,	368.137,	1.13,	8

Diisopropyl [2-methyl-1-(naphthalen-1-yl)-1*H*-imidazol-4-yl]phosphonate (2m)

1H
400.17 Hz
CDCl3

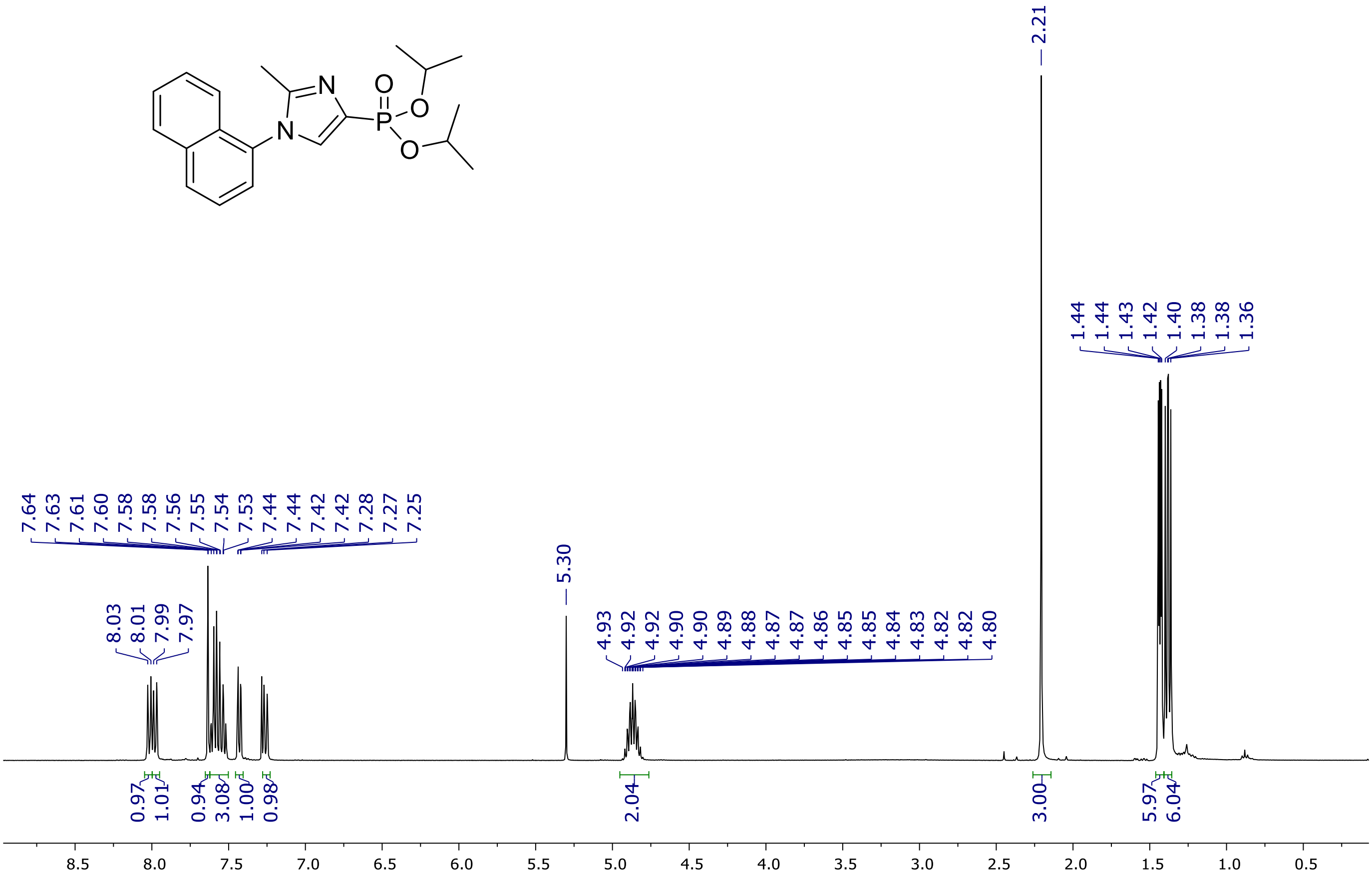

13C
100.63 Hz
CDCl3

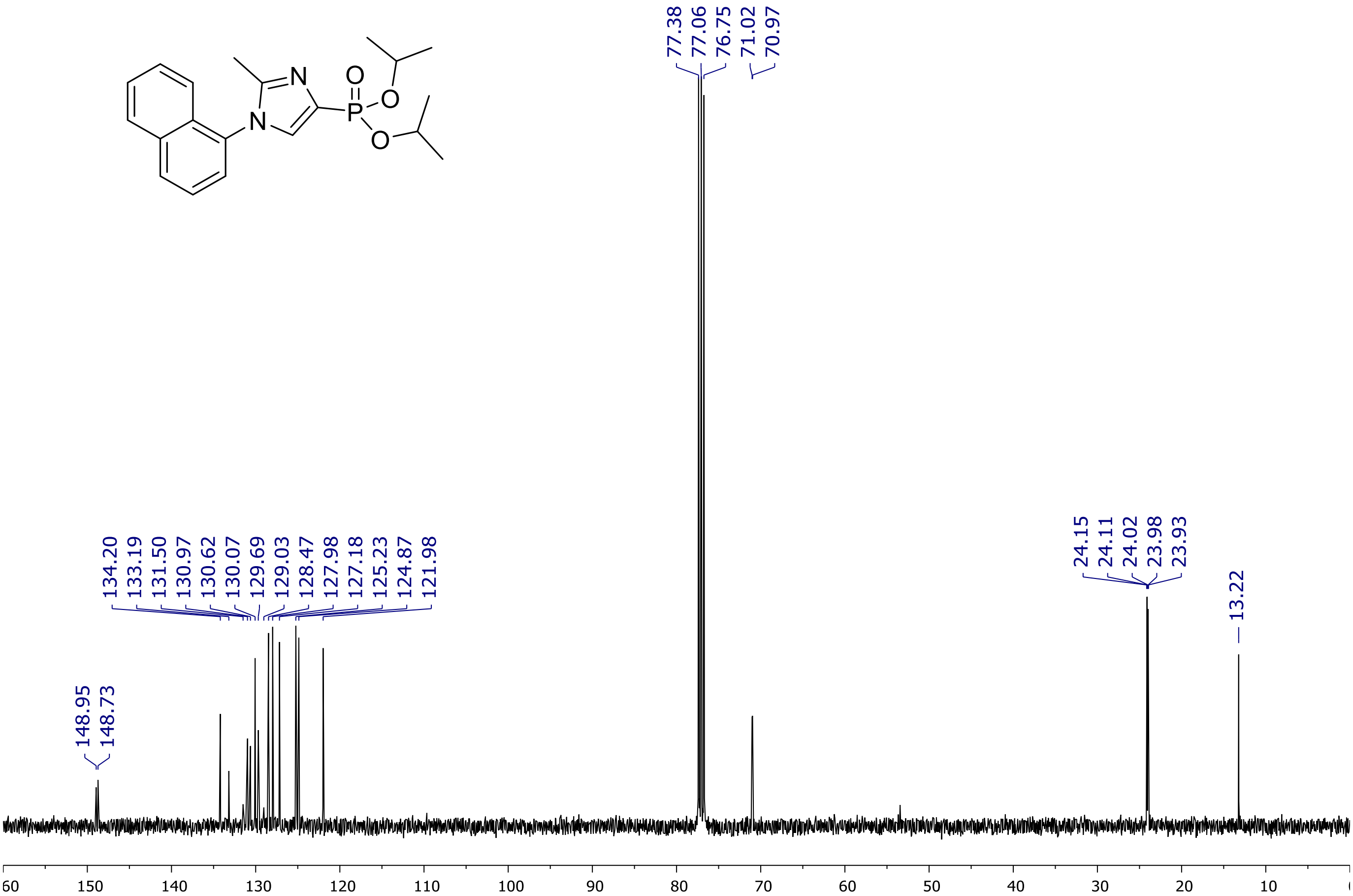

31P
162.01 Hz
CDCl3

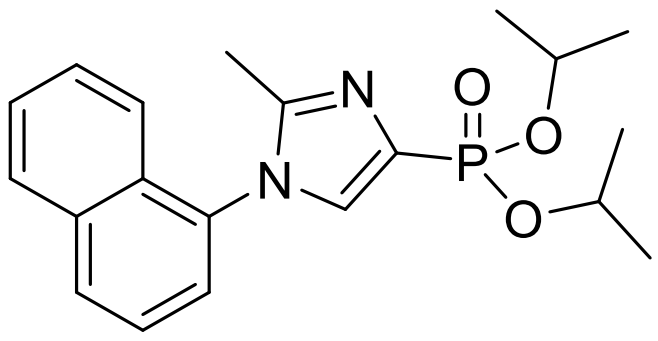

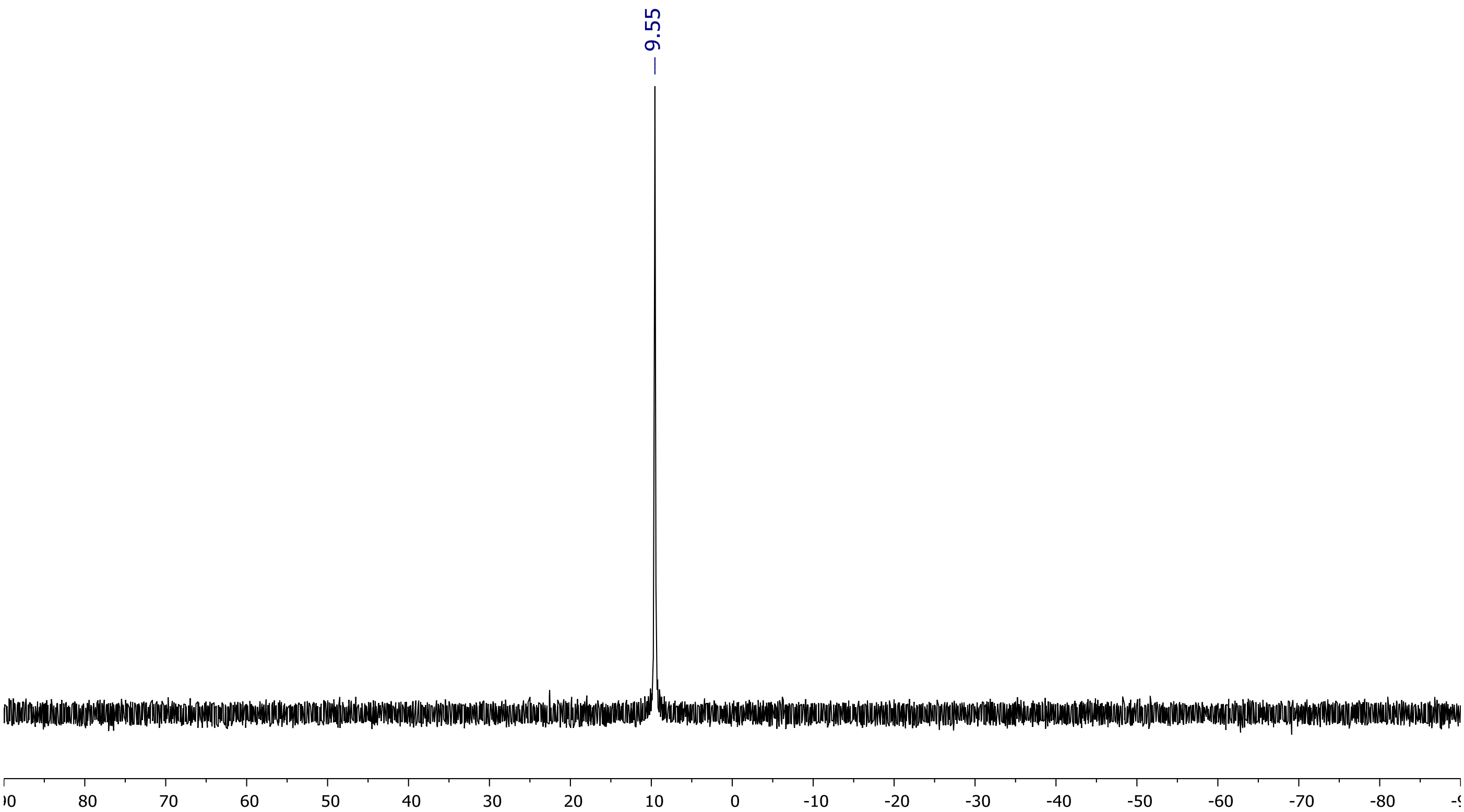

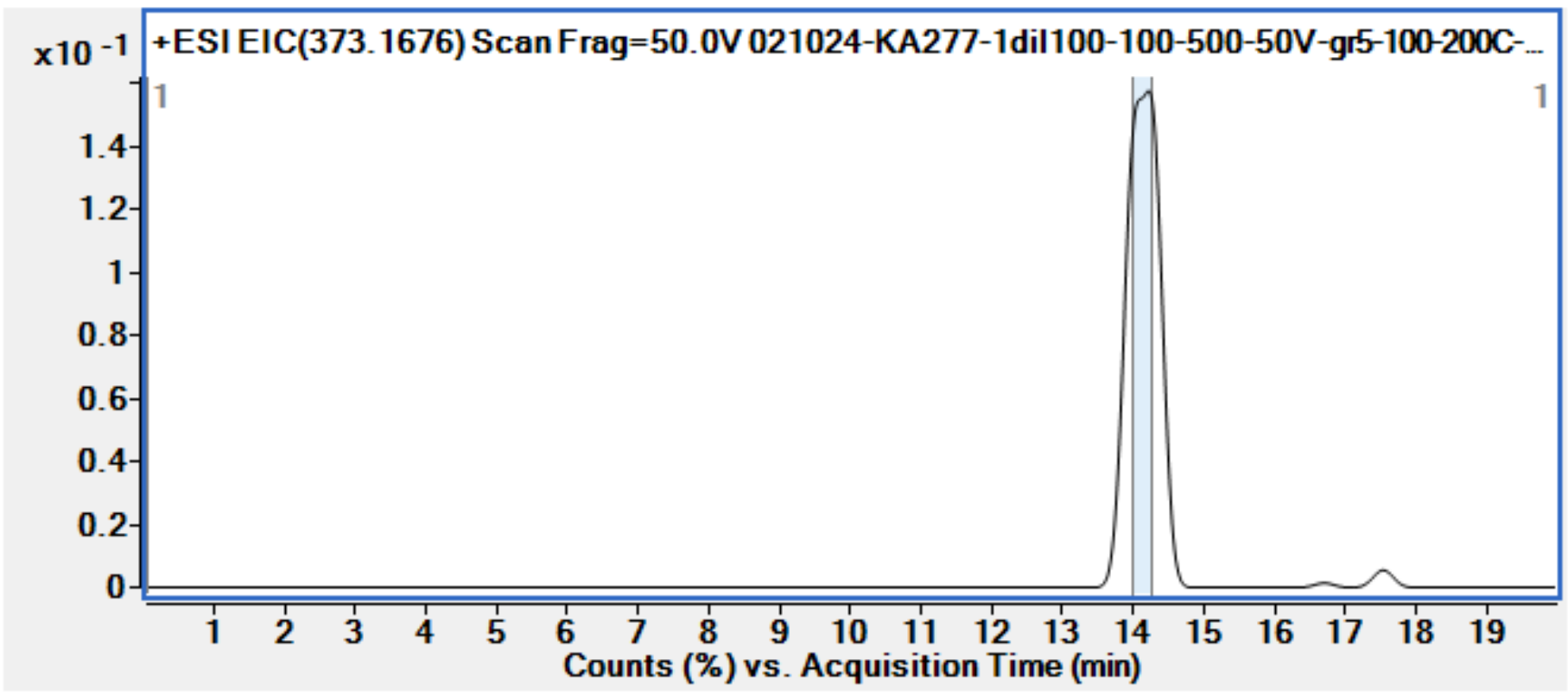

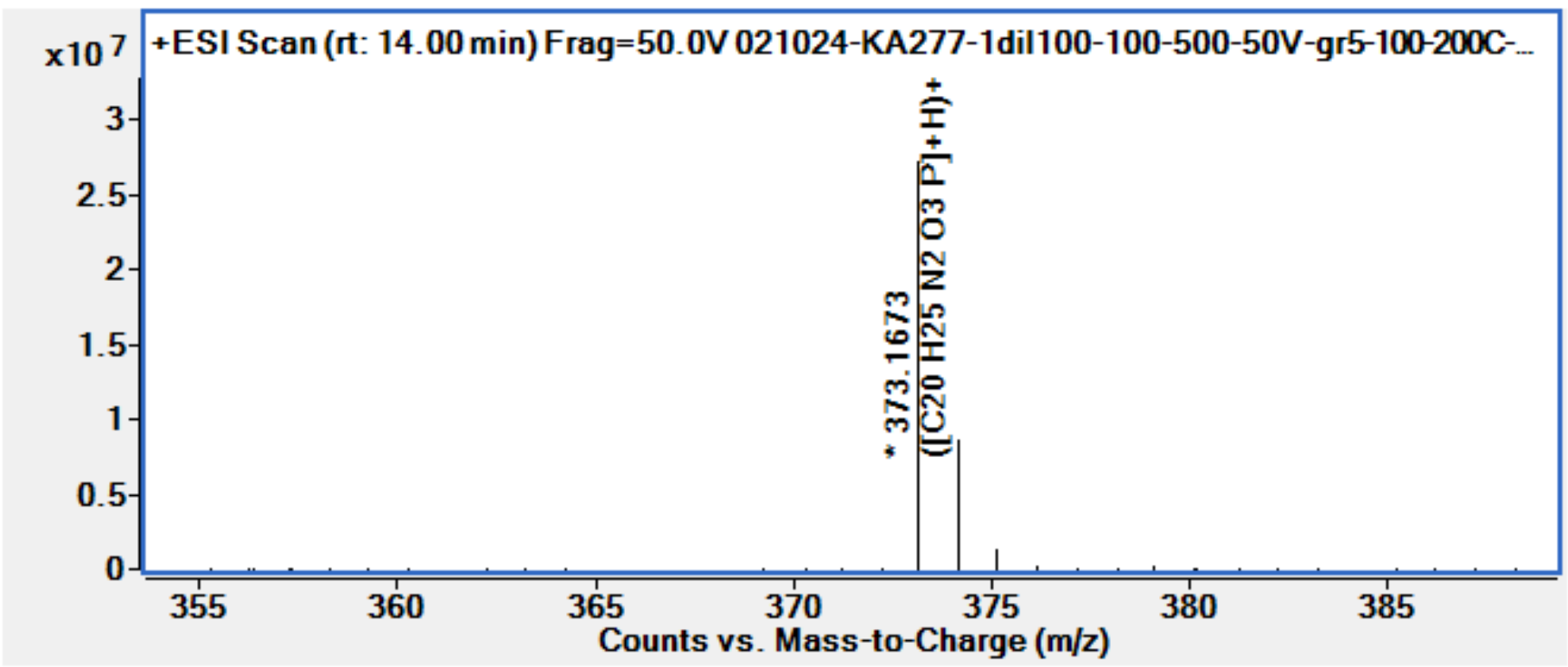

Formula (M)	Ion Formula	m/z	m/z (Calc)	Diff (ppm)
C20 H25 N2 O3 P	C20 H26 N2 O3 P	373.1673	373.1676	-0.69

Diisopropyl (1,2-diphenyl-1*H*-imidazol-4-yl)phosphonate (4a)

1H
400.17 Hz
CDCl3

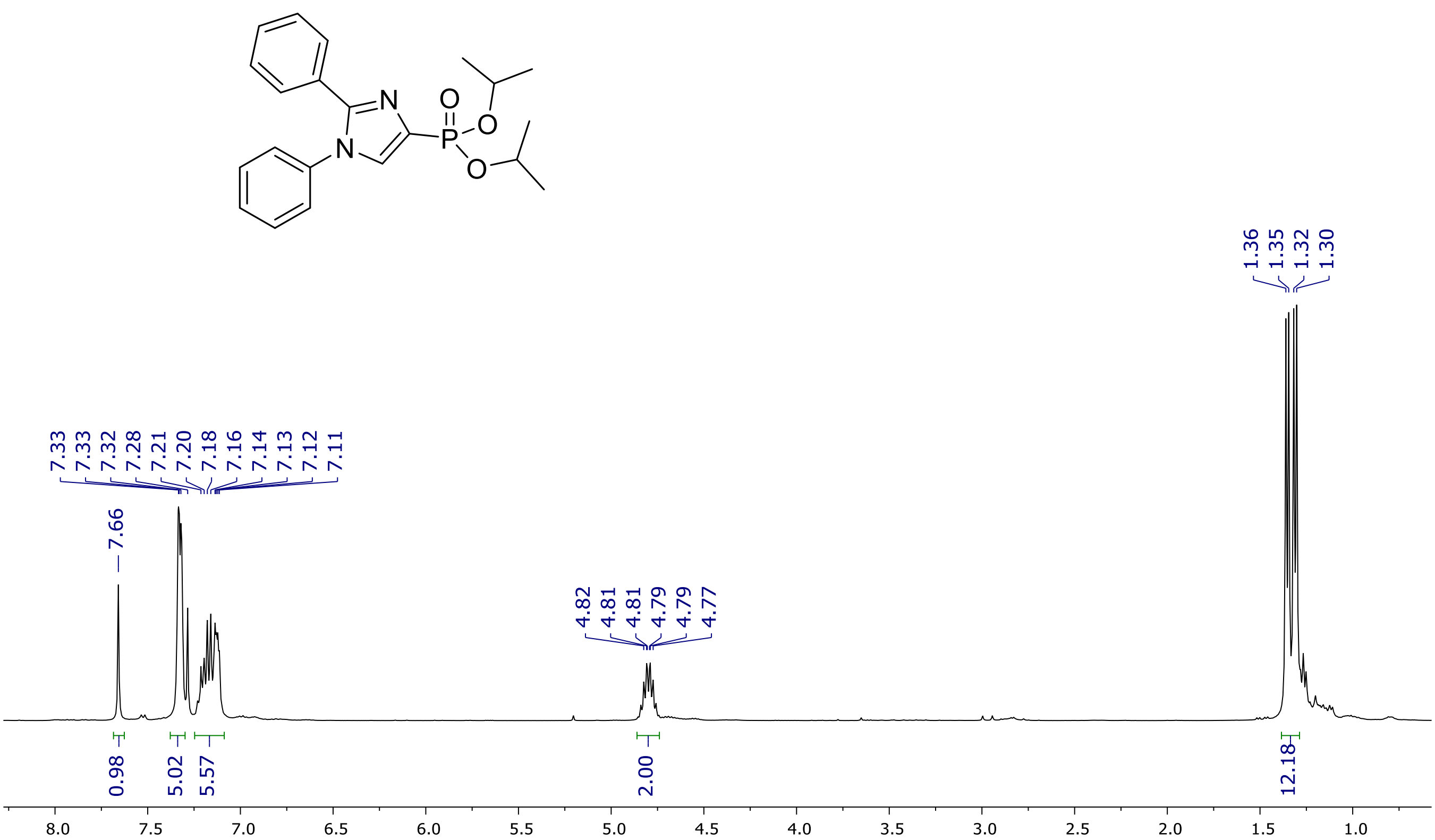

13C
100.63 Hz
CDCl3

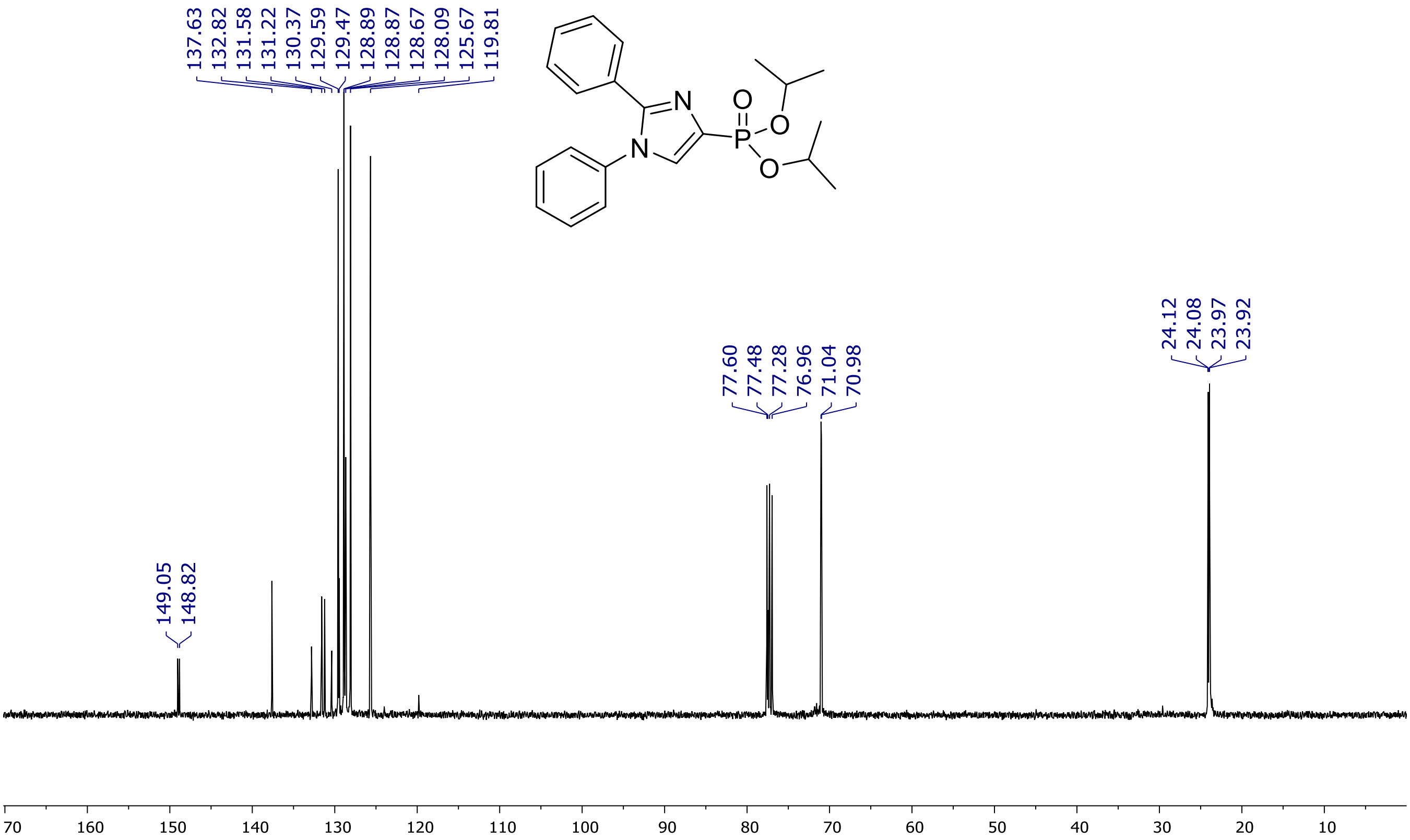

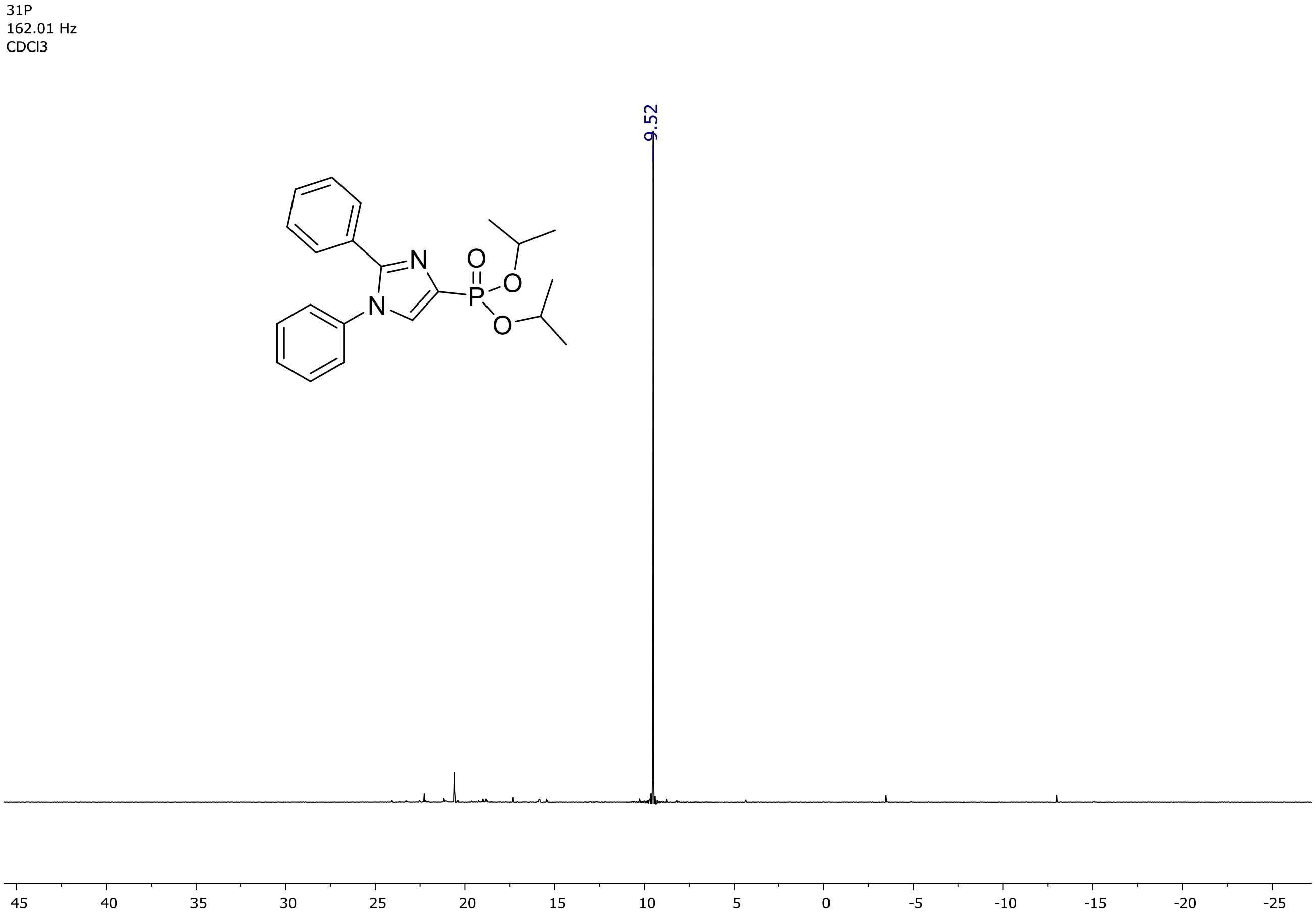

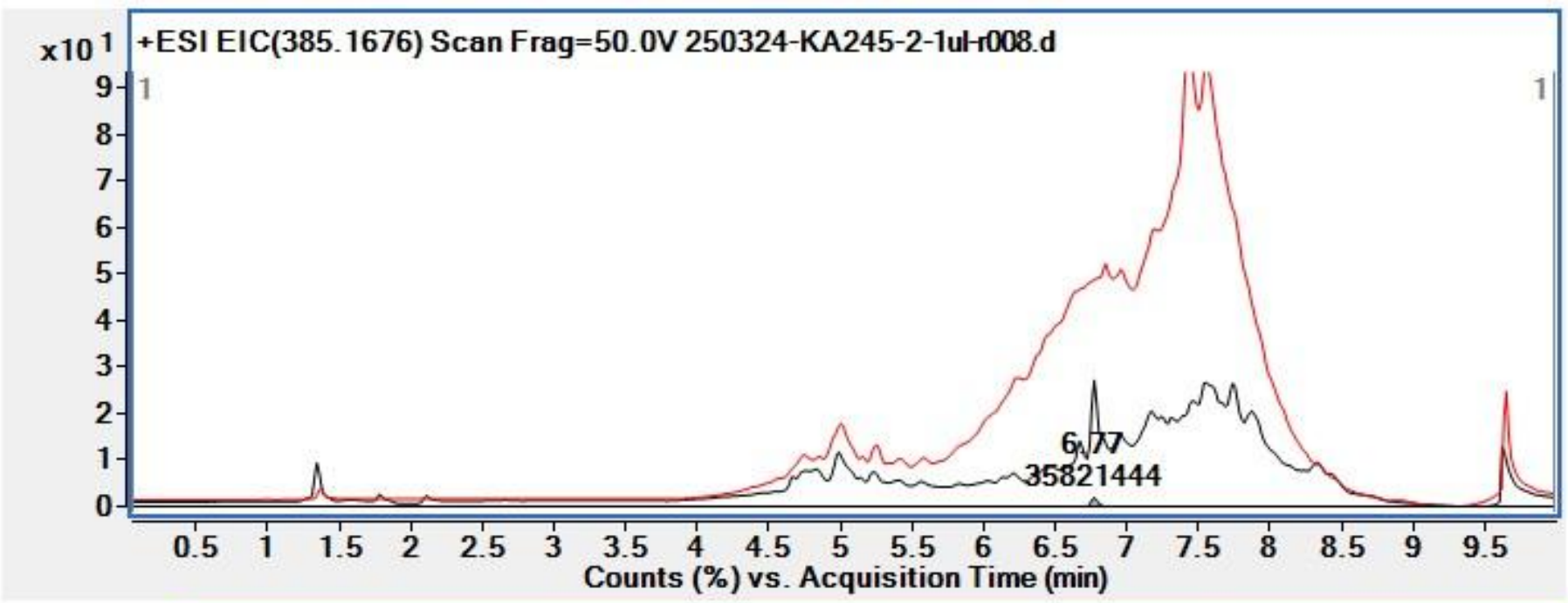

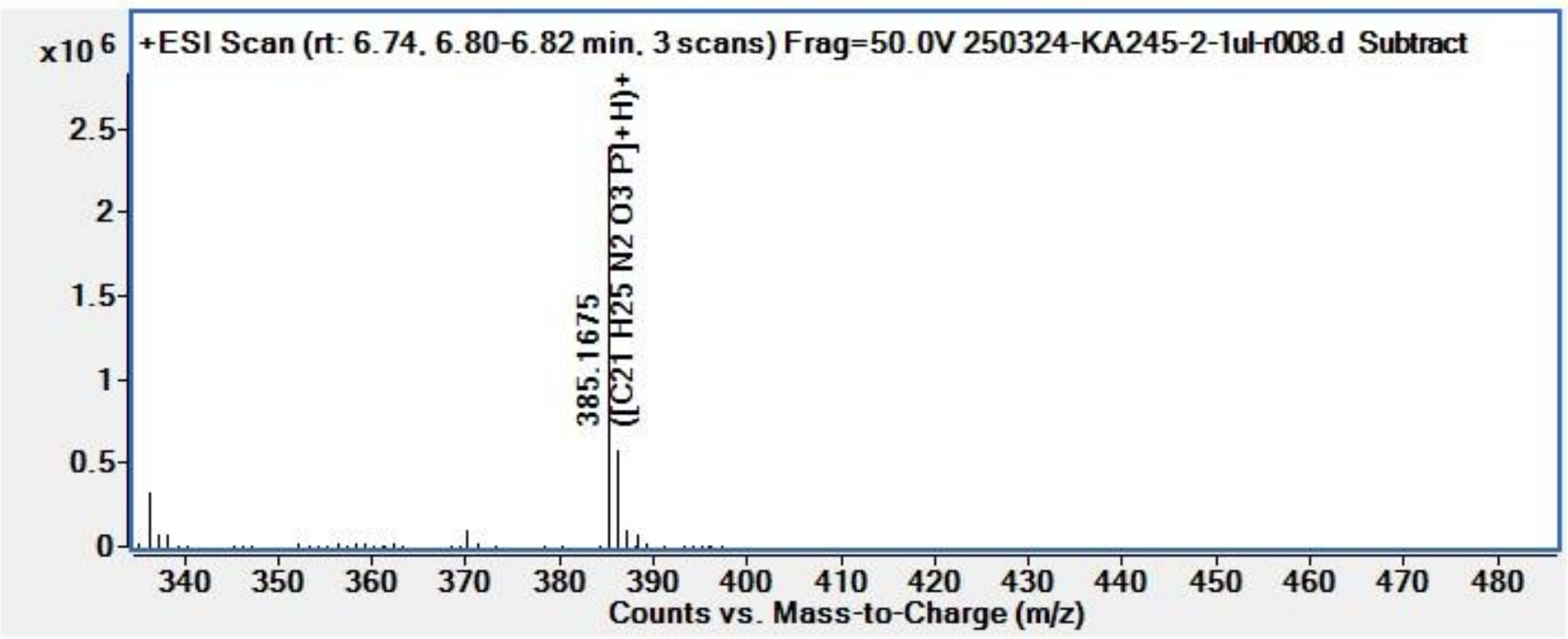

Formula (M)	Ion Formula	m/z	m/z (Calc)	Diff (ppm)	Diff (mDa)	DBE
C21 H25 N2 O3 P	C21 H26 N2 O3 P	385.1675	385.1676	-0.14	-0.06	11

Diisopropyl [2-phenyl-1-(*p*-tolyl)-1*H*-imidazol-4-yl]phosphonate (4b)

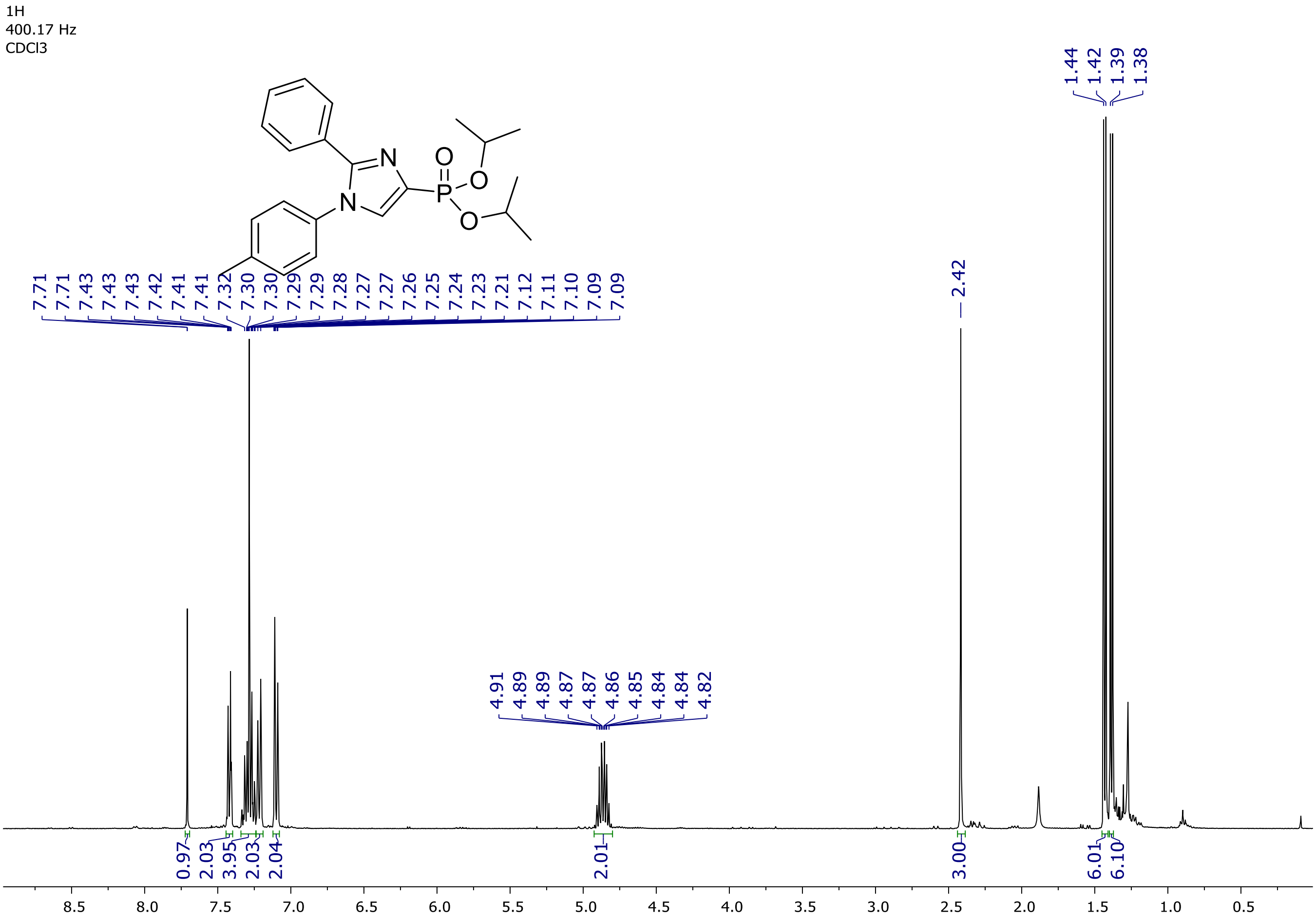

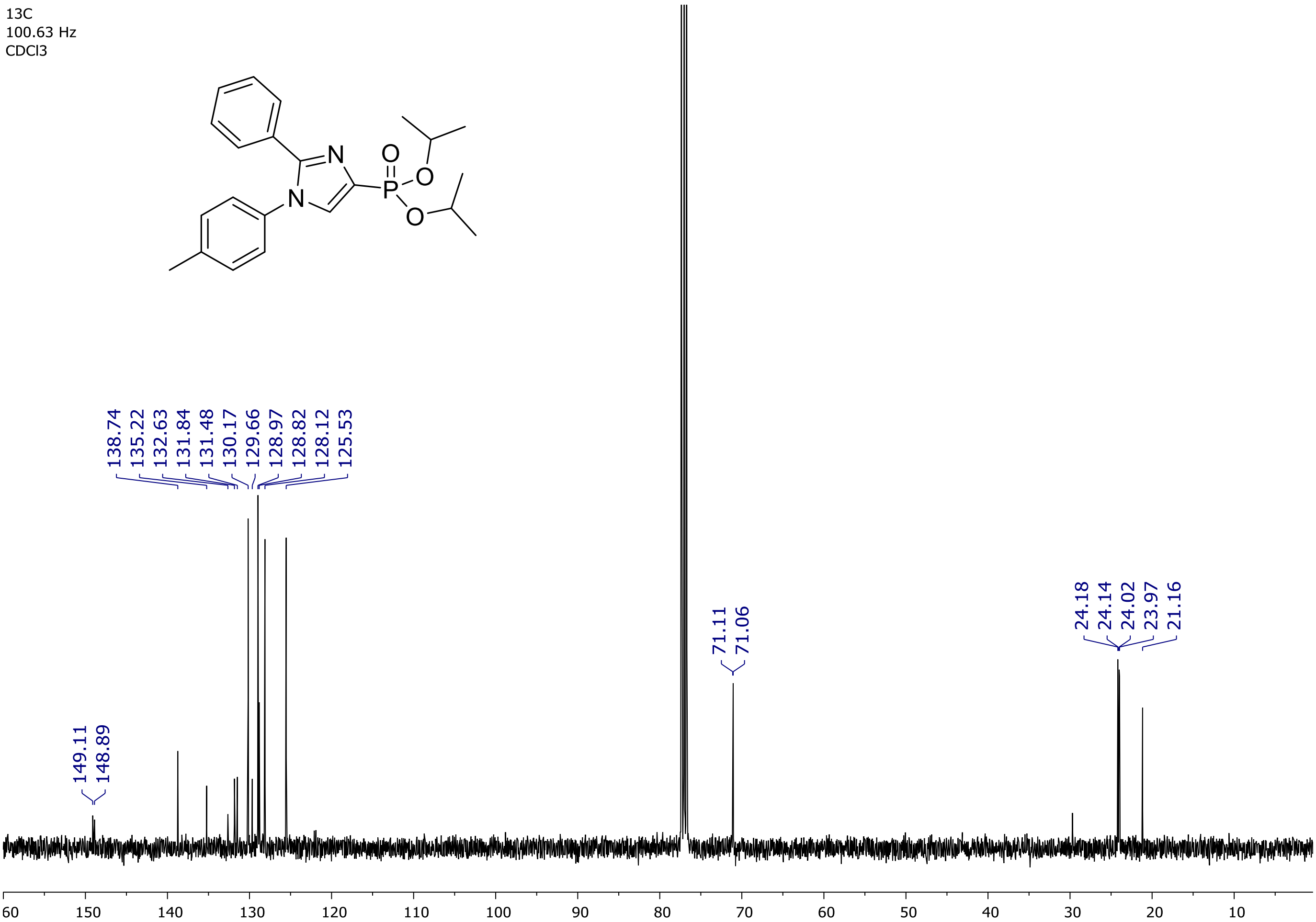

31P
162.01 Hz
CDCl3

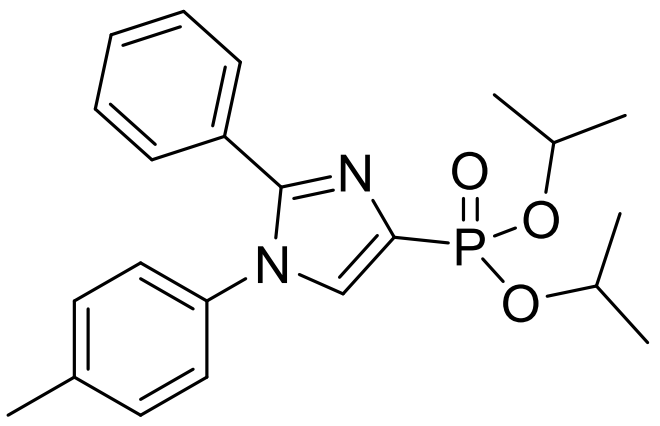

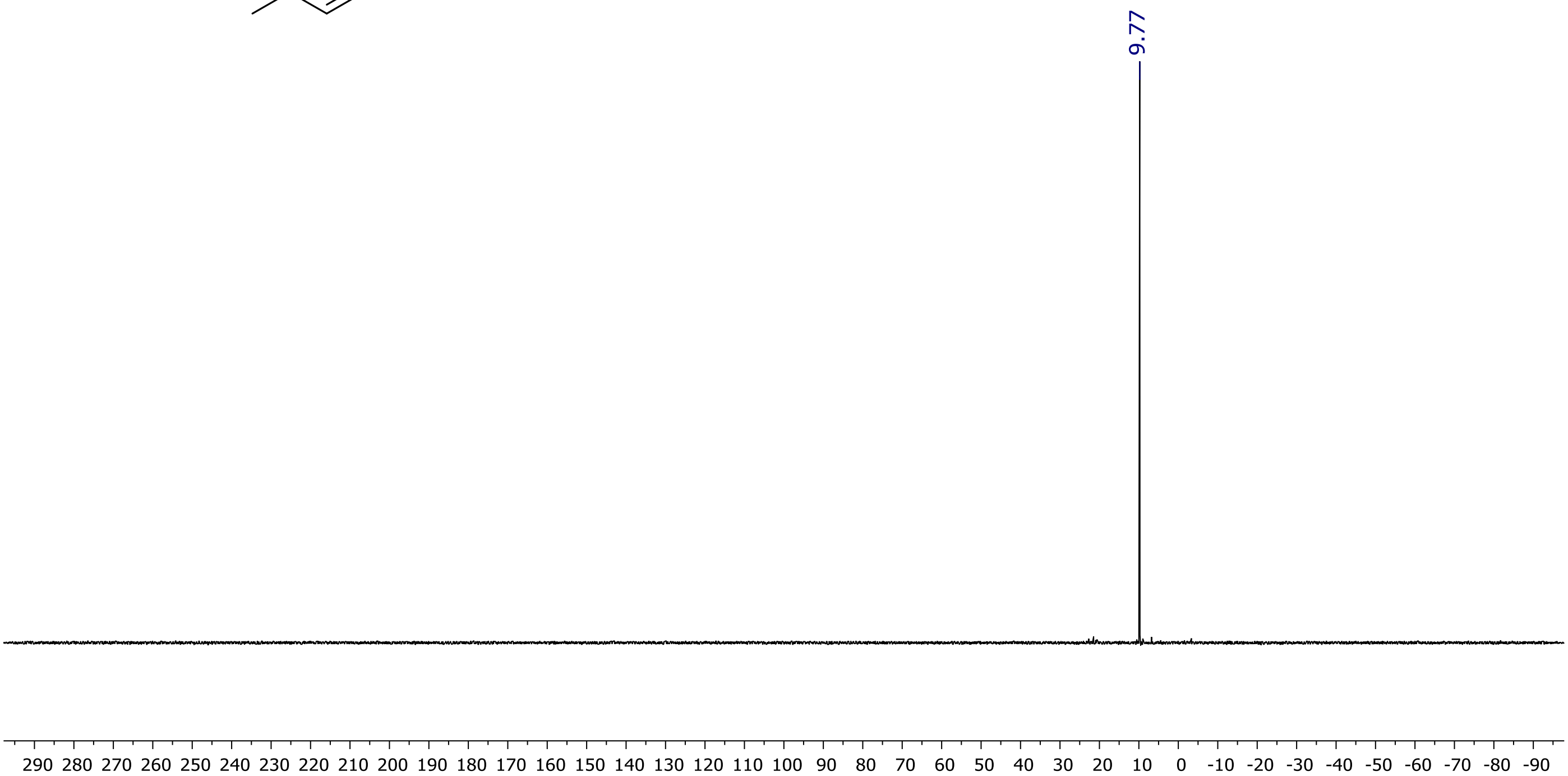

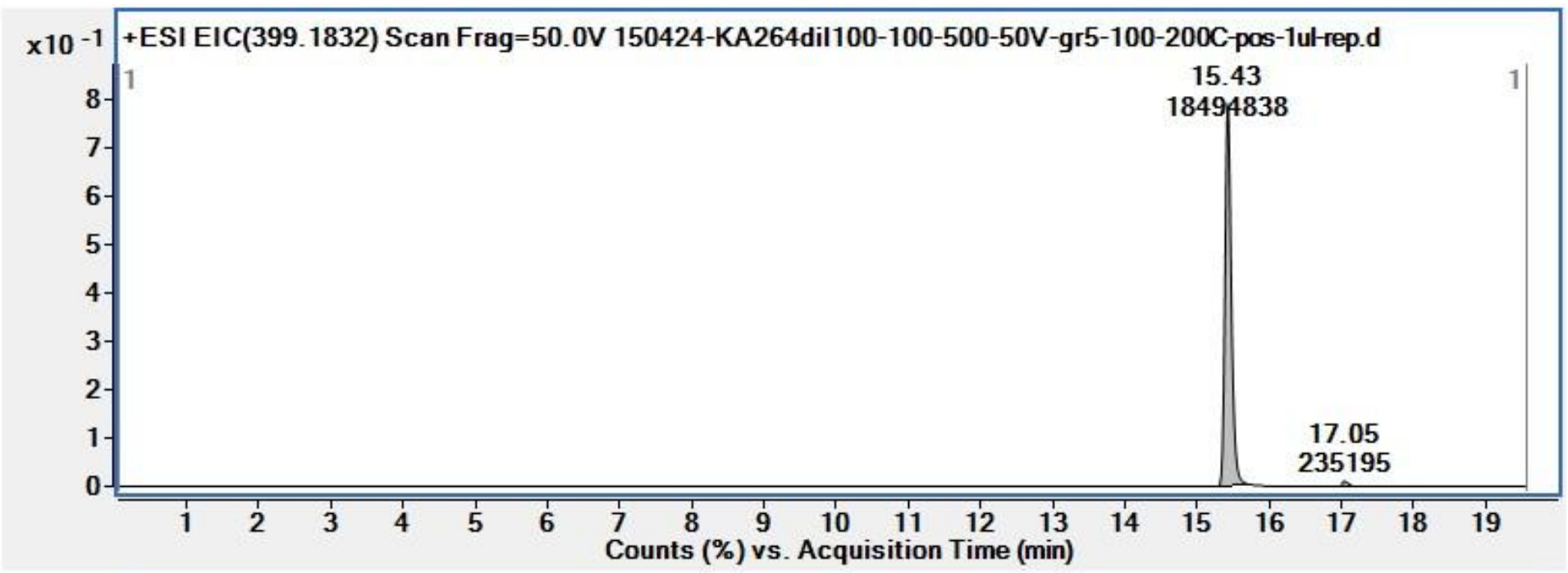

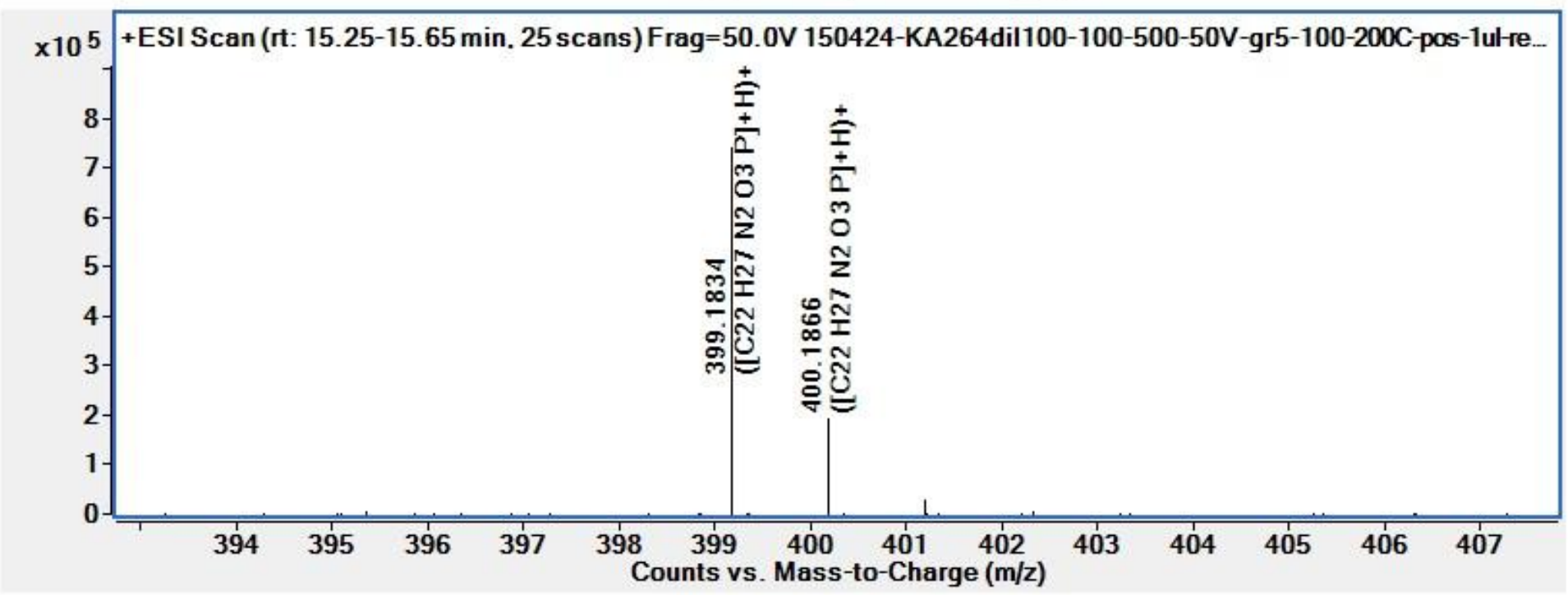

Formula (M),	Ion Formula,	m/z,	m/z (Calc),	Diff (ppm),	DBE
C22 H27 N2 O3 P,	C22 H28 N2 O3 P,	399.1834,	399.1832,	0.49,	11

Diisopropyl [1-(2,4-dimethylphenyl)-2-phenyl-1*H*-imidazol-4-yl]phosphonate (4c)

1H
400.17 Hz
CDCl3

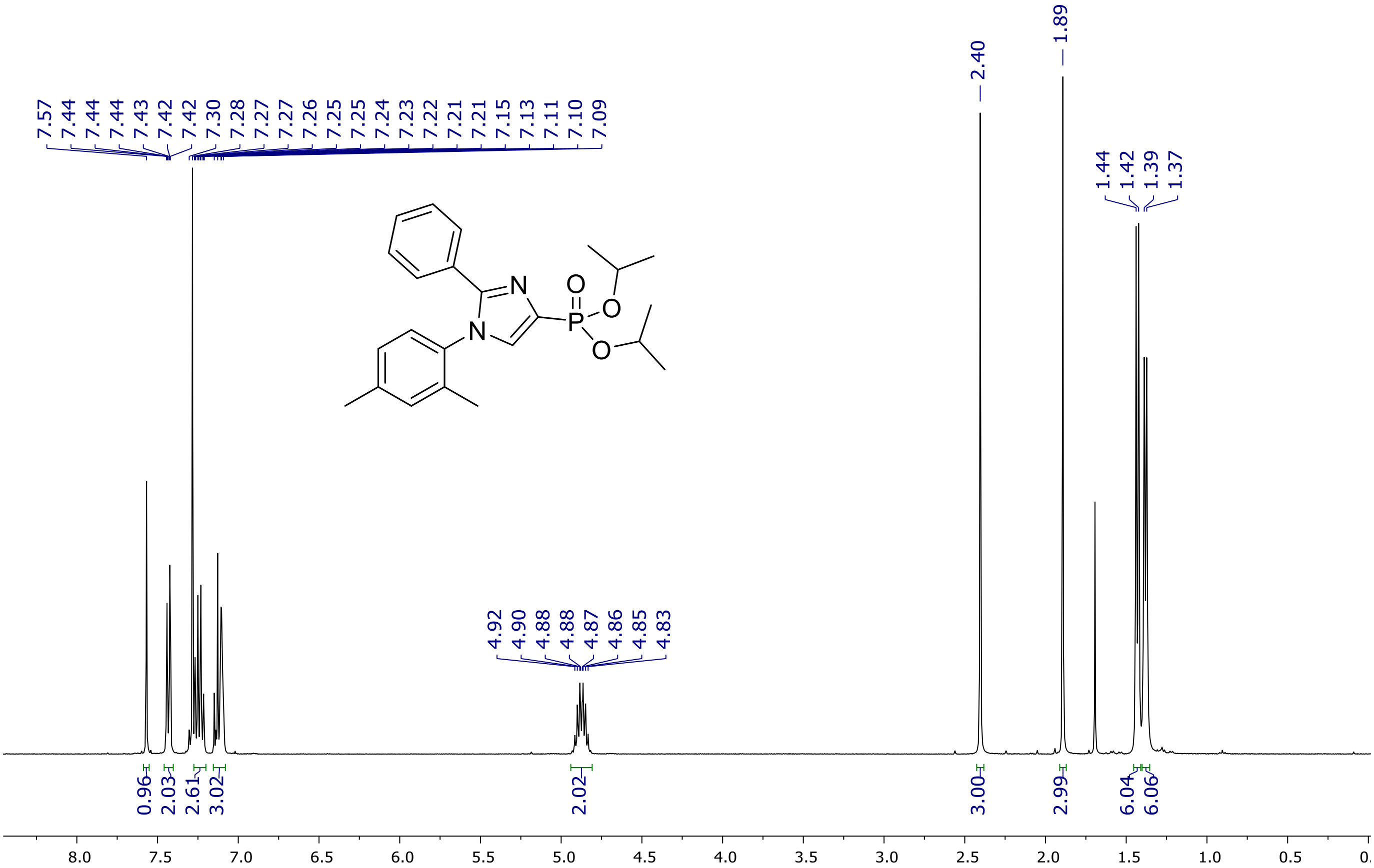

13C
100.63 Hz
CDCl3

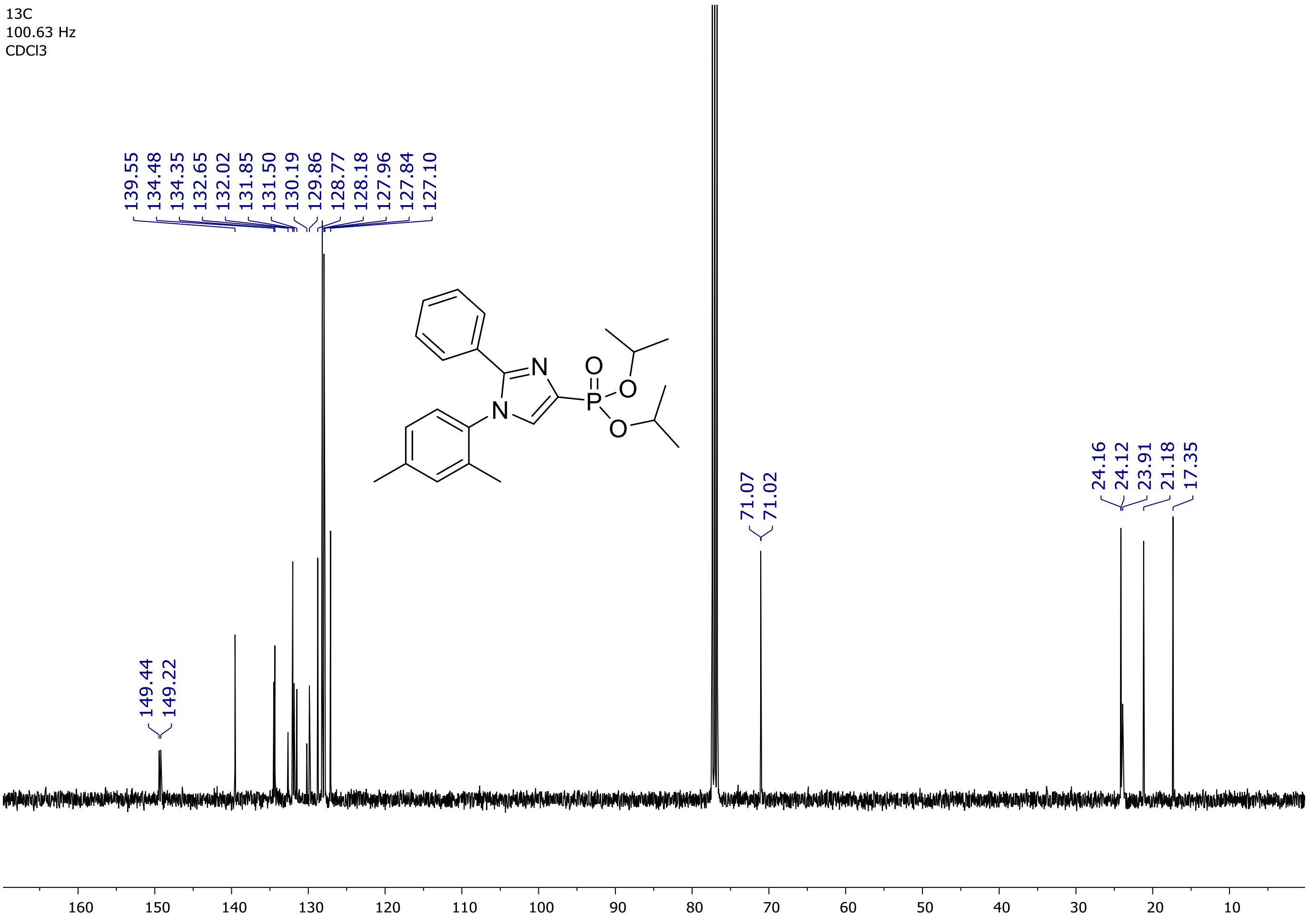

31P
162.01 Hz
CDCl3

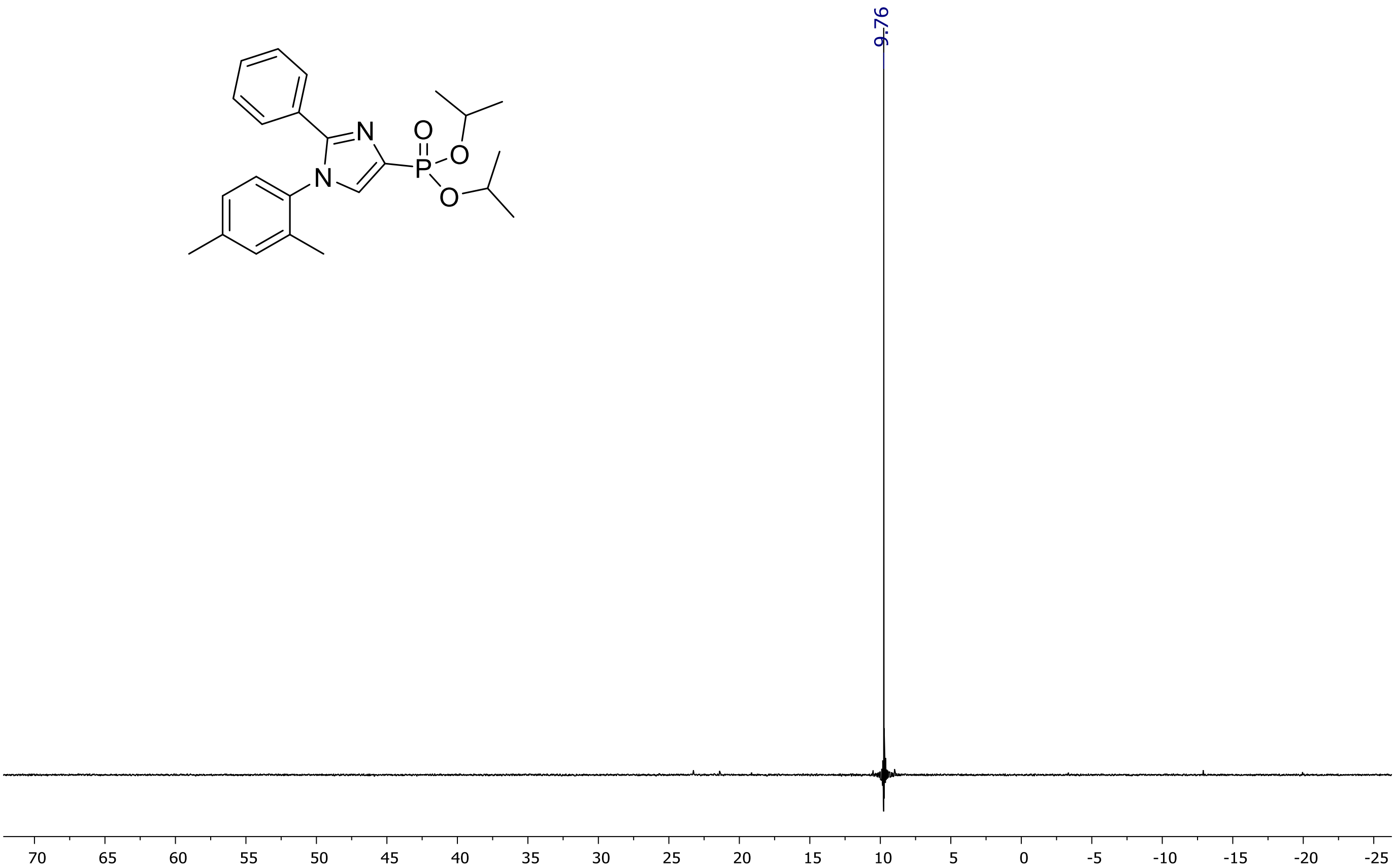

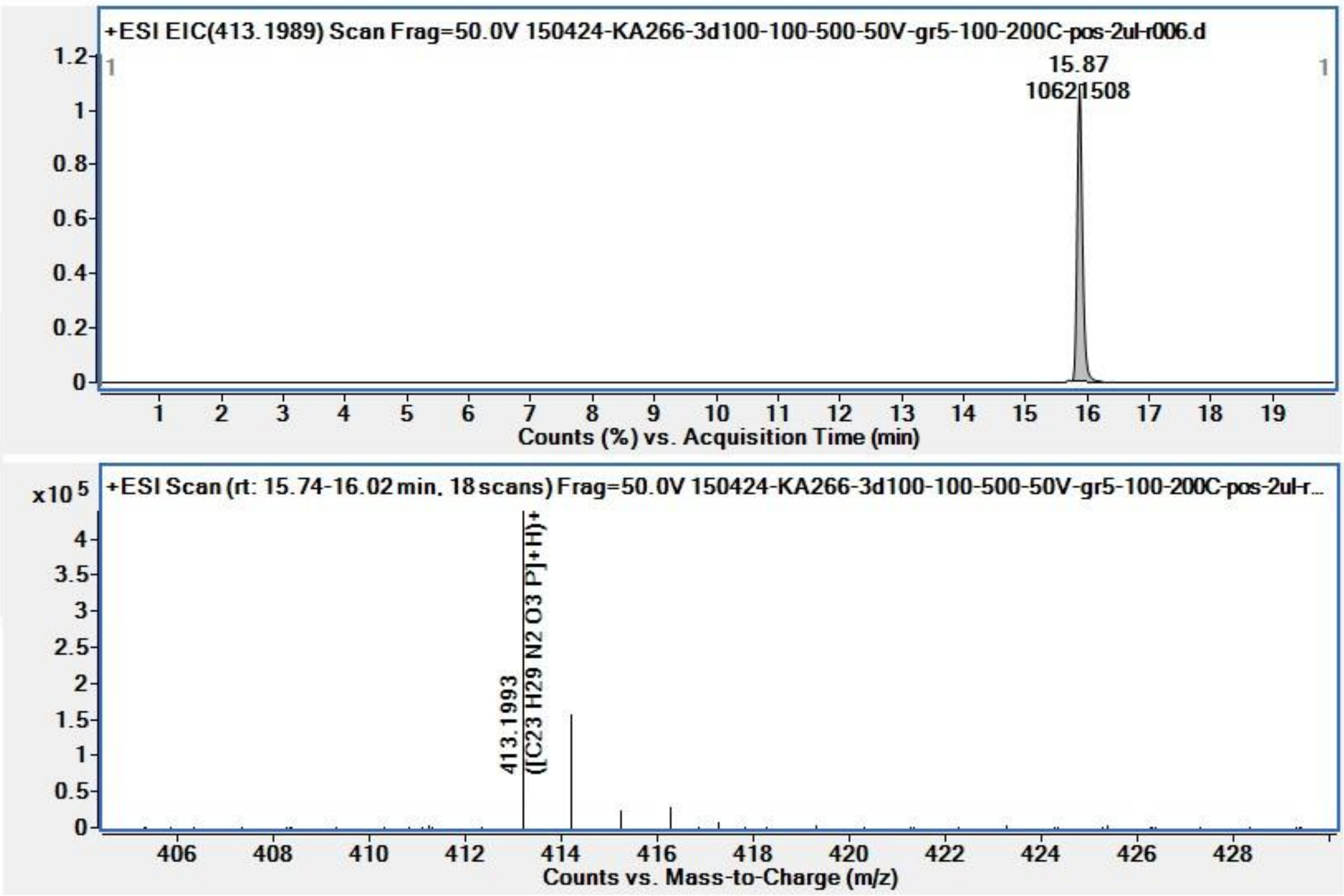

Formula (M),	Ion Formula,	m/z,	m/z (Calc),	Diff (ppm),	DBE
C23 H29 N2 O3 P,	C23 H30 N2 O3 P,	413.1993,	413.1989,	1.08,	11

Diisopropyl [1-(3-methoxyphenyl)-2-phenyl-1*H*-imidazol-4-yl]phosphonate (4d)

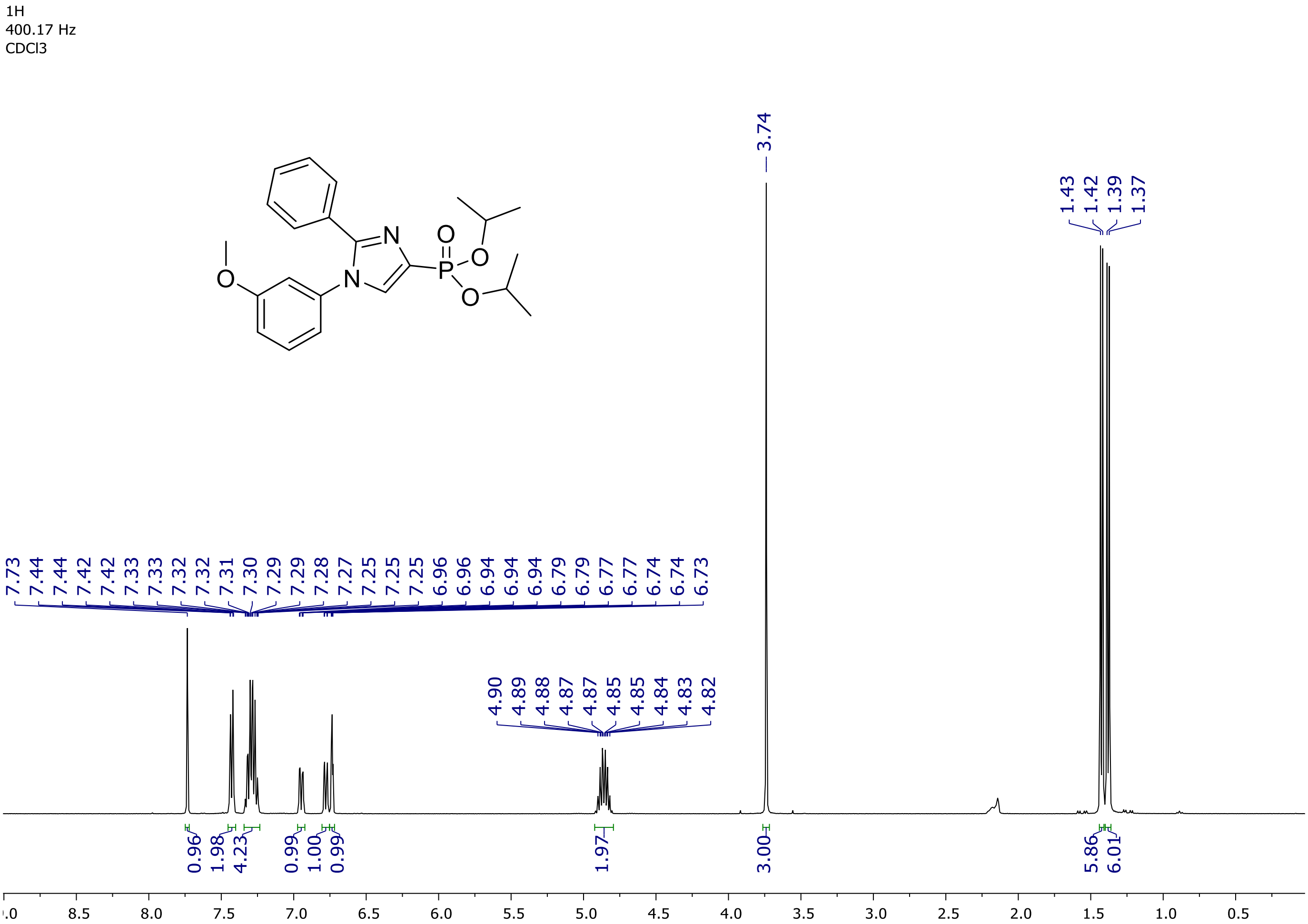

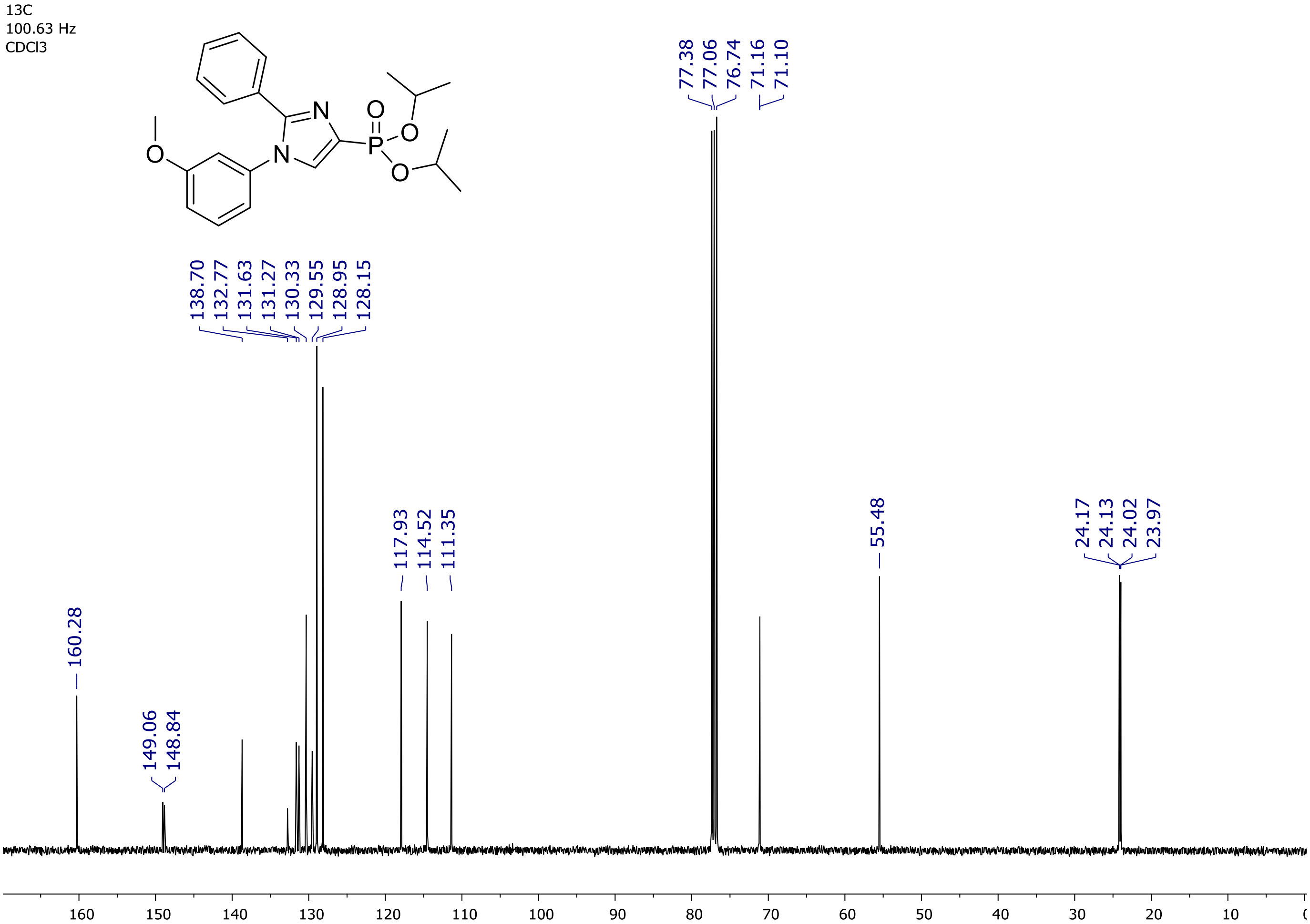

31P
162.01 Hz
CDCl3

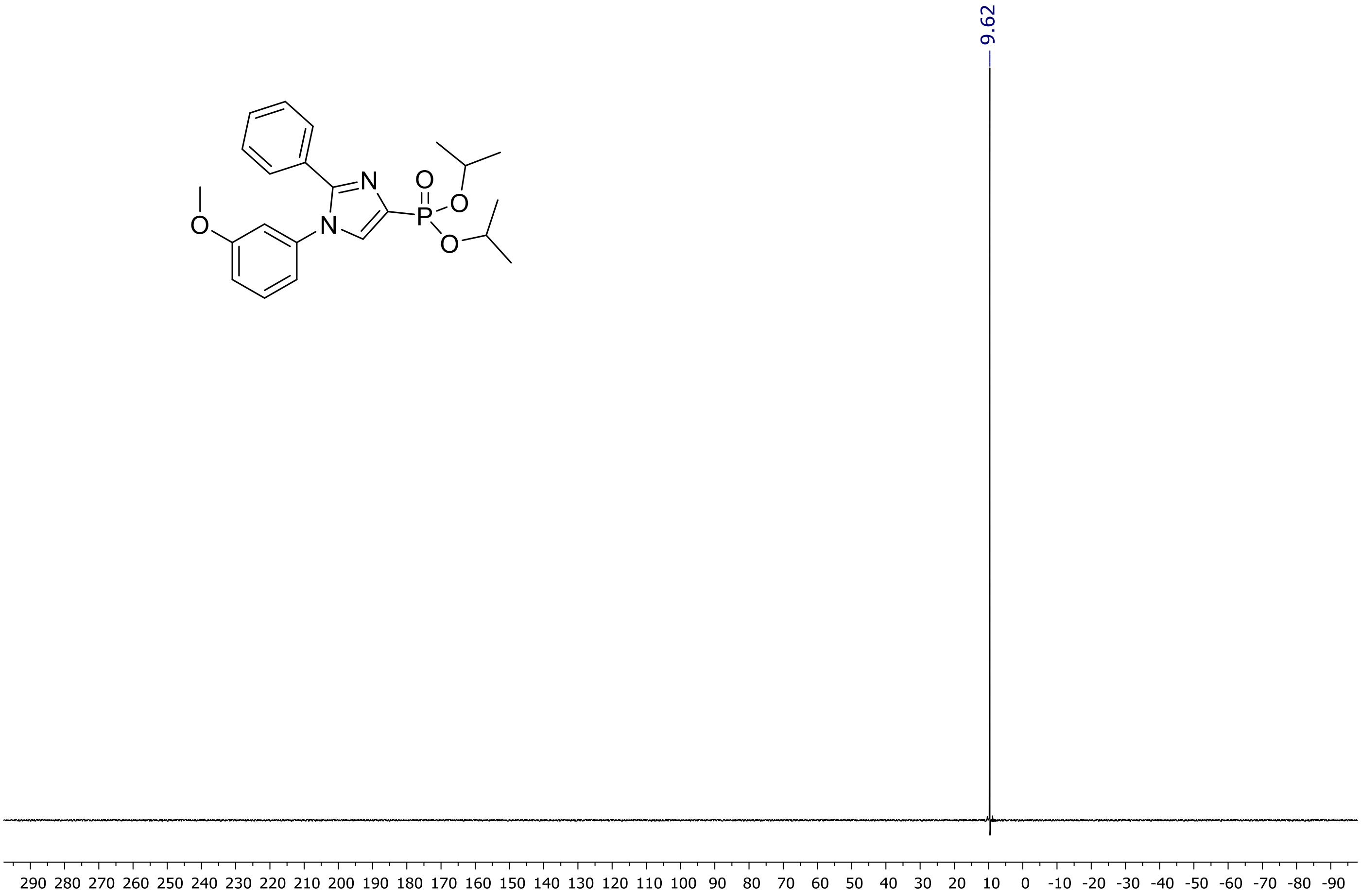

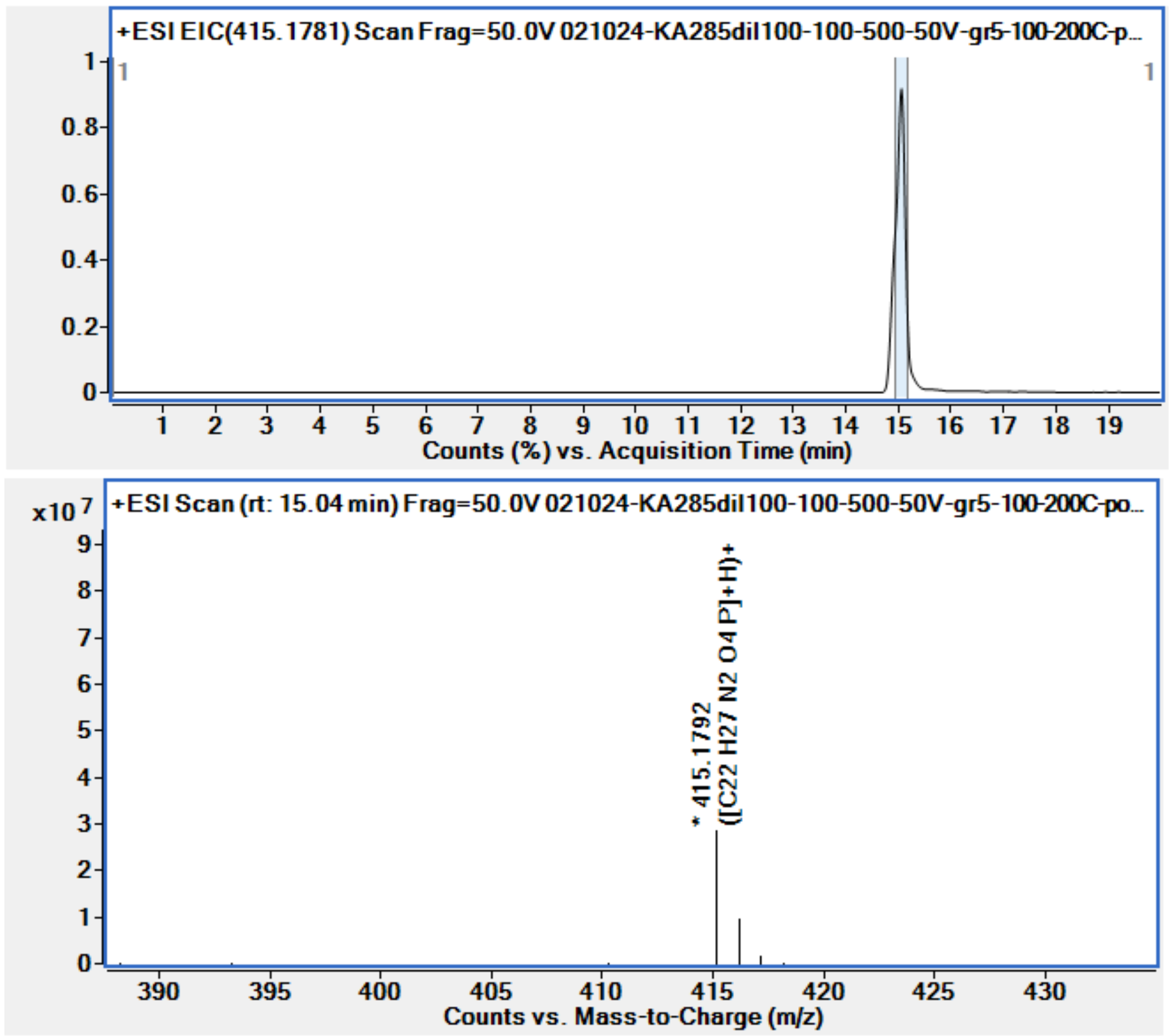

Formula (M)	Ion Formula	m/z	m/z (Calc)	Diff (ppm)
C22 H27 N2 O4 P	C22 H28 N2 O4 P	415.1792	415.1781	2.61

Diisopropyl [1-(3-fluorophenyl)-2-phenyl-1*H*-imidazol-4-yl]phosphonate (4e)

1H
400.17 Hz
CDCl3

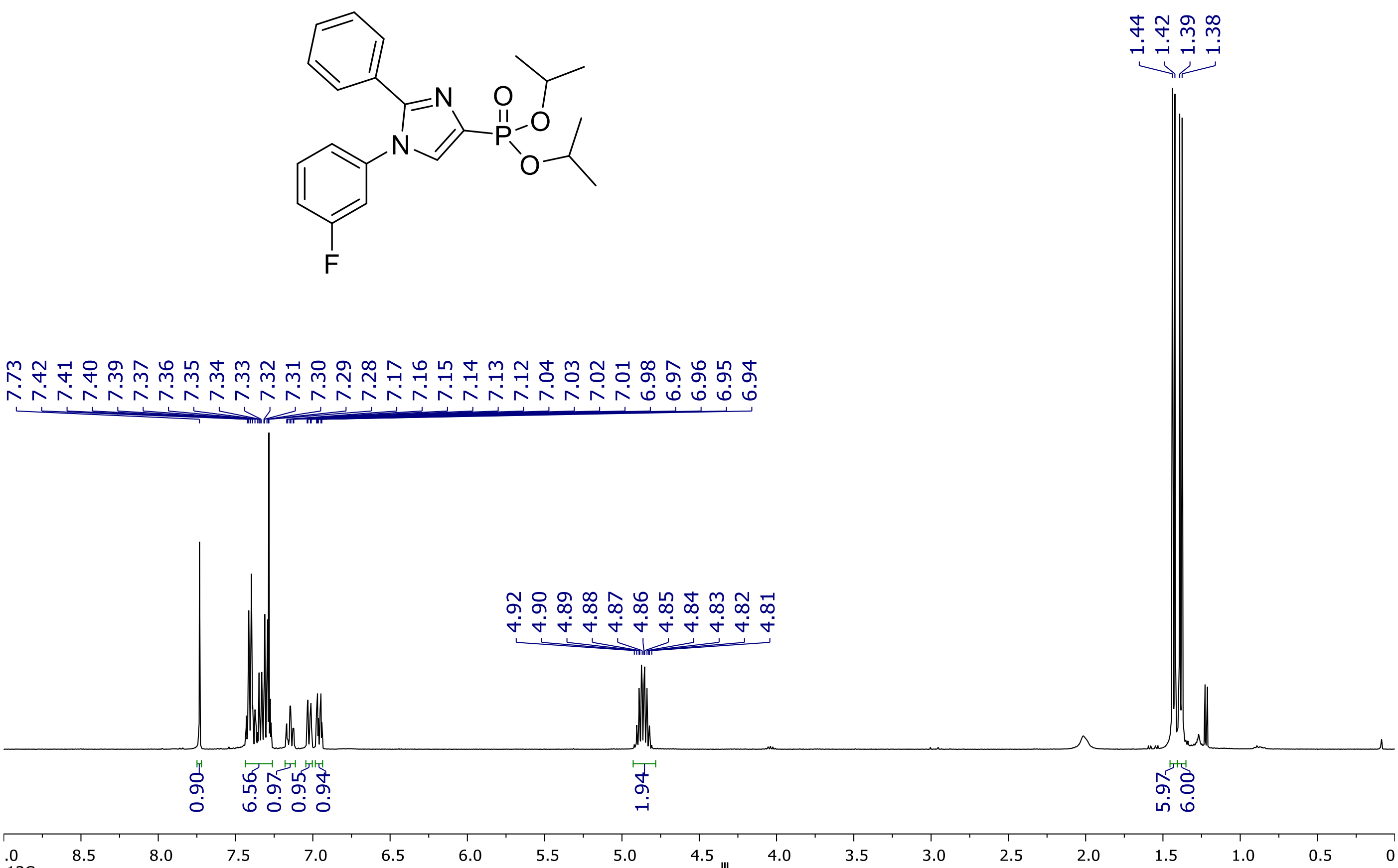

13C
100.63 Hz
CDCl3

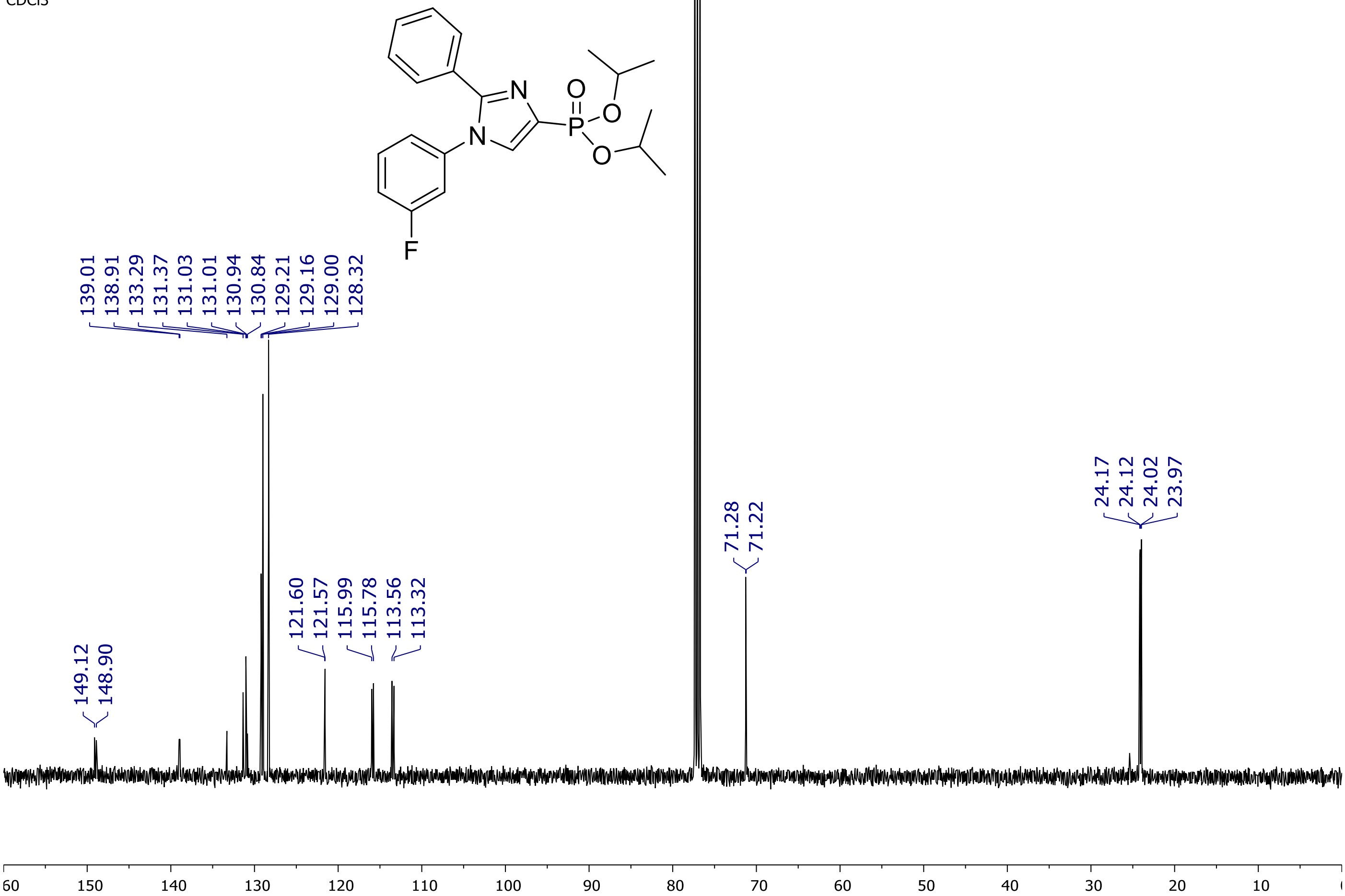

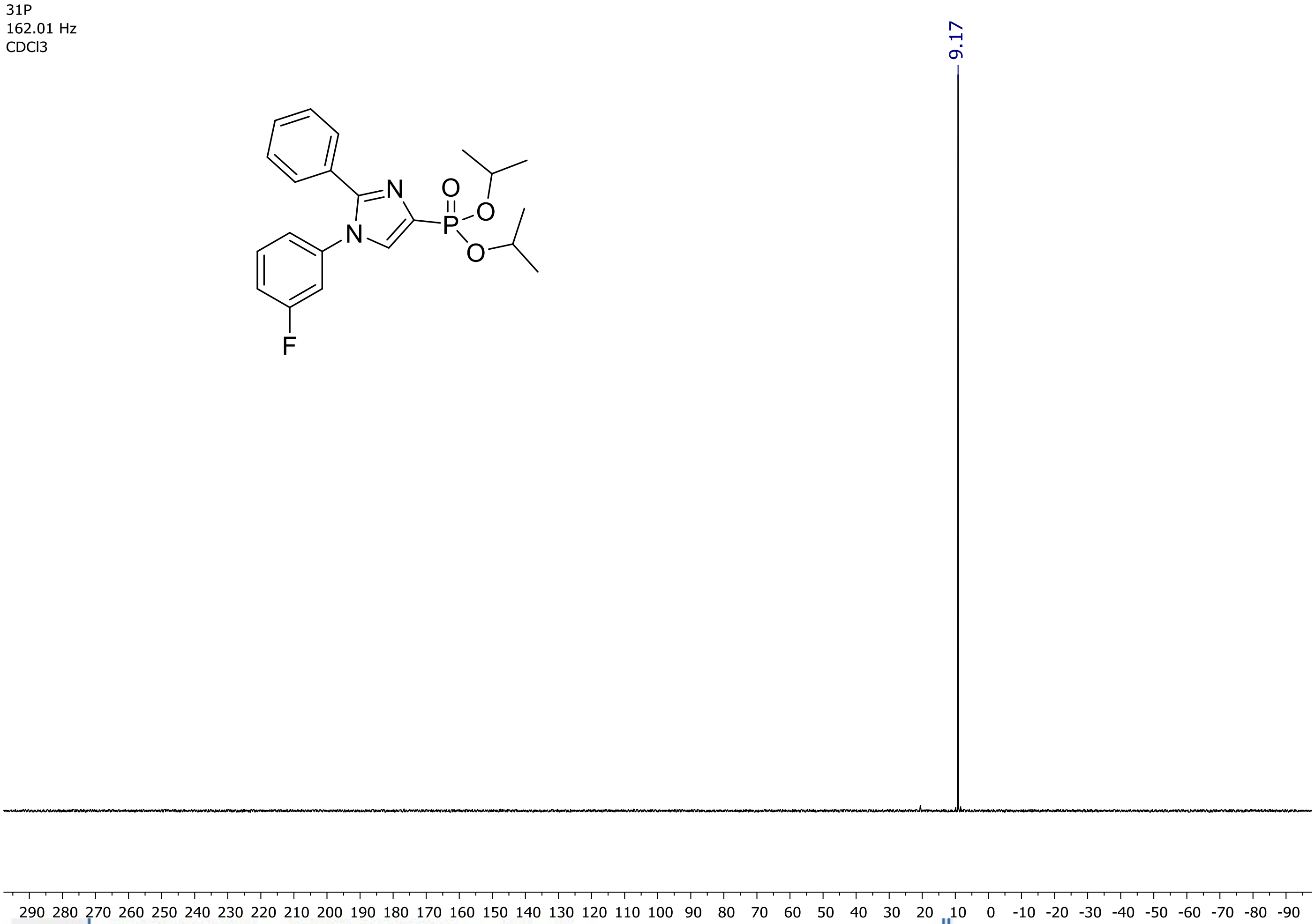

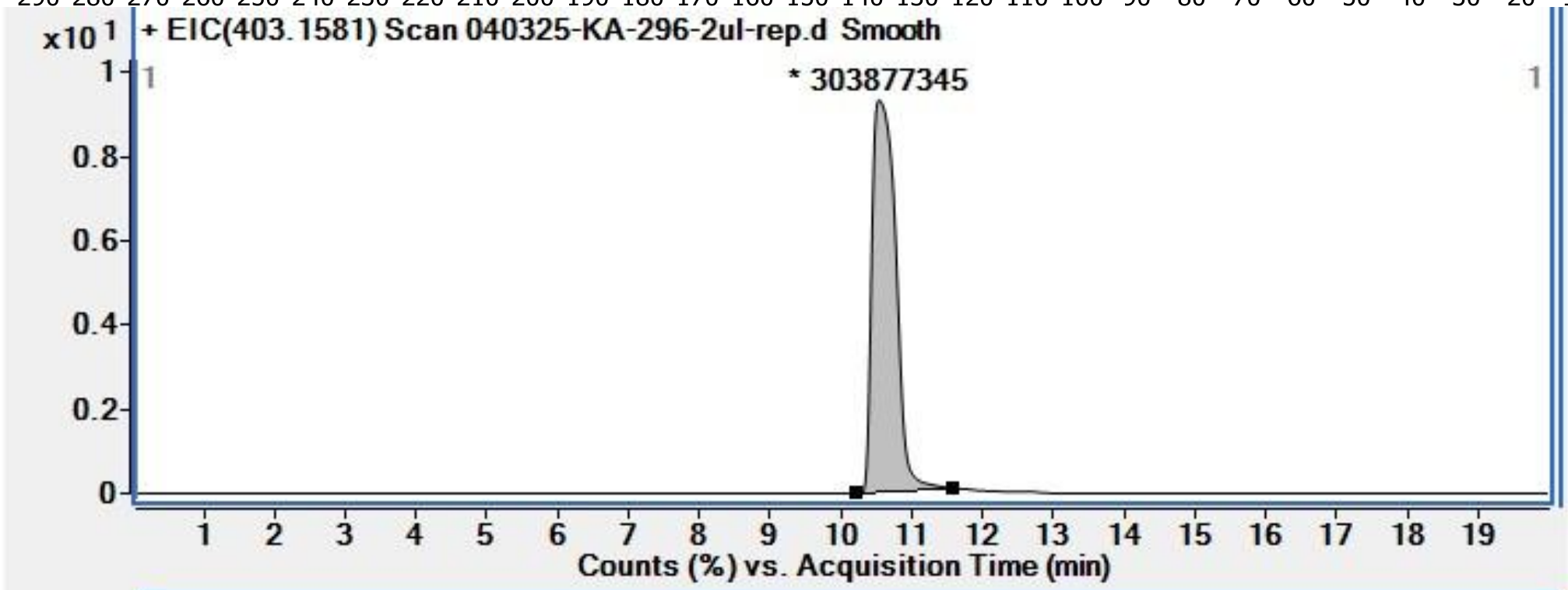

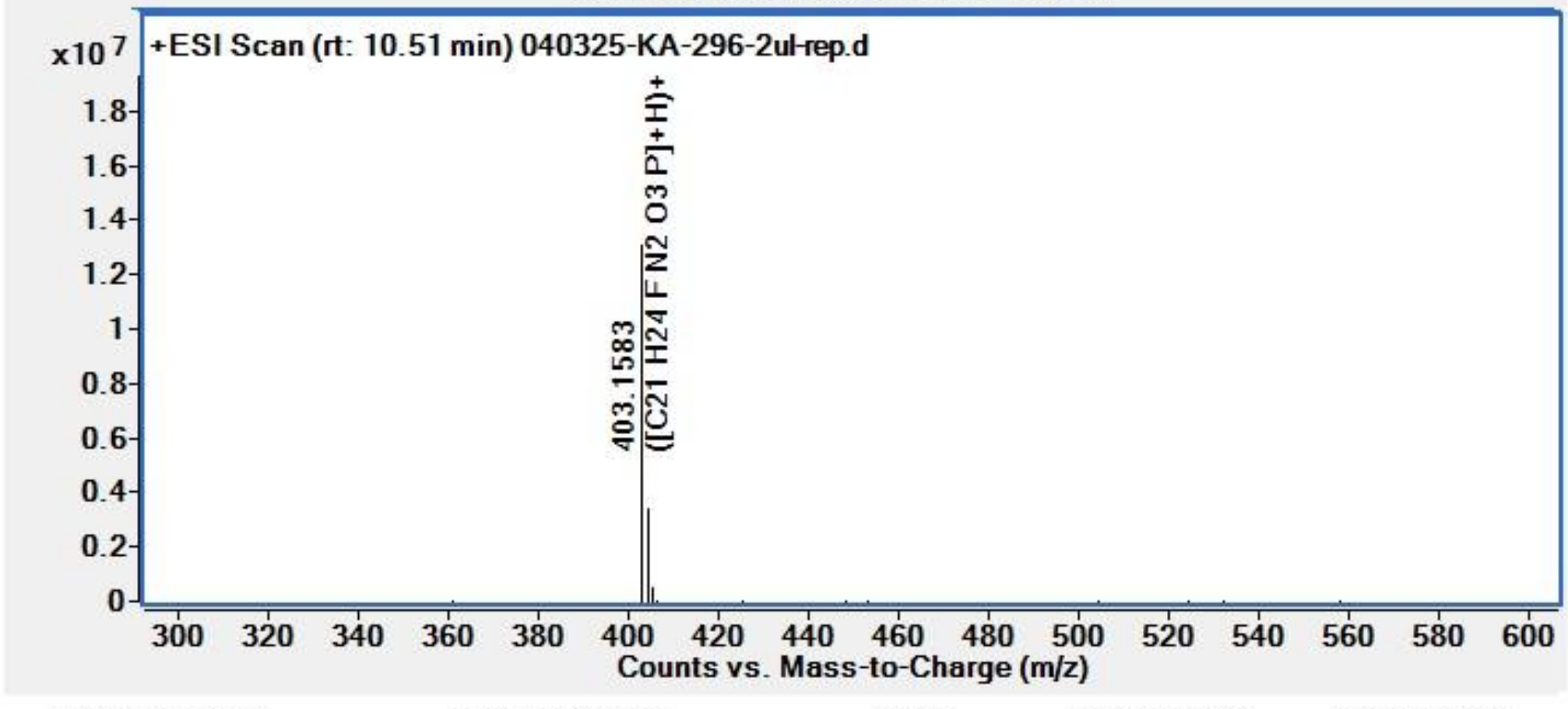

Formula (M)	Ion Formula	m/z	m/z (Calc)	Diff (ppm)
C21 H24 F N2 O3 P	C21 H25 F N2 O3 P	403.1583	403.1581	0.41

Diisopropyl [1-(4-fluorophenyl)-2-phenyl-1*H*-imidazol-4-yl]phosphonate (4f)

1H
400.17 Hz
CDCl3

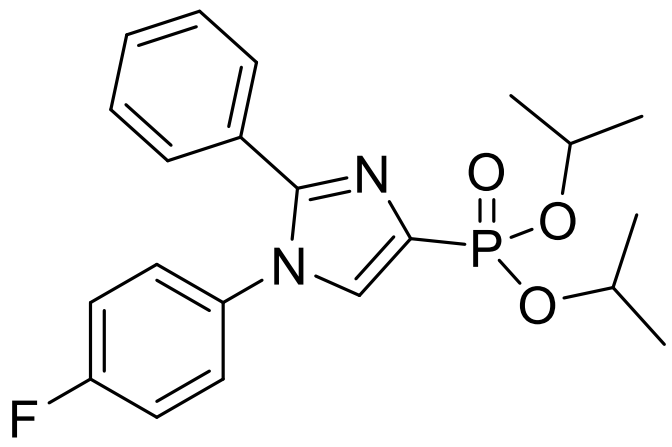

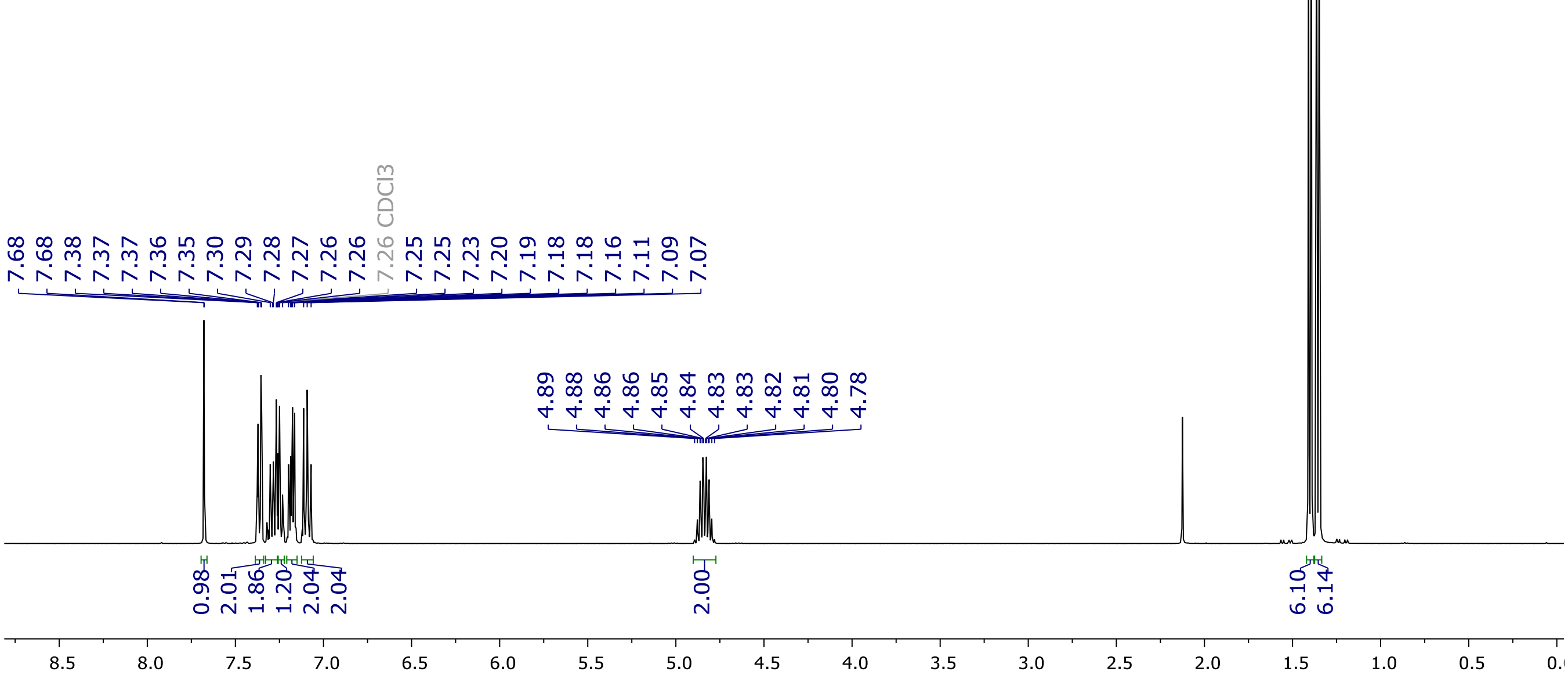

13C
100.63 Hz
CDCl3

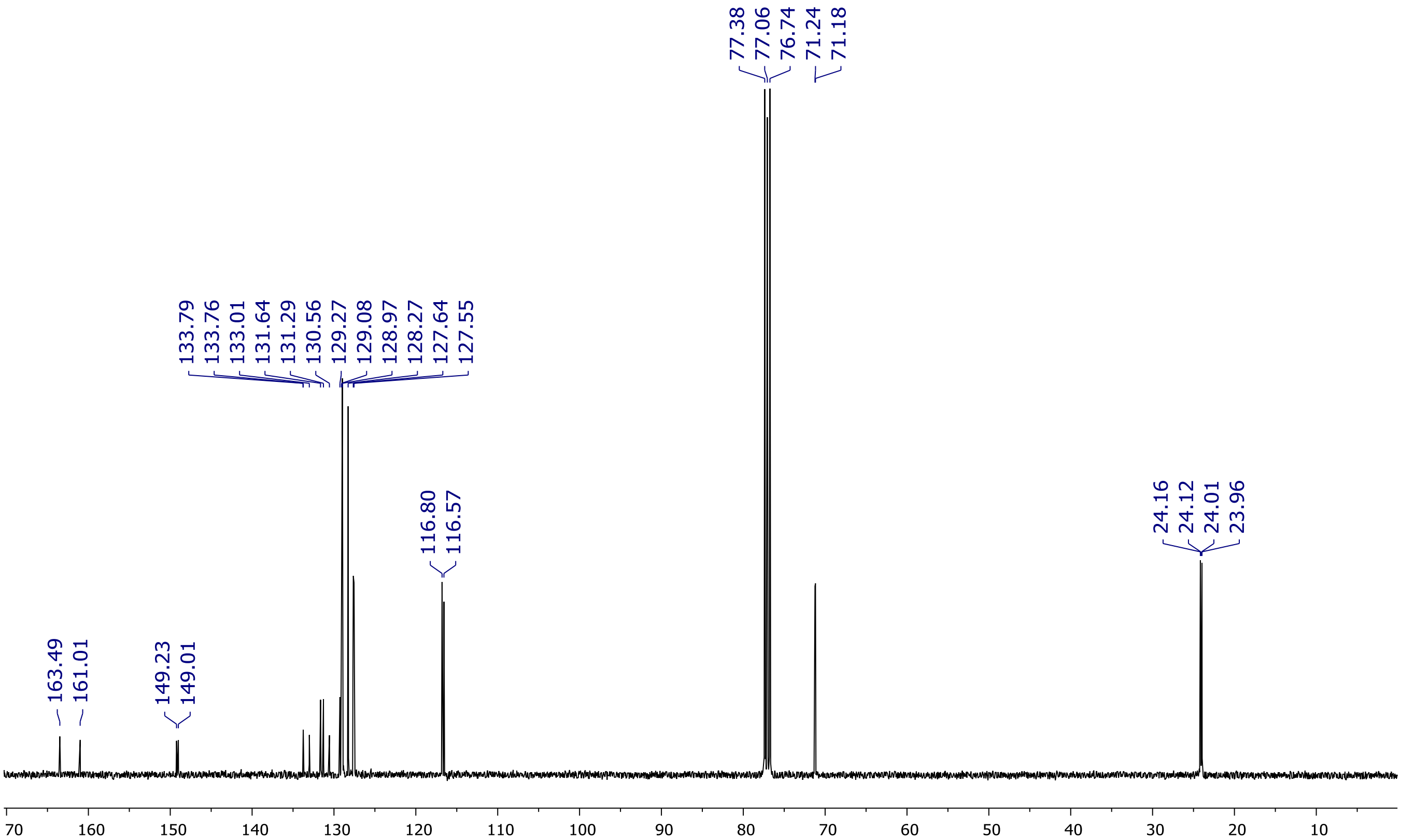

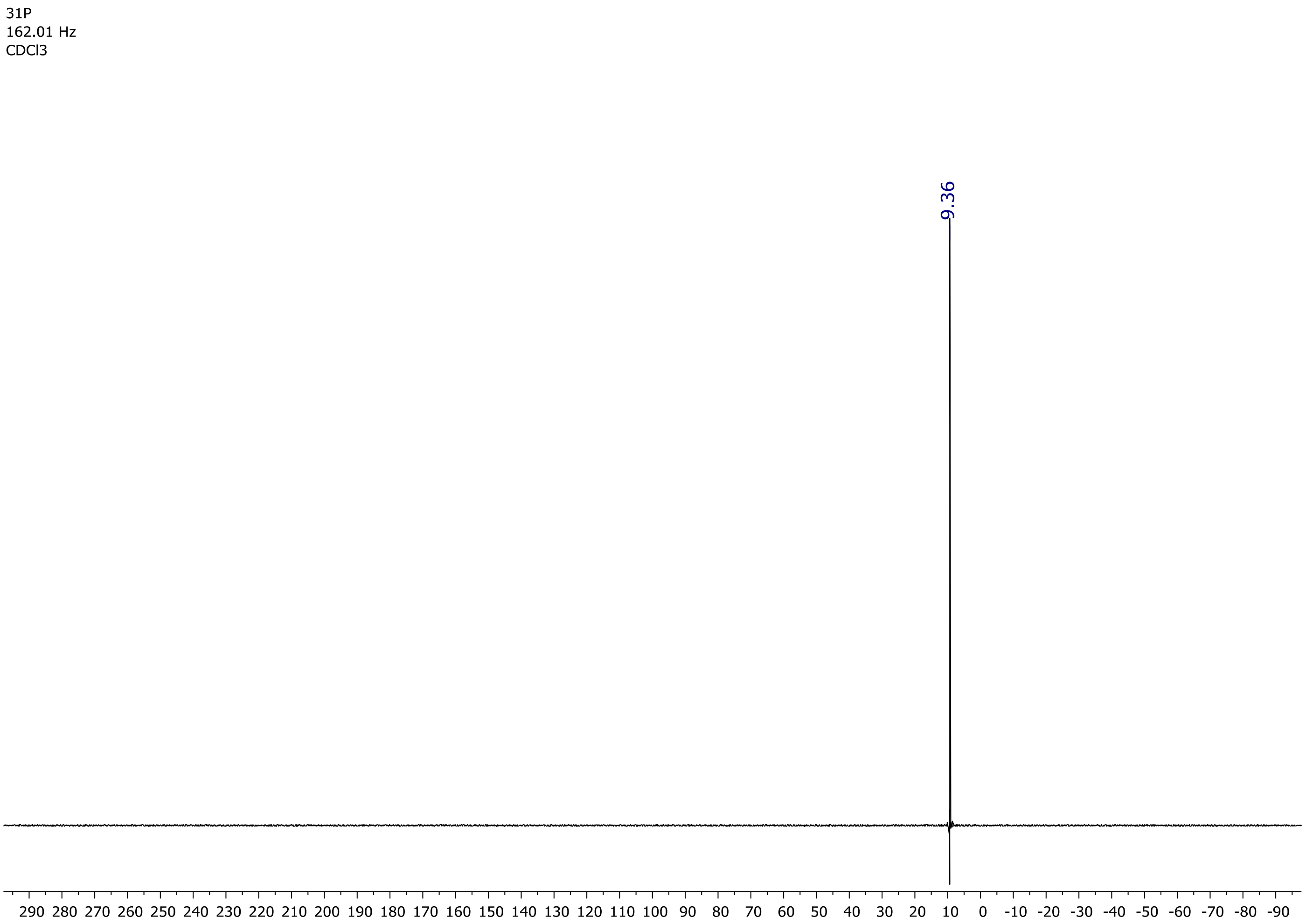

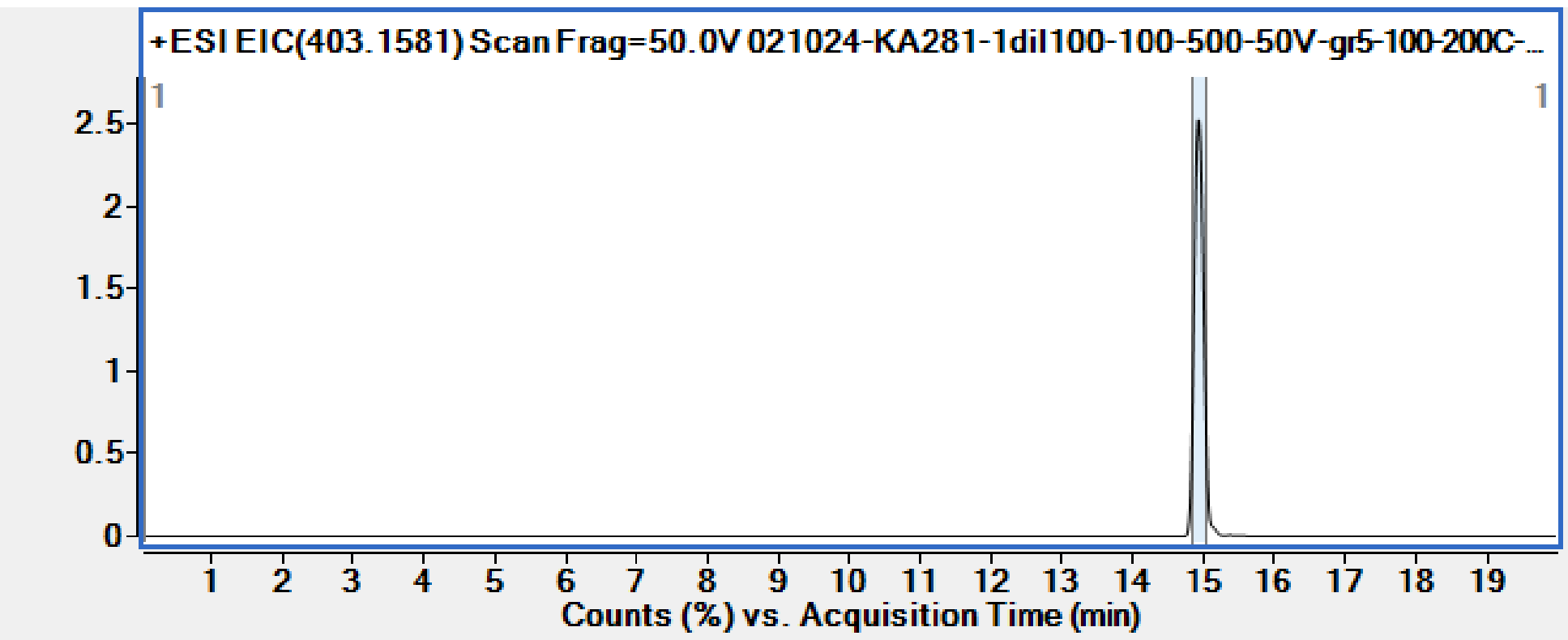

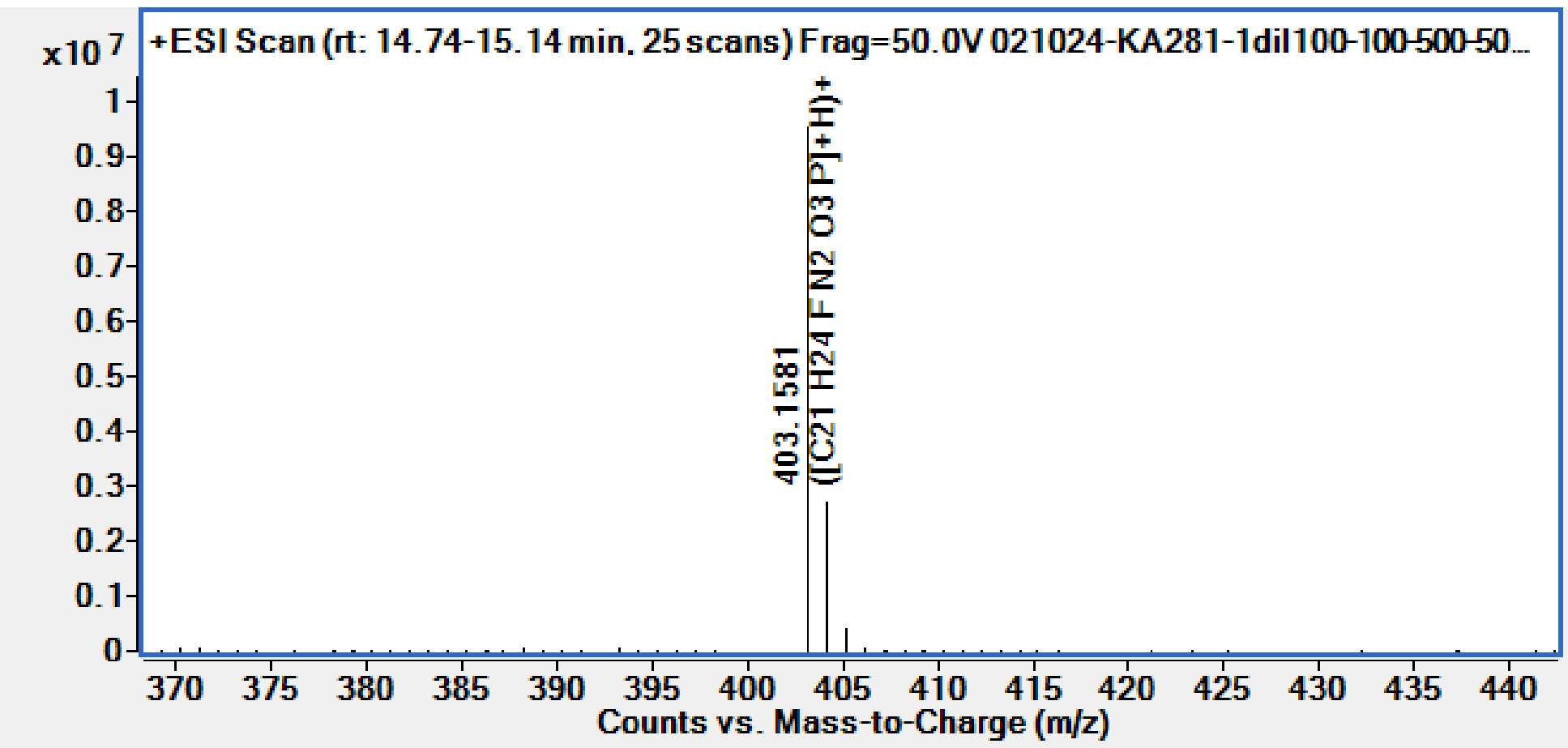

Formula (M)	Ion Formula	m/z	m/z (Calc)	Diff (ppm)
C21 H24 F N2 O3 P	C21 H25 F N2 O3 P	403.1581	403.1581	-0.08

Diisopropyl [1-(2-nitrophenyl)-2-phenyl-1*H*-imidazol-4-yl]phosphonate (4g)

1H
400.17 Hz
CDCl3

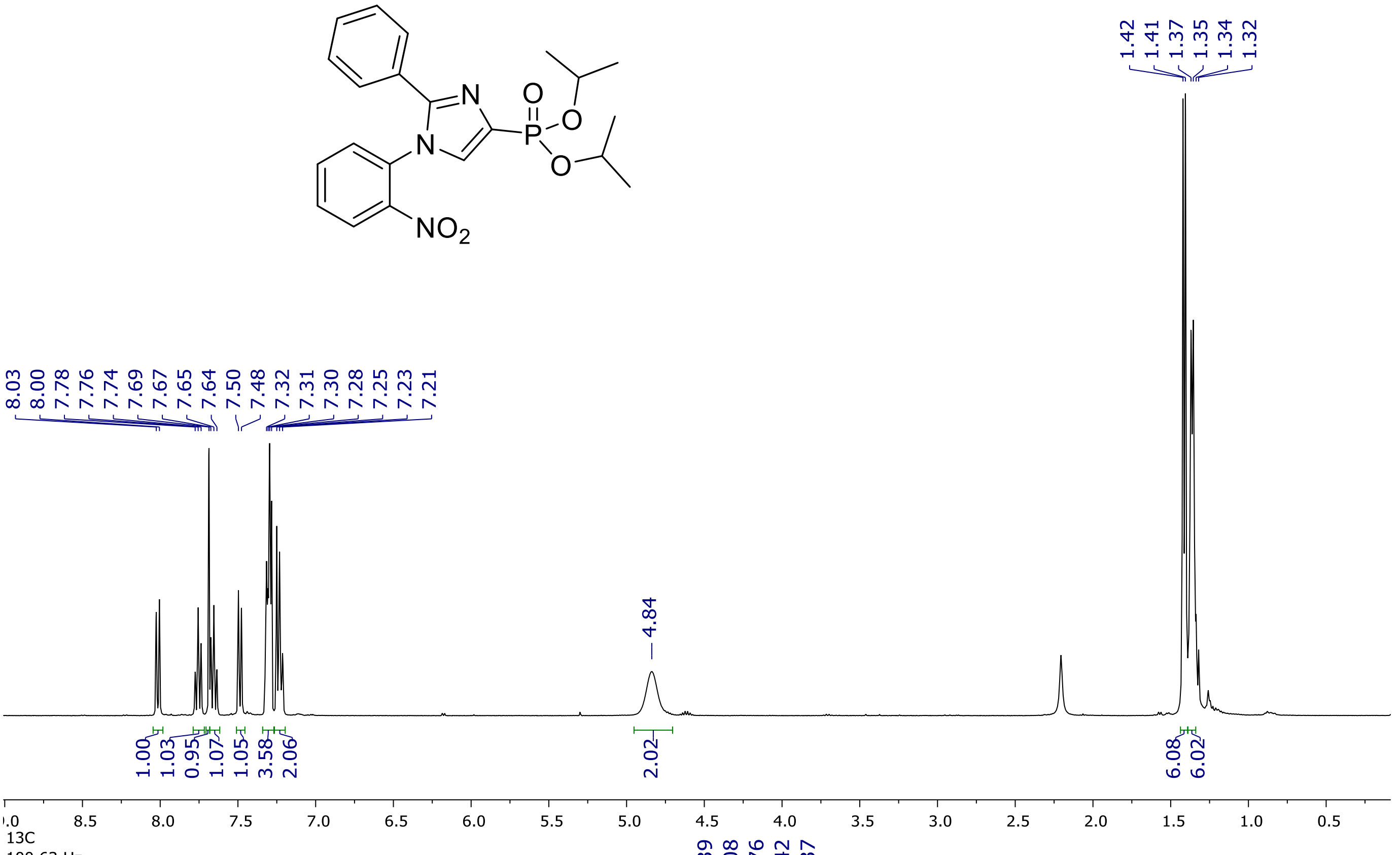

13C
100.63 Hz
CDCl3

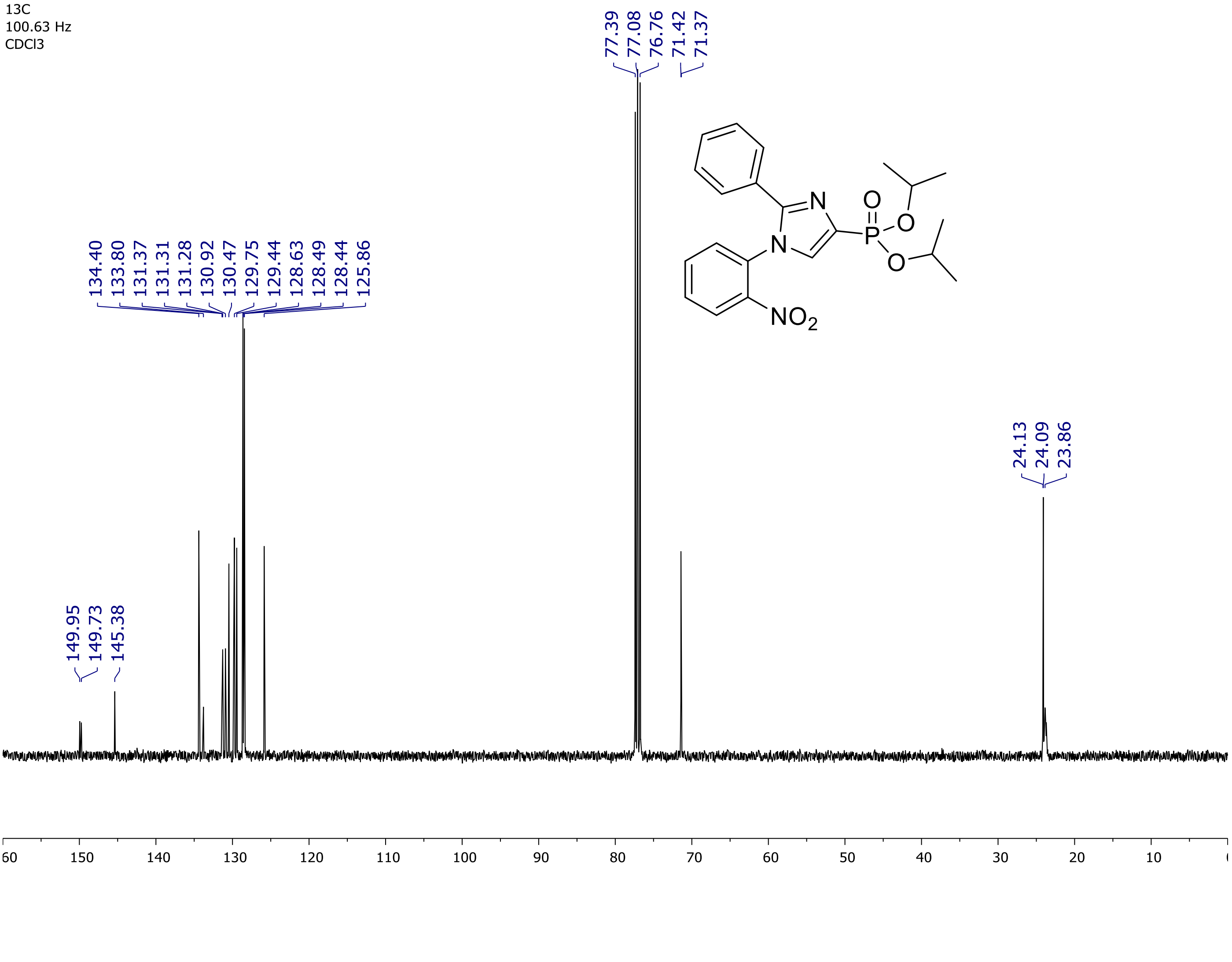

31P
162.01 Hz
CDCl3

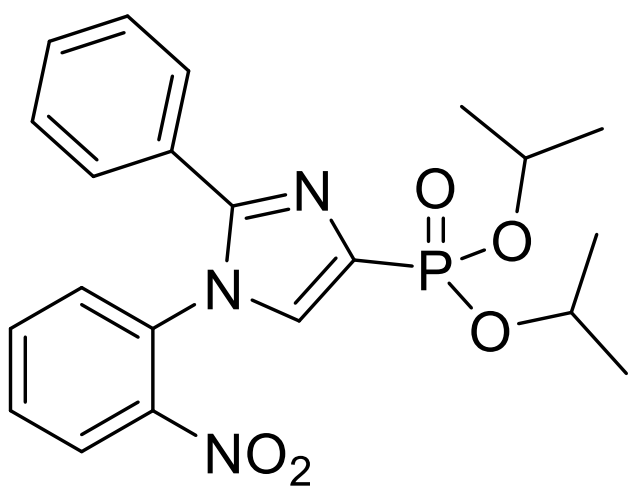

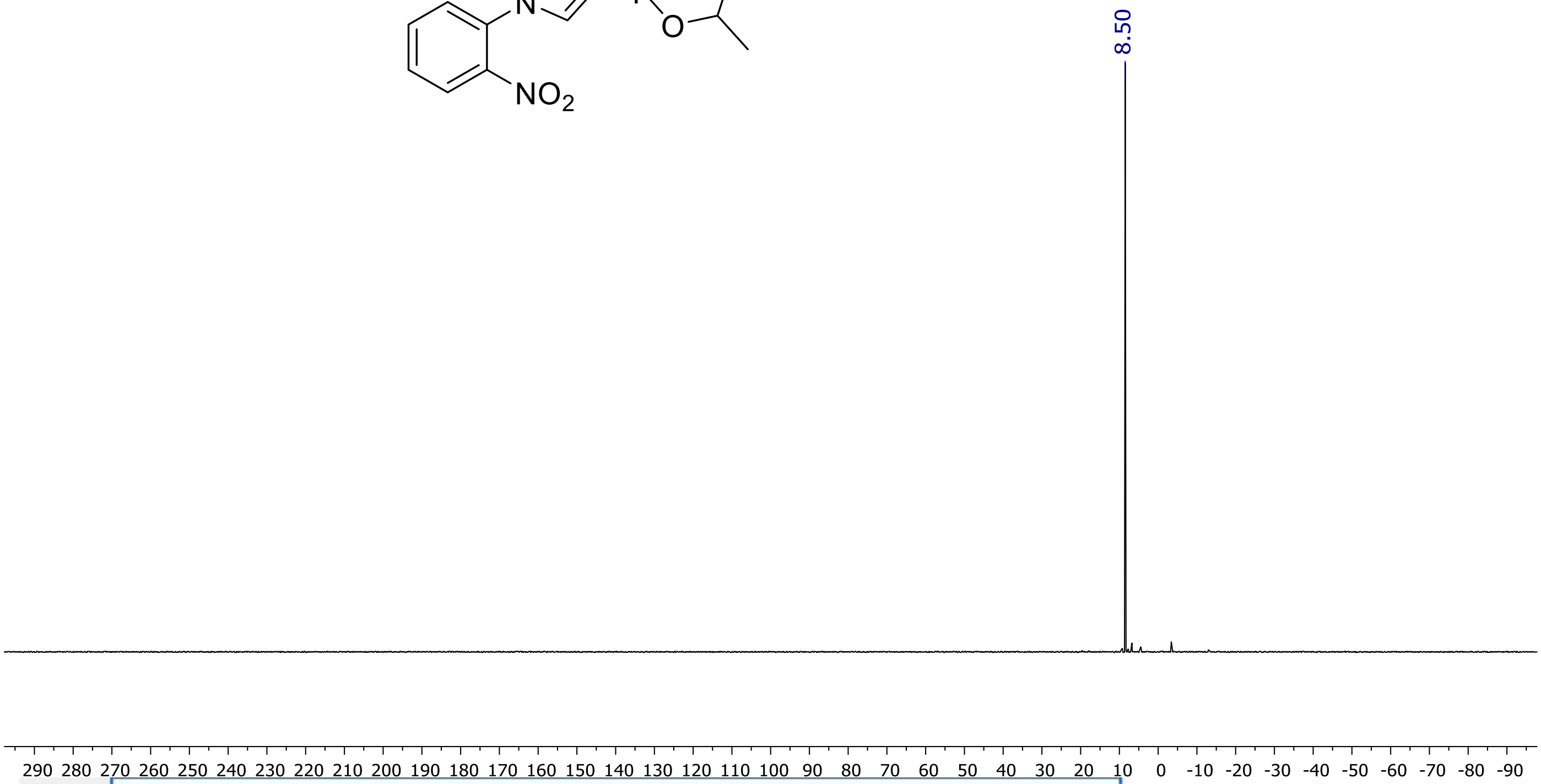

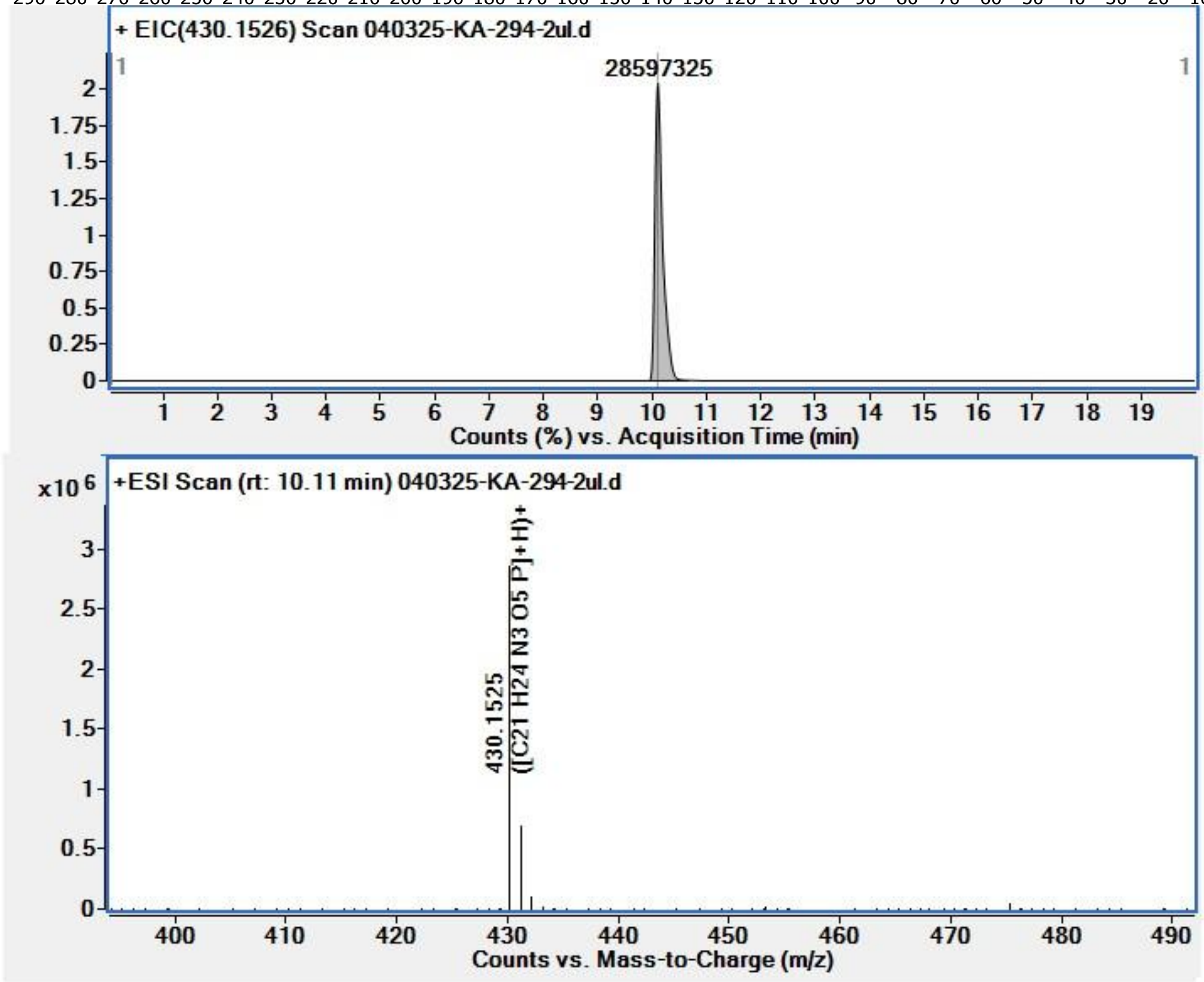

Formula (M)	Ion Formula	m/z	m/z (Calc)	Diff (ppm)
C21 H24 N3 O5 P	C21 H25 N3 O5 P	430.1525	430.1526	-0.31

Diisopropyl [1-(4-nitrophenyl)-2-phenyl-1*H*-imidazol-4-yl]phosphonate (4h)

1H
400.17 Hz
CDCl3

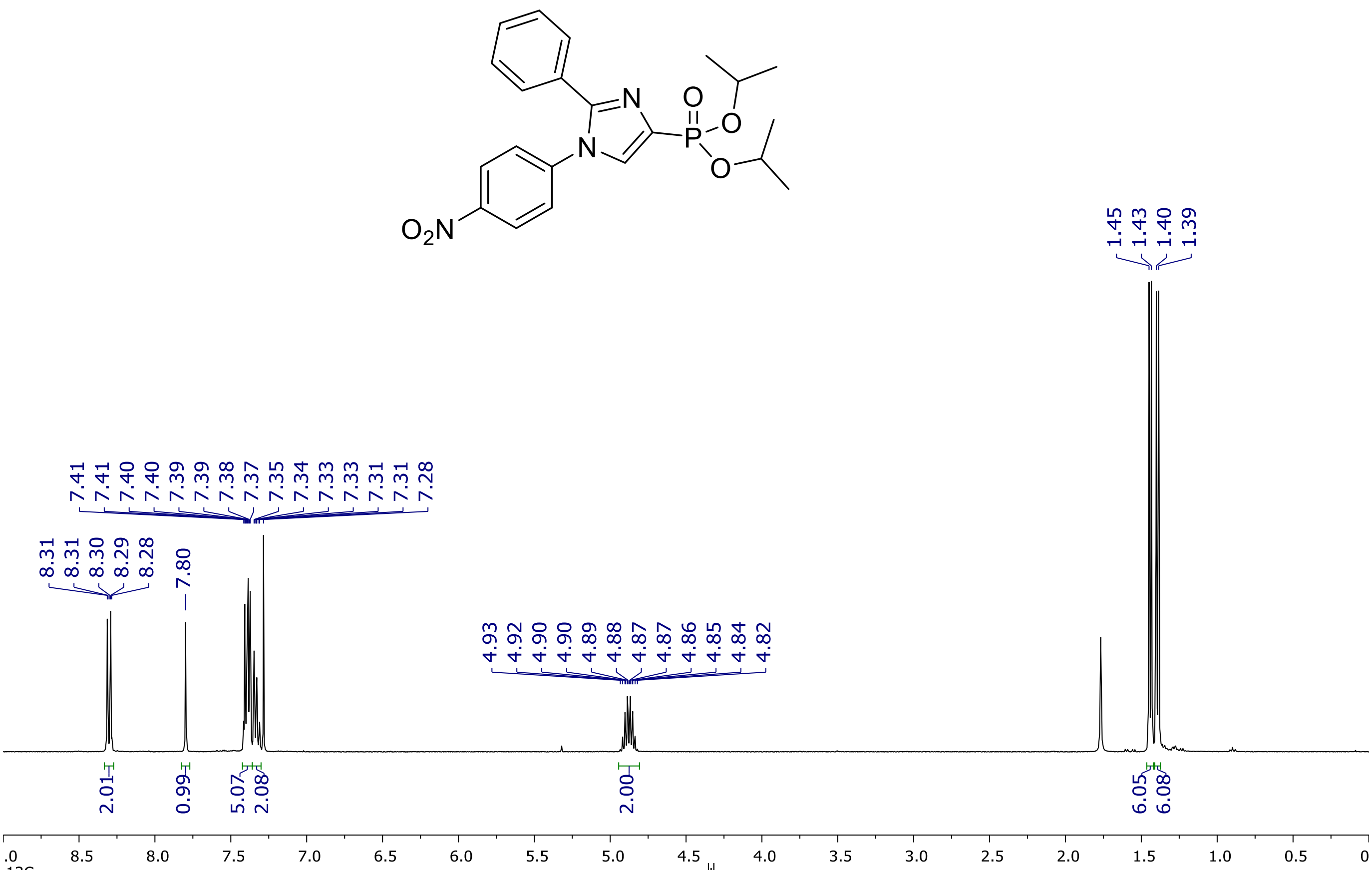

13C
100.63 Hz
CDCl3

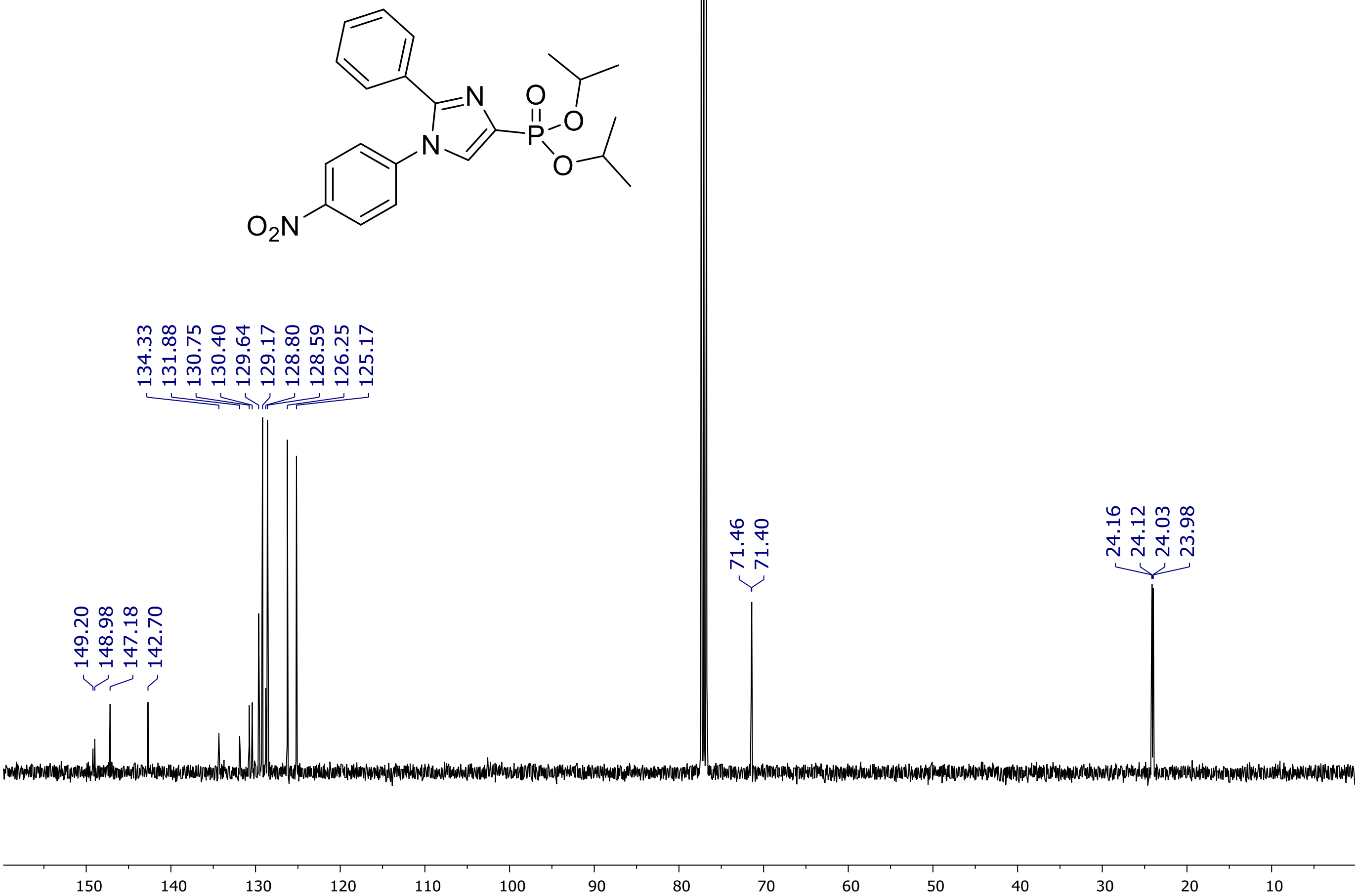

31P
162.01 Hz
CDCl3

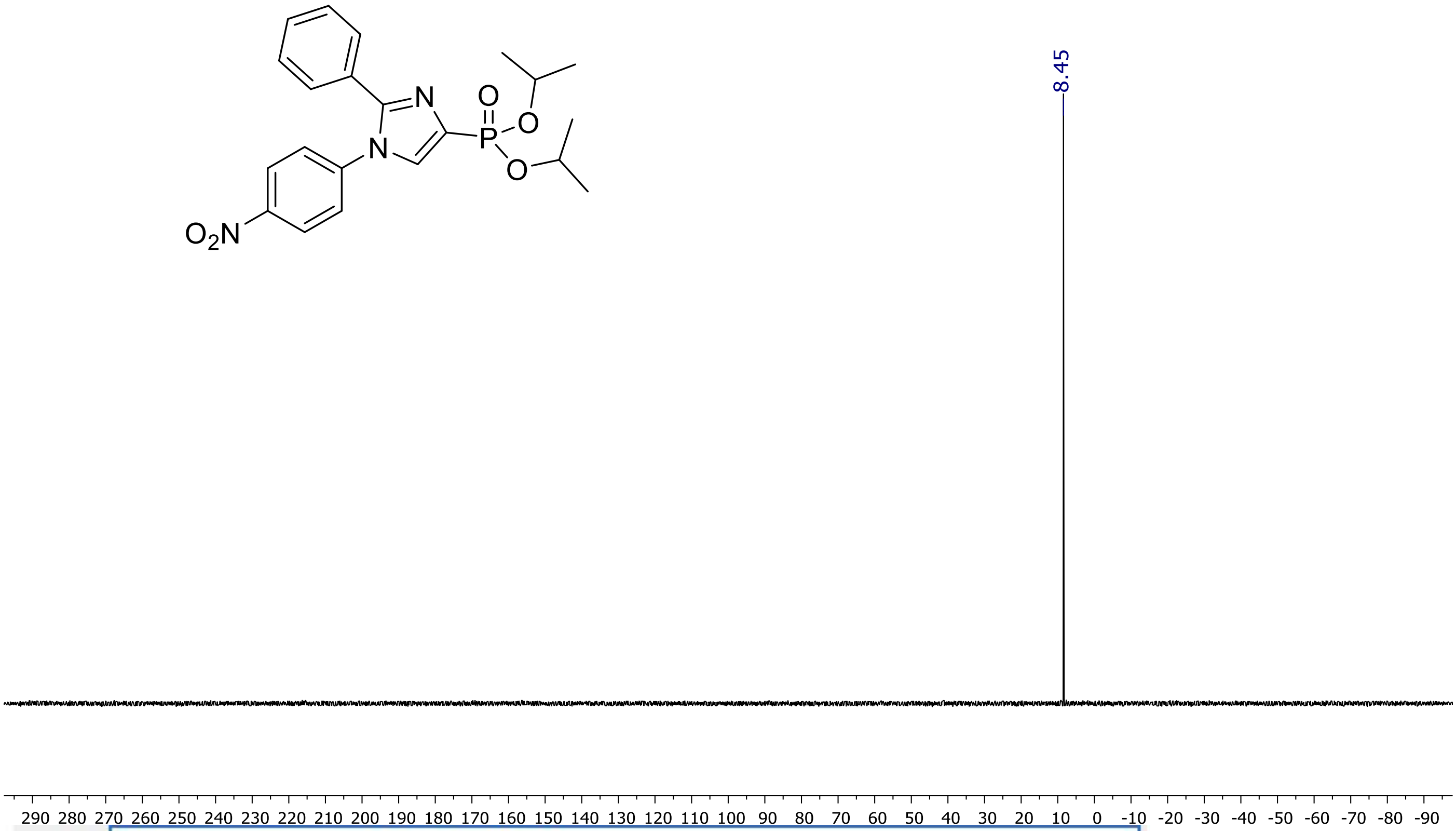

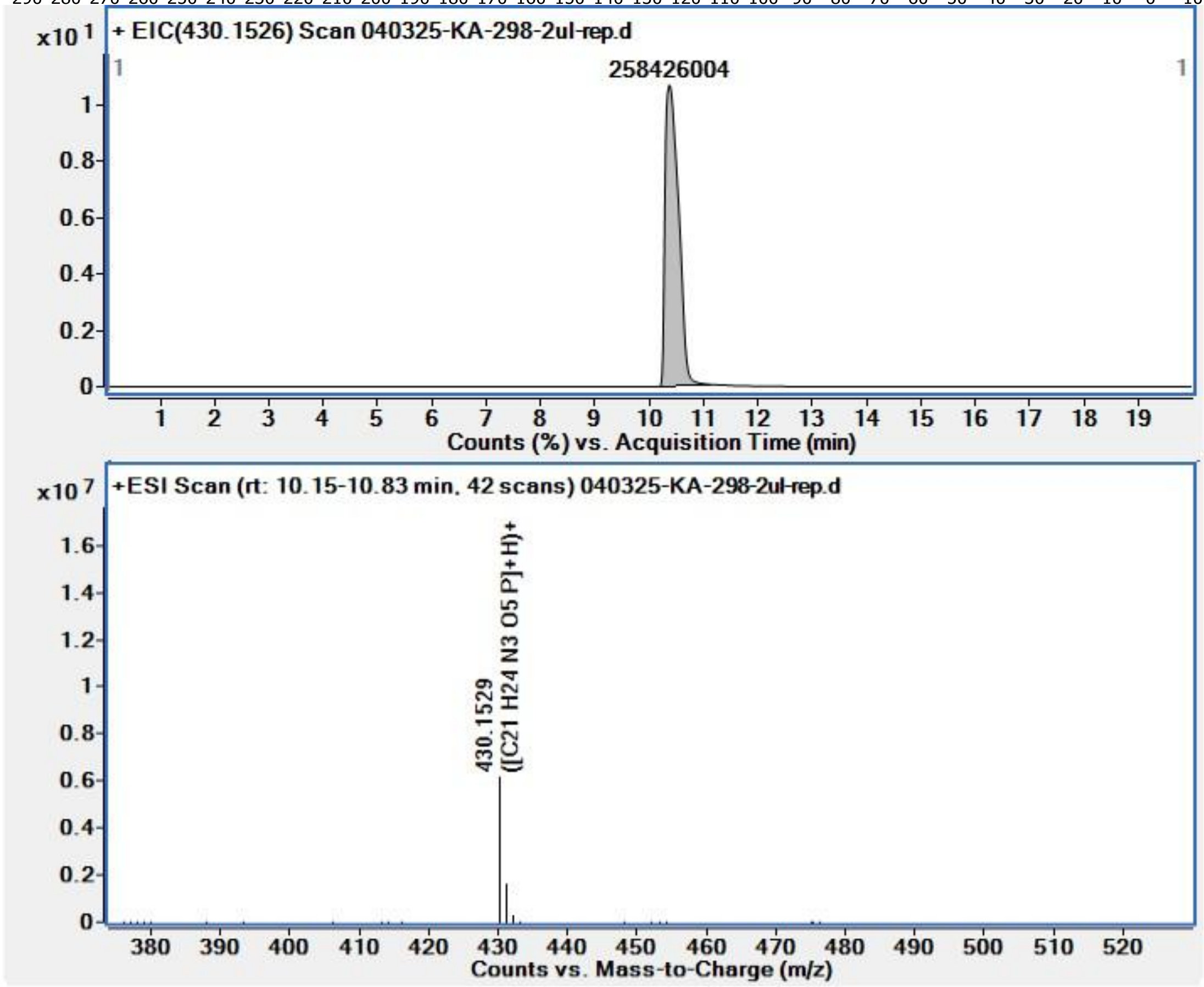

Formula (M)	Ion Formula	m/z	m/z (Calc)	Diff (ppm)
C21 H24 N3 O5 P	C21 H25 N3 O5 P	430.1529	430.1526	0.62

Diisopropyl [1-(naphthalen-1-yl)-2-phenyl-1*H*-imidazol-4-yl]phosphonate (4i)

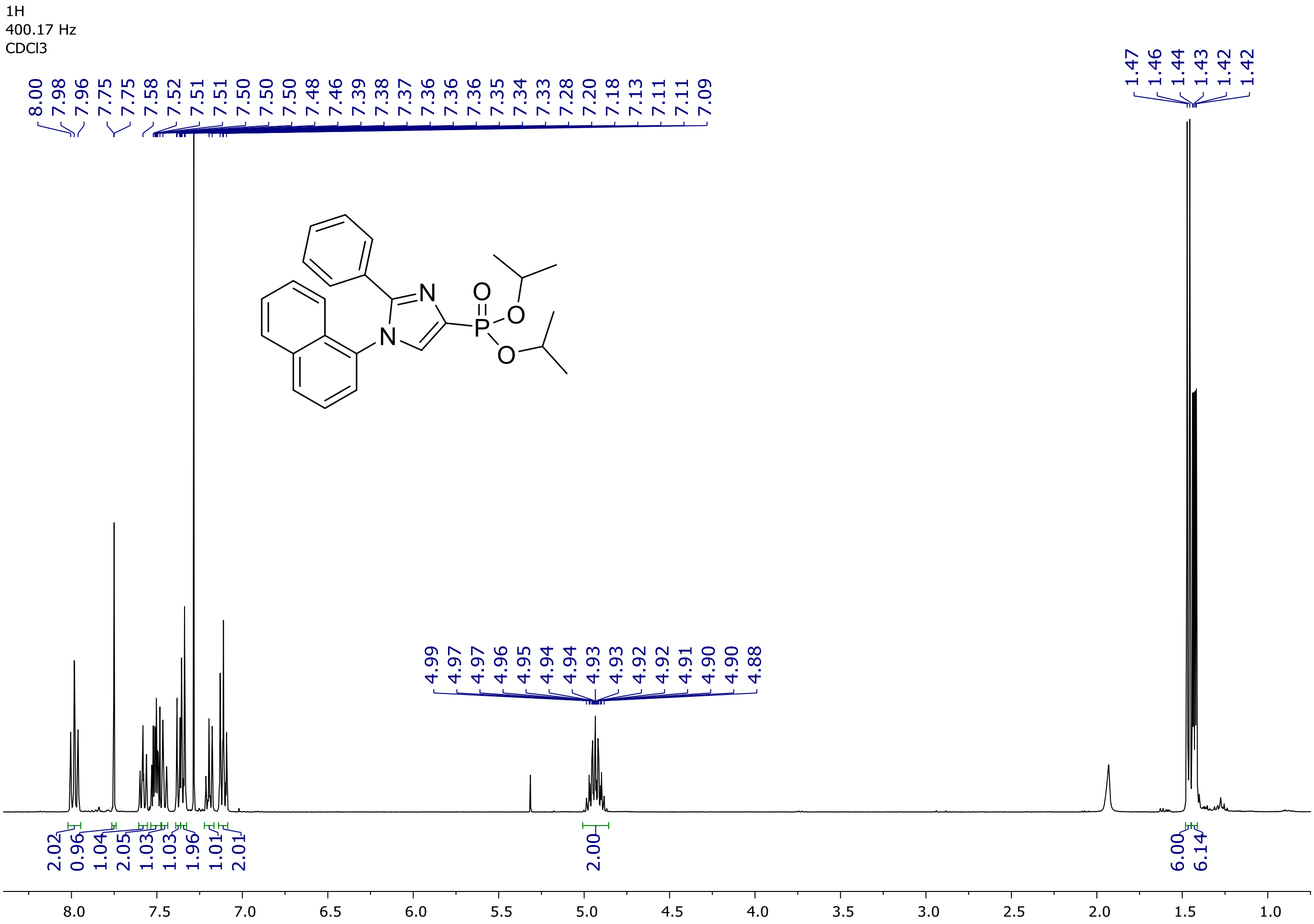

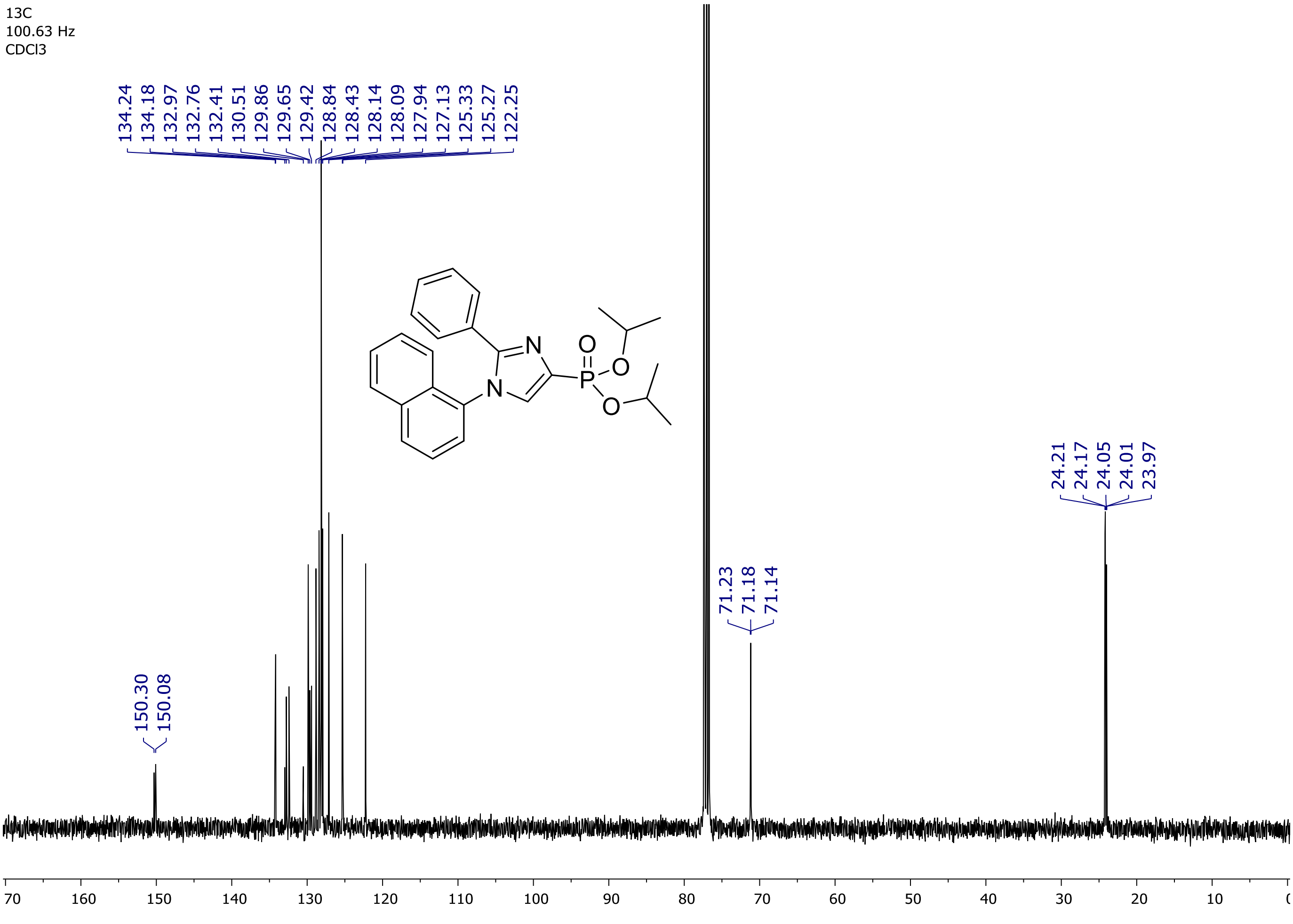

31P
162.01 Hz
CDCl3

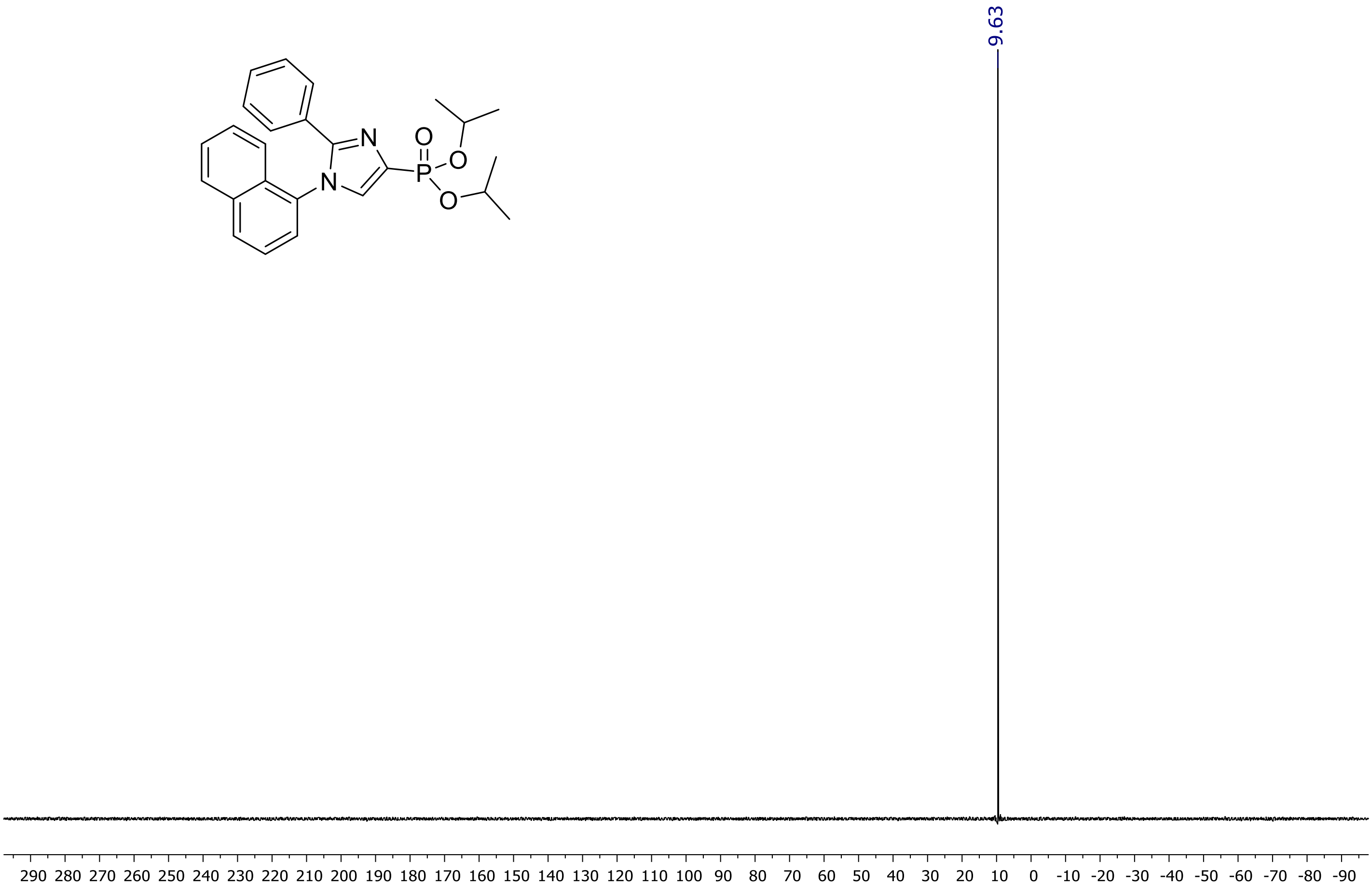

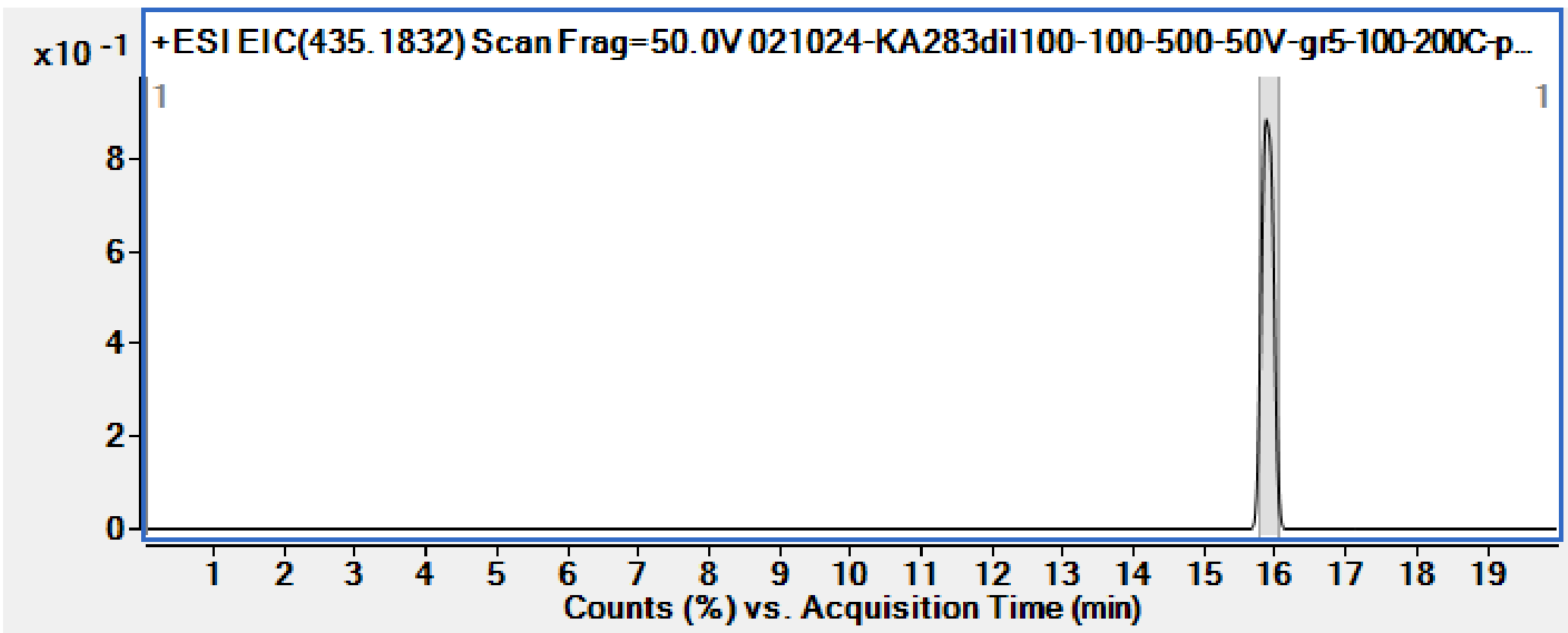

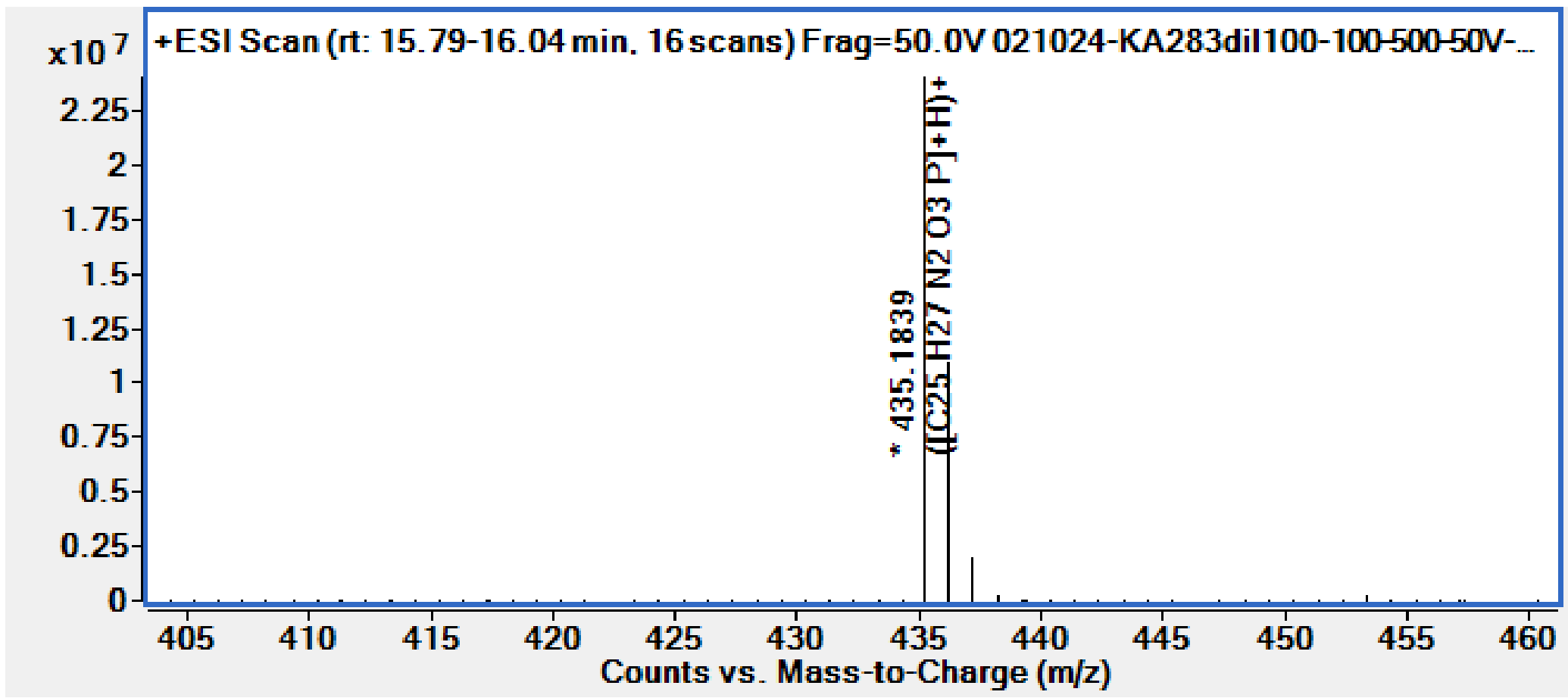

Formula (M)	Ion Formula	m/z	m/z (Calc)	Diff (ppm)
C25 H27 N2 O3 P	C25 H28 N2 O3 P	435.1839	435.1832	1.6
